# Total Synthesis of the Oligostilbenes Anigopreissin A and Fuliginosin A

**DOI:** 10.1021/acs.orglett.5c05397

**Published:** 2026-02-16

**Authors:** Aldahir Ramos Orea, Valeri Martínez-Barrita, Arturo Mejía-Galindo, Rubén O. Torres-Ochoa

**Affiliations:** Departamento de Química Orgánica, Instituto de Química, 7180 Universidad Nacional Autónoma de México, Circuito Exterior, Ciudad Universitaria, Coyoacán, Ciudad de México 04510, México

## Abstract

A convergent synthesis of the natural stilbenes anigopreissin A and fuliginosin A is presented. The key step involves a copper-catalyzed heteroannulation between an oxime acetate and a 4-substituted 1,3-cyclohexanedione, yielding a suitably substituted benzofuran-4-one via a mechanism that does not involve radicals. The use of methoxymethyl (MOM) ethers as protecting groups ensures the expedited completion of the sequence. The scope established for the heterocyclization suggests that this approach may be adapted to synthesize additional stilbenes.

## Graphical abstract

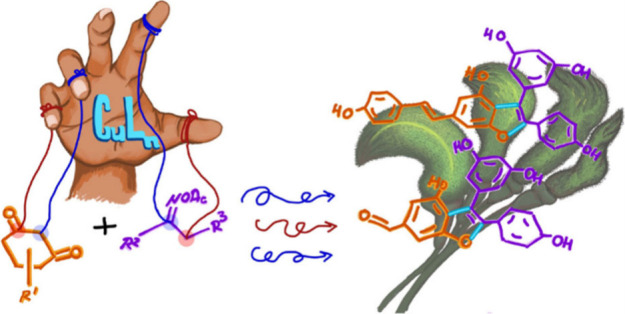

The 2,3-diarylbenzofuran system **1** is recognized as a significant scaffold embedded within a notable series of bioactive secondary metabolites, including oligostilbenes. Structurally, the members of this group of natural products are classified as polyphenols, as they originate from the oxidative coupling of up to four resveratrol units. The most prevalent oligomers formed from resveratrol are dimers, for example gnetuhainin B **2**,^1^ viniferifuran **3**,^2,3^ malibatol A **4**,^4^ diptoindonesin G **5**,^5^ anigopreissin A **6**,^6^ and fuliginosin A **7** ^7^ (Figure 1). In particular, the last two of these oligostilbenes, **6** and **7**, are the only resveratrol dimers reported to date that have a hydroxyl group at C-4.

**Figure 1:**
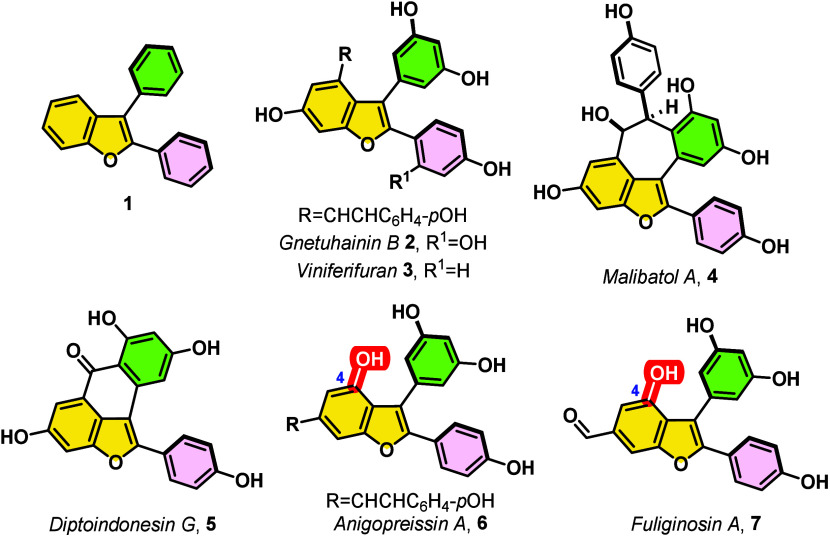
Examples of oligostilbenes derived from resveratrol.

The benzofurans **6** and **7** have been reported to have several biological effects;^8–10^ however, their potential remains relatively unexplored compared with the extensive biological research conducted on their parent compound, resveratrol,^11–14^ or on other resveratrol dimers.^15–17^ To date, the primary obstacles hindering the biological assessment of anigopreissin A and fuliginosin A have been their limited availability in natural sources^6,7^ and the inefficiency of the few reported syntheses for these compounds.^18–20^ In fact, although numerous synthetic efforts have been made to synthesize **6** and **7**,^20,21^ only three lengthy and problematic linear syntheses have been described, which can be classified according to the key step in the synthesis. Elofsson reported two synthetic methods based on a *one-pot* palladium-catalyzed Sonogashira/annulation process,^18,19^ whereas Liu utilized an innovative rhodium-mediated carbonylative benzoannulation^20^ (Scheme 1). In particular, the late-stage installation of the aryl substituent at C-3 and the alkenyl moiety on the preactivated benzofuran nuclei **8** and **9** substantially contributes to the high number of steps. Currently, a convergent synthetic pathway for metabolites **6** and **7** remains elusive.

**Scheme 1:**
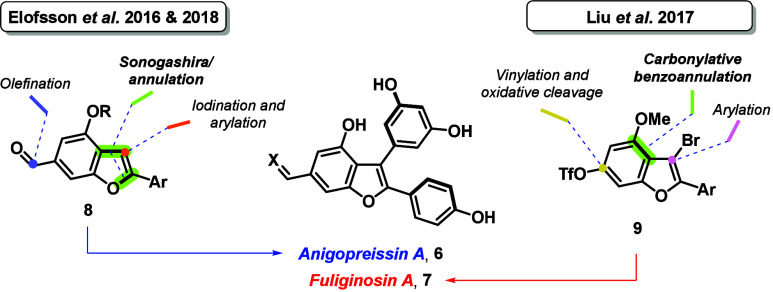
Reported Strategies for the Synthesis of Natural Products **6** and **7**

Given the limited utility of the reported syntheses of the oligostilbenes **6** and **7**, we directed our efforts toward developing an alternative convergent synthetic route to these compounds. We sought to develop a method that, in addition to rapidly and efficiently affording these compounds, could be easily adapted to the synthesis of various analogs and other structurally related natural compounds by simply substituting the substrates involved in the key step. Given that assembling a benzofuran intermediate with all the necessary substituents in a single step may present significant challenges, we deemed that preparing an appropriately substituted surrogate *O*-heterocyclic core, rather than the benzofuran ring itself, would be a more practical strategy. This approach aims to decrease the total number of steps in the synthesis.

In light of the preceding considerations, we proposed the retrosynthetic plan depicted in Scheme 2, which postulates that natural product **7** might be obtained directly from anigopreissin A **6** through oxidative cleavage. In turn, **6** may be derived from benzofuran-4-one **10**, a suitably substituted surrogate intermediate whose carbonyl group would be leveraged to appropriately position the C-4 hydroxy group found in the target natural products and to facilitate the aromatization of the cyclohexenone ring. Although several methodologies have been reported for preparing benzofuran-4-ones,^22^ we opted to synthesize the fused furan **10** following Yang’s copper-catalyzed heteroannulation between the oxime acetate **11** and 1,3-cyclohexanedione **12**.^23^ In our opinion, among the reported protocols for synthesizing 2,3-diaryl­benzofuran-4-ones, Yang’s procedure is the most suitable owing to its wider scope. Successively, the parent ketone of the oxime acetate **11** may be obtained via Claisen condensation of the alkyl benzoate **14** and phenylacetate **15**, followed by decarboxylation. Meanwhile, the cyclic diketone/enol **12** can be prepared from the unsaturated methyl ketone **16** and dimethyl malonate **17** via a tandem Michael addition/cyclization.

**Scheme 2:**
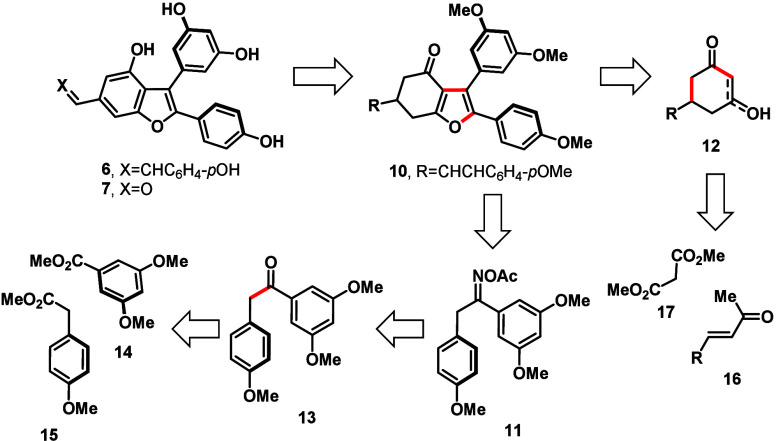
Retrosynthetic Proposal

First, we synthesized the starting materials required for Yang’s protocol. 1,3-Cyclohexanedione **12** was synthesized, predominantly as its enol tautomer, by reacting the methyl ketone **19** with dimethyl malonate, as previously described (Scheme 3a).^24^ In parallel, preparation of the ketone **13** was begun with the NaH-mediated condensation of esters **14** and **15**. Subsequently, the crude reaction mixture was hydrolyzed and decarboxylated, affording the desired ketone in only 20% yield (Scheme 3b).^25^ Because additional efforts to improve this outcome were unsuccessful, an alternative strategy was explored. Specifically, acetophenone **21**, synthesized from benzaldehyde **20** in two steps, was subjected to a palladium-catalyzed α-arylation with 4-iodoanisole.^26^ After a detailed screening of the conditions, the aryl ketone **13** was successfully isolated in 72% yield on a scale of up to 3.9 mmol.^27^ Lastly, the conversion of **13** into the ketoxime acetate **11** proceeded smoothly (Scheme 3c). Having synthesized the required substrates, the heteroannulation was performed according to Yang’s protocol. Disappointingly, the anticipated benzofuran-4-one **10** was not produced; instead, rapid degradation of compound **12**, used as the limiting reagent, was observed (Scheme 3d, entry 1). We thought that this outcome may have arisen because the radicals involved in the reaction may interact with the styryl moiety, altering the reaction pathway. Given that this step represents the key transformation in our plan, other reaction conditions described for a similar transformation were analyzed. Recently, our group described a divergent copper-catalyzed synthesis of medicinally important fused-furan molecules utilizing oxime acetates and active methylene compounds such as 4-hydroxyquinolinones/pyranones/coumarins.^28^ We conjectured that this reaction could be extended to the synthesis of benzofuran-4-ones if cyclic 1,3-diones are employed, notably through a markedly different mechanistic pathway that excludes the participation of radicals. Thankfully, this alternative heteroannulation afforded **10** in 50% yield. Furthermore, utilization of a terpyridine ligand improved the yield to 66%, with consistent results observed up to a scale of 2.3 mmol (60% yield) (Scheme 3d, entries 2 and 3).

**Scheme 3:**
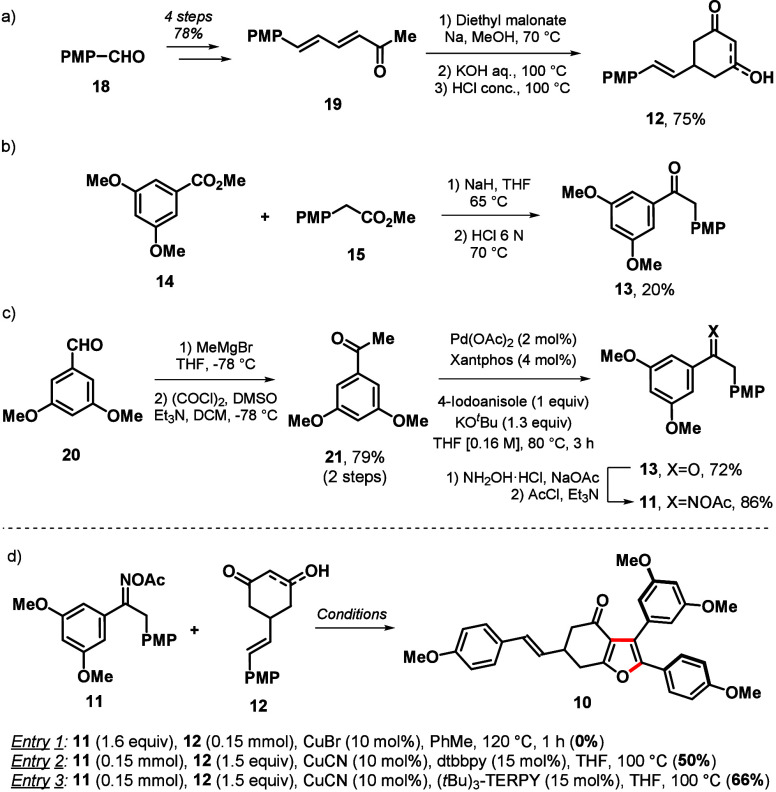
Preparation of the Starting Materials and Synthesis of the Key Benzofuran-4-one

Because our heteroannulation was considered a supplementary protocol to the previously reported procedure, we opted to assess its scope prior to finalizing the synthesis of **6**. For this task, 4,4′-di-*tert*-butyl-2,2′-dipyridyl (dtbbpy) was selected as the preferred ligand owing to its demonstrated efficacy in our previous research^28^ and its cost-effectiveness. Notably, in nearly all experiments, only 1.1 equiv of the cyclic 1,3-diketones were employed without negatively affecting the yields. Consequently, distinct oxime esters and cyclic 1,3-diketones were synthesized and reacted (Scheme 4). The reaction between propiophenone/acetophenone *O*-acetyl oximes and dimedone produced benzofuranones **24a**–**24e** with satisfactory yields, regardless of the substitution pattern on the aryl ring. The presence of an alkyl ester or an alkyl chloride attached to the oxime substrate was tolerated (**24f**, **24g**). Cyclic oxime esters derived from α–tetralone and cyclooctanone were converted to products **24h** and **24i**. However, the conversion of the tetralone-based substrate was less efficient, which was attributed to the premature partial hydrolysis of the *O*-acyl oxime substrate. Acetate acetoxime successfully yielded 2-unsubstituted benzofuran-4-one **24j** in 71% yield. As previously documented, the use of nonsymmetric 4,4-dimethylcyclohexan-1,3-dione produced a 1:1 separable regioisomeric mixture of benzofuranones **24k**/**24k′**.^23^ 4-Methyl cyclohexane-1,3-dione reacted with two different aryl ketoxime acetates to afford the *O*-heterocycles **24l** and **24m** in yields of 97% and 57%, respectively. Conversely, its reaction with *O*-acyl acetoxime resulted in the formation of the natural furanomonoterpene evodone **24n** in only 24% yield.^29^ We deduced that the low yield was primarily attributable to the volatility of the natural product; however, the participation of collateral processes, such as the direct conversion of the *O*-acetyl acetoxime to acetone, cannot be completely ruled out. The presence of unsaturated substituents in the dione, such as aryl or styryl groups, did not represent an obstacle, as evidenced by the yields obtained for the products **24o–24q**. A modest result was obtained when the reaction was conducted with a diketone bearing an ester functionality at *C*–4, which furnished the products **24r** and **24s**. In these reactions, the ester group underwent partial hydrolysis, thereby decreasing the amount of substrate available for heterocyclization, as the carboxylic acid was insoluble in THF. Thankfully, the cyclohexanone-1,3-dione reacted with various oxime acetates, furnishing benzofuran-4-ones **24t**–**24v** in yields ranging from 53% to 78%. Product **24v** was readily crystallized and subjected to X-ray diffraction analysis to conclusively confirm the regioselectivity of the transformation. The oxime esters derived from anti-inflammatory drugs such as indomethacin and nabumetone reacted with dimedone, affording the bis-heterocycle **24w** and naphthalene **24x** in 52% and 28% yield, respectively. An indolyl–furanoid scaffold has been identified within molecules exhibiting properties suitable for electroluminescent and photovoltaic materials,^30^ as well as in compounds with notable bioactivity profiles.^31^ Finally, when 1,3-cyclopentanedione and 1,3-cycloheptanedione were subjected to the optimized conditions, we noticed divergent behavior. The 5-membered diketone reacted with propiophenone oxime acetate as expected, giving the cyclopentafuran-4-one **25** in 53% yield. As a remark, this product could not be synthesized using Jian’s methodology.^23^ Interestingly, when the 7-membered diketone was used, the pyrrole-fused compound **26** was obtained rather than the anticipated benzofuran-4-one, albeit in only 27% yield. In this instance, we hypothesize that the desired pathway was outcompeted by a radical cross-coupling between an α-iminyl radical and the C-centered radical derived from cycloheptan-1,3-dione.

**Scheme 4:**
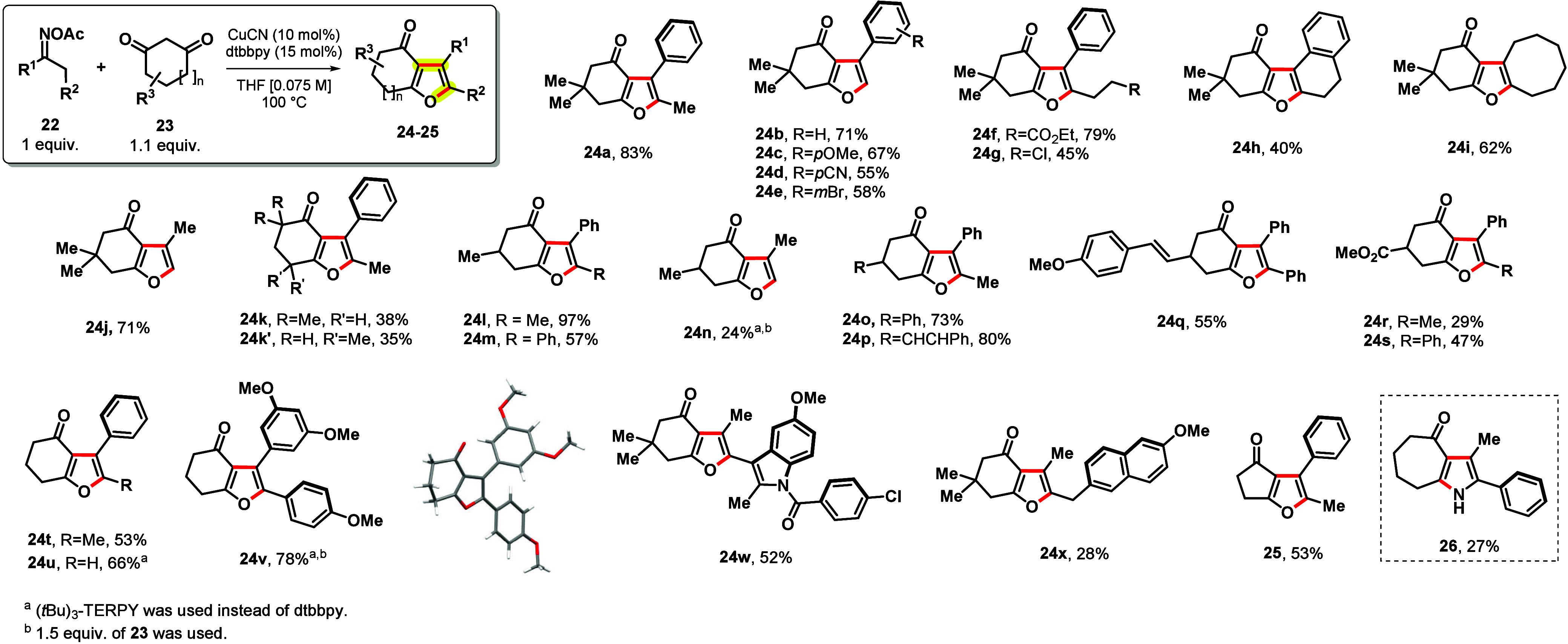
Scope of the Heteroannulation

Having established the reaction scope, we turned to the synthesis of **6** (Scheme 5). Initial attempts to carry out the oxidative aromatization of benzofuran-4-one **10** failed due to sensitivity concerns. Fortunately, 4-hydroxybenzofuran **27** could be prepared in 55% yield using 2,3-dichloro-5,6-dicyano-1,4-benzoquinone (DDQ) and anhydrous 1,4-dioxane. Conversely, the demethylation step did not proceed as projected; after trying several reaction conditions, natural polyphenol **6** could be isolated in only 15% yield using an excess of BCl_3_ in dichloromethane.^18^ Other demethylating reagents such as AlCl_3_ and LiCl were unproductive.^27^ The low efficiency of this step was consistent with previous observations for a similar transformation.^18–20^

**Scheme 5:**
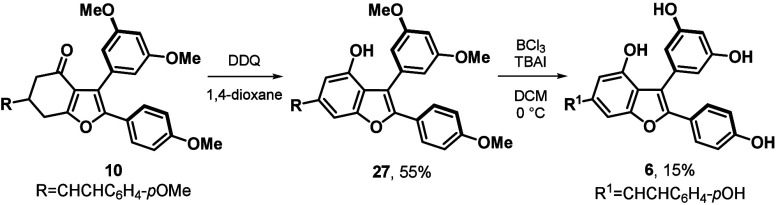
First Attempt to Synthesize Anigopreissin A

To address the problems encountered with the demethylation, we revised our proposal by protecting the phenol moieties with a more labile group; specifically, the MOM-protected compound **12a** and oxime acetate **11a** were proposed as more appropriate intermediates (Scheme 6). Cyclic compound **12a** was prepared using 4-hydroxybenzaldehyde **28** as the starting material, and its conversion into the unsaturated ketone **19a** required five steps.^27^ We sought to convert this ketone into the desired cyclohexanedione while minimizing the use of acid because of the lability of the protecting group. Meticulous experimentation enabled us to synthesize **12a** as a tautomeric keto–enol mixture (2:1) in 80% yield through a domino Michael addition/Dieckmann condensation/hydrolysis/​decarb­oxylation process (Scheme 6a). The synthetic pathway to the oxime ester **11a** required a significant redesign, which entailed the *O*-protection of α-resorcylic acid **30**, its subsequent conversion into the methyl ketone **21a**, and the α-arylation of this carbonyl compound under previously optimized reaction conditions. As anticipated, applying the oximation/acetylation sequence to **13a** proceeded without incident up to a scale of 2.6 mmol (Scheme 6b).

**Scheme 6:**
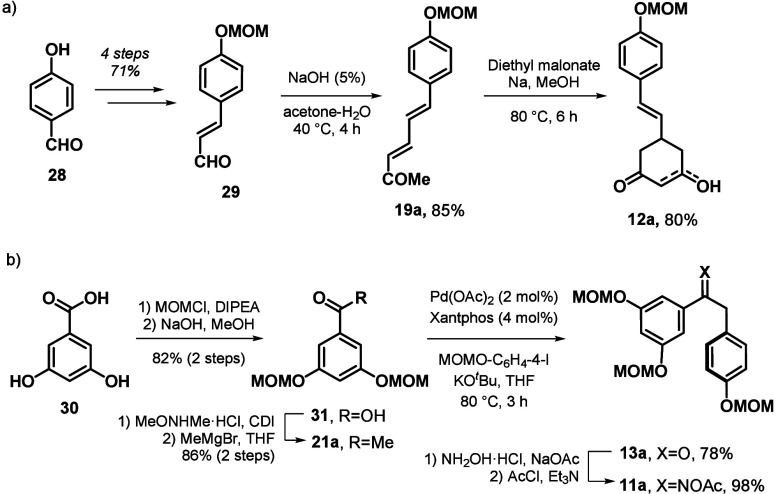
Synthesis of the MOM-Protected Substrates

With substrates **11a** and **12a** in hand, we subjected these compounds to heteroannulation, obtaining the intermediate **10a** in 60% yield (Scheme 7). The primary side-product of this reaction was the parent ketone of reactant **13a**, which was readily isolated and recycled via conversion into the oxime acetate **11a**. The aromatization of **10a** was performed with DDQ, yielding the expected 4-hydroxybenzofuran **27a** in 61% yield. In sharp contrast to the trimethoxy intermediate **27**, its MOM-analog **27a** was deprotected effortlessly using a solution of HCl in methanol, rendering anigopreissin A **6** in 85%. Lastly, fuliginosin A **7** was prepared in 30% yield from benzofuran **6** through oxidative cleavage of the alkenyl side chain using potassium osmate dihydrate.^20^ Considering that the free hydroxy groups in compound **6** might have affected the course of the alkene rupture, we applied the same transformation to the protected intermediate **27a**. Although the olefin cleavage took place in only 39% yield, the *O*-deprotection reaction occurred efficiently, providing fuliginosin A **7** in 77% yield.

**Scheme 7:**
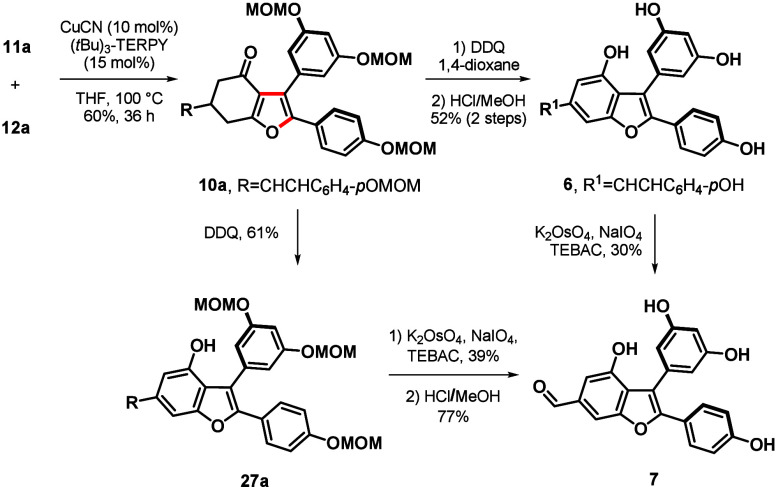
Completion of the Synthesis of the Natural Products **6** and **7**

In conclusion, a convergent total synthesis of the oligostilbene dimers anigopreissin A and fuliginosin A was successfully developed with overall yields of 13% and 4%, respectively. Central to this strategy is the identification of a reliable heteroannulation reaction that furnishes densely substituted benzofuran-4-ones from readily accessible oxime acetates and substituted 1,3-cyclohexanediones. The exclusion of radicals in the heterocyclization enhances its versatility and tolerance toward various functionalities. Furthermore, the use of MOM ether as a protecting group facilitates scalability and allows for straightforward late-stage deprotection. Overall, the developed approach not only streamlines access to 2,3-diarylbenzofuran metabolites but also provides a robust basis for future analog development, thereby expanding our understanding of the biological properties of this class of resveratrol dimers.

## Supplementary Material



## Data Availability

The data underlying this study are available in the published article and its Supporting Information.

## Notes

Experimental procedures, spectroscopic/spectrometric data of all new compounds (PDF)
