# Supplementary material for: Total Synthesis of the Oligostilbenes Anigopreissin A and Fuliginosin A
Source: Org Lett. 2026 Feb 16;28(22):6835–40. doi: 10.1021/acs.orglett.5c05397 (PMC13247974; doi:10.1021/acs.orglett.5c05397)

Supporting information

Total Synthesis of the Oligostilbenes Anigopreissin A and Fuliginosin A

Aldahir Ramos Orea, Valeri Martínez-Barrita, Arturo Mejía-Galindo and Rubén O. Torres-Ochoa*
Instituto de Química, Universidad Nacional Autónoma de México, Circuito Exterior, Ciudad Universitaria, Coyoacán, Ciudad de México, 04510, México
E-mail: romar.torres@iquimica.unam.mx, www.iquimica.unam.mx

Table of contents:

1. **General information** S2
2. **Total synthesis of anigopreissin A: First approach** S2
3. **General procedures** S9
4. **Synthesized substrates** S11
5. **Spectroscopic characterization of the heteroannulation products** S13
6. **Total synthesis of anigopreissin A and fuliginosin A: Second approach** S24
7. **Control experiments** S33
8. **Crystallographic data of benzofuran-4-one 24v** S33
9. **References** S35
10. **^{1}H and ^{13}C NMR spectra** S36

1. General Information

Commercially available chemicals were purchased from Merck and Sigma Aldrich and were used without further purification. ^{1}H NMR and ^{13}C NMR (300 and 75 MHz / 400 and 100 MHz / 500 and 125 MHz / 700 and 175 MHz) spectra were acquired in $CDCl_3$, CD_3OD, $(CD_3)_2CO$ or DMSO-d_6 at 25 °C on a 300 MHz Jeol Eclipse, 300 MHz Fourier Brucker, 300 MHz Avance Bruker, Bruker Avance III 400 MHz Bruker AscendTM (500 MHz), and Bruker AscendTM (700 MHz) spectrometers. The chemical shifts are reported on the δ scale in parts per million (ppm) and calibrated to residual solvents (7.26 ppm in $CDCl_3$, 2.50 ppm in DMSO-d_6, 3.31 ppm in CD_3OD, 2.05 ppm in $(CD_3)_2CO$) for ^{1}H NMR and (77.16 ppm in $CDCl_3$, 39.52 ppm in DMSO-d_6, 49.0 ppm in CD_3OD, 29.84 ppm in $(CD_3)_2CO$) for ^{13}C NMR. The peak shapes are indicated as follows: s, singlet; br s, broad singlet; d, doublet; t, triplet; q, quartet; quint, quintet; sext, sextet; sept, septet; m, multiplet. The coupling constant values (*J*) are reported in Hertz (Hz). Melting points were measured in open capillaries using a Mel-Temp apparatus and were not corrected. IR spectra were obtained using an FTIR Nicolet iS50 spectrometer of Thermo Scientific. Mass spectra were recorded with a JEOL SX 102 A spectrometer by electronic impact (EI) and a Jeol AccuTOF DART instrument for high-resolution measurements. Data collection was performed on a Bruker APEX-II CCD diffractometer using Mo-Kα radiation (λ= 0.7107 Å). Reactions were monitored by TLC and visualized using a dual shortwavelength/long-wavelength UV lamp. Flash column chromatography was carried out on silica gel 60 (230-400 mesh ASTM) from Macherey-Nagel GmbH & Co. All solvents were distilled under a nitrogen atmosphere. Dichloromethane was distilled from calcium hydride, and tetrahydrofuran was distilled from sodium benzophenone ketyl.

2. Total synthesis of anigopreissin A: First approach

Synthesis of the cyclic 1,3-diketone ***12***

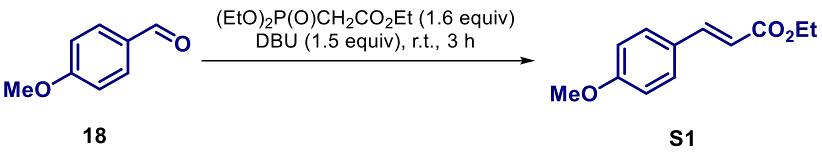

A mixture of anisaldehyde **18** (2.0 g, 14.69 mmol, 1.0 equiv), triethyl phosphonoacetate (4.6 mL ,23.50 mmol, 1.6 equiv), and DBU (3.3 mL, 22.03 mmol, 1.5 equiv) was stirred at room temperature for 3 h. The reaction was quenched with saturated NH_4Cl solution (15 mL). The aqueous layer was extracted with EtOAc (3 × 20 mL). The combined organic layers were dried over Na_2SO_4, filtered, and concentrated under reduced pressure. The crude α,β-unsaturated ester **S1** was used directly in the next step without purification.[1]

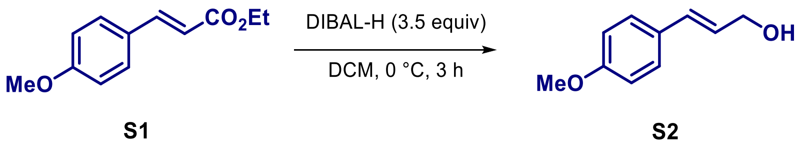

To a solution of the crude ester **S1** in anhydrous DCM (20 mL, 0.73 M), maintained under a nitrogen atmosphere at 0 °C, a 1 M DIBAL-H solution in DCM (51.4 mL, 51.41 mmol, 3.5 equiv) was added dropwise over 10 min. The reaction mixture was allowed to warm to room temperature and stirred for 3 h.

The reaction was then quenched with brine (50 mL) and extracted with EtOAc (3 × 50 mL). The combined organic layers were dried over Na_2SO_4, filtered, and concentrated under reduced pressure to afford the corresponding crude alcohol **S2**, which was used directly in the next step without purification.[2]

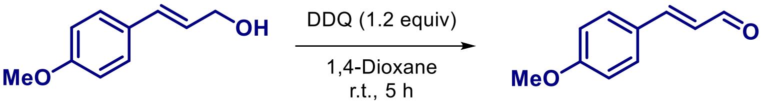

S2 **S3**

To a solution of the crude alcohol **S2** in 1,4-dioxane (20 mL, 0.73 M) was added DDQ (4.0 g, 17.63 mmol, 1.2 equiv) portionwise. The mixture was stirred for 5 h at room temperature and then concentrated under reduced pressure. The residue was dissolved in EtOAc (40 mL) and washed with saturated $NaHCO_3$ solution (3 × 20 mL). The organic layer was dried over Na_2SO_4, filtered, and concentrated under reduced pressure to provide the crude aldehyde **S3**. Since an NMR analysis showed an acceptable purity of the aldehyde, this material was used directly in the subsequent condensation step.

(*E*)-3-(4-Methoxyphenyl)acrylaldehyde (S3).[3] ^{1}H NMR ($CDCl_3$, 300 MHz): δ 9.65 (d, 1H , *J* = 7.8 Hz), 7.52 (d, 2H, *J* = 8.5 Hz), 7.42 (d, 1H, *J* = 15.8 Hz), 6.94 (d, 2H, *J* = 8.9 Hz), 6.61 (dd, 1H, *J* = 15.8, 7.7 Hz,), 3.86 (s, 3H).

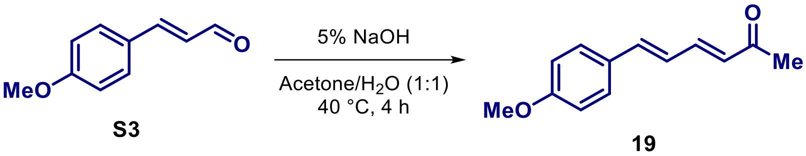

S3 **19**

To a solution of the crude aldehyde **S3** in 16 mL (0.92 M) of a mixture of acetone/water (1:1), a 5% NaOH solution (5 mL) was added dropwise. The reaction mixture was heated at 40 °C for 4 h using a hot plate stirrer and an aluminum block. After completion, the reaction was quenched with 1 N HCl until a pH ≈ 6 was reached, followed by the addition of brine (15 mL). The aqueous layer was extracted with EtOAc (3 × 30 mL). The combined organic layers were dried over anhydrous Na_2SO_4, filtered, and concentrated under reduced pressure. The residue was purified by flash column chromatography using silica gel with 25% EtOAc-hexane as eluent to afford methyl ketone **19**.

(3*E*,5*E*)-6-(4-Methoxyphenyl)hexa-3,5-dien-2-one (19).[4] White solid, 2.32 g (78%). **^{1}H NMR ($CDCl_3$, 300 MHz): δ** 7.42 (d, 2H, *J* = 8.5 Hz), 7.28 (dd, 1H, *J* = 15.4, 10.6 Hz), 7.93-6.88 (m, 3H), 6.76 (ddd, 1H, *J* = 15.4, 10.6, 0.7 Hz), 6.22 (d, 1H, *J* = 15.4 Hz), 3.83 (s, 3H), 2.31 (s, 3H).

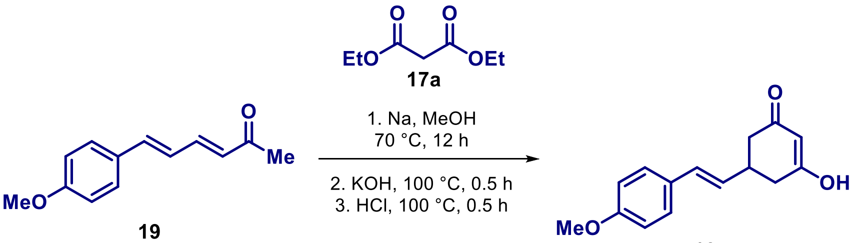

19 **12**

Compound **12** was synthesized following the protocol described by Hayashi and co-workers[4] using 2.47 mmol of methyl ketone **19**. It was isolated predominantly as the enol tautomer.

(*E*)-5-(4-Methoxystyryl)cyclohexane-1,3-dione (12).[4] Orange solid, 453 mg (75%). **^{1}H NMR (DMSO-*d*$_6$, 300 MHz): δ** 11.11 (br s, 1H), 7.32 (d, 2H, J = 8.8 Hz), 6.88 (d, 2H, J = 8.9 Hz), 6.38 (d, 1H, J = 16.1 Hz), 6.13 (dd, 1H, J = 16.0, 6.8 Hz), 5.24 (s, 1H), 3.74 (s, 3H), 2.86 (td, 1H, J = 10.1, 5.0 Hz), 2.43-2.24 (m, 4H).

*Synthesis of the α-arylacetophenone **13** via a Claisen condensation*

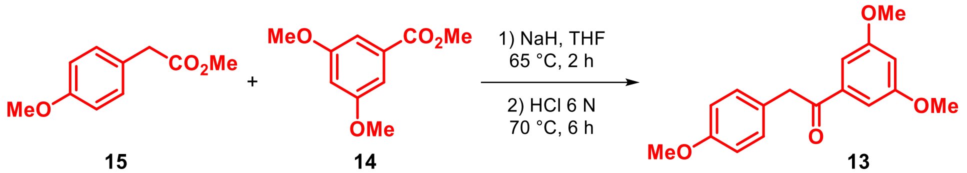

This compound was synthesized following the procedure reported in the patent by Wiffen and McCague (WO 2008012108),[5] with some modifications:

1) A solution of methyl 4-methoxyphenyl acetate **15** (0.35 mL, 2.2 mmol, 1 equiv) in 4 mL (0.55 M) of THF was added dropwise over 6 h to a suspension of methyl 3,5-dimethoxybenzoate **14** (0.56 g, 2.86 mmol, 1.3 equiv) and NaH (60% in mineral oil, 0.24 g, 5.94 mmol, 2.7 equiv) in 4 mL dry THF at 65 °C using a hot plate stirrer and an aluminum block. Afterwards, the resulting mixture was stirred at the same temperature for 2 h instead of overnight. Under these conditions, the starting materials were no longer detected by TLC.
2) The hydrolysis/decarboxylation step was performed using 6 N HCl rather than a15% aqueous HCl solution.

1-(3,5-Dimethoxyphenyl)-2-(4-methoxyphenyl)ethan-1-one (13). White solid, 126 mg (20%), mp 87 °C. **^{1}H NMR (CDCl$_3$, 300 MHz): δ** 7.18 (d, 2H, J = 8.8 Hz), 7.14 (d, 2H, J = 2.3 Hz), 6.86 (d, 2H, J = 8.8 Hz), 6.63 (t, 1H, J = 2.3 Hz), 4.18 (s, 2H), 3.82 (s, 6H), 3.78 (s, 3H). **^{13}C{^{1}H} NMR (CDCl$_3$, 75 MHz): δ** 197.8, 161.0, 158.7, 138.7, 130.5, 126.6, 114.3, 106.6, 105.4, 55.7, 55.4, 44.9. **IR (ATR) ($υ_{max}$, cm^{-1}):** 2934.73, 1592.13, 1510.00, 1203.35, 1154.90. **HRMS (DART) *m/z*:** $[M + H]^+$ calcd for $C_{17}H_{19}O_4$ 287.1283; found 287.1275.

Due to the hydrolysis of both methyl 3,5-dimethoxybenzoate **14** and methyl 4-methoxyphenyl acetate **15** observed while using the conditions reported in the patent, a brief evaluation of alternative bases and solvents was conducted to determine whether milder conditions could suppress this issue (**Table S1**). Only NaH in refluxing THF afforded the β-ketoester **S4**. When the combination NaH/THF was used at 0 °C or at –78 °C, no reaction was observed, and the starting materials remained unchanged. In contrast, the use of KO*t*Bu/*t*BuOH or NaOMe/MeOH resulted in the hydrolysis of both starting materials.

Table S1. Screening of base/solvent systems for the formation of β-ketoester ***S4***

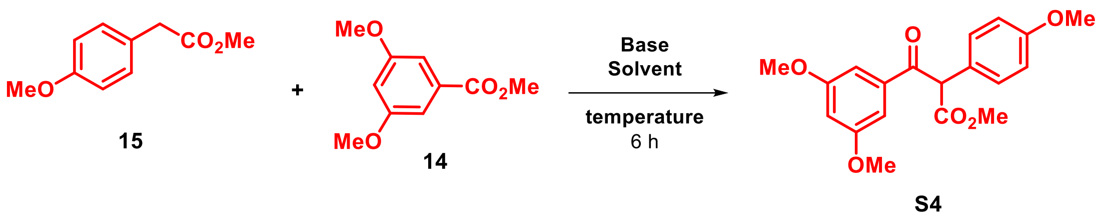

Entry	Base/Solvent	Temperature °C	Result
1[a]	NaH/THF	66	**S4** (22%)
2[b]	NaH/THF	0	N.R.
3	*n*BuLi/THF	-78	N.R.
4	KO*t*Bu/*t*BuOH	0 → 66	Hydrolysis of the starting materials
5	NaOMe/MeOH	0 → 66	Hydrolysis of the starting materials

Reaction conditions: **15** (832 µmol), **14** (998 µmol), and base (1.66 mmol) under a nitrogen atmosphere in anhydrous solvent. N.R. = No reaction. [a] Isolated yield. [b] 24 h instead of 6 h.

Methyl 4-(3,5-dimethoxyphenyl)-3-(4-methoxyphenyl)-2,4-dioxobutanoate (S4).[6] Colorless oil, 63 mg (22%). **^{1}H NMR ($CDCl_3$, 300 MHz): δ** 7.31 (d, 2H, *J* = 8.8 Hz), 7.08 (d, 2H, *J* = 2.2 Hz), 6.89 (d, 2H, *J* = 8.8 Hz), 6.61 (t, 1H, *J* = 2.3 Hz), 5.51 (s, 1H), 3.78 (s, 6H), 3.78 (s, 3H), 3.75 (s, 3H).

Synthesis of the α-arylacetophenone ***13*** *via a α-arylation*

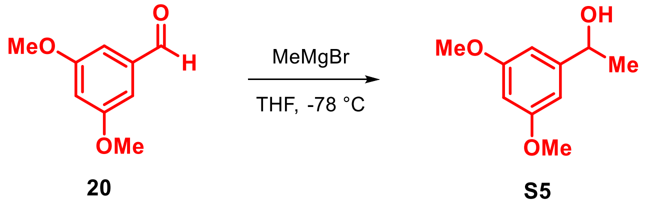

Under a nitrogen atmosphere, a solution of 3,5-dimethoxybenzaldehyde **20** (4.0 g, 24 mmol, 1 equiv) in anhydrous THF (20 mL, 1.2 M) was cooled to −78 °C. To this solution, 12 mL of MeMgBr was slowly added (3 M, 36.1 mmol, 1.5 equiv). The mixture was stirred for 3 h at −78 °C and then 2 h at room temperature. The solvent was removed *in vacuo*, and the residue was extracted with EtOAc (3 × 30 mL) using brine (20 mL) as the aqueous phase. The combined organic layers were dried over Na_2SO_4, filtered, and concentrated *in vacuo*. The crude residue was purified by flash column chromatography on silica gel with 30% EtOAc-hexane as eluent.

1-(3,5-Dimethoxyphenyl)ethan-1-ol (S5).[7] Colorless oil, 4.006 g (91%). **^{1}H NMR ($CDCl_3$, 400 MHz): δ** 6.53 (d, 2H, *J* = 2.3 Hz), 6.36 (t, 1H, *J* = 2.3 Hz), 4.82 (q, 1H, *J* = 6.4 Hz), 3.79 (s, 6H), 1.47 (d, 3H, *J* = 6.5 Hz).

Table S2. *Scale-up of the addition of MeMgBr to aldehyde* **20** *to afford alcohol* **S5**

Entry	Scale (mmol)	Yield[a] (%)
1	1.81	97
2	24	91

[a] Isolated yield.

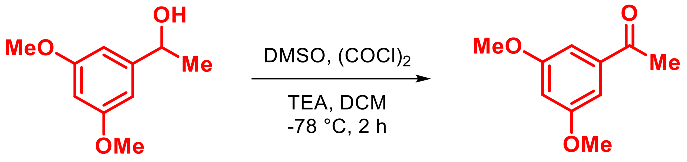

S5 **21**

To a solution of oxalyl chloride (2.8 mL, 33.04 mmol, 1.4 equiv) in dry DCM (10 mL) at −78 °C under a nitrogen atmosphere, DMSO (5.0 mL, 70.8 mmol, 3 equiv) was added dropwise over 10 min. The reaction mixture was stirred at −78 °C for 30 min. Afterwards, a solution of alcohol **S5** (4.3 g, 23.6 mmol, 1 equiv) in dry DCM (10 mL) was added dropwise at the same temperature, and the resulting suspension was stirred for an additional 30 min. Triethylamine (14.8 mL, 106.2 mmol, 4.5 equiv) was then added slowly, and the mixture was continued under stirring at −78 °C for 20 min. The reaction was quenched by the slow addition of saturated NH_4Cl solution (30 mL). The aqueous layer was extracted with DCM (3 × 40 mL), and the combined organic layers were washed with brine, dried over anhydrous Na_2SO_4, filtered, and concentrated *in vacuo*. The crude product was purified by flash column chromatography using silica gel with 20% EtOAc-hexane as eluent.

1-(3,5-Dimethoxyphenyl)ethan-1-one (21).[8] White solid, 1.10 g (87%), mp 42 °C. **^{1}H NMR ($CDCl_3$, 400 MHz): δ** 7.07 (d, 2H, J = 2.4 Hz), 6.63 (t, 1H, J = 2.4 Hz), 3.82 (s, 6H), 2.55 (s, 3H).

Table S3. *Effect of the aryl halide, equivalents of KO^{t}Bu, reaction temperature, and reaction time*

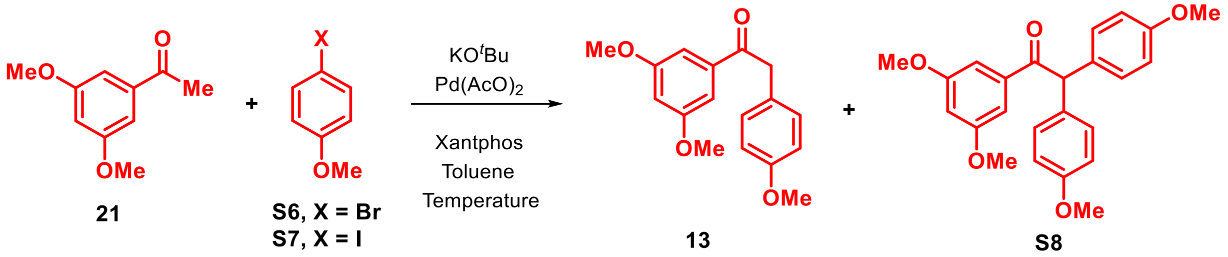

Entry	Aryl halide	KO^{t}Bu (Equiv)	Temperature (°C)	Time (h)	Yield[a] 13 : S8 (%)
1	**S6**	2	80	6	N.R.
2	**S7**	2	80	6	5 : 32
3	**S7**	2	25	6	N.R.
4	**S7**	1	80	6	46 : 8
5	**S7**	1.3	80	6	50 : 5
6	**S7**	1.3	80	3	52 : 0
7	**S7**	1	80	3	33 : 0

Reaction conditions: **21** (332 µmol), **S6** or **S7** (365 µmol), [Pd] 2 mol%, Xantphos (4 mol%) in 2 mL of anhydrous toluene (0.16 M) for 6 h. [a] Isolated yield. N.R. = No reaction.

Table S4. *Effect of base, temperature, and ligand*

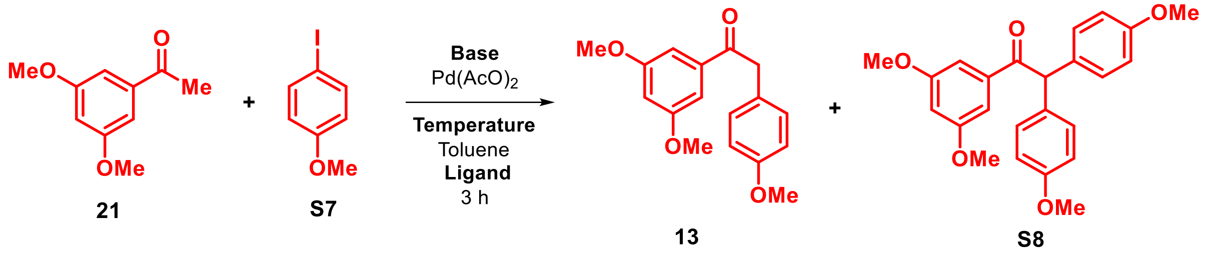

Entry	Base	Temperature (°C)	Ligand	Yield[a] 13 : S8 (%)
-	KO^{t}Bu	80	Xantphos	52 : 0
1	NaO^{t}Bu	80	Xantphos	17 : 0
2	Cs_2CO_3	80	Xantphos	N.R.
3	NaOMe	80	Xantphos	N.R.
4	NaO^{t}Bu	100	Xantphos	34 : 0
5	Cs_2CO_3	100	Xantphos	N.R.
6	NaOMe	100	Xantphos	N.R.
7	KO^{t}Bu	80	-	N.R.
8	KO^{t}Bu	80	BINAP	N.R.
9	KO^{t}Bu	80	DPPE	N.R.
10	KO^{t}Bu	80	DPPF	N.R.

Reaction conditions: **21** (332 µmol), **S7** (365 µmol), [Pd] 2 mol%, base (416 µmol), ligand (4 mol%) in 2 mL of anhydrous toluene (0.16 M) for 3 h. [a] Isolated yield. N.R. = No reaction.

Table S5. *Effect of stoichiometry, solvent, and concentration*

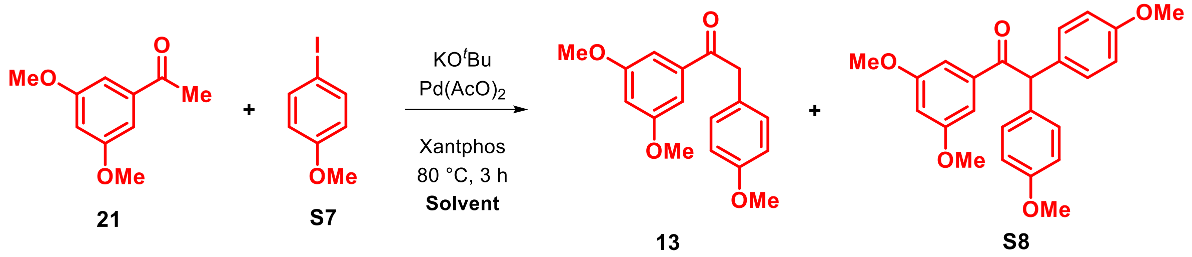

Entry	Stoichiometry 21 / S7	Solvent	Conc. [M]	Yield[a] 13 : S8 (%)
-	1 / 1.1	Toluene	0.16	52 : 0
1	1.1 / 1	Toluene	0.16	50 : 7
2	1.1 / 1	Benzene	0.16	52 : 0
3	1.1 / 1	α,α,α-Trifluorotoluene	0.16	46 : 5
4	1.1 / 1	Fluorobenzene	0.16	61 : 5
5	1.1 / 1	THF	0.16	71 : 0
6	1.1 / 1	MeCN	0.16	-
7	1.1 / 1	THF	0.33	65 : 0
8	1.1 / 1	THF	0.08	68 : 0
9[b]	1.1 / 1	THF	0.16	72 : 0

Reaction conditions: **S7** (320 µmol), **21** (352 µmol), KO^{t}Bu (416 µmol) in 2 mL of anhydrous solvent (0.16 M) at 80 °C for 3 h. [a] Isolated yield. [b] Using 3.93 mmol of **S7** and 4.32 mmol of **21**.

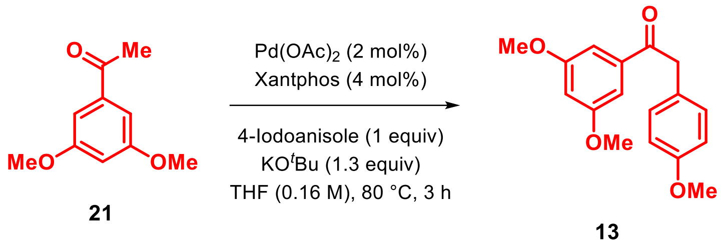

1-(3,5-Dimethoxyphenyl)-2-(4-methoxyphenyl)ethan-1-one (13) was synthesized as follows: To an oven-dried screw-capped tube equipped with a Teflon-coated magnetic stir bar were added the aryl iodide **S9** (0.92 g, 3.93 mmol, 1.0 equiv), acetophenone **21** (0.78 g, 4.32 mmol, 1.1 equiv), Xantphos (0.09 g, 157.2 µmol, 0.04 equiv), $Pd(OAc)_2$ (17.6 mg, 78.6 µmol, 0.02 equiv), and KO*t*Bu (0.57 g, 5.1 mmol, 1.3 equiv), followed by anhydrous THF (24.6 mL, 0.16 M). The reaction tube was degassed using a vacuum pump, charged with nitrogen, and closed. The resulting mixture was heated at 80 °C for 3 h using a hot plate stirrer and an aluminum block. After the reaction time was completed, the excess THF was removed under reduced pressure. The residue was poured into a saturated NH_4Cl solution and extracted with EtOAc (3 × 30 mL). The combined organic layers were dried over anhydrous Na_2SO_4, filtered and concentrated under vacuum. The resulting brown oil was purified by flash column chromatography using silica gel with 20% EtOAc-hexane as eluent to afford 810 mg (72%) of the expected aryl ketone.

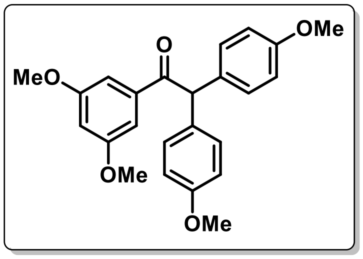

1-(3,5-Dimethoxyphenyl)-2-(4-methoxyphenyl)ethan-1-one (S8). This compound was obtained in several experiments during the optimization of the α-arylation reaction.

Yellow oil, 40 mg (32%, Table S3, entry 2). **^{1}H NMR ($CDCl_3$, 300 MHz): δ** 7.17 (d, 4H, J = 8.9 Hz), 7.14 (d, 2H, J = 2.3 Hz), 6.85 (d, 4H, J = 8.8 Hz), 6.60 (t, 1H, J = 2.3 Hz), 5.87 (s, 1H), 3.74-3.78 (m, 12H). **^{13}C{^{1}H} NMR ($CDCl_3$, 75 MHz): δ** 198.6, 160.9, 158.8, 139.0, 131.7, 130.1, 114.3, 107.0, 105.3, 58.0, 55.6, 55.4. **IR (ATR) (υ_{max}, cm^{-1}):** 2934.25, 2835.91, 1682.87, 1598.90, 1508.04. **HRMS (DART) *m/z*:** $[M + H]^+$ calcd for $C_{24}H_{25}O_5$ 393.1702; found 393.1686.

3. General procedures

General procedure for the synthesis of O-acetyl oximes

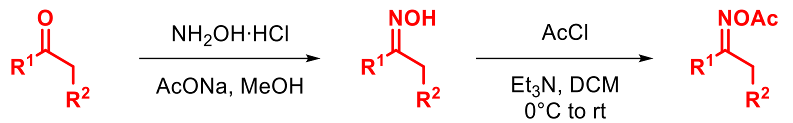

13, 13a, S9a-S9m **S10a-S10o** **11, 11a, 22a-22m**

Ketoxime synthesis: A mixture of ketone (1 equiv), $NH_2OH·HCl$ (1.2 equiv), and AcONa (1.5 equiv) in methanol (3 mL/mmol) was stirred at room temperature until the starting material was completely consumed, as determined by TLC. The reaction mixture was concentrated *in vacuo* until most of the solvent was removed. The reaction mixture was diluted with EtOAc and washed with water, 1 M HCl solution, saturated $NaHCO_3$ solution, and brine. The organic layer was dried over Na_2SO_4, the solvent was removed by evaporation under reduced pressure, and the residue was used directly in the next step.

***O*-Acetyl oxime synthesis:** The crude ketoximes were dissolved in anhydrous DCM (4 mL/mmol) under nitrogen atmosphere. After cooling the solution to 0 °C, acetyl chloride (1.2 equiv) was added, then Et_3N (1.5 equiv) dropwise. The mixture was stirred at room temperature until the completion of the reaction, as determined by TLC. The solvent was concentrated *in vacuo*. The reaction residue was redissolved with EtOAc and washed with water, 1 M HCl solution, saturated $NaHCO_3$ solution, and brine. The organic layer was dried over Na_2SO_4, and the solvent was removed *in vacuo*. The corresponding *O*-acetyl oximes were purified by column chromatography on silica gel.

*Synthesis of the O-acetyl oxime **11***

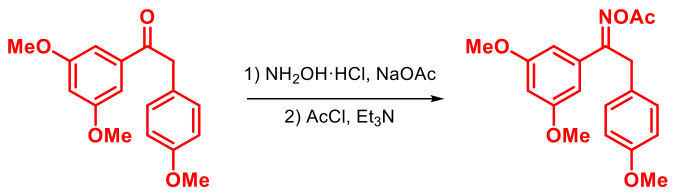

13 **11**

1-(3,5-Dimethoxyphenyl)-2-(4-methoxyphenyl)ethan-1-one *O*-acetyl oxime (11).[9] This compound was synthesized using 680 mg (2.37 mmol) of ketone **13** following the general procedure for the synthesis of *O*-acetyl oximes. The crude product was purified by flash column chromatography using silica gel with 30% EtOAc-hexane as eluent.

Yellow solid, 700 mg (86%), mp 65°C. **^{1}H NMR ($CDCl_3$, 300 MHz): δ** 7.10 (d, 2H, J = 9.0 Hz), 6.85 (d, 2H, J = 2.3 Hz), 6.80 (d, 2H, J = 8.8 Hz), 6.50 (t, 1H, J = 2.3 Hz), 4.12 (s, 2H), 3.77 (s, 6H), 3.76 (s, 3H), 2.23 (s, 3H).

General procedure for the synthesis of benzofuran-4-one compounds

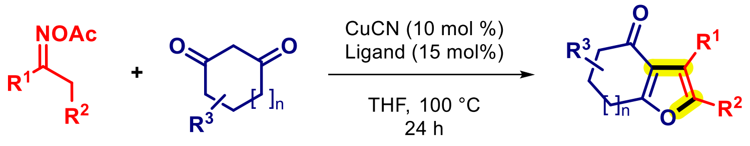

11, 11a, 22a-22m **12,12a, 23a-23i** **10, 10a, 24a-24x, 25**

To an oven-dried screw-capped tube with a Teflon-coated magnetic stir bar were added CuCN (0.015 mmol, 0.1 equiv), 4,4′-di-*tert*-butyl-2,2′-dipyridyl (dtbbpy) (0.022 mmol, 0.15 equiv), and 2 mL of anhydrous THF (0.075 M). The resulting mixture was stirred at room temperature for 30 min. Subsequently, the corresponding *O*-acetyl oxime (0.15 mmol, 1.0 equiv) and 1,3-cyclic diketone (0.16 mmol, 1.1 equiv) were added and the reaction was purged with N_2. The mixture was stirred at 100 °C for 24 h using a hot plate stirrer and an aluminum block. The reaction was filtered through a silica plug and concentrated in vacuum. Afterwards, the reaction residue was redissolved with 3.5 mL of EtOAc and washed twice with 3.5 mL of 1 M HCl solution. The organic layer was dried over Na_2SO_4, concentrated and purified by column chromatography on silica gel.

*Synthesis of the benzofuran-4-one **10** via a copper-mediated heteroannulation*

Table S6. *Study of distinct ligands in the heteroannulation process to access compound **10***

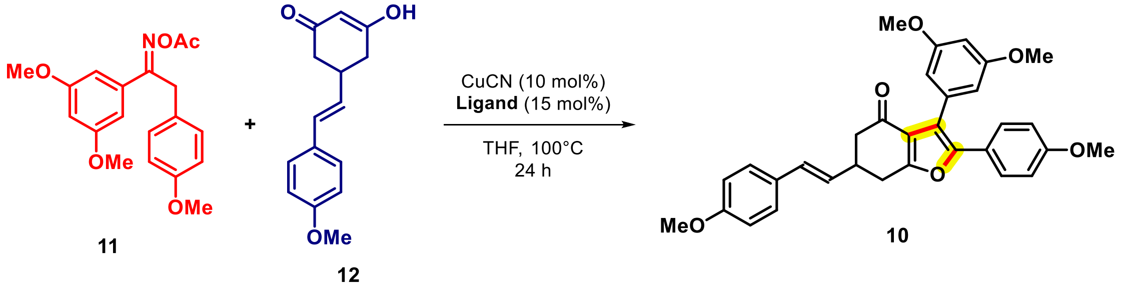

Entry	Ligand	Yield[a] (%)
1	dtbbpy	50
2	(*t*Bu)$_3$-TERPY	66
3[b]	(*t*Bu)$_3$-TERPY	60

11 (150 µmol), **12** (225 µmol), [Cu] 10 mol%, ligand (15 mol%) in 2 mL of anhydrous THF [0.075 M]. [a] Isolated yield. [b] Using 2.33 mmol of **11** and 3.49 mmol of **12**. For the ligand screening studies shown in Table S6, the amount of compound **12** was increased to 1.5 equiv, whereas the general procedure employs 1.1 equiv.

(*E*)-3-(3,5-Dimethoxyphenyl)-2-(4-methoxyphenyl)-6-(4-methoxystyryl)-6,7-dihydrobenzofuran-4(5*H*)-one (10). This compound was synthesized following the general procedure for the synthesis of benzofuran-4-one compounds utilizing 2.33 mmol of oxime acetate **11** and 3.49 mmol of the cyclic 1,3-diketone **12**. The crude product was purified by flash column chromatography using silica gel with 35% EtOAc-hexane as eluent.

White solid, 712 mg (60%), mp 147 °C. **^{1}H NMR ($CDCl_3$, 300 MHz): δ** 7.37 (d, 2H, *J* = 9.0 Hz), 7.32 (d, 2H, *J* = 8.8 Hz), 6.86 (d, 2H, *J* = 8.8 Hz), 6.80 (d, 2H, *J* = 9.0 Hz), 6.57 (d, 2H, *J* = 2.3 Hz), 6.54-6.42 (m, 2H), 6.13 (dd, 1H, *J* = 15.8, 6.7 Hz), 3.81 (s, 3H), 3.79 (s, 3H), 3.75 (s, 6H), 3.19 (dd, 2H, *J* = 12.9, 5.2 Hz), 2.92 (dd, 1H, *J* = 18.3, 11.2 Hz), 2.68 (dd, 1H, *J* = 16.7, 4.4 Hz), 2.54 (dd, 1H, *J* = 16.2, 10.8 Hz). **$^{13}C\{^1H\}$ NMR ($CDCl_3$, 75 MHz): δ** 192.6, 164.8, 160.7, 159.5, 159.4, 149.8, 134.0, 130.1, 129.7, 129.1, 127.9, 127.6, 122.8, 121.3, 118.2, 114.2, 114.0, 108.3, 100.5, 55.5, 55.5, 55.4, 45.2, 38.7, 30.4. **IR (ATR) (υ_{max}, cm^{-1}):** 2905.38, 1675.63, 1590.16, 1508.91, 1245.97, 1152.65. **HRMS (DART) *m/z*:** $[M + H]^+$ calcd for $C_{32}H_{31}O_6$ 511.2120; found 511.2118.

4. Synthesized substrates

The following substrates were used to determine the scope of the heteroannulation:

O-acetyl oximes

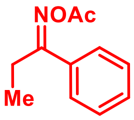

22a

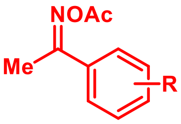

22b, R=H
22c, R=*p*OMe
22d, R=*p*CN
22e, R=*m*Br

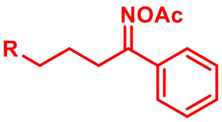

22f, R=CO_2Et
22g, R=Cl

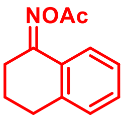

22h

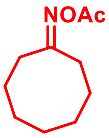

22i

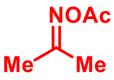

22j

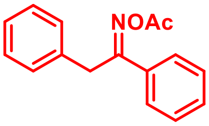

22k

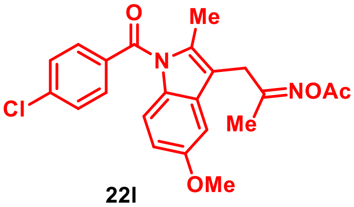

22l

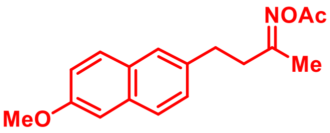

22m

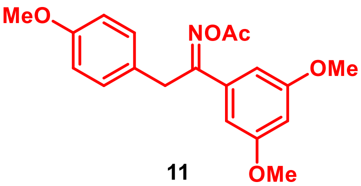

11

Cyclic 1,3-diketones

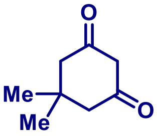

23a

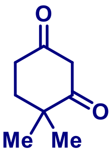

23b

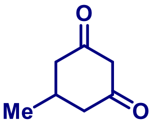

23c

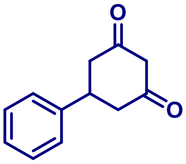

23d

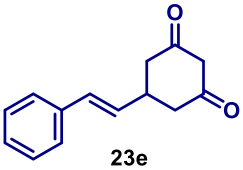

23e

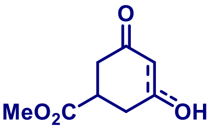

23f

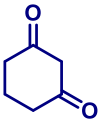

23g

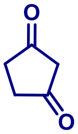

23h

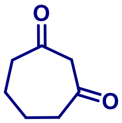

23i

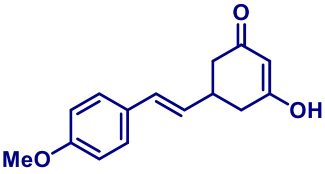

12

*Synthesis of nabumetone O-acetyl oxime **22m***

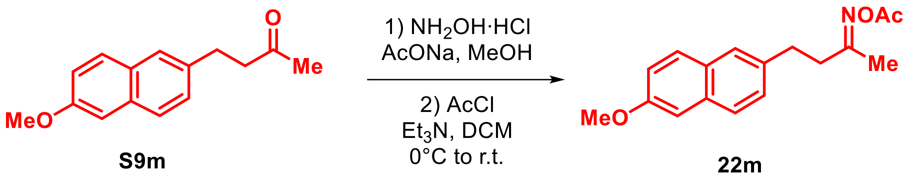

S9m 22m

4-(6-Methoxynaphthalen-2-yl)butan-2-one *O*-acetyl oxime (22m). This compound was synthesized using 120 mg (0.53 mmol) of nabumetone **S9m** following the general procedure for the synthesis of the *O*-acetyl oximes. The crude product was purified by flash column chromatography on silica gel with 10% EtOAc-hexane as eluent.

Colorless oil (*E*/*Z* isomer mixture 3:1), 82 mg (54%). **^{1}H NMR (major isomer) ($CDCl_3$, 300 MHz): δ** 7.68 (d, 2H, *J* = 8.4), 7.61-7.52 (m, 1H), 7.35-7.24 (m, 1H), 7.19-7.07 (m, 2H), 3.91 (s, 3H), 3.06-2.95 (m, 2H), 2.85-2.67 (m, 2H), 2.16 (s, 3H), 2.01 (s, 3H). **$^{13}C\{^1H\}$ NMR (major isomer) ($CDCl_3$, 75 MHz): δ** 169.0, 165.8, 157.5, 135.7, 133.4, 129.1, 127.5, 127.2, 126.5, 126.4, 119.0, 105.8, 55.4, 37.7, 32.7, 19.8, 15.9. **HRMS (DART) *m/z*:** $[M + H]^+$ calcd for $C_{17}H_{20}N_1O_3$ 286.1443; found 286.1433.

*Synthesis of methyl 3-hydroxy-5-oxocyclohex-3-ene-1-carboxylate **23f***

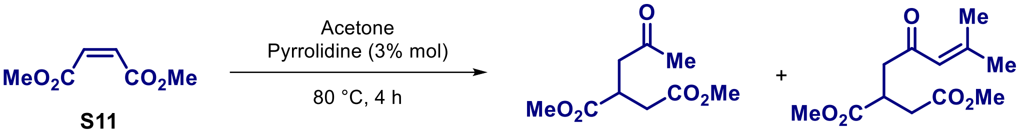

S11 S12 S13

To a screw-capped tube with a Teflon-coated magnetic stir bar were added dimethyl maleate **S11** (2.0 g, 1.7 mL, 13.88 mmol, 1 equiv), acetone (5.27 mL, 70.77 mmol, 5 equiv), and pyrrolidine (34.6 µL, 0.42 mmol, 0.03 equiv). The reaction mixture was heated at 80 °C for 3 h using a hot plate stirrer and an aluminum block. After this time, the residual acetone was removed under reduced pressure. The resulting residue was treated with 1 N HCl (10 mL) and extracted with EtOAc (3 × 15 mL). The combined organic layers were dried over Na_2SO_4, filtered, and concentrated under reduced pressure. The crude product was purified by flash column chromatography on silica gel with 20% EtOAc-hexane as eluent.

Dimethyl 2-(2-oxopropyl)succinate (S12). Colorless oil, 1.25 g (74%). **^{1}H NMR ($CDCl_3$, 300 MHz): δ** 3.65 (s, 3H), 3.64 (s, 3H), 3.23 (p, 1H, *J* = 6.6 Hz), 2.93 (dd, 1H, *J* = 18.0, 6.8 Hz), 2.72-2.50 (m, 3H), 2.13 (s, 3H). **$^{13}C\{^1H\}$ NMR ($CDCl_3$, 75 MHz): δ** 206.0, 174.1, 172.1, 52.3, 51.9, 44.0, 36.3, 35.1, 30.0. **IR (ATR) (υ_{max}, cm^{-1}):** 3001.65, 2955.15, 1730.26, 1715.17. **HRMS (DART) *m/z*:** $[M + H]^+$ calcd for $C_9H_{15}O_5$ 203.0919; found 203.0917.

Dimethyl 2-(2-methyl-4-oxopent-2-en-3-yl)succinate (S13). This compound was obtained as a byproduct in the synthesis of diester **S12**. Colorless oil, 200 mg (20%). **^{1}H NMR ($CDCl_3$, 400 MHz): δ** 6.04 (sept, 1H, *J* = 1.4 Hz), 3.69 (s, 3H), 3.67 (s, 3H), 3.29 (p, 1H, *J* = 6.4 Hz), 2.91 (dd, 1H, *J* = 17.6, 6.3 Hz), 2.77-2.65 (m, 2H), 2.57 (dd, 1H, *J* = 16.6, 6.4 Hz), 2.13 (d, 3H, *J* = 1.3), 1.88 (d, 3H, *J* = 1.5 Hz). **$^{13}C\{^1H\}$ NMR ($CDCl_3$, 100 MHz): δ** 197.6, 174.6, 172.3, 156.7, 123.3, 52.3, 51.9, 44.7, 36.6, 35.3, 27.9, 21.0. **IR (ATR) (υ_{max}, cm^{-1}):** 2953.18, 2916.24, 1732.20. **HRMS (DART) *m/z*:** $[M + H]^+$ calcd for $C_{12}H_{19}O_5$ 243.1232; found 243.1234.

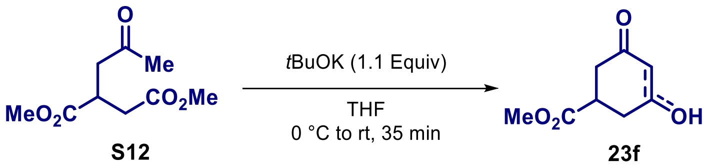

To a round-bottom flask charged with KO*t*Bu (69 mg, 614 µmol, 1.1 equiv) and anhydrous THF (2.5 mL) was added dropwise dimethyl 2-(2-oxopropyl)succinate **S12** (113 mg, 558 µmol, 1 equiv) dissolved in anhydrous THF (2.5 mL) at 0 °C under a nitrogen atmosphere. The reaction mixture was stirred at room temperature for 35 min. After completion, the mixture was quenched with crushed ice–water (5 mL) followed by the addition of 2 N HCl until the pH reached 2. The aqueous phase was extracted with EtOAc (3 × 10 mL), and the combined organic layers were dried over Na_2SO_4 and concentrated under reduced pressure. The crude residue was purified by flash column chromatography on silica gel using a mixture 50% hexane:acetone as eluent.

Methyl 3-hydroxy-5-oxocyclohex-3-ene-1-carboxylate (23f). This compound was isolated predominantly as the enol tautomer, and all spectroscopic data correspond to this form.

White solid, 346 mg (44%), mp < 25 °C. **^{1}H NMR (CDCl$_3$, 300 MHz): δ** 6.96 (s, 1H), 5.51 (s, 1H), 3.72 (s, 3H), 3.10 (p, 1H, *J* = 7.6 Hz), 2.82 (d, 1H, *J* = 6.4 Hz), 2.65 (d, 3H, *J* = 7.5 Hz). **^{13}C{^{1}H} NMR (CDCl$_3$, 75 MHz): δ** 201.8, 189.4, 173.3, 104.5, 52.5, 42.1, 38.5, 34.4. **IR (ATR) (υ_{max}, cm^{-1}):** 2954.51, 2919.36, 1721.19, 1606.31, 1516.23. **HRMS (DART) *m/z*:** $[M + H]^+$ calcd for $C_8H_{11}O_4$ 171.0657; found 171.0662.

5. Spectroscopic characterization of the heteroannulation products

Note: The general synthetic procedure for compounds **24-26** is detailed on pages S9-S10 of this document.

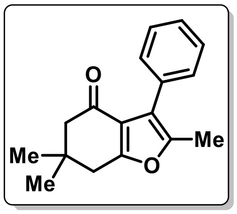

2,6,6-Trimethyl-3-phenyl-6,7-dihydrobenzofuran-4(5*H*)-one (24a).[10] This compound was synthesized using *O*-acetyl oxime **22a** and dimedone **23a** following the general procedure for the synthesis of benzofuran-4-one compounds. The crude reaction mixture was purified by flash column chromatography on silica gel with 3% EtOAc-Hexane as eluent.

Light yellow oil, 31.6 mg (83%). **^{1}H NMR (CDCl$_3$, 300 MHz): δ** 7.41-7.27 (m, 5H), 2.75 (s, 2H), 2.38 (s, 2H), 2.32 (s, 3H), 1.16 (s, 6H). **^{13}C NMR{^{1}H} (CDCl$_3$, 75 MHz): δ** 193.6, 165.0, 149.2, 131.8, 129.9, 128.0, 127.1, 119.2, 118.6, 53.2, 37.8, 35.1, 28.7, 12.2.

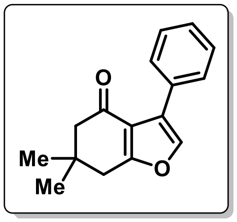

6,6-Dimethyl-3-phenyl-6,7-dihydrobenzofuran-4(5*H*)-one (24b).[10] This compound was synthesized using *O*-acetyl oxime **22b** and dimedone **23a** following the general procedure for the synthesis of benzofuran-4-one compounds. The crude product was purified by flash column chromatography on silica gel with 15% EtOAc-hexane as eluent.

White solid, 25.6 mg (71%). **^{1}H NMR (CDCl$_3$, 300 MHz): δ** 7.64 (d, 2H, *J* = 6.9 Hz), 7.46 (s, 1H), 7.42-7.30 (m, 3H), 2.80 (s, 2H), 2.44 (s, 2H), 1.17 (s, 6H).

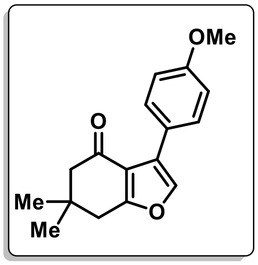

3-(4-Methoxyphenyl)-6,6-dimethyl-6,7-dihydrobenzofuran-4(5*H*)-one (24c). This compound was synthesized using *O*-acetyl oxime **22c** and dimedone **23a** following the general procedure for the synthesis of benzofuran-4-one compounds. The crude product was purified by flash column chromatography on silica gel with 20% EtOAc-hexane as eluent.

Yellow solid, 27.3 mg (67%), mp <30 °C. **^{1}H NMR ($CDCl_3$, 300 MHz): δ** 7.59 (d, 2H, *J* = 8.7 Hz), 7.40 (s, 1H), 6.91 (d, 2H, *J* = 8.7 Hz), 3.82 (s, 3H), 2.78 (s, 2H), 2.42 (s, 2H), 1.16 (s, 6H). **^{13}C{^{1}H} NMR ($CDCl_3$, 75 MHz): δ** 193.9, 167.7, 159.4, 139.1, 129.9, 125.2, 123.2, 117.8, 113.8, 55.4, 53.4, 38.0, 35.0, 28.6. **IR (ATR) (υ_{max}, cm^{-1}):** 2924.47, 1665.59, 1247.30. **HRMS (DART) *m/z*:** $[M + H]^+$ calcd for $C_{17}H_{19}O_3$ 271.1334; found 271.1327.

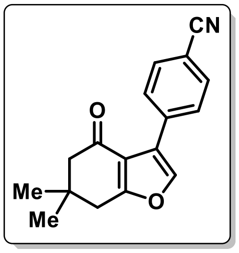

4-(6,6-Dimethyl-4-oxo-4,5,6,7-tetrahydrobenzofuran-3-yl)benzonitrile (24d). This compound was synthesized using *O*-acetyl oxime **22d** and dimedone **23a** following the general procedure for the synthesis of benzofuran-4-one compounds. The crude product was purified by flash column chromatography on silica gel with 10% EtOAc-hexane as eluent.

White solid, 22 mg (55%), mp 147 °C. **^{1}H NMR ($CDCl_3$, 300 MHz): δ** 7.78 (d, 2H, *J* = 8.7 Hz), 7.64 (d, 2H, *J* = 8.7 Hz), 7.54 (s, 1H), 2.81 (s, 2H), 2.45 (s, 2H), 1.18 (s, 6H). **^{13}C{^{1}H} NMR ($CDCl_3$, 75 MHz): δ ppm** 193.8, 168.5, 140.8, 135.7, 132.1, 129.2, 124.3, 119.1, 117.5, 111.2, 53.3, 37.9, 35.1, 28.5. **IR (ATR) (υ_{max}, cm^{-1}):** 3147.38, 2227.58, 1669.94, 1607.44, 1557.56. **HRMS (DART) *m/z*:** $[M + H]^+$ calcd for $C_{17}H_{16}NO_2$ 266.1181; found 266.1178.

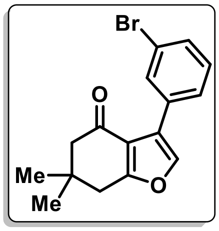

3-(3-Bromophenyl)-6,6-dimethyl-6,7-dihydrobenzofuran-4(5*H*)-one (24e). This compound was synthesized using *O*-acetyl oxime **22e** and dimedone **23a** following the general procedure for the synthesis of benzofuran-4-one compounds. The crude product was purified by flash column chromatography on silica gel with 15% EtOAc-hexane as eluent.

Yellow oil, 28 mg (58%). **^{1}H NMR ($CDCl_3$, 300 MHz): δ** 7.80 (t, 1H, *J* = 1.8 Hz), 7.61 (ddd, 1H, *J* = 7.8, 1.7, 1.0 Hz,), 7.47 (s, 1H), 7.44 (ddd, 1H, *J* = 8.0, 2.0, 1.0 Hz), 7.23 (t, 1H, *J* = 6.0 Hz), 2.80 (s, 2H), 2.44 (s, 2H), 1.17 (s, 6H). **^{13}C{^{1}H} NMR ($CDCl_3$, 75 MHz): δ** 193.8, 168.0, 140.2, 132.9, 131.4, 130.7, 129.9, 127.4, 124.4, 122.3, 117.6, 53.3, 38.0, 35.1, 28.6. **IR (ATR) (υ_{max}, cm^{-1}):** 2921.39, 1678.62, 1465.39, 1437.13. **HRMS (DART) *m/z*:** $[M + H]^+$ calcd for $C_{16}H_{16}BrO_2$ 319.0333; found 319.0340.

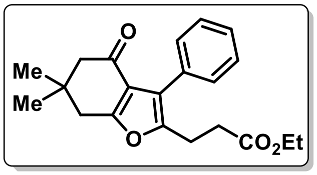

Ethyl 3-(6,6-dimethyl-4-oxo-3-phenyl-4,5,6,7-tetrahydrobenzofuran-2-yl)propanoate (24f). This compound was synthesized using *O*-acetyl oxime **22f** and dimedone **23a** following the general procedure for the synthesis of benzofuran-4-one compounds. The crude reaction mixture was purified by flash column chromatography on silica gel with 2% EtOAc-hexane as eluent.

Yellow oil, 40.4 mg (79%). **^{1}H NMR ($CDCl_3$, 300 MHz): δ** 7.38-7.27 (m, 5H), 4.11 (q, 2H, *J* = 7.2 Hz), 3.00 (dd, 2H, *J* = 9.0, 6.4 Hz), 2.74 (s, 2H), 2.64 (dd, 2H, *J* = 8.7, 6.8 Hz), 2.37 (s, 2H), 1.22 (t, 3H, *J* = 7.1 Hz), 1.16 (s, 6H). **^{13}C{^{1}H} NMR ($CDCl_3$, 75 MHz): δ** 193.6, 172.4, 165.3, 150.8, 131.3, 129.9, 128.1,

127.4, 119.9, 118.6, 60.7, 53.1, 37.8, 35.0, 32.9, 28.7, 21.8, 14.3. **IR (ATR) (υ_{max}, cm^{-1}):** 2958.04, 2925.80, 1675.95, 1181.13. **HRMS (DART) *m/z*:** $[M + H]^+$ calcd for $C_{21}H_{25}O_4$ 341.1752; found 341.1740.

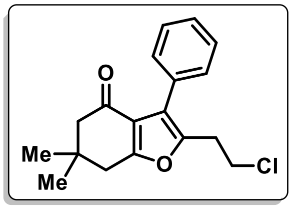

2-(2-Chloroethyl)-6,6-dimethyl-3-phenyl-6,7-dihydrobenzofuran-4(5*H*)-one (24g). This compound was synthesized using *O*-acetyl oxime **22g** and dimedone **23a** following the general procedure for the synthesis of benzofuran-4-one compounds. The crude product was purified by flash column chromatography on silica gel with 5% EtOAc-hexane as eluent.

Yellow oil, 20.6 mg (45%). **^{1}H NMR ($CDCl_3$, 300 MHz): δ** 7.40-7.29 (m, 5H), 3.75 (t, 2H, J = 7.2 Hz), 3.11 (t, 2H, J = 7.2 Hz), 2.77 (s, 2H), 2.38 (s, 2H), 1.17 (s, 6H). **$^{13}C\{^1H\}$ NMR ($CDCl_3$, 75 MHz): δ** 193.5, 165.6, 148.6, 131.0, 129.9, 128.2, 127.6, 121.5, 118.7, 53.1, 41.8, 37.8, 35.1, 29.8, 28.7. **IR (ATR) (υ_{max}, cm^{-1}):** 2926.74, 1668.01, 1448.38. **HRMS (DART) *m/z*:** $[M + H]^+$ calcd for $C_{18}H_{20}ClO_2$ 303.1151; found 303.1150.

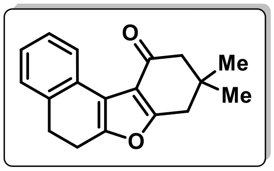

9,9-Dimethyl-6,8,9,10-tetrahydronaphtho[2,1-*b*]benzofuran-11(5*H*)-one (24h). This compound was synthesized using *O*-acetyl oxime **22h** and dimedone **23a** following the general procedure for the synthesis of benzofuran-4-one compounds. The crude product was purified by flash column chromatography on silica gel with 50% CH_2Cl_2-hexane as eluent.

Yellow oil, 15.9 mg (40%). **^{1}H NMR ($CDCl_3$, 300 MHz): δ** 8.47 (dd, 1H, J = 7.5, 1.2 Hz), 7.30-7.24 (m, 1H), 7.18-7.10 (m, 2H), 3.07 (t, 2H, J = 8.1 Hz), 2.87 (t, 2H, J = 8.4 Hz), 2.77 (s, 2H), 2.46 (s, 2H), 1.17 (s, 6H). **$^{13}C\{^1H\}$ NMR ($CDCl_3$, 75 MHz): δ** 194.0, 166.3, 153.7, 133.4, 130.3, 127.7, 127.3, 126.8, 126.6, 118.2, 116.9, 53.3, 38.0, 35.0, 29.8, 28.6, 22.0. **IR (ATR) (υ_{max}, cm^{-1}):** 2953.55, 2928.98, 1665.11, 1563.68. **HRMS (DART) *m/z*:** $[M + H]^+$ calcd for $C_{18}H_{19}O_2$ 267.1385; found 267.1378.

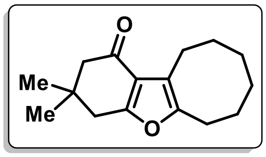

3,3-Dimethyl-3,4,6,7,8,9,10,11-octahydrocycloocta[*b*]benzofuran-1(2*H*)-one (24i).[11] This compound was synthesized using *O*-acetyl oxime **22i** and dimedone **23a** following the general procedure for the synthesis of benzofuran-4-one compounds. The crude product was purified by flash column chromatography on silica gel with 2% EtOAc-hexane as eluent.

Yellow oil, 22.9 mg (62%). **^{1}H NMR ($CDCl_3$, 300 MHz): δ** 2.83-2.79 (m, 2H), 2.75-2.71 (m, 2H), 2.66 (s, 2H), 2.31 (s, 2H), 1.74-1.66 (m, 4H), 1.52-1.38 (m, 4H), 1.11 (s, 6H). **$^{13}C\{^1H\}$ NMR ($CDCl_3$, 75 MHz): δ** 195.4, 164.1, 152.4, 119.4, 116.9, 52.9, 37.7, 35.3, 28.8, 28.0, 27.7, 25.9, 25.8, 25.8, 21.8. **IR (ATR) (υ_{max}, cm^{-1}):** 2926.68, 1660.57, 1047.58. **HRMS (DART) *m/z*:** $[M + H]^+$ calcd for $C_{16}H_{23}O_2$ 247.1698; found 247.1697.

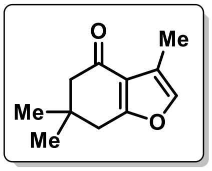

3,6,6-Trimethyl-6,7-dihydrobenzofuran-4(5*H*)-one (24j).[12] This compound was synthesized using *O*-acetyl oxime **22j** and dimedone **23a** following the general procedure for the synthesis of benzofuran-4-one compounds. The crude product was purified by flash column chromatography on silica gel with 10% EtOAc-hexane as eluent.

Colorless oil, 19 mg (71%). **^{1}H NMR ($CDCl_3$, 300 MHz): δ ppm** 7.06 (s, 1H), 2.69 (s, 2H), 2.34 (s, 2H), 2.18 (s, 3H), 1.12 (s, 6H).

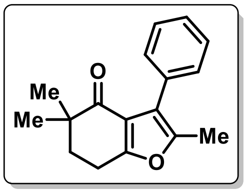

2,5,5-Trimethyl-3-phenyl-6,7-dihydrobenzofuran-4(5*H*)-one (24k). This compound was synthesized using *O*-acetyl oxime **22a** and 1,3-cyclohexanedione **23b** following the general procedure for the synthesis of benzofuran-4-one compounds. The crude product was purified by flash column chromatography on silica gel with 20% EtOAc-hexane as eluent.

Yellow oil, 14.4 mg (38%). **^{1}H NMR ($CDCl_3$, 400 MHz): δ** 7.39-7.35 (m, 4H), 7.32-7.27 (m, 1H), 2.90 (t, 2H, *J* = 6.3 Hz), 2.30 (s, 3H), 2.01 (t, 2H, *J* = 6.4 Hz), 1.17 (s, 6H). **^{13}C{^{1}H} NMR ($CDCl_3$, 100 MHz): δ** 199.2, 164.0, 149.2, 132.0, 130.0, 127.9, 127.1, 119.9, 118.0, 42.5, 36.4, 24.4, 21.2, 12.2. **IR (ATR) (υ_{max}, cm^{-1}):** 2924.17, 1671.83, 1316.28. **HRMS (DART) *m/z*:** $[M + H]^+$ calcd for $C_{17}H_{19}O_2$ 255.1385; found 255.1382.

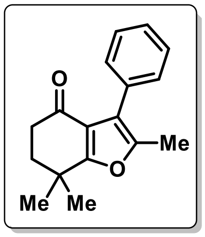

2,7,7-Trimethyl-3-phenyl-6,7-dihydrobenzofuran-4(5*H*)-one (24k'). This compound was synthesized using *O*-acetyl oxime **22a** and 1,3-cyclohexanedione **23b** following the general procedure for the synthesis of benzofuran-4-one compounds. The crude product was purified by flash column chromatography on silica gel with 20% EtOAc-hexane as eluent.

White solid, 13.5 mg (35%), mp 96-98 °C. **^{1}H NMR ($CDCl_3$, 400 MHz): δ** 7.40-7.35 (m, 4H), 7.32-7.27 (m, 1H), 2.55 (dd, 2H, *J* = 7.1, 6.0 Hz), 2.30 (s, 3H), 2.00 (dd, 2H, *J* = 7.2, 5.9 Hz), 1.41 (s, 6H). **^{13}C{^{1}H} NMR ($CDCl_3$, 100 MHz): δ** 194.1, 171.7, 148.8, 131.9, 130.0, 127.9, 127.1, 119.2, 117.5, 37.8, 36.4, 32.9, 26.3, 12.1. **IR (ATR) (υ_{max}, cm^{-1}):** 2959.65, 1681.93, 1359.91. **HRMS (DART) *m/z*:** $[M + H]^+$ calcd for $C_{17}H_{19}O_2$ 255.1385; found 255.1385.

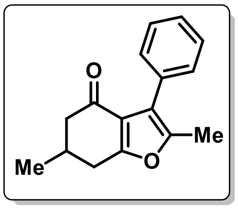

2,6-Dimethyl-3-phenyl-6,7-dihydrobenzofuran-4(5*H*)-one (24l). This compound was synthesized using *O*-acetyl oxime **22a** and 1,3-cyclohexanedione **23c** following the general procedure for the synthesis of benzofuran-4-one compounds. The crude reaction mixture was purified by flash column chromatography on silica gel with 2% EtOAc-hexane as eluent.

Yellow oil, 35.2 mg (97%). **^{1}H NMR ($CDCl_3$, 300 MHz): δ** 7.39-7.28 (m, 5H), 2.96 (dd, 1H, *J* = 16.3, 4.2 Hz), 2.60-2.41 (m, 3H), 2.31 (s, 3H), 2.31-2.21 (m, 1H), 1.18 (d, 3H, *J* = 6.2 Hz). **^{13}C{^{1}H} NMR ($CDCl_3$, 75 MHz): δ** 193.8, 165.6, 149.0, 131.8, 129.9, 128.0, 127.1, 119.5, 119.3, 47.3, 31.9, 30.7, 21.2, 12.1. **IR (ATR) (υ_{max}, cm^{-1}):** 2922.85, 1672.94, 1576.19. **HRMS (DART) *m/z*:** $[M + H]^+$ calcd for $C_{16}H_{17}O_2$ 241.1228; found 241.1232.

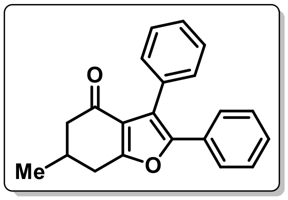

6-Methyl-2,3-diphenyl-6,7-dihydrobenzofuran-4(5*H*)-one (24m). This compound was synthesized using *O*-acetyl oxime **22k** and 1,3-cyclohexanedione **23c** following the general procedure for the synthesis of benzofuran-4-one compounds. The crude product was purified by flash column chromatography on silica gel with 10% EtOAc-hexane as eluent.

White solid, 25.8 mg (57%), mp 124 °C. **^{1}H NMR ($CDCl_3$, 400 MHz): δ** 7.44-7.37 (m, 7H), 7.30-7.24 (m, 3H), 3.11 (ddd, 1H, *J* = 17.0, 4.9, 1.1 Hz), 2.69 (dd, 1H, *J* = 17.1, 10.1 Hz), 2.61-2.49 (m, 2H), 2.38-2.27 (m, 1H), 1.24 (d, 3H, *J* = 6.5 Hz). **^{13}C{^{1}H} NMR ($CDCl_3$, 100 MHz): δ** 193.7, 166.0, 149.3, 132.0, 130.2, 128.5, 128.4, 128.0, 127.8, 126.3, 121.1, 119.9, 47.3, 32.0, 30.7, 21.2. **IR (ATR) (υ_{max}, cm^{-1}):** 2920.77, 2873.60, 1768.73, 1678.05. **HRMS (EI, 70eV) *m/z*:** $[M]^+$ calcd for $C_{21}H_{18}O_2$ 302.1307; found 302.1313.

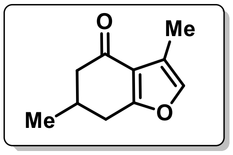

3,6-Dimethyl-6,7-dihydrobenzofuran-4(5*H*)-one (evodone, 24n).[13] This compound was synthesized using *O*-acetyl oxime **22j**, 1,3-cyclohexanedione **23c** (1.5 equiv) and 4,4',4''-tri-*tert*-butyl-2,2':6',2''-terpyridine as ligand following the general procedure for the synthesis of benzofuran-4-one compounds. The crude product was purified by flash column chromatography on silica gel with 20% EtOAc-hexane as eluent.

Colorless oil, 6 mg (24%). **^{1}H NMR ($CDCl_3$, 300 MHz): δ** 7.06 (d, 1H, *J* = 1.3 Hz), 2.98-2.81 (m, 1H), 2.57-2.31 (m, 3H), 2.27-2.20 (m, 1H), 2.19 (d, 3H, *J* = 1.4 Hz), 1.15 (d, 3H, *J* = 6.4 Hz). **^{13}C{^{1}H} NMR ($CDCl_3$, 75 MHz): δ** 195.4, 167.3, 139.3, 120.2, 119.2, 47.0, 31.9, 31.0, 21.2, 9.2.

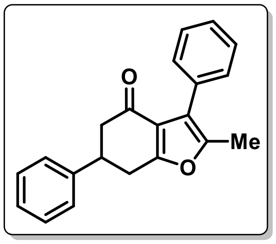

2-Methyl-3,6-diphenyl-6,7-dihydrobenzofuran-4(5*H*)-one (24o). This compound was synthesized using *O*-acetyl oxime **22a** and 1,3-cyclohexanedione **23d** following the general procedure for the synthesis of benzofuran-4-one compounds. The crude product was purified by flash column chromatography on silica gel with 3% EtOAc-hexane as eluent.

Light yellow solid, 33.2 mg (73%), mp 139-140 °C. **^{1}H NMR ($CDCl_3$, 400 MHz): δ** 7.44-7.27 (m, 10H), 3.60 (tt, 1H, *J* = 11.1, 5.1 Hz), 3.20 (dd, 1H, *J* = 17.0, 5.1 Hz), 3.08 (dd, 1H, *J* = 17.0, 11.2 Hz), 2.85-2.72 (m, 2H), 2.35 (s, 3H). **^{13}C{^{1}H} NMR ($CDCl_3$, 100 MHz): δ** 192.7, 165.1, 149.4, 142.7, 131.7, 129.9, 129.0, 128.0, 127.3, 127.3, 126.9, 119.7, 119.4, 46.0, 41.2, 31.6, 12.2. **IR (ATR) (υ_{max}, cm^{-1}):** 3053.39, 1668.98, 1048.12. **HRMS (DART) *m/z*:** $[M + H]^+$ calcd for $C_{21}H_{19}O_2$ 303.1385; found 303.1395.

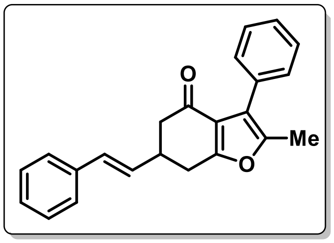

(*E*)-2-Methyl-3-phenyl-6-styryl-6,7-dihydrobenzofuran-4(5*H*)-one (24p). This compound was synthesized using *O*-acetyl oxime **22a** and cyclic 1,3-cyclohexanedione **23e** following the general procedure for the synthesis of benzofuran-4-one compounds. The crude product was purified by flash column chromatography on silica gel with 5% EtOAc-hexane as eluent.

Light yellow solid, 39.3 mg (80%), mp 140-141 °C. **^{1}H NMR ($CDCl_3$, 300 MHz): δ** 7.42-7.22 (m, 10H), 6.53 (d, 1H, *J* = 15.9 Hz), 6.27 (dd, 1H, *J* = 15.9, 6.9 Hz), 3.27-3.09 (m, 2H), 2.87 (dd, 2H, *J* = 16.6, 9.6 Hz), 2.54 (dd, 1H, *J* = 16.3, 10.8 Hz), 2.34 (s,

3H). **$^{13}C\{^{1}H\}$ NMR ($CDCl_3$, 75 MHz): δ** 192.7, 164.8, 149.3, 136.9, 131.7, 131.3, 130.6, 129.9, 128.7, 128.0, 127.8, 127.2, 126.4, 119.7, 119.3, 44.9, 38.7, 30.1, 12.2. **IR (ATR) (υ_{max}, cm^{-1}):** 2958.14, 1670.41, 1042.96. **HRMS (DART) *m/z*:** $[M + H]^+$ calcd for $C_{23}H_{21}O_2$ 329.1541; found 329.1526.

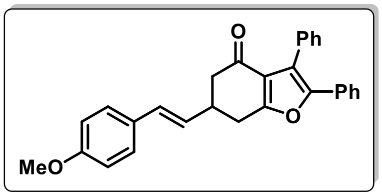

(*E*)-6-(4-Methoxystyryl)-2,3-diphenyl-6,7-dihydrobenzofuran-4(5*H*)-one (24q). This compound was synthesized using *O*-acetyl oxime **22k** and cyclic1,3-cyclohexanedione **12** following the general procedure for the synthesis of benzofuran-4-one compounds. The crude product was purified by flash column chromatography on silica gel with 30% EtOAc-hexane as eluent.

Yellow oil, 34.8 mg (55%). **^{1}H NMR ($CDCl_3$, 300 MHz): δ** 7.42-7.27 (m, 10H), 7.26-7.22 (m, 2H), 6.87 (d, 2H, *J* = 8.7 Hz), 6.48 (d, 1H, *J* = 15.6 Hz), 6.14 (dd, 1H, *J* = 15.9, 6.9 Hz), 3.83 (s, 3H), 3.27-3.19 (m, 2H), 2.95 (dd, 1H, *J* = 18.0, 11.1 Hz), 2.69 (dd, 1H, *J* = 16.5, 4.2 Hz), 2.55 (dd, 1H, *J* = 16.2, 10.8 Hz). **$^{13}C\{^{1}H\}$ NMR ($CDCl_3$, 75 MHz): δ** 192.9, 165.4, 159.4, 149.6, 131.9, 130.2, 130.1, 130.1, 129.6, 129.0, 128.5, 128.4, 128.1, 127.9, 127.6, 126.3, 121.3, 119.9, 114.2, 55.5, 45.1, 38.7, 30.4. **IR (ATR) (υ_{max}, cm^{-1}):** 2959.25, 1679.77, 1510.89,1247.95. **HRMS (DART) *m/z*:** $[M + H]^+$ calcd for $C_{29}H_{25}O_3$ 421.1803; found 421.1794.

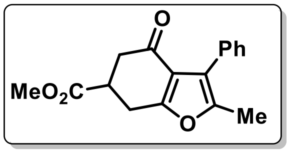

Methyl 2-methyl-4-oxo-3-phenyl-4,5,6,7-tetrahydrobenzofuran-6-carboxylate (24r). This compound was synthesized using *O*-acetyl oxime **22a** and 1,3-cyclohexanedione **23f** following the general procedure for the synthesis of benzofuran-4-one compounds. The crude product was purified by flash column chromatography on silica gel with 10% EtOAc-hexane as eluent.

Yellow oil, 12.6 mg (29%). **^{1}H NMR ($CDCl_3$, 400 MHz): δ** 7.41-7.35 (m, 4H), 7.33-7.28 (m, 1H), 3.75 (s, 3H), 3.36-3.24 (m, 1H), 3.18 (dd, 2H, *J* = 7.3, 2.6 Hz), 2.84-2.67 (m, 2H), 2.32 (s, 3H). **$^{13}C\{^{1}H\}$ NMR ($CDCl_3$, 100 MHz): δ** 190.9, 173.2, 163.6, 149.8, 131.5, 129.9, 128.1, 127.4, 119.8, 119.4, 52.6, 40.7, 40.0, 26.3, 12.2. **IR (ATR) (υ_{max}, cm^{-1}):** 2922.08, 2851.73, 1732.92, 1678.58, 1194.66. **HRMS (DART) *m/z*:** $[M + H]^+$ calcd for $C_{17}H_{17}O_4$: 285.1126; found 285.1125.

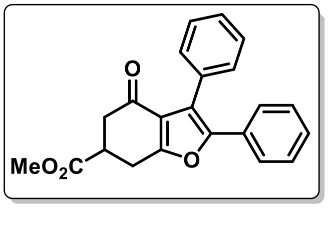

Methyl 4-oxo-2,3-diphenyl-4,5,6,7-tetrahydrobenzofuran-6-carboxylate (24s). This compound was synthesized using *O*-acetyl oxime **22k** and 1,3-cyclohexanedione **23f** following the general procedure for the synthesis of benzofuran-4-one compounds. The crude product was purified by flash column chromatography on silica gel with 10% EtOAc-hexane as eluent.

Yellow oil, 24.4 mg (47%). **^{1}H NMR ($CDCl_3$, 400 MHz): δ** 7.43-7.30 (m, 6H), 7.30-7.18 (m, 4H), 3.77 (s, 3H), 3.40-3.24 (m, 3H), 2.86-2.69 (m, 2H). **$^{13}C\{^{1}H\}$ NMR ($CDCl_3$, 100 MHz): δ** 190.8, 173.1, 164.0, 150.0, 131.6, 130.2, 129.9, 128.5, 128.4, 128.2, 128.0, 126.3, 121.3, 119.8, 52.6, 40.8, 39.9, 26.4. **IR (ATR) (υ_{max}, cm^{-1}):** 2922.88, 2850.74, 1727.19, 1680.87, 1617.13. **HRMS (EI, 70eV) *m/z*:** $[M]^+$ calcd for $C_{22}H_{18}O_4$ 346.1205; found 346.1210.

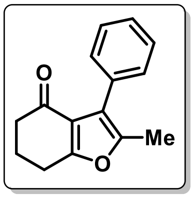

2-Methyl-3-phenyl-6,7-dihydrobenzofuran-4(5*H*)-one (24t).[11] This compound was synthesized using *O*-acetyl oxime **22a** and 1,3-cyclohexanedione **23g** following the general procedure for the synthesis of benzofuran-4-one compounds. The crude product was purified by flash column chromatography on silica gel with 5% EtOAc-hexane as eluent.

Yellow oil, 17.9 mg (53%). **^{1}H NMR ($CDCl_3$, 300 MHz): δ** 7.38-7.27 (m, 5H), 2.89 (t, 2H, *J* = 6.3 Hz), 2.49 (dd, 2H, *J* = 7.4, 5.6 Hz), 2.31 (s, 3H), 2.18 (p, 2H, *J* = 6.5 Hz). **^{13}C{^{1}H} NMR ($CDCl_3$, 75 MHz): δ** 194.2, 165.9, 148.9, 131.9, 129.9, 128.0, 127.2, 119.9, 119.3, 38.8, 23.8, 22.7, 12.1.

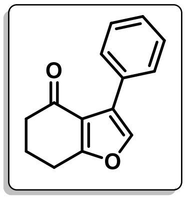

3-Phenyl-6,7-dihydrobenzofuran-4(5*H*)-one (24u).[10] This compound was synthesized using *O*-acetyl oxime **22b**, 1,3-cyclohexanedione **23g** and 4,4',4''-tri-*tert*-butyl-2,2':6',2''-terpyridine as ligand following the general procedure for the synthesis of benzofuran-4-one compounds. The crude product was purified by flash column chromatography on silica gel with 15% EtOAc-hexane as eluent.

White solid, 21 mg (66%). **^{1}H NMR ($CDCl_3$, 300 MHz): δ** 7.62 (dd, 2H, *J* = 8.2, 1.5 Hz), 7.48-7.23 (m, 4H), 2.93 (t, 2H, *J* = 6.3 Hz), 2.55 (t, 2H, *J* = 6.3 Hz), 2.21 (td, 2H, *J* = 6.2, 1.4 Hz).

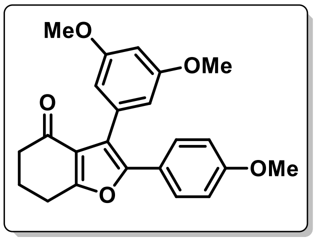

3-(3,5-Dimethoxyphenyl)-2-(4-methoxyphenyl)-6,7-dihydrobenzofuran-4(5*H*)-one (24v). This compound was synthesized using *O*-acetyl oxime **11**, 1,3-cyclohexanedione **23g** (1.5 equiv) and 4,4',4''-tri-*tert*-butyl-2,2':6',2''-terpyridine as ligand following the general procedure for the synthesis of benzofuran-4-one compounds. The crude product was purified by flash column chromatography on silica gel with 25% EtOAc-hexane as eluent.

White solid, 44.2 mg (78%), mp 158 °C. **^{1}H NMR ($CDCl_3$, 500 MHz): δ** 7.35 (d, 2H, *J* = 9.0 Hz), 6.79 (d, 2H, *J* = 8.9 Hz), 6.55 (d, 2H, *J* = 2.4 Hz), 6.45 (t, 1H, *J* = 2.3 Hz), 3.78 (s, 3H), 3.74 (s, 6H), 2.97 (t, 2H, *J* = 6.3 Hz), 2.50 (dd, 2H, *J* = 7.3, 5.7 Hz), 2.21 (p, 2H, *J* = 6.4 Hz). **^{13}C{^{1}H} NMR ($CDCl_3$, 126 MHz): δ** 194.0, 165.8, 160.6, 159.4, 149.4, 134.1, 127.6, 122.8, 121.3, 118.2, 114.0, 108.2, 100.4, 55.5, 55.4, 38.8, 23.9, 22.5. **IR (ATR) (υ_{max}, cm^{-1}):** 2931.74, 2938.82, 2835.90, 1673.03, 1580.82, 1247.54. **HRMS (DART) *m/z*:** $[M + H]^+$ calcd for $C_{23}H_{23}O_5$ 379.1545; found 379.1543.

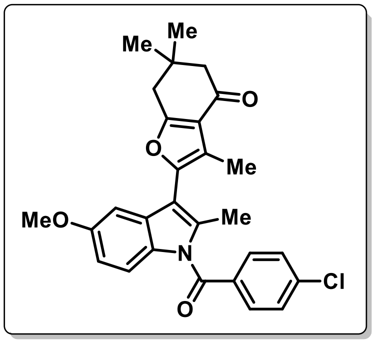

2-(1-Benzoyl-5-methoxy-2-methyl-1H-indol-3-yl)-3,6,6-trimethyl-6,7-dihydrobenzofuran-4(5*H*)-one (24w). This compound was synthesized using *O*-acetyl oxime **22l** and dimedone **23a** following the general procedure for the synthesis of benzofuran-4-one compounds. The crude product was purified by flash column chromatography on silica gel with 30% EtOAc-hexane as eluent.

Yellow oil, 37 mg (52%). **^{1}H NMR ($CDCl_3$, 300 MHz): δ** 7.71 (d, 2H, *J* = 8.5 Hz), 7.50 (d, 2H, *J* = 8.5 Hz), 6.96 (d, 1H, *J* = 9.1 Hz), 6.91 (d, 1H, *J* = 2.5 Hz), 6.72 (dd, 1H, *J* = 9.0, 2.6 Hz), 3.81 (s, 3H), 2.81 (s, 2H), 2.43 (s, 2H), 2.34 (s, 3H), 2.22 (s, 3H), 1.20 (s, 6H). **^{13}C{^{1}H} NMR ($CDCl_3$, 75 MHz): δ** 195.1, 168.4, 166.5, 156.4, 144.2, 139.8, 137.3, 133.7, 131.4, 131.1, 129.9, 129.4,

120.5, 117.3, 115.2, 112.3, 111.0, 102.7, 55.9, 52.8, 37.9, 35.3, 28.9, 15.0, 10.5. **IR (ATR) (υ_{max}, cm^{-1}):** 3088.07, 2956.93, 1670.91, 1311.98, 1089.02, 756.21. **HRMS (DART) *m/z*:** $[M + H]^+$ calcd for $C_{28}H_{27}NO_4Cl$ 476.1628; found 476.1619.

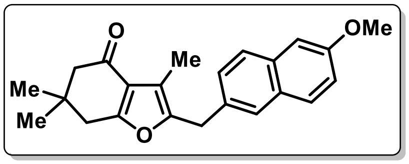

2-((6-Methoxynaphthalen-2-yl)methyl)-3,6,6-trimethyl-6,7-dihydrobenzofuran-4(5*H*)-one (24x). This compound was synthesized using *O*-acetyl oxime **22m** and dimedone **23a** following the general procedure for the synthesis of benzofuran-4-one compounds. The crude product was purified by flash column chromatography on silica gel with 20% EtOAc-hexane as eluent, followed by preparative plate chromatography using 10% THF-toluene as eluent.

White solid, 14.6 mg (28%). **^{1}H NMR ($CDCl_3$, 300 MHz): δ** 7.67 (d, 2H, *J* = 8.4 Hz), 7.54 (s, 1H), 7.28 (dd, 1H, *J* = 8.4, 1.9 Hz), 7.18-7.07 (m, 2H), 4.01 (s, 2H), 3.91 (s, 3H), 2.63 (s, 2H), 2.32 (s, 2H), 2.24 (s, 3H), 1.11 (s, 6H). **$^{13}C\{^1H\}$ NMR ($CDCl_3$, 75 MHz):** δ 195.3, 165.2, 157.6, 150.2, 133.5, 133.4, 129.2, 127.5, 127.3, 126.7, 120.0, 119.1, 113.8, 105.8, 55.4, 52.8, 37.7, 35.2, 31.9, 28.8, 9.2. **IR (ATR) (υ_{max}, cm^{-1}):** 2952.44, 2917.5, 1675.87, 1604.39. **HRMS (DART) *m/z*:** $[M + H]^+$ calcd for $C_{23}H_{25}O_3$ 349.1803; found 349.1792.

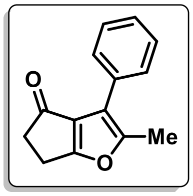

2-Methyl-3-phenyl-5,6-dihydro-4*H*-cyclopenta[*b*]furan-4-one (25). This compound was synthesized using *O*-acetyl oxime **22a** and 1,3-cyclopentanedione **23h** following the general procedure for the synthesis of benzofuran-4-one compounds. The crude product was purified by flash column chromatography on silica gel using a gradient of 10% to 25% EtOAc-hexane.

White solid, 17 mg (53%), mp 81-83 °C. **^{1}H NMR ($CDCl_3$, 300 MHz): δ** 7.65-7.63 (m, 2H), 7.44-7.39 (m, 2H), 7.31-7.26 (m, 1H), 3.03-2.96 (m, 4H), 2.53 (s, 3H). **$^{13}C\{^1H\}$ NMR ($CDCl_3$, 75 MHz): δ** 195.1, 181.4, 155.3, 131.3, 128.7, 128.6, 127.6, 127.3, 118.5, 42.0, 22.1, 14.1. **IR (ATR) (υ_{max}, cm^{-1}):** 2917.68, 1688.72, 1305.64. **HRMS (DART) *m/z*:** $[M + H]^+$ calcd for $C_{14}H_{13}O_2$ 213.0915; found 213.0908.

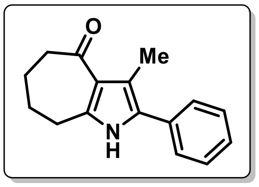

3-Methyl-2-phenyl-5,6,7,8-tetrahydrocyclohepta[*b*]pyrrol-4(1*H*)-one (26). This compound was synthesized using *O*-acetyl oxime **22a** and 1,3-cycloheptanedione **23i** following the general procedure for the synthesis of benzofuran-4-one compounds. The crude product was purified by flash column chromatography on silica gel with 7% EtOAc-hexane as eluent.

Light yellow solid, 9.9 mg (27%), mp 159-161 °C. **^{1}H NMR ($CDCl_3$, 400 MHz): δ** 8.12 (s, 1H), 7.43-7.37 (m, 4H), 7.30-7.27 (m, 1H), 2.94 (dd, 2H, *J* = 6.9, 5.1 Hz), 2.72-2.69 (m, 2H), 2.41 (s, 3H), 1.98-1.88 (m, 4H). **$^{13}C\{^1H\}$ NMR ($CDCl_3$, 100 MHz): δ** 199.2, 140.0, 132.6, 128.9, 128.0, 127.4, 126.9, 122.8, 118.4, 43.4, 27.6, 25.0, 22.4, 11.9. **IR (ATR) (υ_{max}, cm^{-1}):** 3165.91, 2919.90, 1605.80, 1470.25. **HRMS (DART) *m/z*:** $[M + H]^+$ calcd for $C_{16}H_{18}NO$ 240.1388; found 240.1395.

*Synthesis of the benzofuran **27** via an oxidative aromatization*

Table S7.** Optimization of the oxidative aromatization of benzofuran-4-one **10

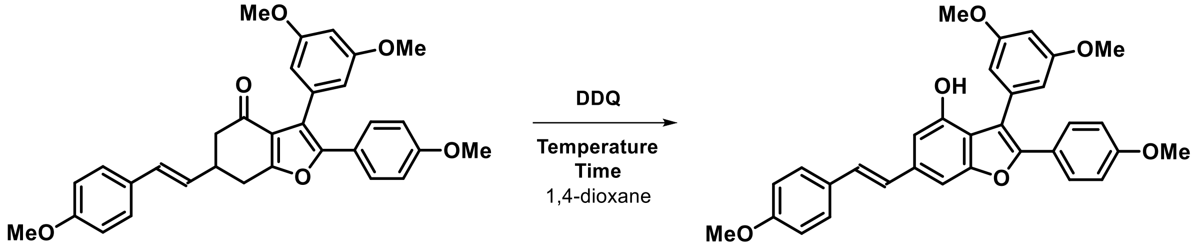

Entry	DDQ (Equiv)	Temperature (°C)	Time (h)	Yield[a] 27 (%)
1	1.4	100	18	C.M.
2	1.4	60	18	C.M.
3	1.4	rt	2	C.M.
4	1	rt	2	55
5	0.5	rt	2	40

Reaction conditions: **10** (39 µmol) in 2 mL of anhydrous 1,4-dioxane. [a] Isolated yield. C.M.: Complex mixture; rt: room temperature (≈ 25 °C).

To a solution of the benzofuran-4-one **10** (20 mg, 39 µmol, 1 equiv) in dry 1,4-dioxane (2 mL, 0.02 M) was added DDQ (8.8 mg, 39 µmol, 1 equiv) under a nitrogen atmosphere. The mixture was stirred at room temperature for a period of 2 h. Subsequently, the reaction was quenched by the addition of a saturated $NaHCO_3$ solution (10 mL) and extracted with EtOAc (3 × 10 mL). The combined organic layers were dried over Na_2SO_4 and concentrated under reduced pressure. The crude product was purified by flash column chromatography using silica gel with 40% EtOAc-hexane as eluent.

(*E*)-3-(3,5-Dimethoxyphenyl)-2-(4-methoxyphenyl)-6-(4-methoxystyryl)benzofuran-4-ol (27). Yellow oil, 11 mg (55%). **^{1}H NMR ($CDCl_3$, 300 MHz): δ** 7.54 (d, 2H, *J* = 9.0 Hz), 7.47 (d, 2H, *J* = 8.9 Hz), 7.24 (s, 1H), 7.09 (d, 1H, *J* = 16.2 Hz), 7.01 (d, 1H, *J* = 16.3 Hz), 6.91 (d, 2H, *J* = 8.9 Hz), 6.87 (s, 1H), 6.84 (d, 2H, *J* = 9.0 Hz), 6.69 (d, 2H, *J* = 2.3 Hz), 6.57 (t, 1H, *J* = 2.3 Hz), 5.24 (br s, 1H), 3.84 (s, 3H), 3.81 (s, 9H). **^{13}C {^{1}H} NMR ($CDCl_3$, 75 MHz): δ** 162.1, 159.8, 159.5, 155.3, 150.1, 149.9, 136.0, 135.4, 130.3, 128.2, 128.0, 127.9, 126.9, 123.1, 117.4, 114.3, 114.1, 114.1, 107.6, 107.1, 102.0, 101.1, 55.7, 55.5, 55.4. **IR (ATR) (υ_{max}, cm^{-1}):** 2929.99, 1679.17, 1597.56, 1508.34, 1246.42, 1151.10, 1028.01. **HRMS (DART) *m/z*:** $[M + H]^+$ calcd for $C_{32}H_{29}O_6$ 509.1964; found 509.1942.

Synthesis of anigopreissin A **6** *via a demethylation process*

Table S8. *Study of the demethylation conditions using benzofuran* **27**

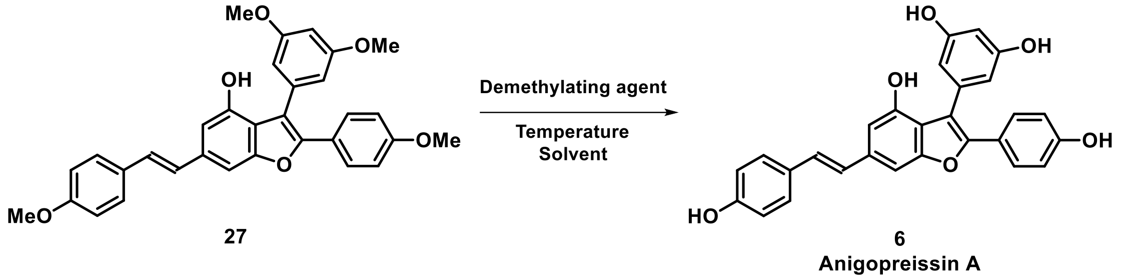

Entry	Demethylating agent / equiv	Temperature (°C)	Solvent	Yield[a] 6 (%)
1	BCl_3 / 15	0	DCM	C.M.
2	BCl_3 / 15	-78	DCM	C.M.
3	BCl_3 / 25	0	DCM	15
4	$AlCl_3$ / 10	100	Toluene	N.R.
5[b]	LiCl / 10	120	DMF	N.R.

Reaction conditions: **27** (39 µmol), TBAI (585 µmol) for 6 h. [a] Isolated yield. [b] PTSA (195 µmol). C.M.: Complex mixture; N.R.= No reaction.

Entry 3, procedure: To a solution of compound **27** (20 mg, 39 µmol, 1 equiv) and TBAI (142 mg, 585 µmol, 15 equiv) in anhydrous DCM (5 mL, 7.8 mM), BCl_3 (980 µL, 975 µmol, 25 equiv) was added dropwise at 0 °C under a nitrogen atmosphere. The reaction mixture was stirred for 6 h while maintaining the same temperature. The reaction was quenched by the addition of H_2O (5 mL), followed by the dropwise addition of a saturated $NaHCO_3$ solution (5 mL). The resulting mixture was extracted with EtOAc (3 × 15 mL). The combined organic layers were dried over Na_2SO_4, filtered, and the solvent was removed under reduced pressure. The crude product was purified by flash column chromatography on silica gel using 10% acetone-CH_2Cl_2 as the eluent

(*E*)-5-(4-Hydroxy-2-(4-hydroxyphenyl)-6-(4-hydroxystyryl)benzofuran-3-yl)benzene-1,3-diol (anigopreissin A, 6). Yellow oil, 2.6 mg (15%). **^{1}H NMR (acetone-*d*$_6$, 400 MHz): δ** 8.69 (br s, 1H), 8.50 (br s, 1H), 8.36 (br s, 2H), 7.89 (br s, 1H), 7.47 (d, 2H, J = 8.4 Hz), 7.46 (d, 2H, J = 8.7 Hz), 7.26 (d, 1H, J = 1.3 Hz), 7.14 (d, 1H, J = 16.4 Hz), 7.06 (d, 1H, J = 16.3 Hz), 6.88 (d, 1H, J = 1.3 Hz), 6.86 (d, 2H, J = 8.7 Hz), 6.81 (d, 2H, J = 9.0 Hz), 6.51 (d, 2H, J = 2.2 Hz), 6.42 (t, 1H, J = 2.3 Hz). **^{13}C{^{1}H} NMR (acetone-*d*$_6$, 100 MHz): δ** 159.6, 158.5, 158.1, 156.6, 152.4, 150.7, 136.5, 130.1, 129.0, 128.8, 128.7, 126.9, 123.1, 118.7, 116.5, 116.3, 116.2, 109.8, 107.5, 103.0, 101.5. **IR (ATR) (υ_{max}, cm^{-1}):** 3380.46, 2976.77, 1692.99, 1366.04. **HRMS (DART) *m/z*:** $[M + H]^+$ calcd for $C_{28}H_{21}O_6$ 453.1338; found 453.1325.

Table S9. *Comparative ¹H/¹³C NMR data of the isolated natural product, reported sample and this work*

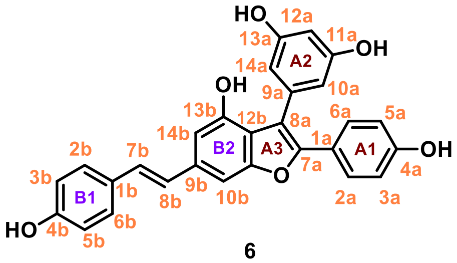

6

Anigopreissin A

Position	Isolated natural product ^{1}H NMR Acetone-d_6, 500 MHz[14]	Reported synthetic sample ^{1}H NMR CD_3OD, 400 MHz[15]	Synthesized in this work ^{1}H NMR Acetone-d_6, 400 MHz	Isolated natural product ^{13}C{^{1}H} NMR Acetone-d_6, 500 MHz[14]	Reported synthetic sample ^{13}C{^{1}H} NMR CD_3OD, 400 MHz[15]	Synthesized in this work ^{13}C{^{1}H} NMR Acetone-d_6, 400 MHz
**1a**	-	-	-	123.0	123.7	123.1
**2a/6a**	7.46 (d, 2H, *J* = 8.8 Hz)	7.44-7.35 (m, 4H)	7.47 (d, 2H, *J* = 8.4 Hz)	128.6	128.8	128.7
**3a/5a**	6.80 (d, 2H, *J* = 8.8 Hz)	6.71 (d, 2H, *J* = 8.8 Hz)	6.81 (d, 2H, *J* = 9.0 Hz)	116.1	116.2	116.2
**4a**	8.73 (br s, 1H, OH)	-	8.69 (s, 1H, OH)	158.4	158.7	158.5
**7a**	-	-	-	150.6	151.2	150.7
**8a**	-	-	-	116.3	116.6	116.3
**9a**	-	-	-	136.5	137.2	136.5
**10a/14a**	6.49 (d, 2H, *J* = 2.2 Hz)	6.42 (d, 2H, *J* = 2.2 Hz)	6.51 (d, 2H, *J* = 2.2 Hz)	109.7	110.3	109.8
**11a/13a**	8.38 (br s, 2H, OH)	-	8.36 (s, 2H, OH)	159.5	159.5	159.6
**12a**	6.41 (t, 1H, *J* = 2.2 Hz)	6.31 (t, 1H, *J* = 2.2 Hz)	6.42 (t, 1H, *J* = 2.2 Hz)	102.9	102.8	103.0
**1b**	-	-	-	130.0	130.7	130.1
**2b/6b**	7.45 (d, 2H, *J* = 8.5 Hz)	-	7.46 (d, 2H, *J* = 8.7 Hz)	128.9	129.3	129.0
**3b/5b**	6.85 (d, 2H, *J* = 8.5 Hz)	6.78 (d, 2H, *J* = 8.6 Hz)	6.86 (d, 2H, *J* = 8.7 Hz)	116.4	116.8	116.5
**4b**	8.54 (s, 1H, OH)	-	8.50 (s, 1H, OH)	158.1	158.4	158.1
**7b**	7.13 (d, 1H, *J* = 16.3 Hz)	7.06 (d, 1H, *J* = 16.2 Hz)	7.14 (d, 1H, *J* = 16.4 Hz)	128.7	128.9	128.8
**8b**	7.06 (d, 1H, *J* = 16.3 Hz)	6.98 (d, 1H, *J* = 16.2 Hz)	7.06 (d, 1H, *J* = 16.3 Hz)	126.8	127.3	126.9
**9b**	-	-	-	136.4	136.8	136.5
**10b**	7.25 (d, 1H, *J* = 0.8 Hz)	7.15 (d, 1H, *J* = 0.9 Hz)	7.26 (d, 1H, *J* = 1.3 Hz)	101.4	101.7	101.5
**11b**	-	-	-	156.5	157.2	156.6
**12b**	-	-	-	118.6	119.1	118.7

**13b**	7.96 (s, 1H, OH)	-	7.89 (s, 1H, OH)	152.4	153.0	152.4
**14b**	6.87 (d, 1H, *J* = 0.8 Hz)	6.76 (d, 1H, *J* = 0.9 Hz)	6.88 (d, 1H, *J* = 1.3 Hz)	107.4	107.2	107.5

6. Total synthesis of anigopresissin A and fuliginosin A: Second approach

*Synthesis of cyclic 1,3-diketone **12a***

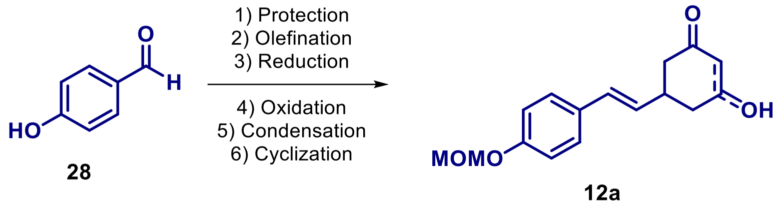

The synthesis of **12a** was carried out using 2.5 g (20.47 mmol, 1 equiv) of 4-hydroxybenzaldehyde **28** through an initial protection step, followed by the synthetic sequence olefination/reduction/oxidation/condensation applied to aldehyde **18** (see pages S2-S3). No purification was performed between steps due to the high purity of the intermediates. The identity of each intermediate was confirmed by ^{1}H NMR analysis of the crude reaction mixtures.

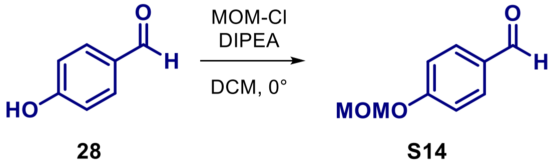

To a round-bottom flask equipped with a magnetic stir bar, 4-hydroxybenzaldehyde **28** (2.50 g, 20.47 mmol, 1 equiv) was dissolved in anhydrous DCM (20 mL, 1.02 M). DIPEA (5.35 mL, 30.71 mmol, 1.5 equiv) was added under a nitrogen atmosphere and cooled to 0 °C. The reaction was stirred for 10 min, and MOM-Cl (3.11 mL, 40.94 mmol, 2 equiv) was added dropwise while maintaining the temperature at 0 °C. After stirring for 5 h, the reaction mixture was quenched with a saturated NH_4Cl solution and extracted with DCM (3 × 30 mL). The combined organic layers were dried over Na_2SO_4, filtered, and concentrated under reduced pressure. The crude product, which showed high purity by ^{1}H NMR, was used directly in the next step without further purification.

4-(Methoxymethoxy)benzaldehyde (S14).[16] **^{1}H NMR ($CDCl_3$, 300 MHz): δ** 9.90 (s, 1H), 7.83 (d, 2H, *J* = 8.9 Hz), 7.14 (d, 2H, *J* = 8.7 Hz), 5.25 (s, 2H), 3.49 (s, 3H).

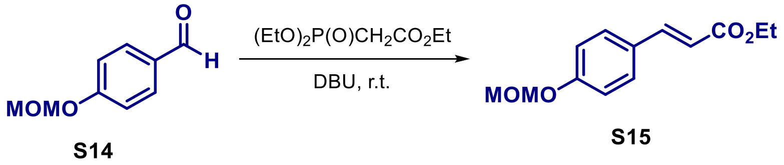

Ethyl (*E*)-3-(4-(methoxymethoxy)phenyl)acrylate (S15). Brown oil. **^{1}H NMR ($CDCl_3$, 300 MHz): δ** 7.64 (d, 1H, *J* = 16.1 Hz), 7.46 (d, 2H, *J* = 9.0 Hz), 7.03 (d, 2H, *J* = 8.9 Hz), 6.32 (d, 1H, *J* = 16.0 Hz), 5.20 (s, 2H), 4.25 (q, 2H, *J* = 7.2 Hz), 3.47 (s, 3H), 1.33 (t, 3H, *J* = 7.2 Hz).

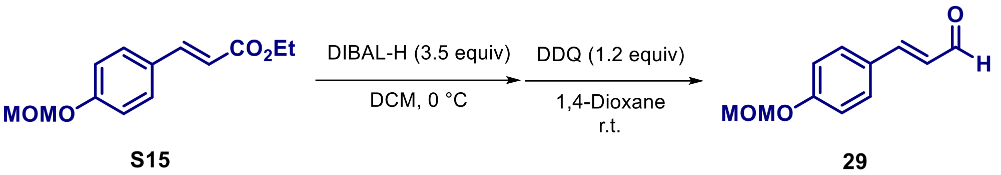

(*E*)-3-(4-(Methoxymethoxy)phenyl)acrylaldehyde (29).[17] Yellow oil, 2.81 g (71%). Purified by flash column chromatography on silica gel using 20% EtOAc/hexane as eluent. **^{1}H NMR ($CDCl_3$, 300 MHz): δ** 9.66 (d, 1H, *J* = 7.7 Hz), 7.52 (d, 2H, *J* = 8.5 Hz), 7.43 (d, 1H, *J* = 15.8 Hz), 7.08 (d, 2H, *J* = 8.8 Hz), 6.62 (dd, 1H, *J* = 15.9, 7.8 Hz), 5.22 (s, 2H), 3.48 (s, 3H).

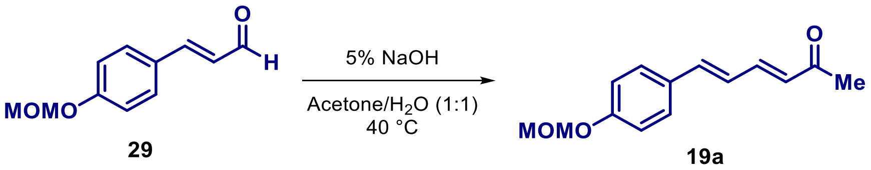

(3*E*,5*E*)-6-(4-(Methoxymethoxy)phenyl)hexa-3,5-dien-2-one (19a).[17] Yellow solid, 2.88 g (85%). Purified by flash column chromatography on silica gel using 20% EtOAc/hexane as eluent. **^{1}H NMR ($CDCl_3$, 300 MHz): δ** 7.42 (d, 2H, *J* = 8.6 Hz), 7.28 (dd, 1H, *J* = 15.5, 10.5 Hz), 7.03 (d, 2H, *J* = 8.9 Hz), 6.91 (d, 1H, *J* = 15.7 Hz), 6.85-6.70 (m, 1H), 6.22 (d, 1H, *J* = 15.4 Hz), 5.20 (s, 2H), 3.48 (s, 3H), 2.31 (s, 3H).

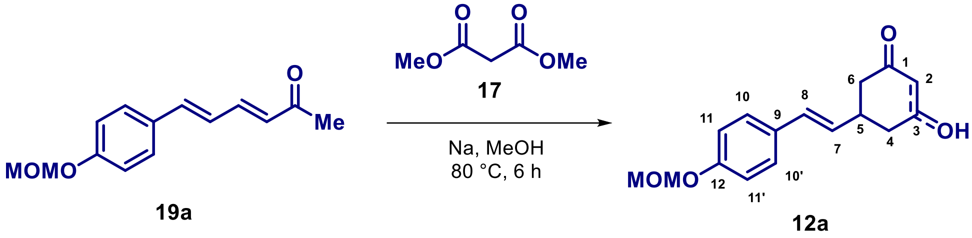

In a round-bottom flask equipped with a magnetic stir bar, 4 mL of MeOH (0.22 M) was added and cooled to 0 °C. Sodium (0.14 g, 6.03 mmol, 7 equiv) was gradually introduced in portions, and the mixture was stirred at this temperature. Once the metal was consumed, dimethyl malonate (0.12 mL, 1.03 mmol, 1.2 equiv) was added, and the reaction mixture was stirred for another 10 min. Afterwards, 0.2 g (0.86 mmol, 1 equiv) of **19a** was added, and the solution was heated at 80 °C for 6 h using a hot plate stirrer and an aluminum block. The reaction mixture was poured into ice-cold H_2O (10 mL) and acidified to pH 5 with 1 N HCl. The resulting red solution was extracted with EtOAc (3 x 15 mL) and the organic phases were combined, dried over Na_2SO_4, filtered, and evaporated under reduced pressure. The crude product was purified by flash column chromatography on silica gel using a mixture of 5% acetone-CH_2Cl_2 as eluent.

(*E*)-5-(4-(Methoxymethoxy)styryl)cyclohexane-1,3-dione (12a). This compound was isolated as a tautomeric keto-enol mixture (2:1). Dark brown oil, 190 mg (80%).

^{1}H NMR (keto-enol mixture) ($CDCl_3$, 300 MHz): δ 7.26 (d, 3.55H, *J* = 8.6 Hz), 6.98 (d, 3.51H, *J* = 8.8 Hz), 6.41 (d, 1.61H, *J* = 15.6 Hz), 6.10-5.88 (m, 1.66H), 5.54 (s, 0.52H, **enol-2**), 5.16 (s, 3.55H), 3.47 (s, 4.63H), 3.41 (s, 2H, **keto-2**), 3.03-2.87 (m, 1.69H), 2.83 (dd, 1.78H, *J* = 15.5, 4.4 Hz), 2.70-2.50 (m, 3.59H), 2.37 (dd, 1.60H, *J* = 17.1, 10.7 Hz). **$^{13}C\{^1H\}$ NMR ($CDCl_3$, 75 MHz): δ** 203.0 (keto, **C1**), 190.0 (enol, **C3**), 157.3 (keto, **C12**), 157.1 (enol, **C12**), 131.0 (enol, **C9**), 130.7 (keto, **C8**), 130.5 (keto, **C9**), 129.9 (enol,

C8), 129.6 (enol, **C7**), 128.0 (keto, **C7**), 127.7 (keto, **C10**, **C10'**), 127.6 (enol, **C10**, **C10'**), 116.7 (keto and enol, **C11**, **C11'**), 104.9 (enol, **C2**), 94.7 (keto and enol, **CH_2**-MOM), 57.9 (keto, **C2**), 56.1 (keto and enol, **CH_3**-MOM), 46.3 (keto, **C6**, **C4**), 38.7 (enol, **C6**, **C4**), 37.1 (enol, **C5**), 33.8 (keto, **C5**). **IR (ATR) (υ_{max}, cm^{-1}):** 2922.94, 2894.58, 1723.45, 1598.23, 1507.04, 1227.51, 966.66. **HRMS (DART) *m/z*:** $[M + H]^+$ calcd for $C_{16}H_{19}O_4$ 275.1283; found 275.1279.

*Synthesis of the α-arylacetophenone **13a** via a α-arylation*

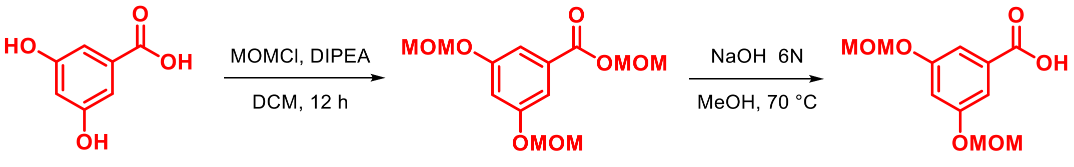

30 S16 31

***O*-Alkylation:** To a solution of 3,5-dihydroxybenzoic acid **30** (1.5 g, 9.75 mmol, 1 equiv) in anhydrous DCM (10 mL, 0.98 M), under a nitrogen atmosphere at 0 °C, was added DIPEA (12 mL, 68.2 mmol, 7 equiv) followed by MOM-Cl (5.18 mL, 68.2 mmol, 7 equiv). The reaction was stirred at room temperature for 12 h, quenched by the addition of a saturated NH_4Cl solution (15 mL), and extracted with DCM (3 × 35 mL). The combined organic layers were combined, dried over anhydrous Na_2SO_4, filtered, and concentrated under reduced pressure to afford the crude ester intermediate.

Ester hydrolysis: The ester was dissolved in MeOH (8 mL, 1.22 M), and a 6 N NaOH solution (10 mL, 0.98 M) was added. The mixture was heated at 70 °C for 3 h using a hot plate stirrer and an aluminum block, then cooled in an ice bath, and the pH was adjusted to 5 with 6 N HCl. The resulting precipitate was collected by vacuum filtration to give 1.9 g (7.84 mmol) of compound **31**. The MeOH was removed from the mother liquors under reduced pressure, and the remaining aqueous layer was extracted with EtOAc (3 × 25 mL). The combined organic extracts were combined, dried over anhydrous Na_2SO_4, filtered, and concentrated under reduced pressure. The crude residue was purified by flash column chromatography using silica gel with 30% EtOAc-hexane as eluent to afford an additional 100 mg (0.41 mmol) of benzoic acid **31**.

3,5-Bis(methoxymethoxy)benzoic acid (31).[18] White solid, 2.0 g (84%), mp 125 °C. **^{1}H NMR ($CDCl_3$, 300 MHz): δ** 7.44 (d, 2H, J = 2.3 Hz), 6.97 (t, 1H, J = 2.3 Hz), 5.21 (s, 4H), 3.49 (s, 6H).

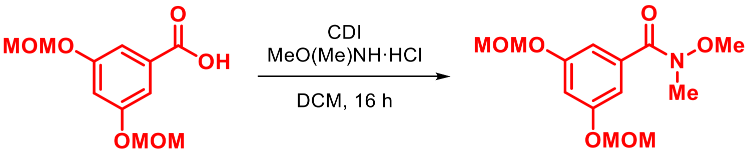

31 S17

To a solution of 3,5-bis(methoxymethoxy)benzoic acid **31** (0.8 g, 3.3 mmol, 1 equiv) in anhydrous DCM (15 mL, 0.22 M) was added *N,N'*-carbonyldiimidazole (0.59 g, 3.63 mmol, 1.1 equiv) portionwise under nitrogen atmosphere. The reaction mixture was stirred for 1 h at room temperature. Afterwards, *N,O*-dimethylhydroxylamine hydrochloride (0.39 g, 3.96 mmol, 1.2 equiv) was added, and the mixture was stirred overnight at room temperature. The reaction was quenched with a saturated $NaHCO_3$ solution and extracted with EtOAc (3 × 15 mL). The combined organic layers were dried over Na_2SO_4, filtered, and

concentrated under reduced pressure. The crude product was purified by flash column chromatography on silica gel with 35% EtOAc-hexane as eluent.

***N*-Methoxy-3,5-bis(methoxymethoxy)-*N*-methylbenzamide (S17).** Colorless oil, 850 mg (90%). **^{1}H NMR (CD_3OD, 300 MHz): δ** 6.97 (d, 2H, *J* = 2.3 Hz), 6.88 (t, 1H, *J* = 2.3 Hz), 5.23 (s, 4H), 3.67 (s, 3H), 3.49 (s, 6H), 3.36 (s, 3H). **^{13}C{^{1}H} NMR (CD_3OD, 75 MHz): δ** 171.1, 159.5, 137.2, 110.2, 108.1, 95.7, 61.7, 56.3, 34.5. **IR (ATR) (υ_{max}, cm^{-1}):** 2934.24, 2826.99, 1645.17, 1589.53, 1419.23, 1140.26. **HRMS (DART) *m/z*:** $[M + H]^+$ calcd for $C_{13}H_{20}NO_6$ 286.1290; found 286.1284.

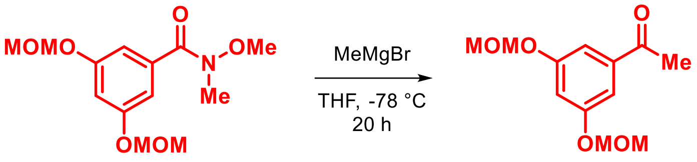

S17 **21a**

A solution of Weinreb amide **S17** (0.7 g, 2.45 mmol, 1 equiv) in anhydrous THF (10 mL, 0.25 M) was cooled to −78 °C. Afterwards, 2.86 mL of MeMgBr (8.59 mmol, 3.5 equiv) was added dropwise under a nitrogen atmosphere. The mixture was stirred for 6 h at −78 °C, and then overnight at room temperature. The reaction was quenched with a saturated NH_54Cl solution and extracted with EtOAc (3 × 15 mL). The combined organic layers were dried over Na_2SO_4, filtered, and concentrated under reduced pressure. The crude product was purified by flash column chromatography using silica gel with 20% EtOAc-hexane as eluent.

1-(3,5-Bis(methoxymethoxy)phenyl)ethan-1-one (21a).[19] Colorless oil, 555 mg (94%). **^{1}H NMR ($CDCl_3$, 300 MHz): δ** 7.27 (d, 2H, *J* = 2.3 Hz), 6.94 (t, 1H, *J* = 2.3 Hz), 5.20 (s, 4H), 3.49 (s, 6H), 2.57 (s, 3H). **^{13}C{^{1}H} NMR ($CDCl_3$, 75 MHz): δ** 197.6, 158.5, 139.3, 109.6, 94.6, 56.3, 26.9.

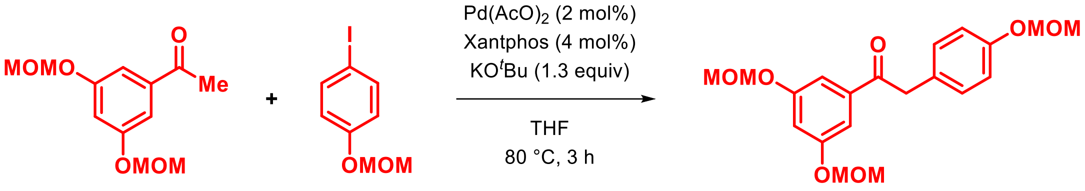

21a **S18** **13a**

Following the procedure employed to synthesize the aryl ketone **13** (see page S8), 1-(3,5-bis(methoxymethoxy)phenyl)ethan-1-one **21a** (0.95 g, 3.95 mmol, 1.1 equiv), 4-(methoxymethoxy)iodobenzene **S18** (0.95 g, 3.59 mmol, 1 equiv), KO*t*Bu (0.52 g, 4.67 mmol, 1.3 equiv), $Pd(AcO)_2$ (16 mg, 71.8 µmol, 0.02 equiv), and Xantphos (83 mg, 143 µmol, 0.04 equiv) were reacted at 80 °C for 3 h in anhydrous THF (22.4 mL, 0.16 M). The crude product was purified by flash column chromatography on silica gel with 30% EtOAc-hexane as eluent.

1-(3,5-Bis(methoxymethoxy)phenyl)-2-(4-(methoxymethoxy)phenyl)ethan-1-one (13a). Yellow oil, 1.05 g (78%). **^{1}H NMR ($CDCl_3$, 300 MHz): δ** 7.33 (d, 2H, *J* = 2.2 Hz), 7.18 (d, 2H, *J* = 8.8 Hz), 6.99 (d, 2H, *J* = 8.8 Hz), 6.92 (t, 1H, *J* = 2.3 Hz), 5.17 (s, 4H), 5.14 (s, 2H), 4.17 (s, 2H), 3.47 (s, 6H), 3.46 (s, 3H). **^{13}C{^{1}H} NMR ($CDCl_3$, 75 MHz): δ** 197.2, 158.5, 156.3, 138.7, 130.6, 127.8, 116.6, 110.0, 109.7, 94.7,

94.6, 56.3, 56.0, 45.0. **IR (ATR) (υ_{max}, cm^{-1}):** 2954.28, 2901.53, 1719.67, 1680.50, 1591.96, 1509.97. **HRMS (DART) *m/z*:** $[M + H]^+$ calcd for $C_{20}H_{25}O_7$ 377.1600; found 377.1598.

*Synthesis of the O-acetyl oxime **11a***

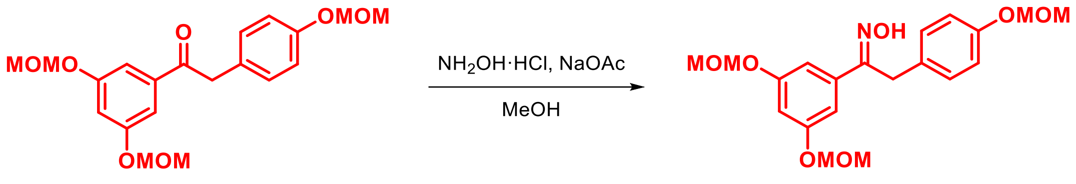

13a **S10o**

1-(3,5-Bis(methoxymethoxy)phenyl)-2-(4-(methoxymethoxy)phenyl)ethan-1-one oxime (S10o). This compound was synthesized following the ketoxime synthesis method using 0.98 g (2.6 mmol) of **13a**. The crude oxime was purified by flash column chromatography on silica gel with 30% EtOAc-hexane as eluent.

Colorless oil, 770 mg (75%). **^{1}H NMR ($CDCl_3$, 300 MHz): δ** 7.20 (d, 2H, *J* = 8.8 Hz), 7.00 (d, 2H, *J* = 2.3 Hz), 6.94 (d, 2H, *J* = 8.7 Hz), 6.74 (t, 1H, *J* = 2.2 Hz), 5.13 (s, 6H), 4.11 (s, 2H), 3.45 (s, 9H). **$^{13}C\{^1H\}$ NMR ($CDCl_3$, 75 MHz): δ** 158.4, 157.2, 155.8, 137.7, 130.0, 129.8, 116.5, 108.3, 105.8, 94.6, 94.6, 56.2, 56.0, 31.3. **IR (ATR) (υ_{max}, cm^{-1}):** 3365.15, 2930.25, 2900.79, 1586.54, 1508.38. **HRMS (DART) *m/z*:** $[M + H]^+$ calcd for $C_{20}H_{26}NO_7$ 392.1709; found 392.1694.

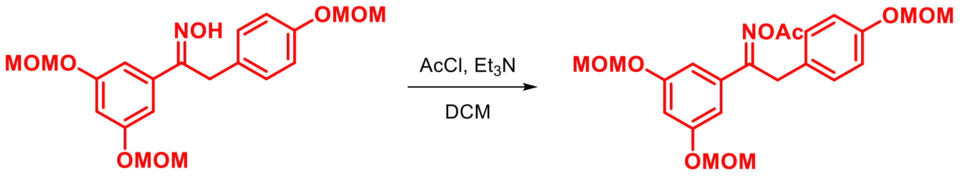

S10o **11a**

1-(3,5-Bis(methoxymethoxy)phenyl)-2-(4-(methoxymethoxy)phenyl)ethan-1-one *O*-acetyl oxime (11a). This compound was synthesized following the *O*-acetyl oxime synthesis method using 0.77 g (1.97 mmol) of **S10o**. The crude acetate was purified by flash column chromatography on silica gel with 40% EtOAc-hexane as eluent.

White solid, 840 mg (98%), mp 50 °C. **^{1}H NMR ($CDCl_3$, 300 MHz): δ** 7.10 (d, 2H, *J* = 8.8 Hz), 7.03 (d, 2H, *J* = 2.3 Hz), 6.92 (d, 2H, *J* = 8.7 Hz), 6.78 (t, 1H, *J* = 2.2 Hz), 5.12 (s, 6H), 4.11 (s, 2H), 3.43 (s, 9H), 2.23 (s, 3H). **$^{13}C\{^1H\}$ NMR ($CDCl_3$, 75 MHz): δ** 168.9, 163.7, 158.4, 156.1, 136.0, 129.7, 128.5, 116.6, 109.3, 106.8, 94.6, 94.5, 56.2, 56.0, 33.6, 19.9. **IR (ATR) (υ_{max}, cm^{-1}):** 2953.66, 2901.60, 1766.44, 1583.12, 1509.26. **HRMS (EI, 70eV) *m/z*:** $[M]^+$ calcd for $C_{22}H_{27}NO_8$ 433.1737; found 433.1752.

*Synthesis of the benzofuran-4-one **10a** via a copper-mediated heteroannulation*

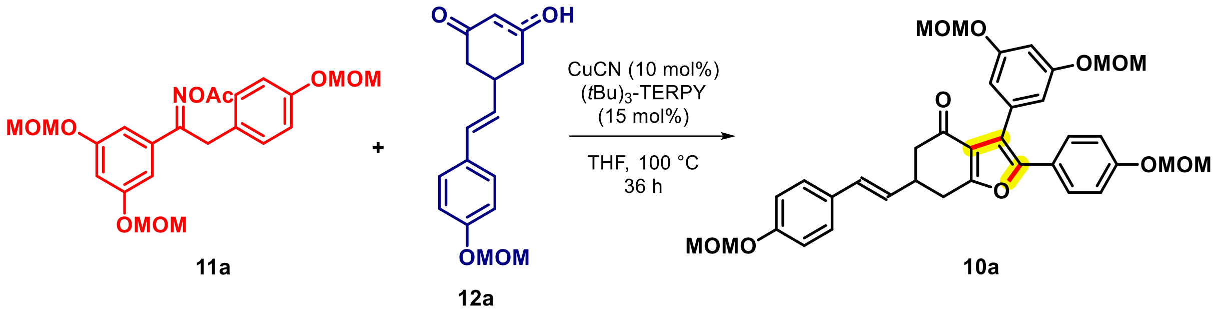

This compound was synthesized using the general procedure for the synthesis of benzofuran-4-one compounds (see page S9) using 65 mg (0.15 mmol, 1 equiv) of oxime acetate **11a** and 60.3 mg (0.22 mmol, 1.5 equiv) of the cyclic 1,3-diketone **12a** in the presence of CuCN (1.3 mg, 0.01 mmol, 0.1 equiv) and (tBu)$_3$-TERPY (9 mg, 0.02 mmol, 0.15 equiv) as ligand for 36 h. The crude product was purified by flash column chromatography on silica gel with 40% EtOAc-hexane as eluent.

(*E*)-3-(3,5-Bis(methoxymethoxy)phenyl)-2-(4-(methoxymethoxy)phenyl)-6-(4-(methoxymethoxy)styryl)-6,7-dihydrobenzofuran-4(5*H*)-one (10a). Yellow oil, 57 mg (60%). **^{1}H NMR ($CDCl_3$, 300 MHz): δ** 7.37 (d, 2H, J = 9.0 Hz), 7.31 (d, 2H, J = 8.8 Hz), 6.99 (d, 2H, J = 8.8 Hz), 6.94 (d, 2H, J = 9.0 Hz), 6.76 (d, 2H, J = 2.3 Hz), 6.73-6.68 (m, 1H), 6.47 (d, 1H, J = 15.8 Hz), 6.14 (dd, 1H, J = 15.9, 6.8 Hz), 5.17 (s, 2H), 5.15 (s, 2H), 5.12 (s, 4H), 3.48 (s, 3H), 3.46 (s, 3H), 3.45 (s, 6H), 3.24-3.12 (m, 2H), 2.91 (dd, 1H, J = 18.1, 11.1 Hz), 2.67 (dd, 1H, J = 16.5, 4.5 Hz), 2.53 (dd, 1H, J = 16.2, 10.9 Hz). **^{13}C{^{1}H} NMR ($CDCl_3$, 75 MHz): δ** 192.6, 164.9, 158.1, 157.0, 149.8, 133.8, 130.8, 130.0, 129.6, 128.0, 127.5, 123.8, 121.1, 118.3, 116.5, 116.2, 111.9, 104.6, 94.8, 94.5, 94.4, 56.2, 45.1, 38.6, 30.3. **IR (ATR) (υ_{max}, cm^{-1}):** 2952.22, 2920.89, 2850.33, 1739.78, 1676.51, 1594.11, 1507.81, 1145.18, 979.82. **HRMS (DART) *m/z*:** [M + H]$^+$ calcd for $C_{36}H_{39}O_{10}$ 631.2543; found 631.2528.

*Synthesis of the benzofuran **27a** via an oxidative aromatization*

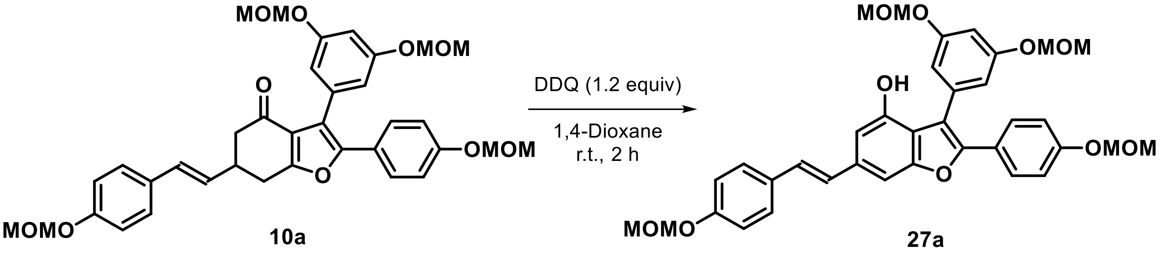

Following the procedure employed to synthesize the benzofuran **27** (see page S21), 20 mg (32 μmol) of **10a** and 8.6 mg (38 μmol) of DDQ were reacted. The crude product was purified by flash column chromatography on silica gel with 50% EtOAc-hexane as eluent.

(*E*)-3-(3,5-Bis(methoxymethoxy)phenyl)-2-(4-(methoxymethoxy)phenyl)-6-(4-(methoxymethoxy)styryl)benzofuran-4-ol (27a). Yellow oil, 12.2 mg (61%). **^{1}H NMR ($CDCl_3$, 300 MHz): δ** 7.54 (d, 2H, J = 9.0 Hz), 7.46 (d, 2H, J = 8.8 Hz), 7.23 (d, 1H, J = 1.3 Hz), 7.06 (d, 2H, J = 2.9 Hz), 7.03 (d, 2H, J = 3.6 Hz), 6.97 (d, 2H, J = 9.0 Hz), 6.88-6.87 (m, 3H), 6.83 (t, 1H, J = 2.3 Hz), 5.20 (s, 2H), 5.17 (s, 6H), 3.50 (s, 3H), 3.48 (s, 9H). **^{13}C{^{1}H} NMR ($CDCl_3$, 75 MHz): δ** 159.5, 157.5, 157.0, 155.4, 150.2, 149.9, 135.9, 135.4, 131.5, 128.1, 127.8, 127.4, 124.1, 117.3, 116.6, 116.3, 114.3, 111.0, 107.3, 105.1, 102.1, 94.6, 94.4,

56.3, 56.2, 56.2. **IR (ATR) (υ_{max}, cm^{-1}):** 3512.73, 3368.72, 2921.57, 2899.62, 2158.67, 1973.98, 1597.76, 1506.68. **HRMS (DART) *m/z*:** $[M + H]^+$ calcd for $C_{36}H_{37}O_{10}$ 629.2386; found 629.2401.

*Synthesis of anigopreissin A **6** via MOM deprotection*

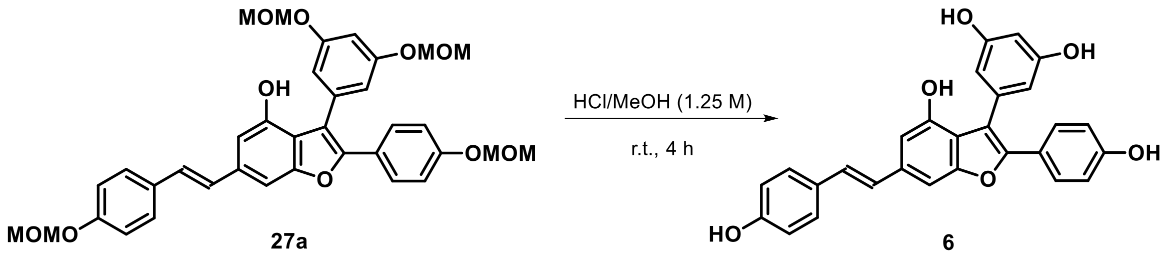

Benzofuran **27a** (28.9 mg, 46 µmol) was dissolved in commercial HCl/MeOH (2 mL, 1.25 M) and stirred at room temperature for 4 h. The solvent was removed under reduced pressure, and the crude residue was adsorbed on silica gel and purified by flash column chromatography on silica gel using a 20% acetone-CH_2Cl_2 mixture as eluent. Following this procedure, 17.8 mg (85%) of anigopreissin A **6** were isolated as a yellow oil.

Synthesis of fuliginosin A 7

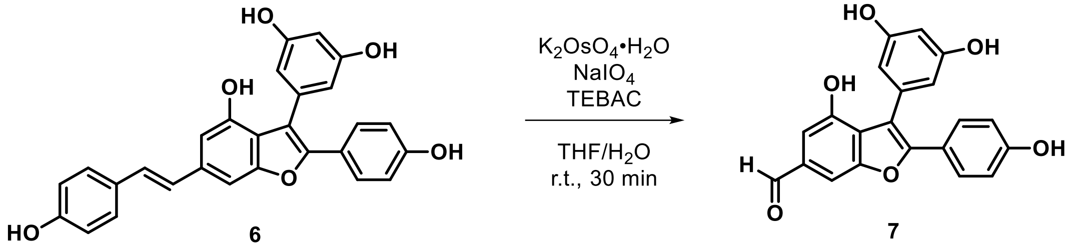

To round-bottom flask with 12 mg (26.5 µmol, 1 equiv) of **6**, 6 mL (4.42 mM) of THF/H2O (5:1) were added $NaIO_4$ (22.7 mg, 106 µmol, 4 equiv), $K_2OsO_4{\cdot}H_2O$ (9.3 mg, 26.5 µmol, 1 equiv) and TEBAC (1.2 mg, 5.3 µmol, 0.2 equiv) at room temperature and stirred for 30 min. The reaction mixture was extracted with EtOAc (3 × 10 mL). The combined organic layers were dried over Na_2SO_4 and concentrated under reduced pressure. The crude product was purified by preparative plate using a mixture of 10% acetone-CH_2Cl_2 mixture as eluent.

3-(3,5-Dihydroxyphenyl)-4-hydroxy-2-(4-hydroxyphenyl)benzofuran-6-carbaldehyde (fuliginosin A, 7). Dark brown oil, 2.9 mg (8 µmol, 30%). **^{1}H NMR (acetone-*d*$_6$, 700 MHz): δ** 10.00 (s, 1H), 8.82 (s, 1H), 8.63 (s, 1H), 8.35 (s, 2H), 7.66 (d, 1H, *J* = 1.2 Hz), 7.53 (d, 2H, *J* = 8.8 Hz), 7.20 (d, 1H, *J* = 1.2 Hz), 6.85 (d, 2H, *J* = 8.8 Hz), 6.50 (d, 2H, *J* = 2.3 Hz), 6.44 (t, 1H, *J* = 2.2 Hz). **^{13}C{^{1}H} NMR (acetone-*d*$_6$, 175 MHz): δ** 192.0, 159.5, 159.2, 155.6, 154.1, 153.1, 135.8, 135.1, 129.5, 124.7, 122.4, 116.6, 116.3, 109.9, 108.2, 106.7, 103.1. **IR (ATR) (υ_{max}, cm^{-1}):** 3409.54, 2924.62, 1706.19, 1360.66, 1221.65. **HRMS (EI, 70eV) *m/z*:** $[M]^+$ calcd for $C_{21}H_{14}O_6$ 362.0790; found 362.0796.

Table S10. *Comparative ¹H/¹³C NMR data of the isolated natural product, reported sample, and this work*

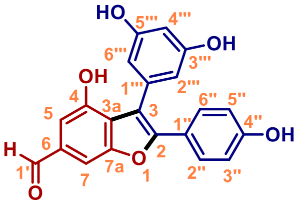

7

Fuliginosin A

Position	Isolated natural product ¹H NMR DMSO-*d*₆, 500 MHz[20]	Reported synthetic sample ¹H NMR DMSO-*d*₆ 500 MHz[21]	Synthesized in this work ¹H NMR Acetone-*d*₆, 700 MHZ	Isolated natural product ¹³C{¹H} NMR DMSO-*d*₆, 500 MHz[20]	Reported synthetic sample ¹³C{¹H} NMR DMSO-*d*₆ 500 MHz[21]	Synthesized in this work ¹³C{¹H} NMR Acetone-*d*₆, 700 MHZ
**1**	-	-	-	-	-	-
**2**	-	-	-	152.9	152.8	153.1
**3**	-	-	-	116.1	116.1	116.6
**3a**	-	-	-	missing	134.7	135.8
**4**	N.D.	N.D.	8.82 (s, 1H, OH)	152.8	152.8	154.1
**5**	7.07, (s, 1H)	7.08, (d, 1H, *J* = 0.8 Hz)	7.20 (d, 1H, *J* = 1.2 Hz)	107.3	107.2	108.2
**6**	-	-	-	124.0	124.0	124.7
**7**	7.61 (s, 1H)	7.64, (d, 1H, *J* = 0.8 Hz)	7.66 (d, 1H, *J* = 1.2 Hz)	106.2	106.4	106.7
**7a**	-			154.5	154.5	155.6
**1’**	9.91 (s, 1H)	9.92, (s, 1H)	10.0 (s, 1H)	192.6	192.7	192.0
**1’’**	-			120.9	120.9	122.4
**2’’, 6’’**	7.35 (d, 2H, *J*= 9.0 Hz)	7.35, (d, 2H, *J* = 6.3 Hz)	7.53 (d, 2H, *J* = 8.8 Hz)	128.7	128.7	129.5
**3’’, 5’’**	6.73 (d, 2H, *J*= 9.0 Hz)	6.74, (d, 2H, *J* = 6.3 Hz)	6.85 (d, 2H, *J* = 8.8 Hz)	116.0	116.0	116.3
**4’’**	N.D.	N.D.	8.63 (s, 1H, OH)	158.7	158.7	159.2
**1’’’**	-	-	-	133.9	133.9	135.1
**2’’’, 6’’’**	6.22 (d, 2H, *J* = 2.0 Hz)	6.23, (d, 2H, *J* = 1.7 Hz)	6.50 (d, 2H, *J* = 2.3 Hz)	109.0	109.0	109.9
**3’’’, 5’’’**	N.D.	N.D.	8.35 (s, 2H, OH)	158.5	158.6	159.5
**4’’’**	6.20 (dd, 1H, *J*= 2.0 Hz)	6.21, (d, 1H, *J* = 1.7 Hz)	6.44 (t, 1H, *J* = 2.2 Hz)	102.4	102.4	103.1

N.D.: No detected.

Table S11. *Study of the oxidative cleavage of benzofuran* ***27a***

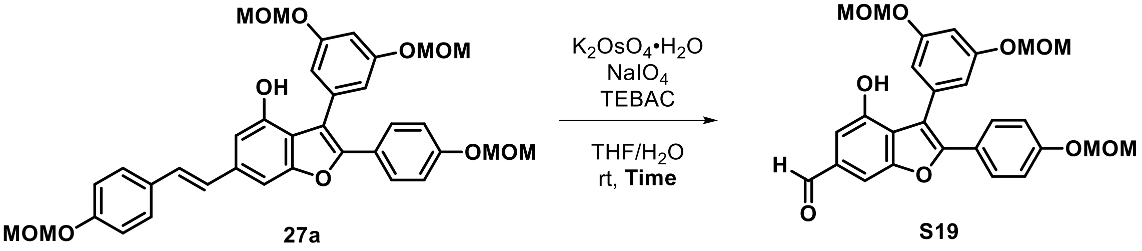

Entry	Time(h)	Yield[a] S19 (%)
1	0.5	39
2	1.5	M.C.
3	1	M.C.

27a (15.6 µmol), $K_2OsO_4{\cdot}H_2O$ (15.6 µmol), $NaIO_4$ (62.35 µmol), TEBAC (3.12 µmol) in 6 mL (4.42 mM) of THF/H_2O (5:1). [a] Isolated yield. M.C. = Complex mixture.

Following the procedure employed to synthesize fuliginosin A **7** via an oxidative cleavage (see page S30), 9.8 mg (15.6 µmol) of **27a**, 13.3 mg (62.35 µmol) $NaIO_4$, 5.4 mg (15.6 µmol) of $K_2OsO_4{\cdot}H_2O$ and 0.7 mg (3.12 µmol) of TEBAC were reacted. The crude product was purified by preparative plate using a mixture of 10% acetone-CH_2Cl_2 mixture as eluent.

3-(3,5-Bis(methoxymethoxy)phenyl)-4-hydroxy-2-(4-(methoxymethoxy)phenyl)benzofuran-6-carbaldehyde (S19). Yellow oil, 3 mg (39%). **^{1}H NMR ($CDCl_3$, 300 MHz): δ** 9.99 (s, 1H), 7.64 (d, 1H, J = 1.2 Hz), 7.57 (d, 2H, J = 9.0 Hz), 7.21 (d, 1H, J = 1.2 Hz), 6.99 (d, 2H, J = 9.1 Hz), 6.86 (m, 3H), 5.44 (br s, 1H), 5.19 (s, 2H), 5.18 (s, 4H), 3.48 (s, 9H). **^{13}C{^{1}H} NMR ($CDCl_3$, 75 MHz): δ** 191.6, 159.6, 158.1, 154.5, 153.3, 150.7, 134.4, 134.3, 128.6, 123.2, 116.4, 114.3, 110.9, 109.7, 106.3, 105.4, 94.6, 94.3, 56.4, 56.3. **IR (ATR) (υ_{max}, cm^{-1}):** 3337.11, 2922.02, 1687.18, 1591.01, 1508.59. **HRMS (DART) *m/z*:** [M + H]$^+$ calcd for $C_{27}H_{27}O_9$ 495.1655; found 495.1665.

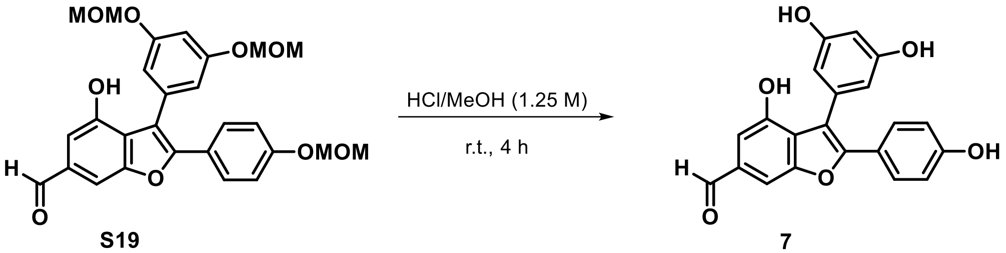

Following the procedure employed to deprotect anigopreissin A **6** (see page S30), 3 mg (6 µmol) of compound **S19** was deprotected. The crude residue was purified by preparative TLC using a mixture of 10% acetone-CH_2Cl_2 mixture as eluent to afford 1.7 mg of fuliginosin A **7** (4.7 µmol, 77%).

7. Control experiments

Additionally, we performed the following experiments to rule out the participation of radical intermediates during the heterocyclization reaction.

Table S12. *Effect of additives on the formation of benzofuran* ***24a****.*

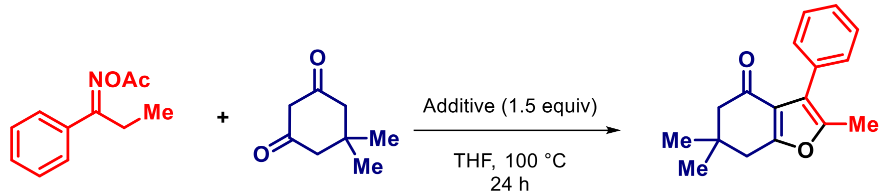

22a 23a 24a

Entry	Additive	Yield[a] (%)
1	-	83
2	TEMPO	19
3	BHT	81
4	1,1-Diphenylethylene	46

Reaction conditions: **22a** (0.15 mmol), **23a** (0.17 mmol), [CuCN] 10 mol%, 4,4′-bis(*tert*-butyl)-2,2′-bipyridine (15 mol%) in 2 mL of anhydrous THF [0.075 M]. [a] Isolated yield.

We found that the addition of an external oxidant such as TEMPO significantly inhibited the formation of **24a** and the presence of non-oxidizing radical scavengers, such as *tert*-butylhydroxytoluene (BHT) and 1,1-diphenylethylene, resulted in the formation of **24a** in 81% and 46% yields, respectively. Given that total inhibition was not observed in any of the assays and no radical-trapping adducts were isolated, the involvement of radical species in the reaction pathway was discarded (see Table S12).

8. Crystallographic data of benzofuran-4-one 24v

Single crystal **24v** were obtained by slow vapor diffusion, using ethyl acetate as the solvent for the sample and pentane as the external diffusion solvent. To control the diffusion rate, a cotton plug was placed at the vial opening. Data collection was performed on a Bruker APEX-II CCD diffractometer using Mo-Kα radiation (λ= 0.7107 Å).

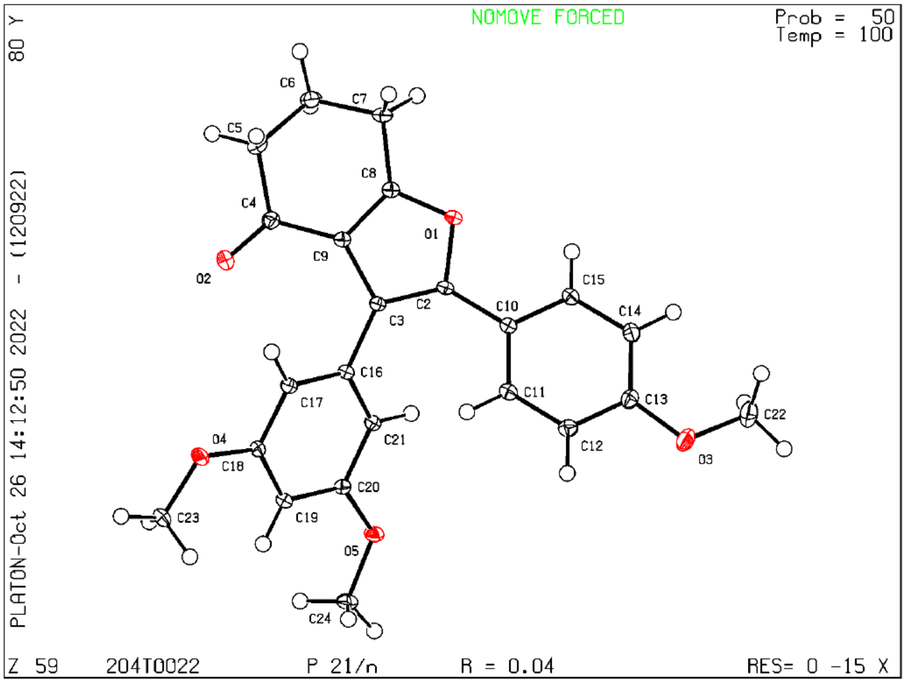

Supplementary Figure 1. ORTEP drawing of **24v**. Ellipsoids are drawn at the 50% probability level. CCDC deposition number 2504181 contains the supplementary crystallographic data for this paper, which can be obtained free of charge at https://www.ccdc.cam.ac.uk/structures/

Bond precision: C-C = 0.0012 A Wavelength=0.71073

Cell:	a=7.7508(3)	b=11.2756(4)	c=20.8559(8)
	alpha=90	beta=95.718(1)	gamma=90
Temperature:	100 K		

	Calculated	Reported
Volume	1813.63(12)	1813.63(12)
Space group	P 21/n	P 21/n
Hall group	-P 2yn	-P 2yn
Moiety formula	C23 H22 O5	C23 H22 O5
Sum formula	C23 H22 O5	C23 H22 O5
Mr	378.41	378.40
Dx,g cm-3	1.386	1.386
Z	4	4
Mu (mm-1)	0.097	0.097
F000	800.0	800.0
F000'	800.43	
h,k,lmax	11,16,30	11,16,30
Nref	6039	6035
Tmin,Tmax	0.968,0.994	0.711,0.746
Tmin'	0.968	

Correction method= # Reported T Limits: Tmin=0.711 Tmax=0.746
AbsCorr = MULTI-SCAN

Data completeness= 0.999 Theta(max)= 31.505

R(reflections)= 0.0361(5348) wR2(reflections)= 0.1034(6035)

S = 1.026 Npar= 256

9. References

1. Ando, K.; Yamada, K. Solvent-free Horner–Wadsworth–Emmons reaction using DBU. *Tetrahedron Lett.* **2010**, *51* (25), 3297–3299. DOI:10.1016/j.tetlet.2010.04.072.
2. Nguyen, S. T.; Murray, P. R. D.; Knowles, P. R. Light-Driven Depolymerization of Native Lignin Enabled by ProtonCoupled Electron Transfer. *ACS Catal*. **2020**, *10* (1), 800–805. DOI: 10.1021/acscatal.9b04813.
3. Kumar, N. S. S.; Varghese, S.; Narayan, G.; Das, S. Hierarchical Self-Assembly of Donor-Acceptor-Substituted Butadiene Amphiphiles into Photoresponsive Vesicles and Gels. *Angew. Chem. Int. Ed*. **2006**, *45* (38), 6317–6321. DOI: 10.1002/anie.200602088.
4. El-Deeb, I. Y.; Funakoshi, T.; Shimomoto, Y.; Matsubara, R.; Hayashi, M. Dehydrogenative Formation of Resorcinol Derivatives Using Pd/C-Ethylene Catalytic System. *J. Org. Chem*. **2017**, *82* (5), 2630–2640. DOI: 10.1021/acs.joc.6b03037.
5. Wiffen, J. W.; Mccague, R. Preparation of Polyhydroxylated Stil-benes, WO 2008012108 A2, 2008.
6. Schouteeten, A.; Jus, S.; Vallejos, J.-C. Novel process for the synthesis of (E)-stilbene derivatives including resveratrol and piceatannol, WO2008012321 A1, 2008.
7. Jin, S.; Lin, C.; Wang, Y.; Wang, H.; Wen, X.; Xiao, P.; Li, X.; Peng, Y.; Sun, J.; Lu, Y.; Wang, X. Cannabidiol Analogue CIAC001 for the Treatment of Morphine-Induced Addiction by Targeting PKM2. *J. Med. Chem.* **2023**, *66* (16), 11498–11516. DOI: 10.1021/acs.jmedchem.3c01029.
8. Jana, N.; Nanda, S. Asymmetric Total Syntheses of Cochliomycin A and Zeaenol. *Eur. J. Org. Chem.* **2012**, *2012* (23), 4313–4320. DOI:10.1002/ejoc.201200241.
9. Ramos Orea, A.; Torres-Ochoa, R. O. Synthesis of Furoquinolinones/Pyranones/Coumarins via a Copper-Catalyzed Heteroannulation of Oxime Acetates and 1,3-Cyclic Dicarbonyls. *Tetrahedron* **2024**, *166*, 134212. DOI: 10.1016/j.tet.2024.134212.
10. Yang, H.-T.; Zhou, S.-Q.; Chen, D.-M.; Hu, Z.-J.; Qiang, X.-Q.; Song, X.-Q.; Tan, S.; Jiang, W.-H.; Sun, Y.-Q.; Miao, C.-B. Copper-Catalyzed Annulation of *O*-acyl Oximes with Cyclic 1,3-Diones for the Synthesis of 7,8-Dihydroindolizin-5(6H)-ones and Cyclohexanone-Fused Furans. *Org. Lett.* **2023**, *25* (5), 838–842. DOI: 10.1021/acs.orglett.3c00003.
11. Ghosh, M.; Santra, S.; Mondal, P.; Kundu, D.; Hajra, A. Diversified Synthesis of Furans by Coupling between Enols/1,3-Dicarbonyl Compounds and Nitroolefins: Direct Access to Dioxa[5]helicenes. *Chem. - Asian J.* **2015**, *10* (11), 2525–2536. DOI: 10.1002/asia.201500710.
12. Ding, Z.-J.; Zhang, F.; Xu, T.-X.; Wang, W.-B.; Jin, L.; Huang, N.-Y. Solvent-free synthesis and crystal structure of 2,2'-dialkyl bis(3-methyl-6,7-dihydrobenzofuran-4(5H)-one) derivatives. *Chin. J. Struct. Chem*. **2017**, *36* (7), 1124–1129. DOI: 10.14102/j.cnki.0254-5861.2011-1480.
13. Van Hulssen, C. J. Evodone, a crystalline ketone from the essential oil of *Evodia hortensis*. *De Ing. Nederland-Indie* **1941**, 8, 89–91.
14. Hölscher, D.; Schneider, B. A resveratrol dimer from *Anigozanthos preissii* and *Musa cavendish*. *Phytochemistry*. **1996**, *43* (2), 471–473. DOI: 10.1016/0031-9422(96)00317-2.
15. Vo, D. D.; Elofsson, M. Total Synthesis of Viniferifuran, Resveratrol-Piceatannol Hybrid, Anigopreissin A and Analogues – Investigation of Demethylation Strategies. *Adv. Synth. Catal.* **2016**, *358* (24), 4085–4092. DOI: 10.1002/adsc.201601089.

16. Zhao, X.; Fang, J.; Jia, Y.; Wu, Z.; Zhang, M.; Xia, M.; Dong, J. Synthesis and Anti-Neuroinflammatory Activity of 1,7-diphenyl-1,4-heptadien-3-ones in LPS-Stimulated BV2 Microglia via Inhibiting NF-κB/MAPK Signaling Pathways. *Molecules* **2022**, *27* (11), 3537. DOI: 10.3390/molecules27113537.
17. Jirásek, P.; Amslinger, S.; Heilmann, J. Synthesis of Natural and Non-natural Curcuminoids and Their Neuroprotective Activity against Glutamate-Induced Oxidative Stress in HT-22 Cells. *J. Nat. Prod.* **2014**, *77* (10), 2206–2217. DOI: 10.1021/np500396y.
18. Andrus, M. B.; Liu, J. Synthesis of polyhydroxylated ester analogs of the stilbene resveratrol using decarbonylative Heck couplings. *Tetrahedron Lett.* **2006**, *47* (32), 5811–5814. DOI: 10.1016/j.tetlet.2006.05.065.
19. Baek, K. H.; Karki, R.; Lee, E. S.; Na, Y.; Kwon, Y. Synthesis and investigation of dihydroxychalcones as calpain and cathepsin inhibitors. *Bioorg. Chem*. **2013**, *51*, 24–30. DOI: 10.1016/j.bioorg.2013.09.002.
20. Brkljača, R.; White, J. M.; Urban, S. Phytochemical Investigation of the Constituents Derived from the Australian Plant *Macropidia fuliginosa*. *J. Nat. Prod.* **2015**, *78* (7), 1600–1608. DOI: 10.1021/acs.jnatprod.5b00161.
21. Liu, J.-T.; Simmons, C. J.; Xie, H.; Yang, F.; Zhao, X.-L.; Tang, Y.; Tang, W. Synthesis of Highly Substituted Benzofuran–containing Natural Products via Rh–Catalyzed Carbonylative Benzannulation. *Adv. Synth. Catal.* **2017**, *359* (4), 693–697. DOI: 10.1002/adsc.201600992.

10. ^{1}H and ^{13}C NMR spectra

^{1}H NMR ($CDCl_3$, 300 MHz) of **S3**.

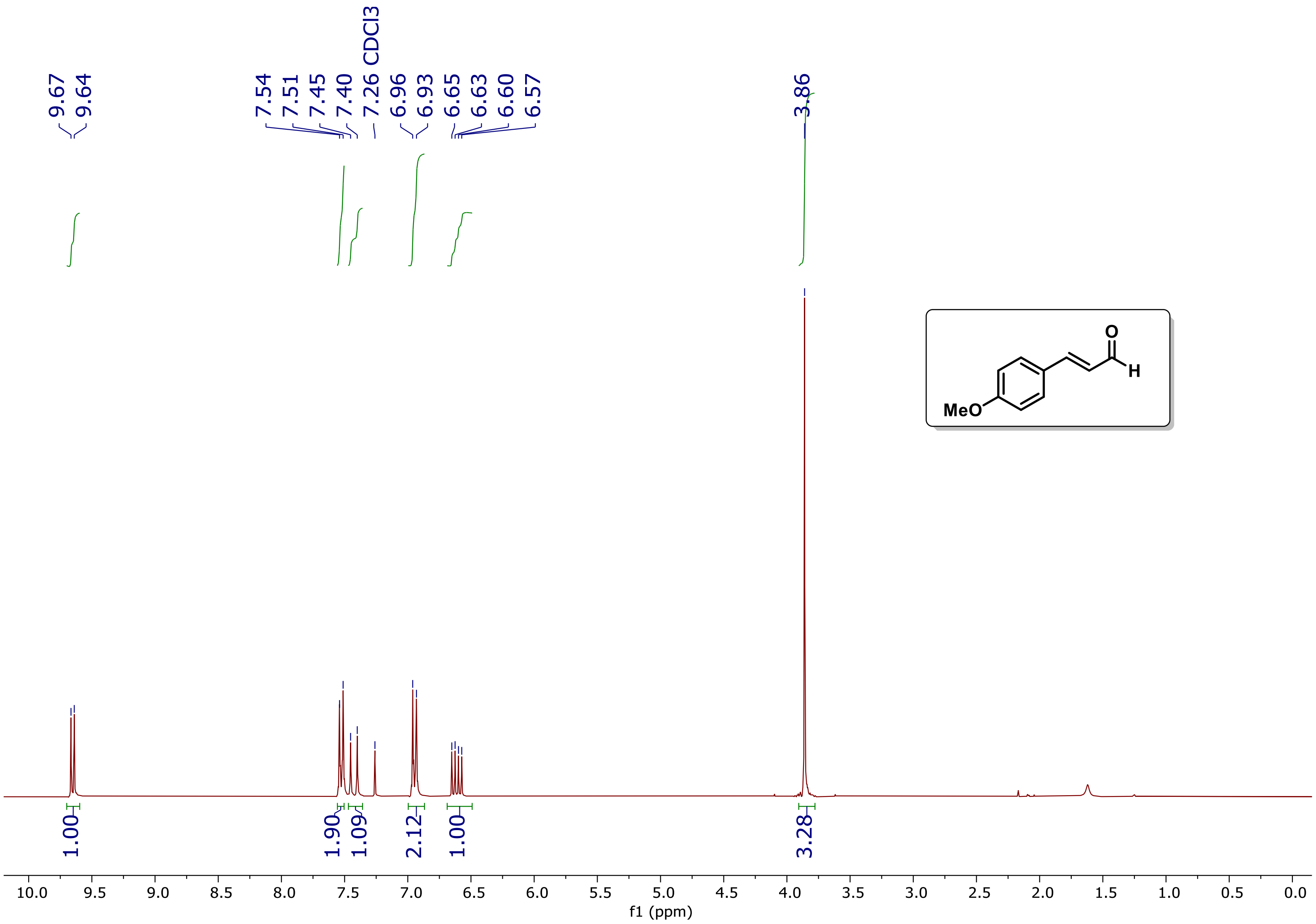

^{1}H NMR ($CDCl_3$, 300 MHz) of **19**.

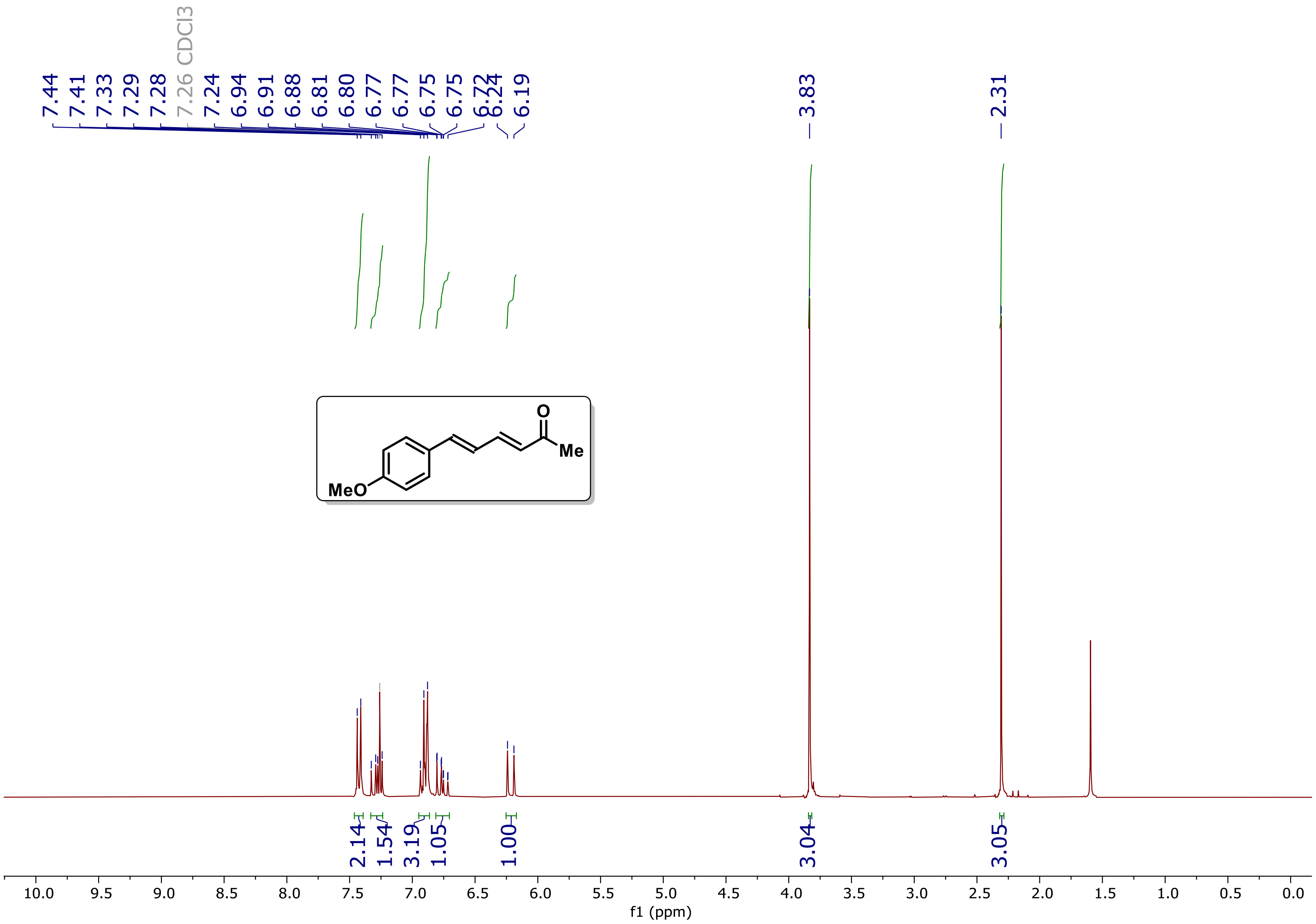

^{1}H NMR (DMSO-d_6, 300 MHz) of **12**.

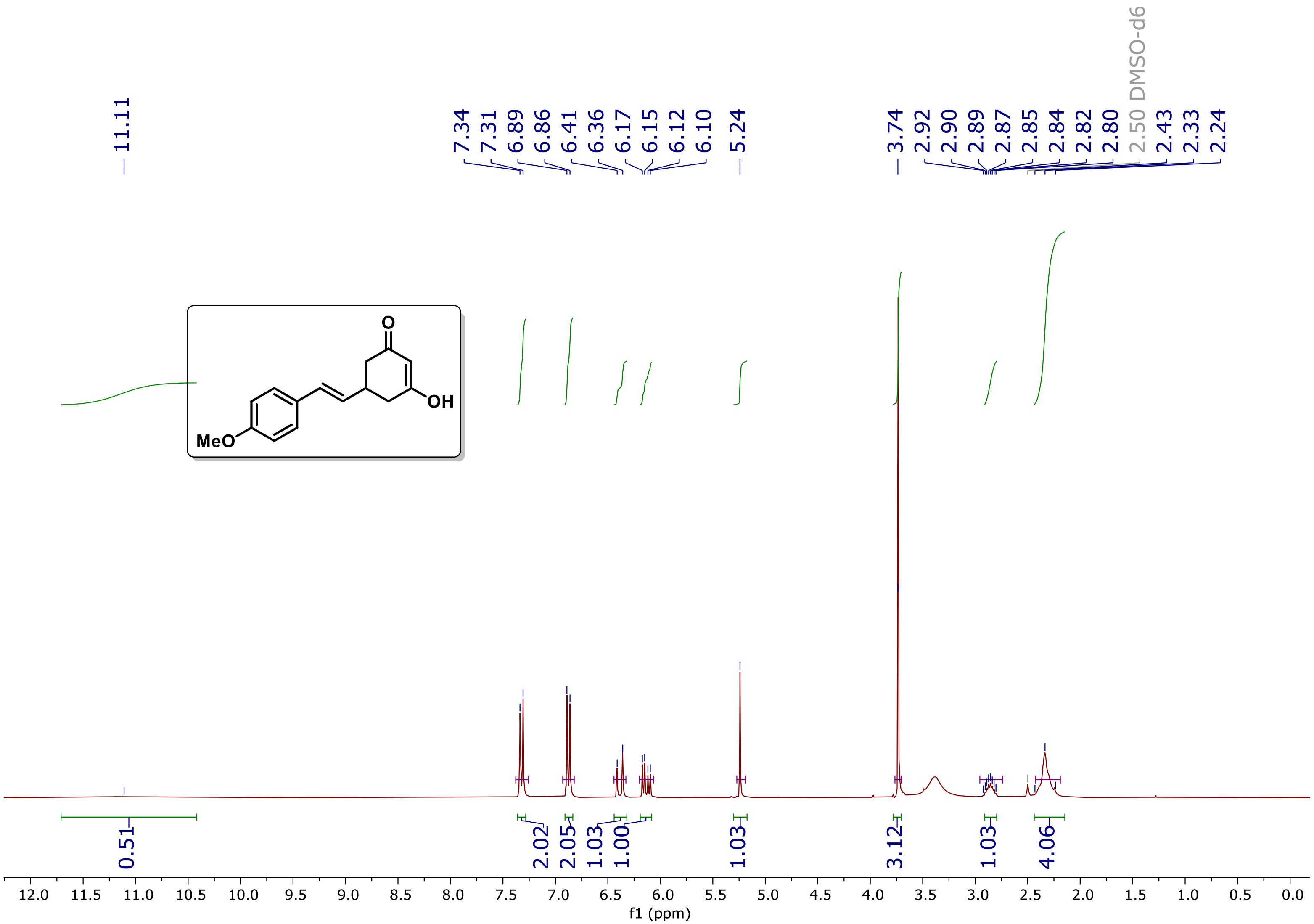

^{1}H NMR ($CDCl_3$, 300 MHz) of **13**.

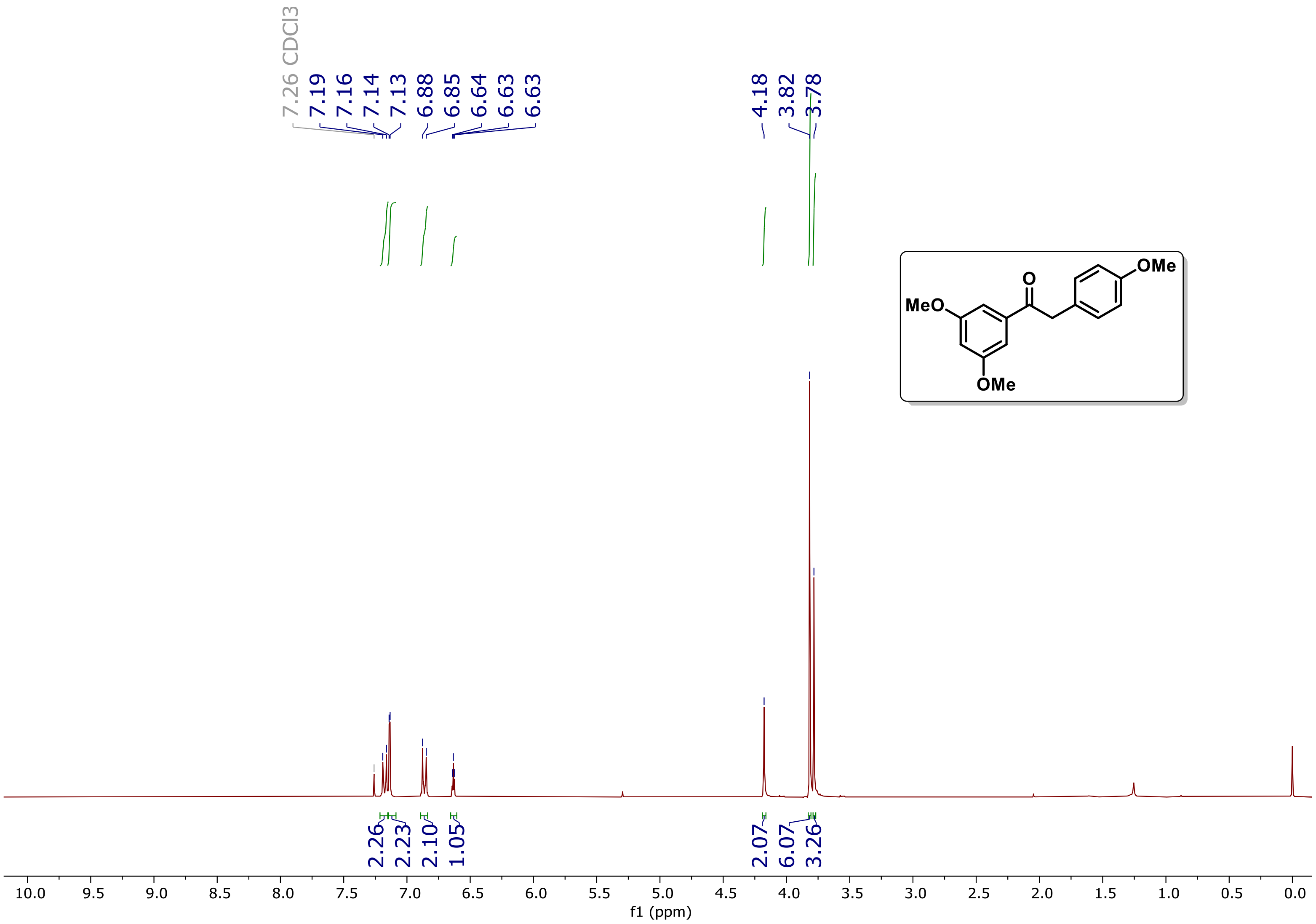

$^{13}C\{^{1}H\}$ NMR ($CDCl_3$, 75 MHz) of **13**.

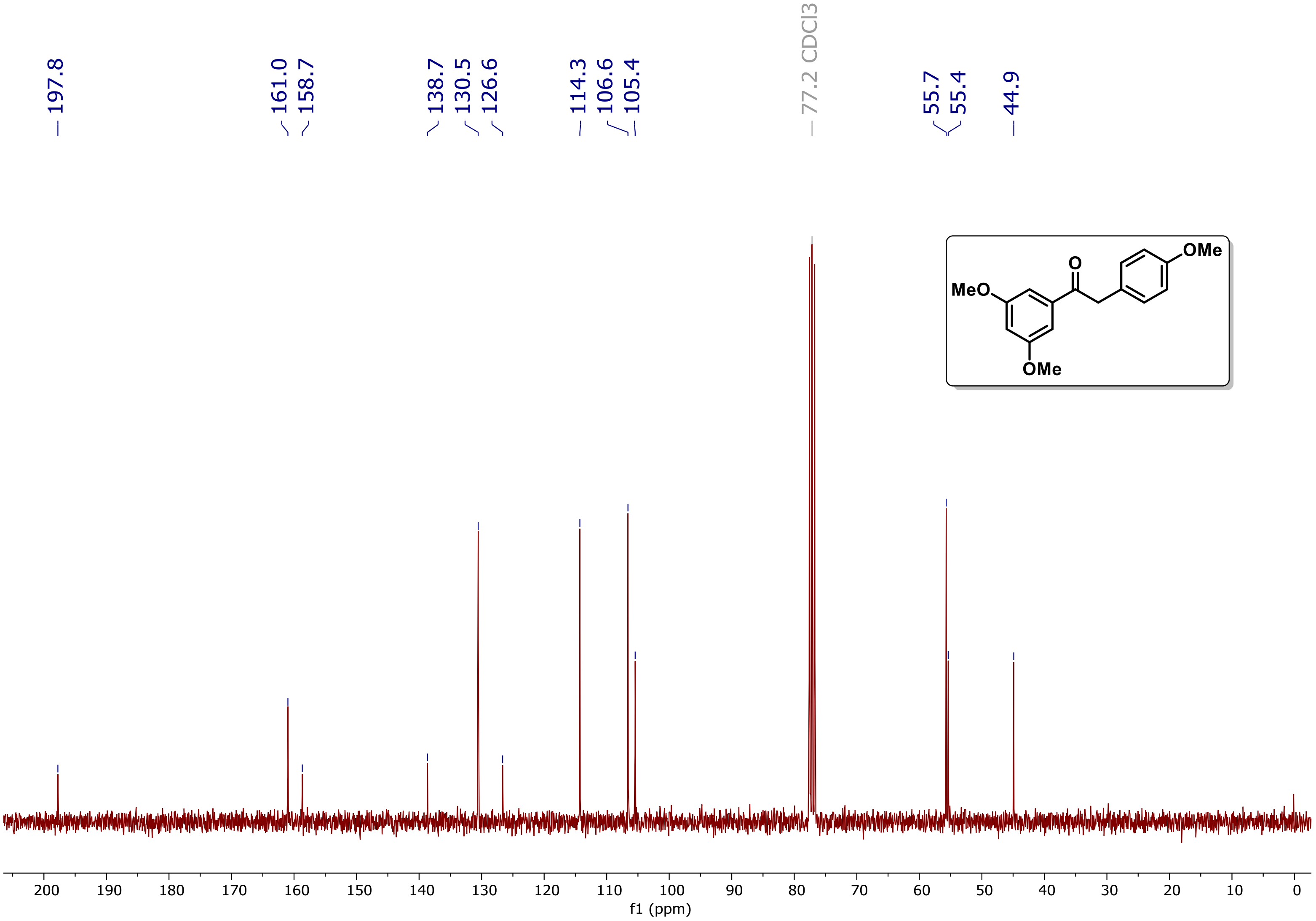

^{1}H NMR ($CDCl_3$, 300 MHz) of **S4**.

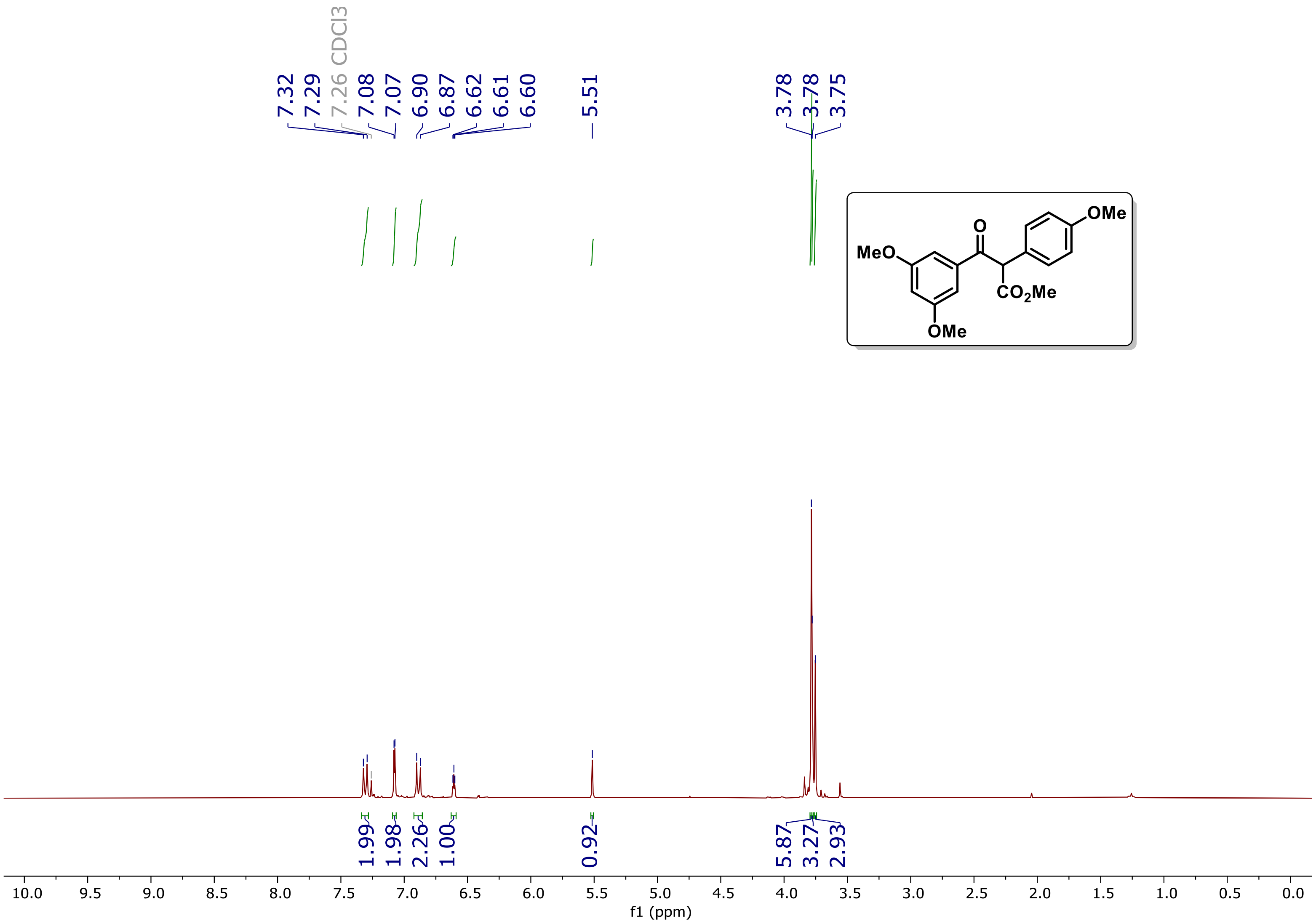

^{1}H NMR ($CDCl_3$, 400 MHz) of **S5**.

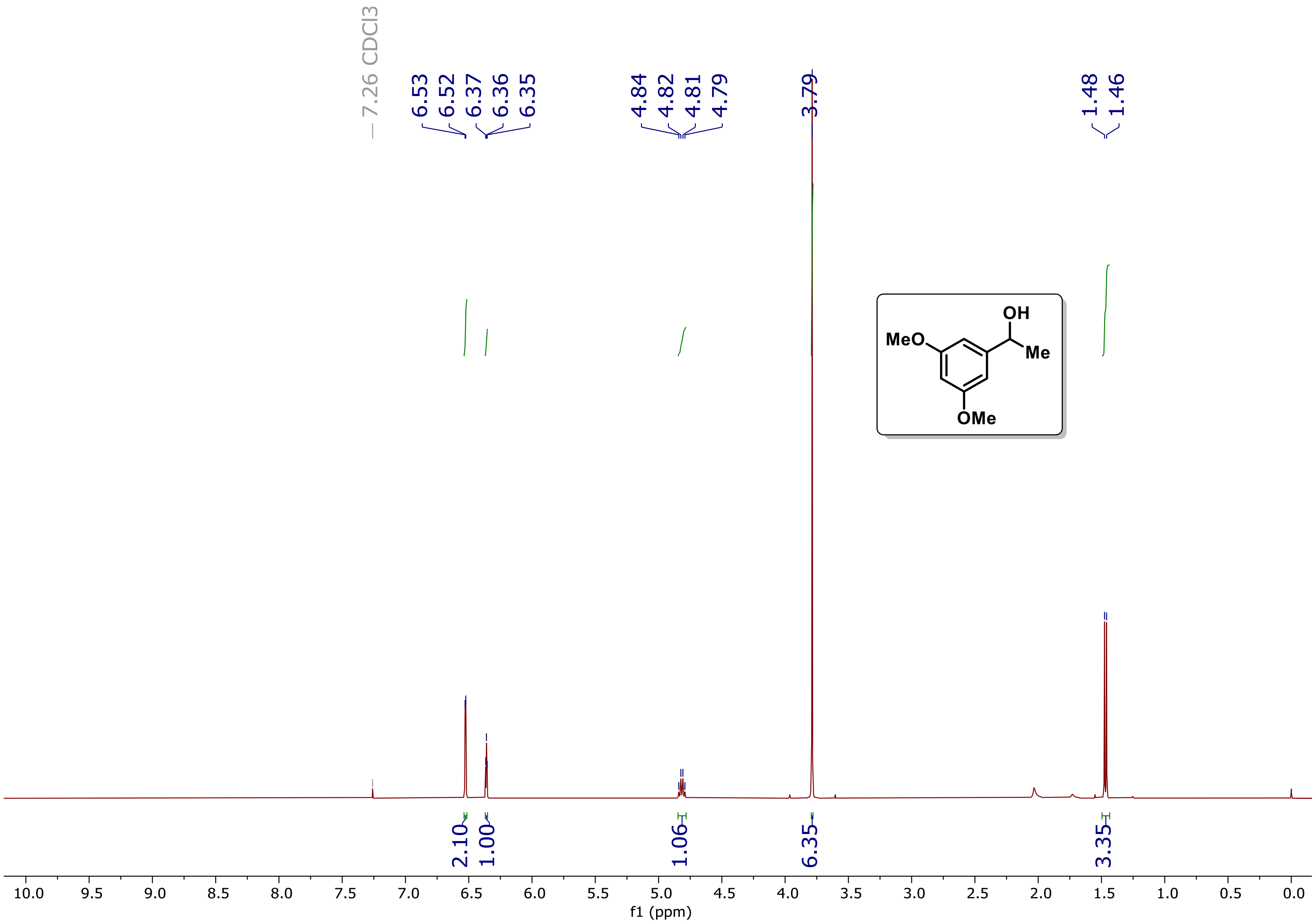

^{1}H NMR ($CDCl_3$, 400 MHz) of **21**.

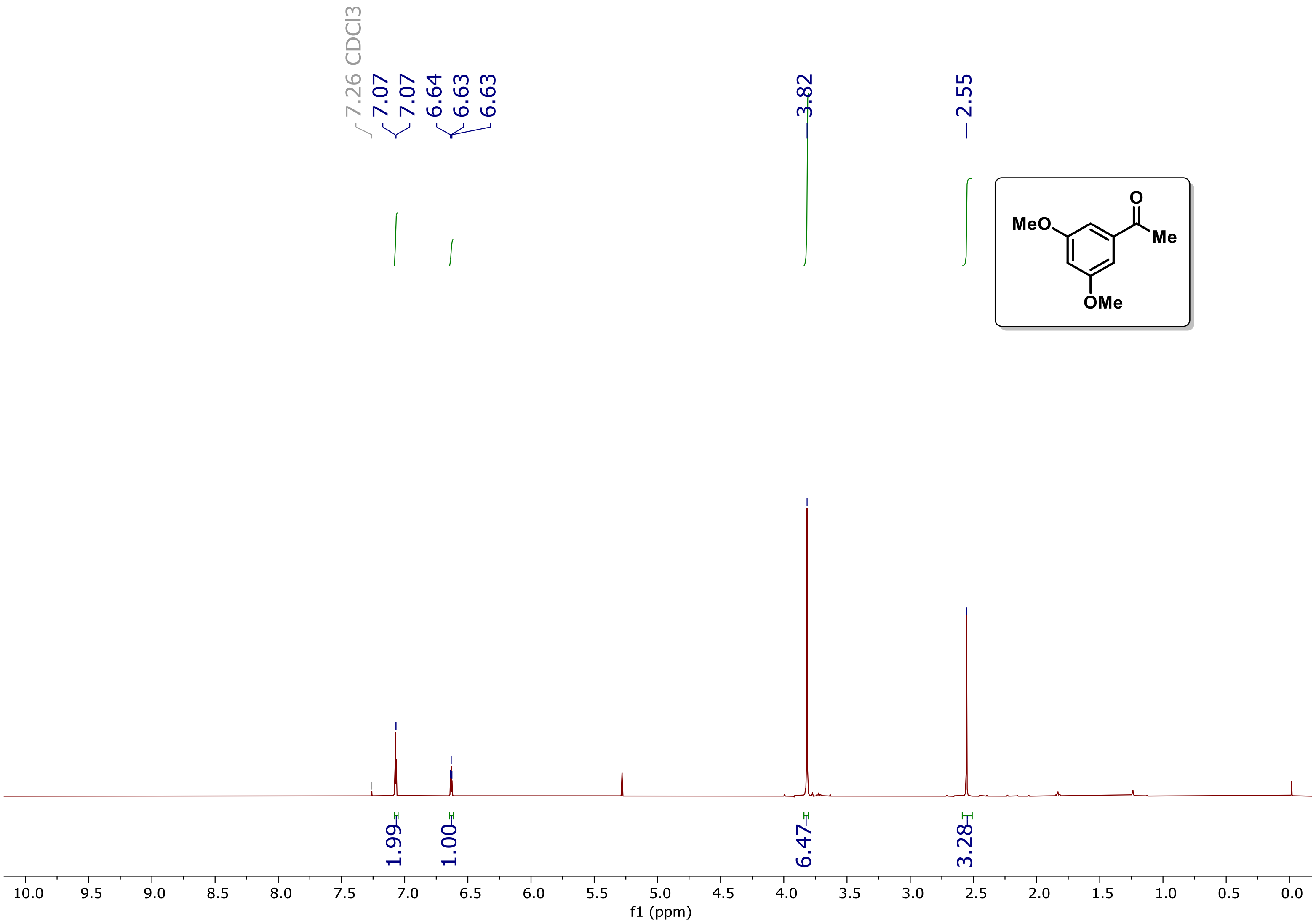

^{1}H NMR (CDCl$_3$, 300 MHz) of **S8**.

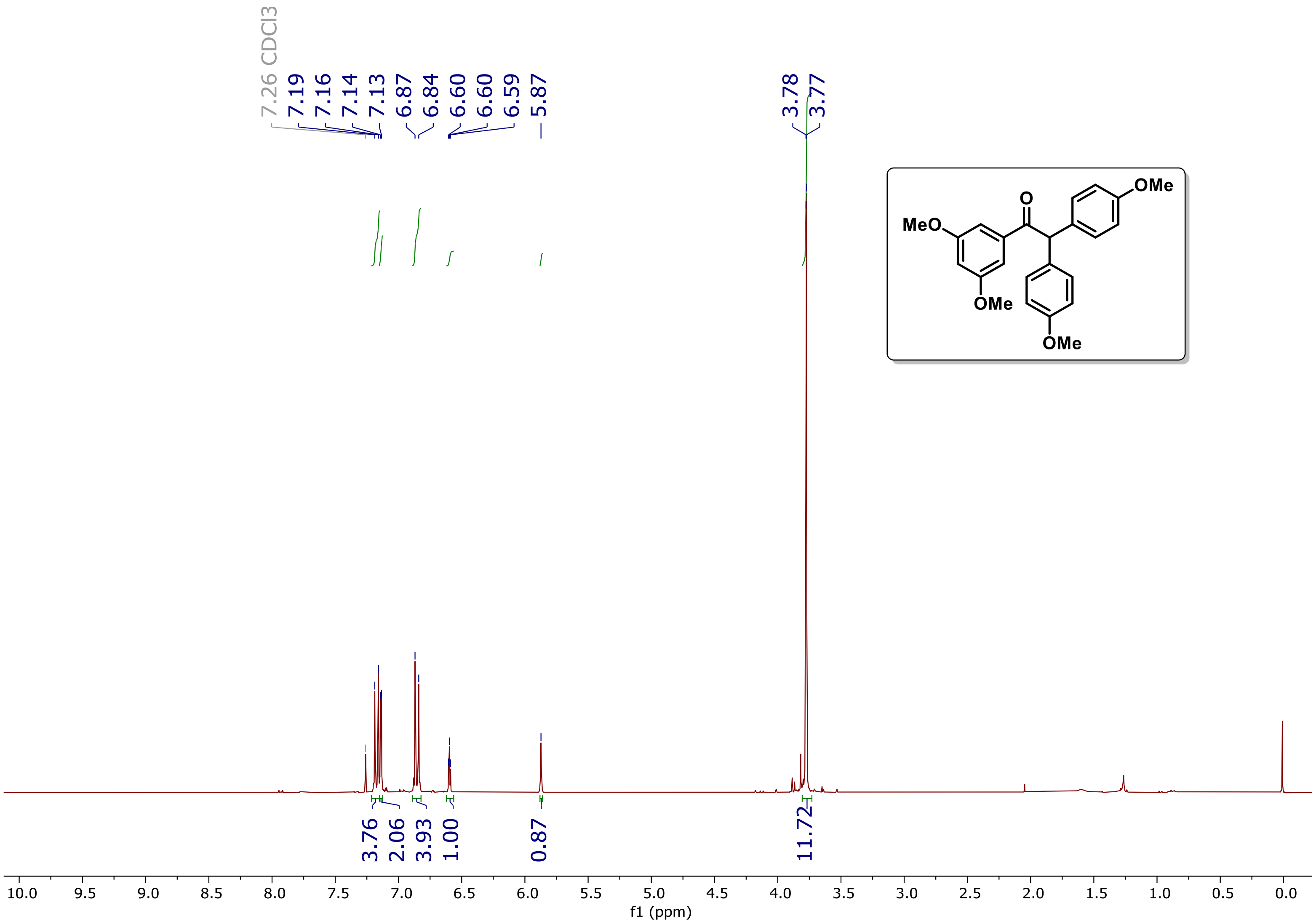

$^{13}C\ \{^1H\}$ NMR ($CDCl_3$, 75 MHz) of **S8**.

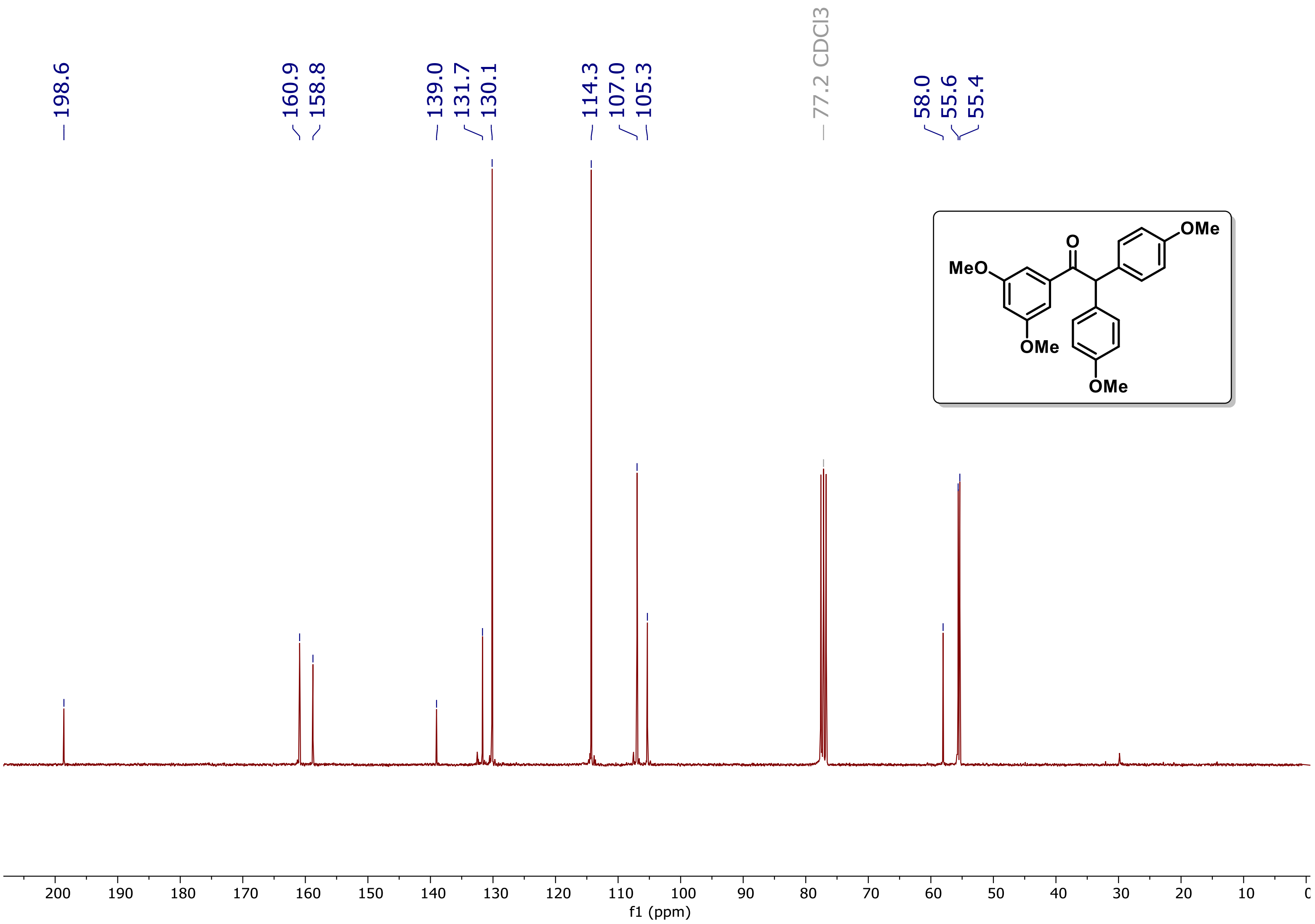

^{1}H NMR ($CDCl_3$, 300 MHz) of **11**.

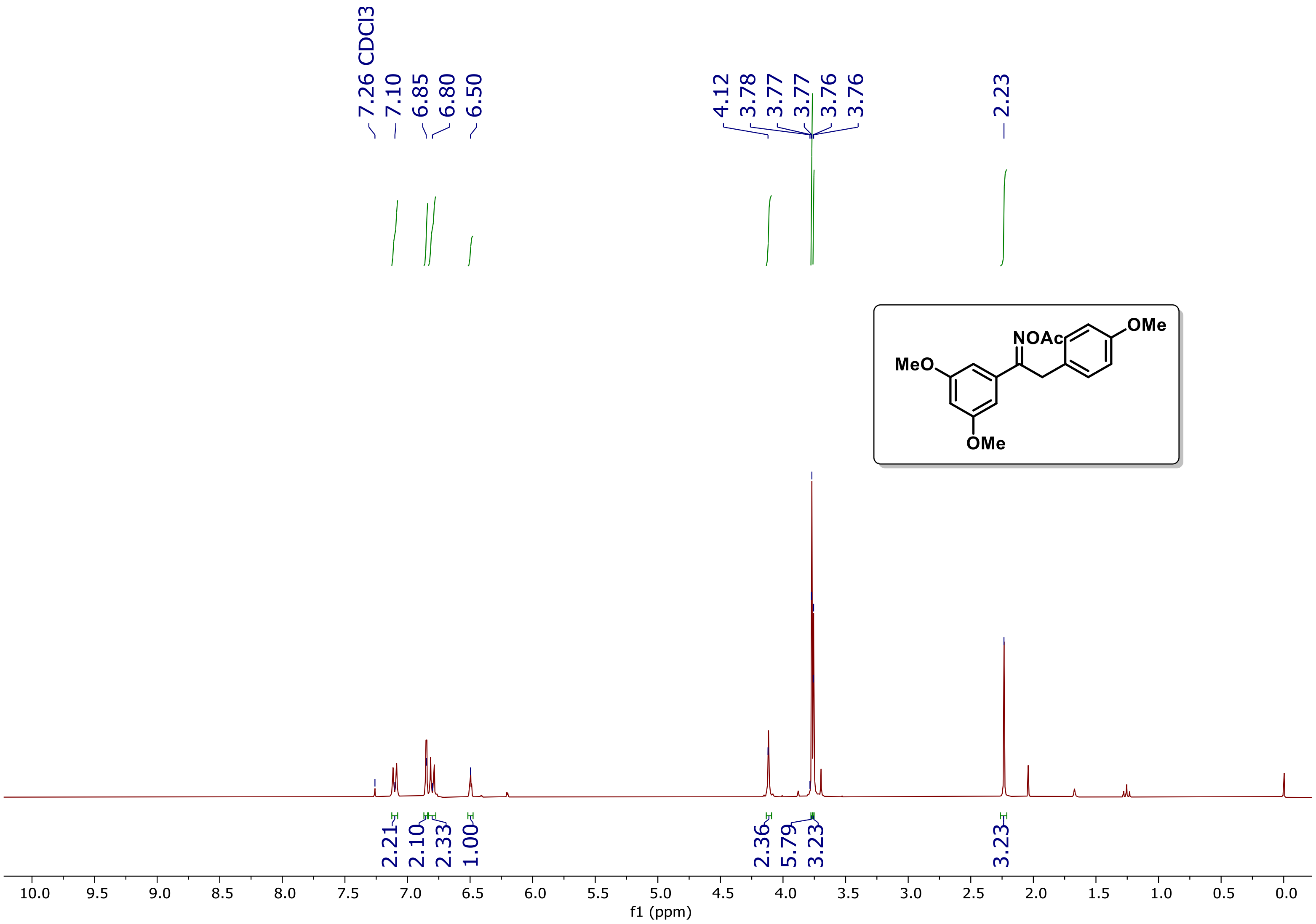

^{1}H NMR ($CDCl_3$, 300 MHz) of **10**.

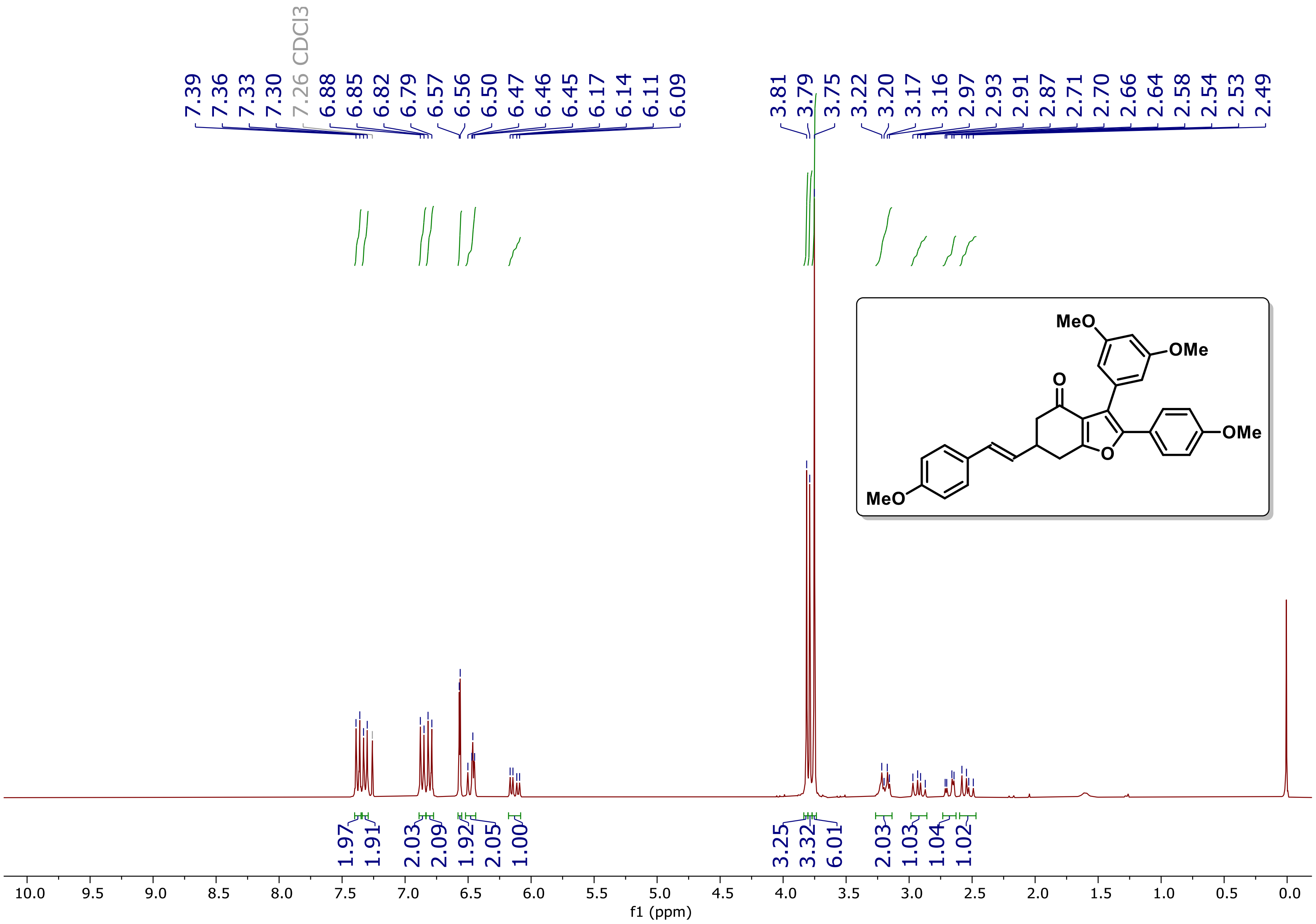

$^{13}C\{^1H\}$ NMR ($CDCl_3$, 75 MHz,) of **10**.

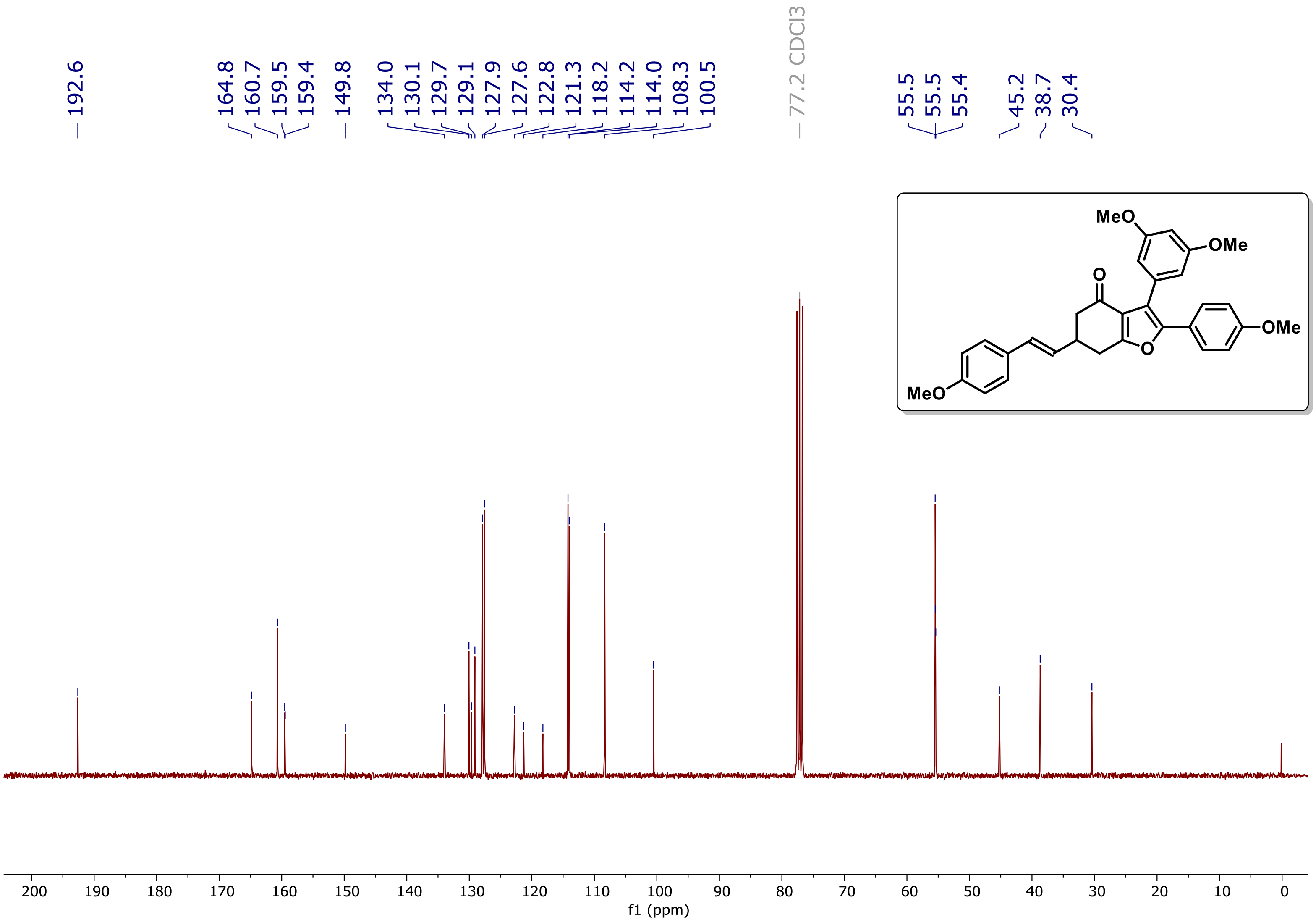

^{1}H NMR ($CDCl_3$, 300 MHz) of **22m**.

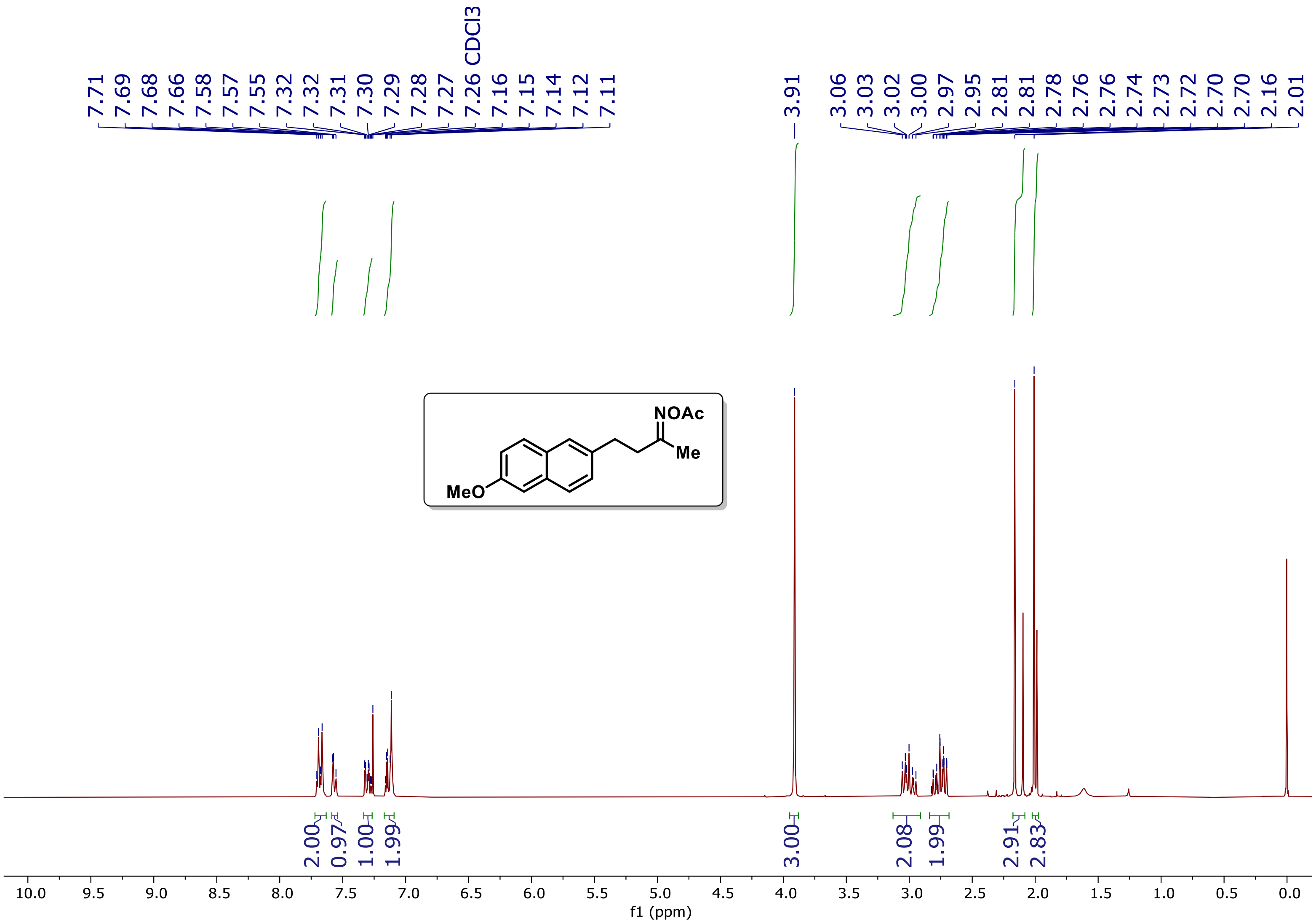

$^{13}C\{^{1}H\}$ NMR ($CDCl_3$, 75 MHz) of **22m**.

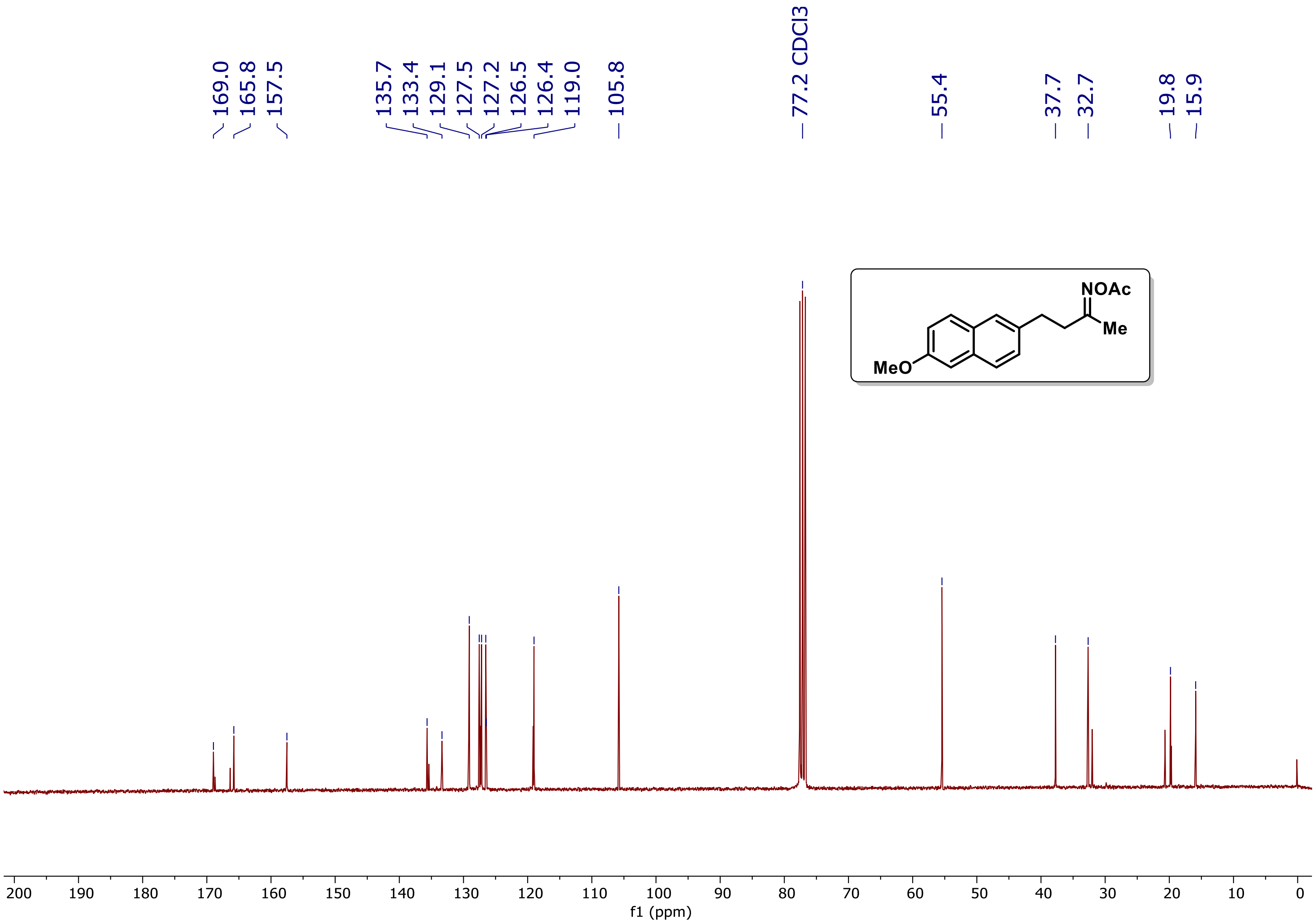

^{1}H NMR ($CDCl_3$, 300 MHz) of **S12**.

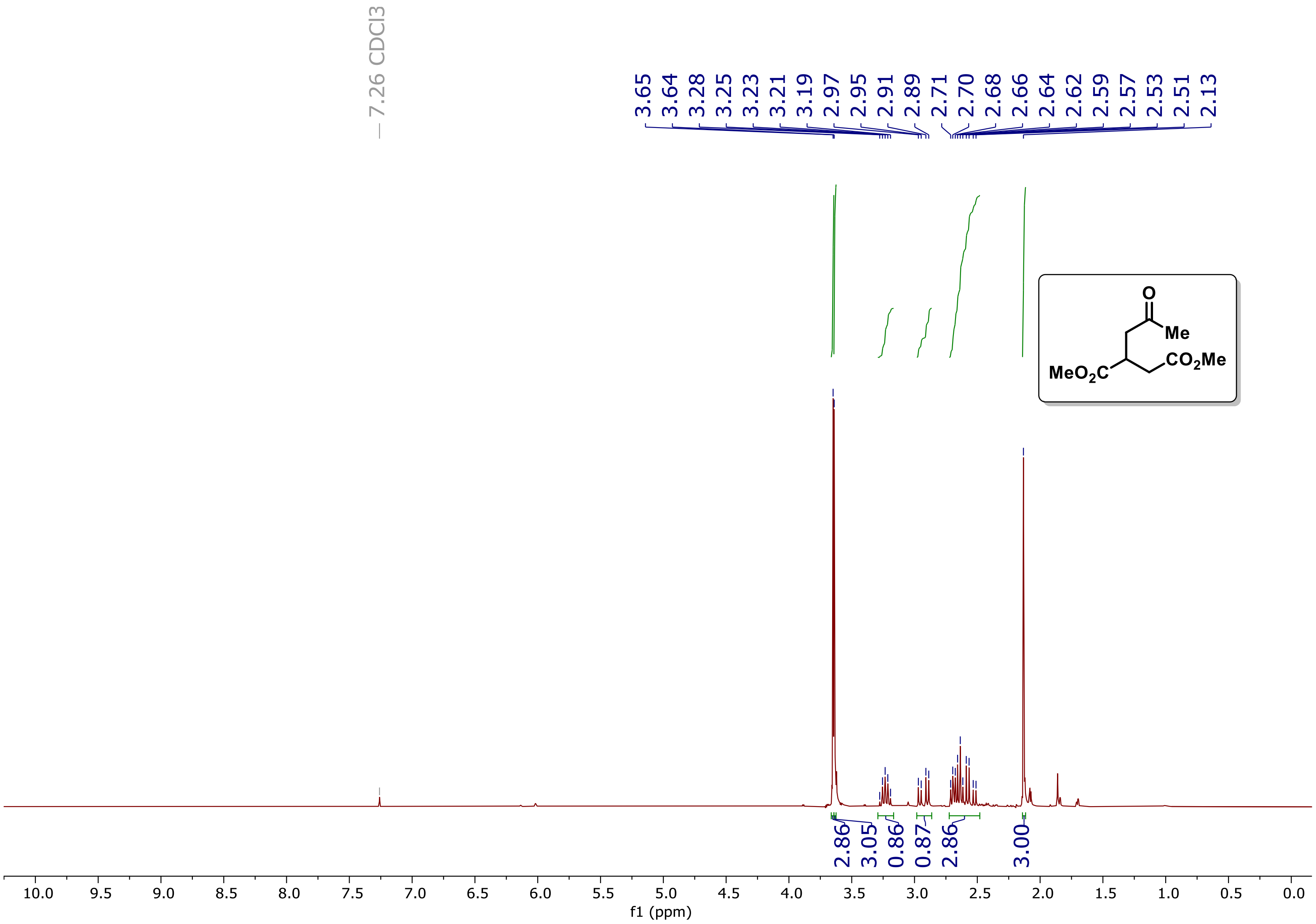

^{13}C {^{1}H} NMR ($CDCl_3$, 75 MHz) of **S12**.

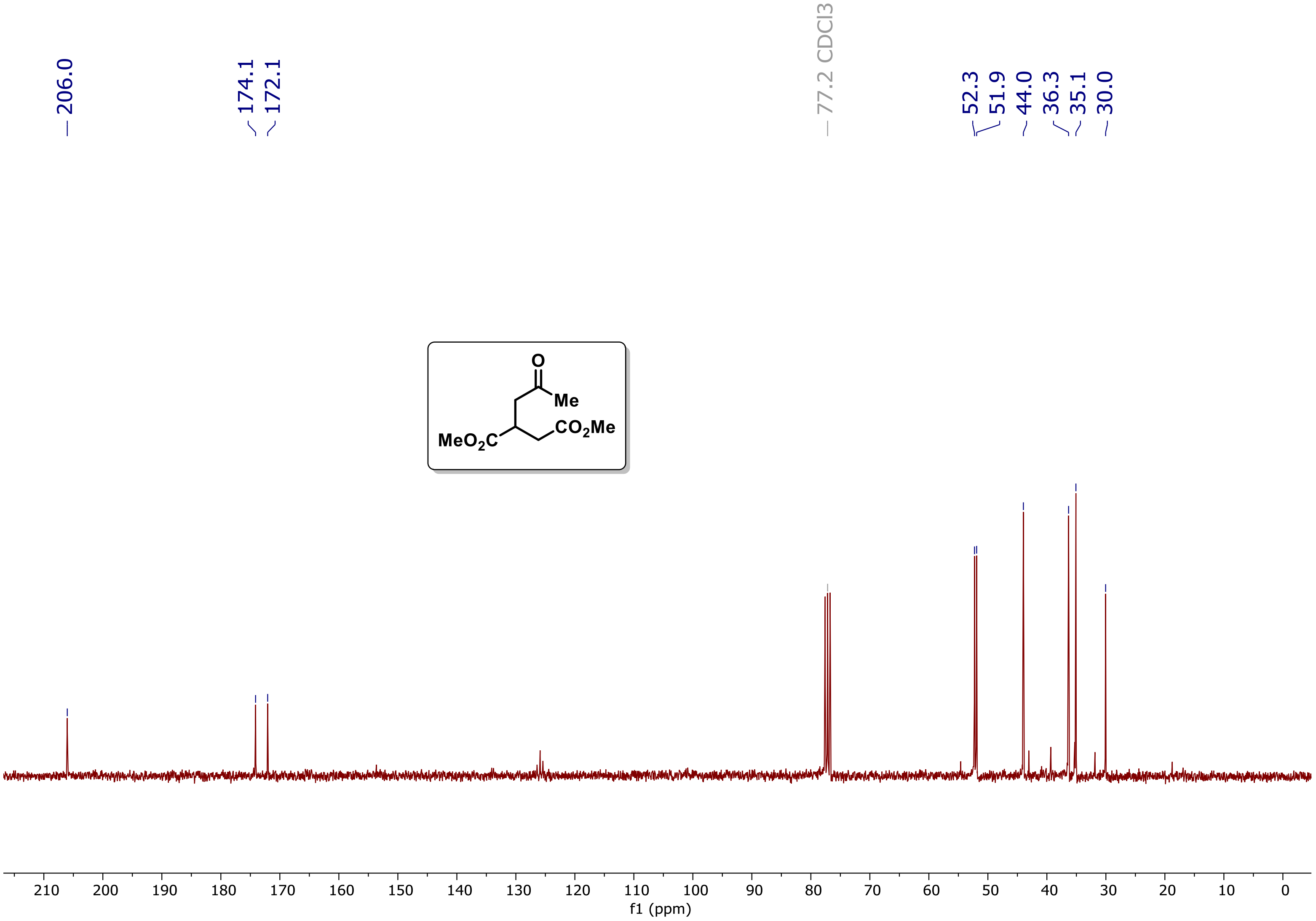

^{1}H NMR ($CDCl_3$, 400 MHz) of **S13**.

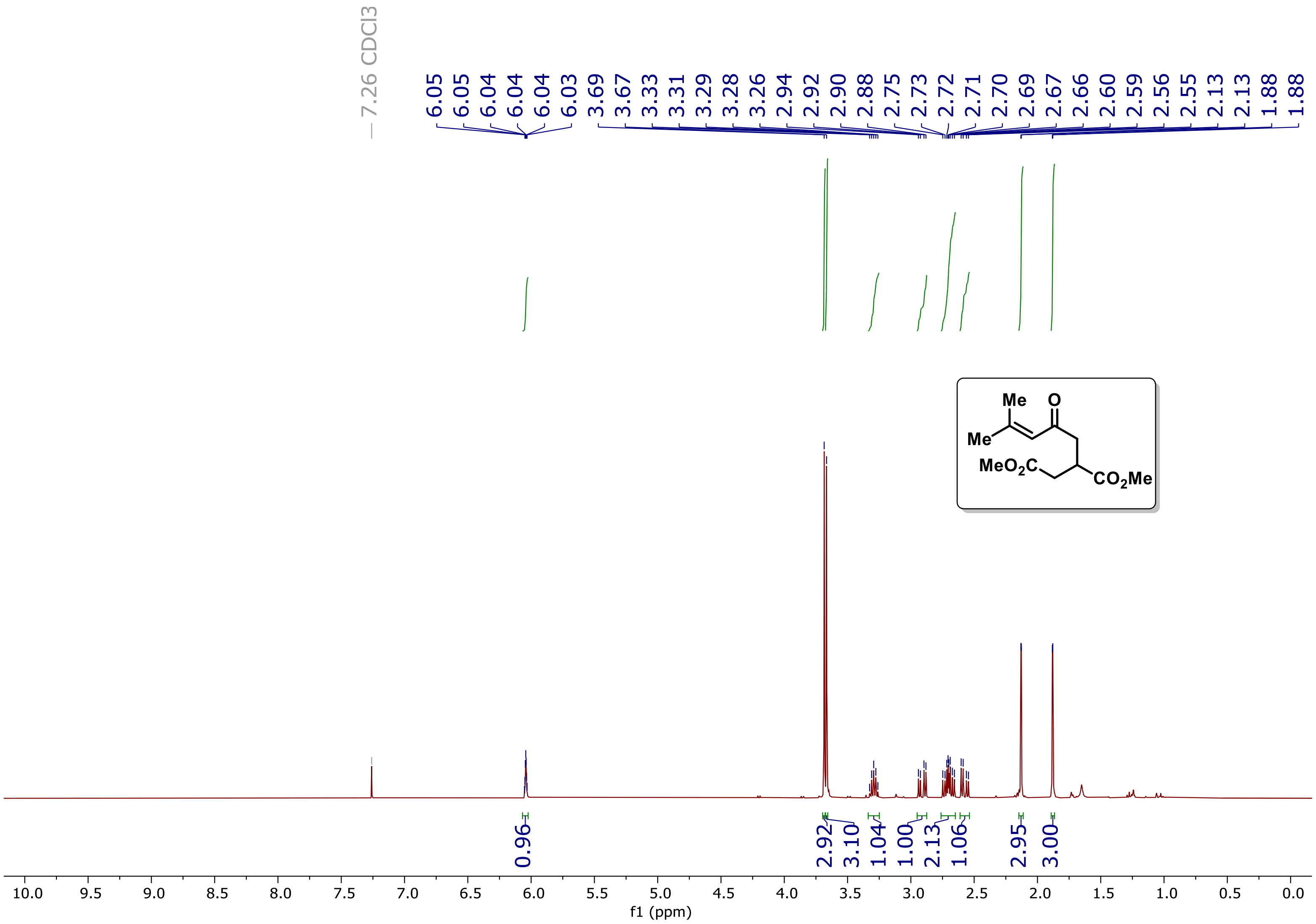

$^{13}C\{^{1}H\}$ NMR ($CDCl_3$, 100 MHz) of **S13**.

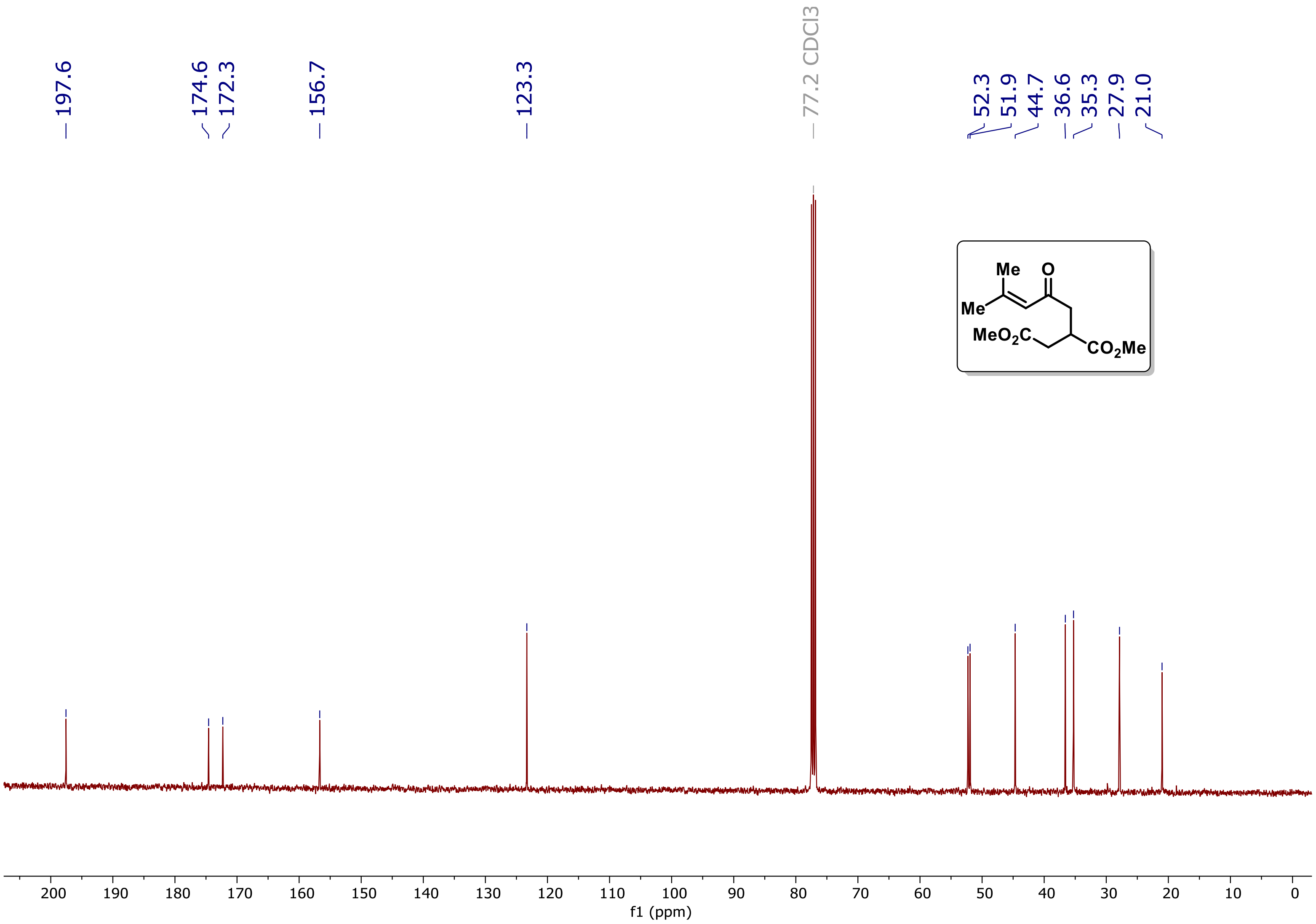

^{1}H NMR ($CDCl_3$, 300 MHz) of **23f**.

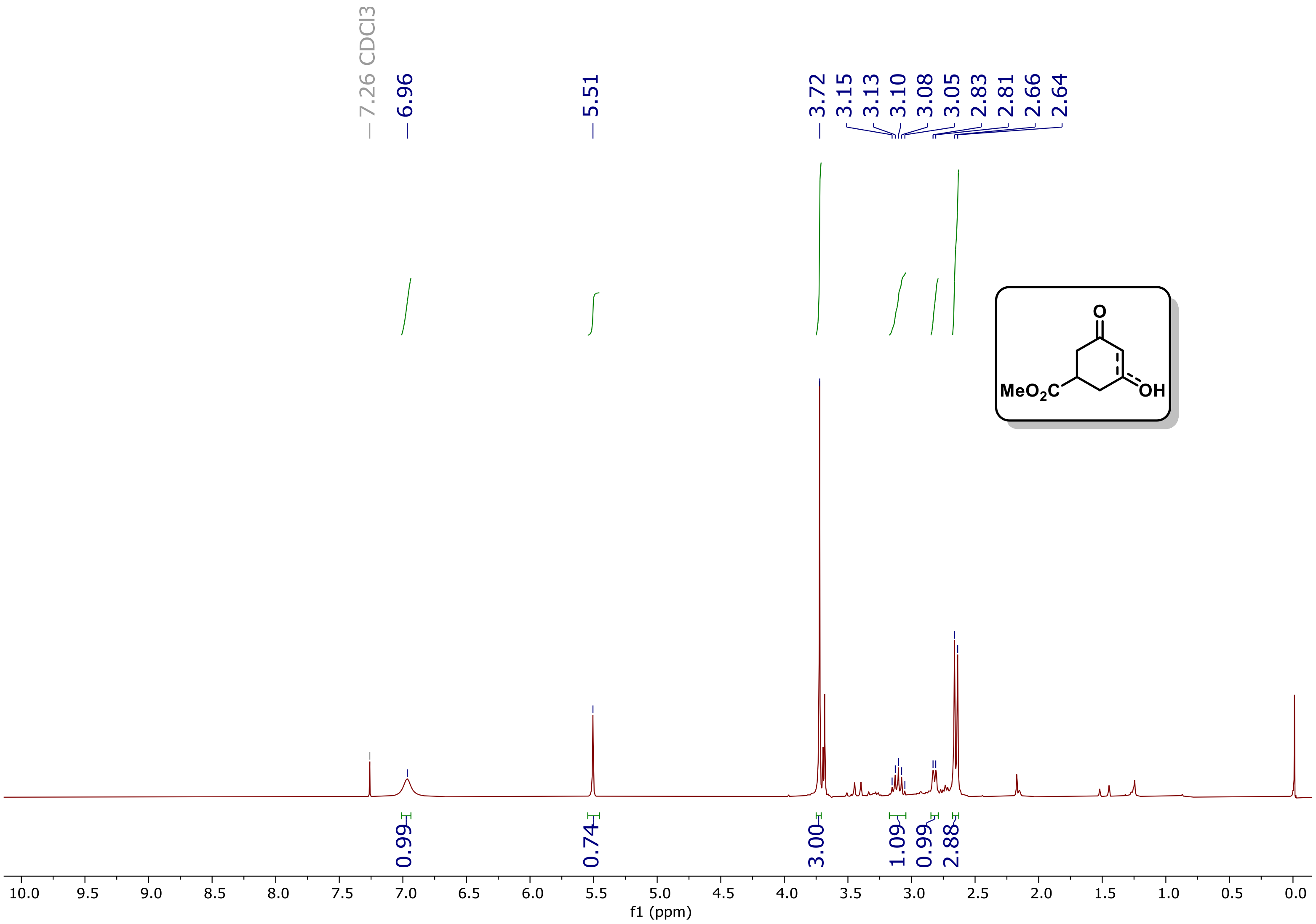

^{13}C {^{1}H} NMR ($CDCl_3$, 75 MHz) of **23f**.

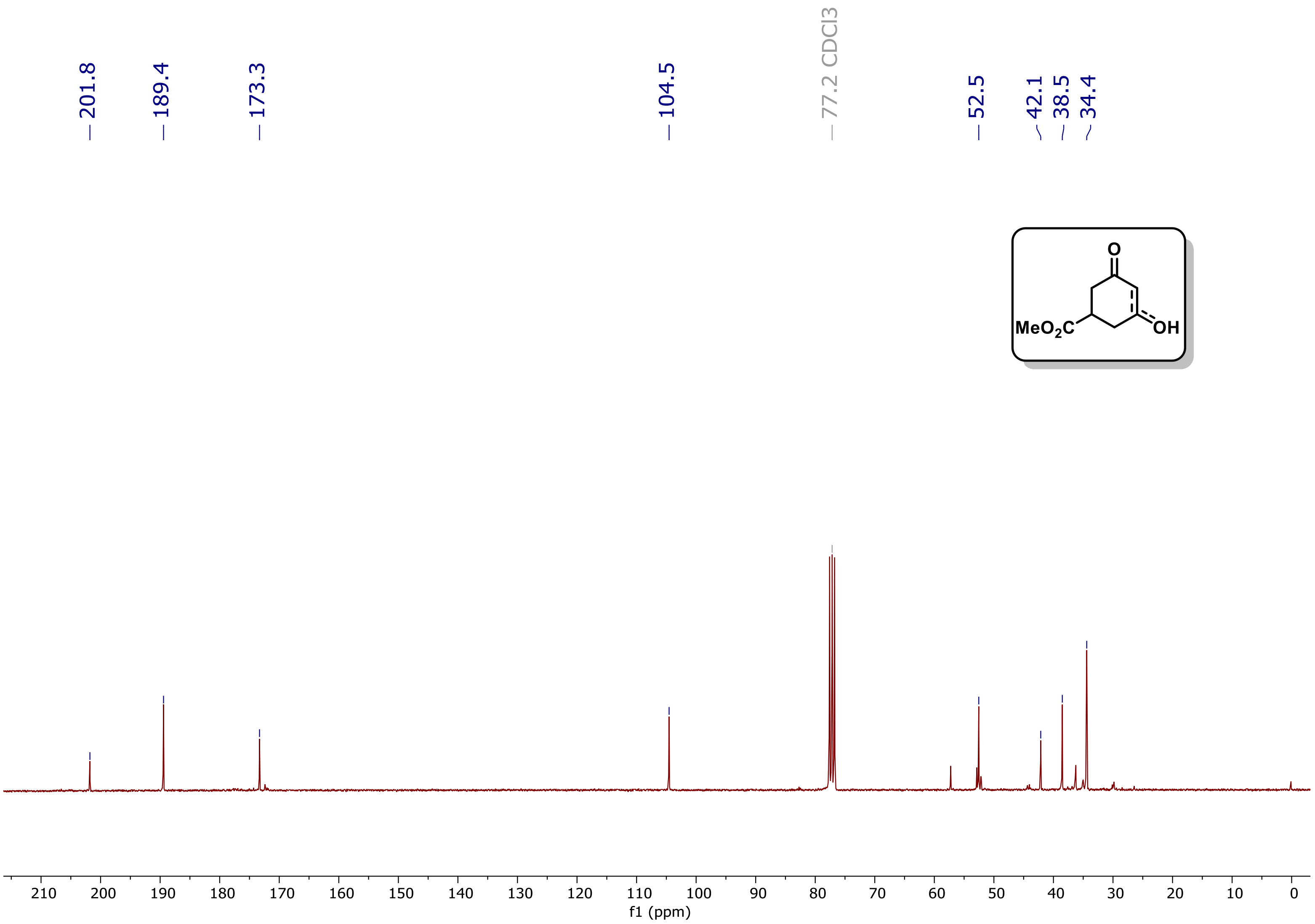

^{1}H NMR Spectra ($CDCl_3$, 300 MHz) of **24a**.

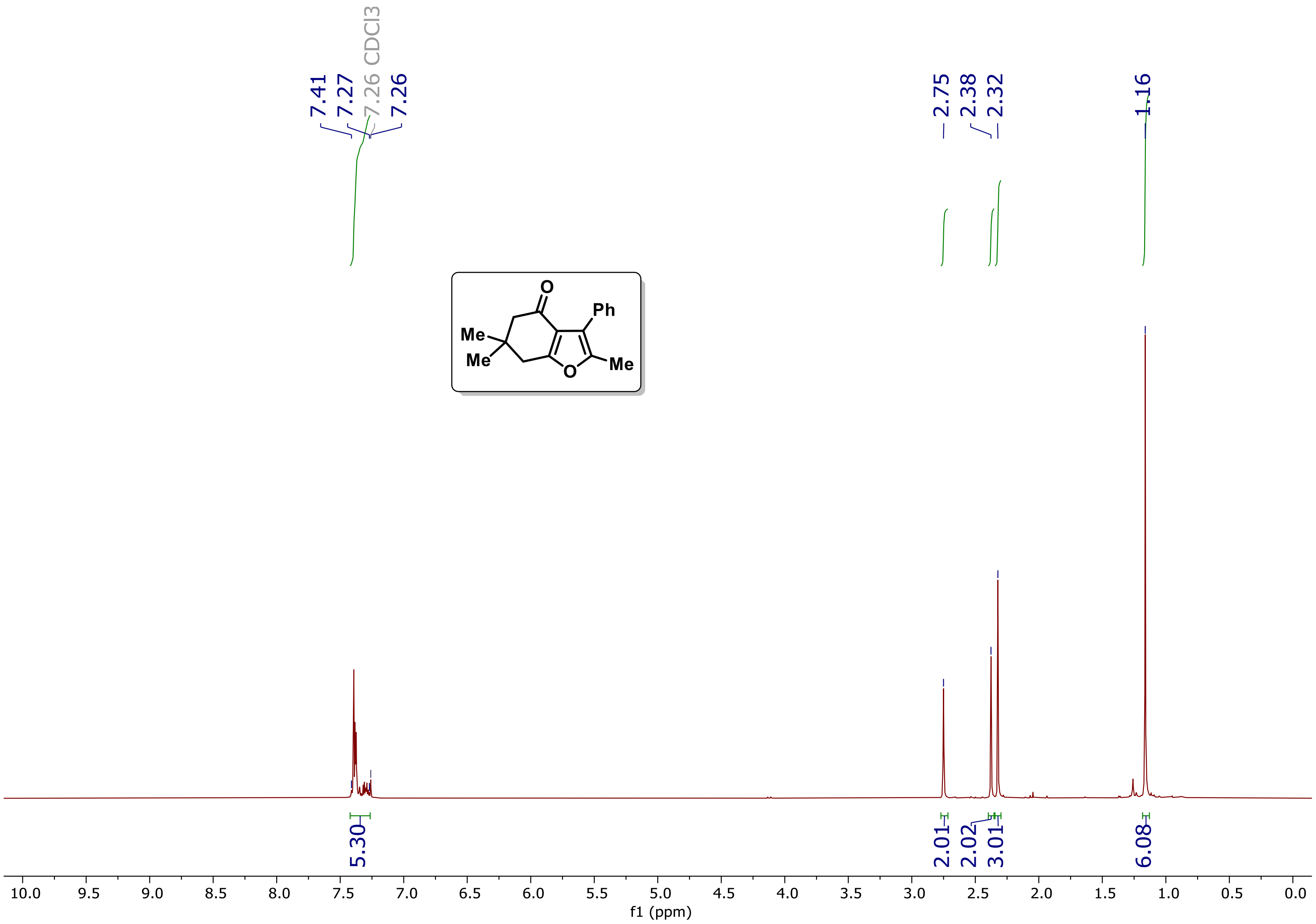

$^{13}C\{^{1}H\}$ NMR ($CDCl_3$, 75 MHz) of **24a**.

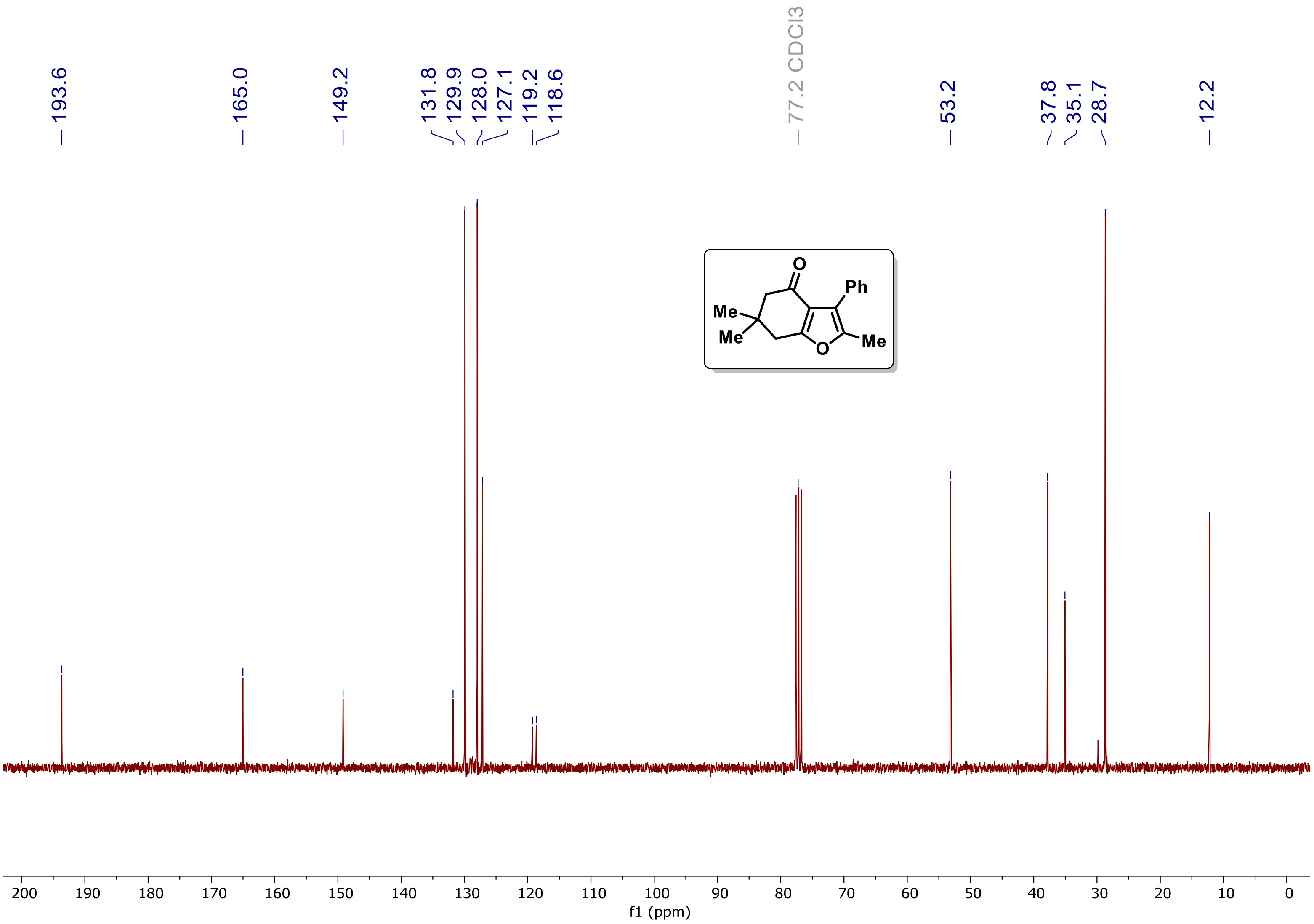

^{1}H NMR Spectra ($CDCl_3$, 300 MHz) of **24b**.

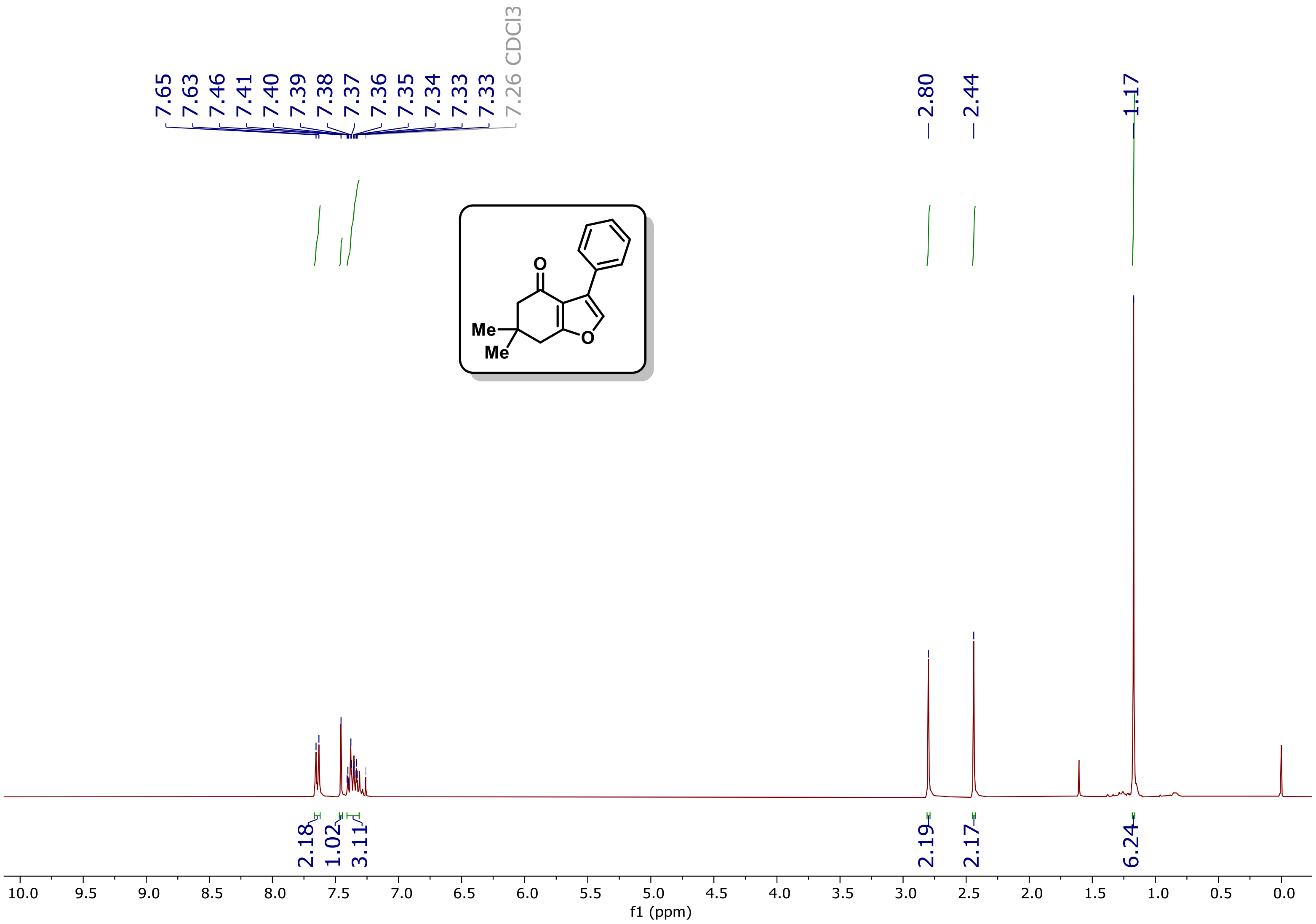

^{1}H NMR ($CDCl_3$, 300 MHz) of **24c**.

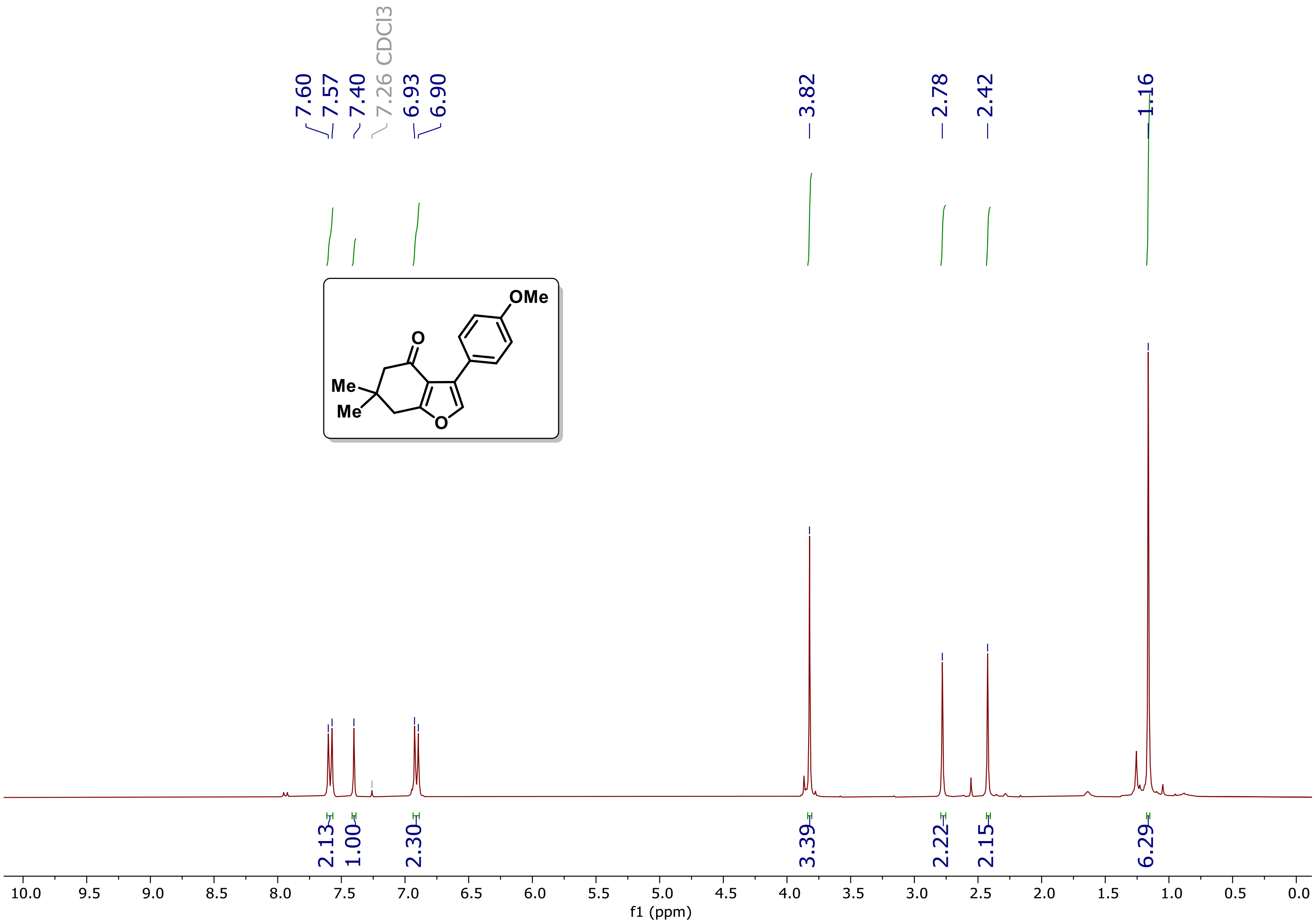

$^{13}C\{^{1}H\}$ NMR ($CDCl_3$, 75 MHz) of **24c**.

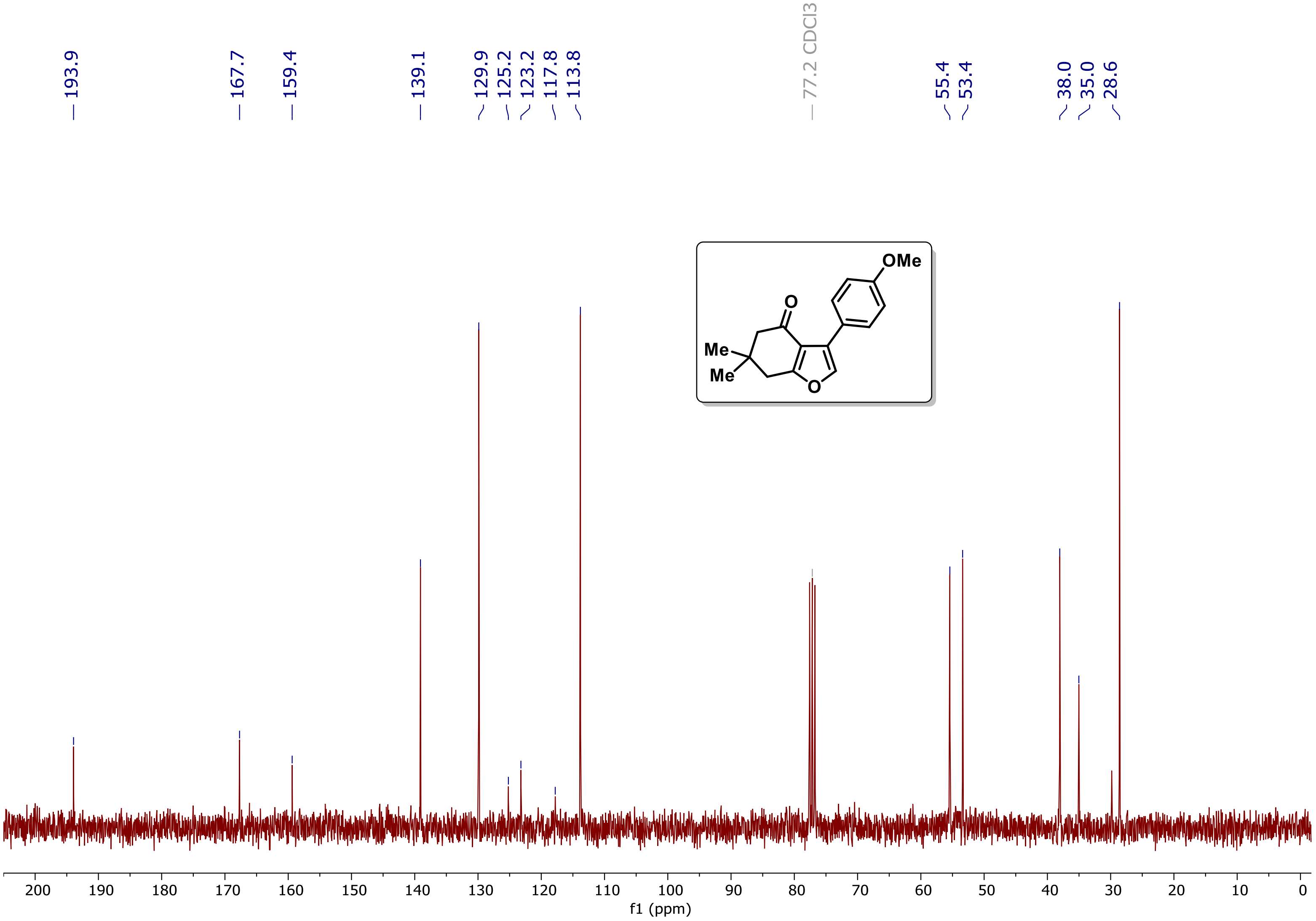

^{1}H NMR ($CDCl_3$, 300 MHz) of **24d**.

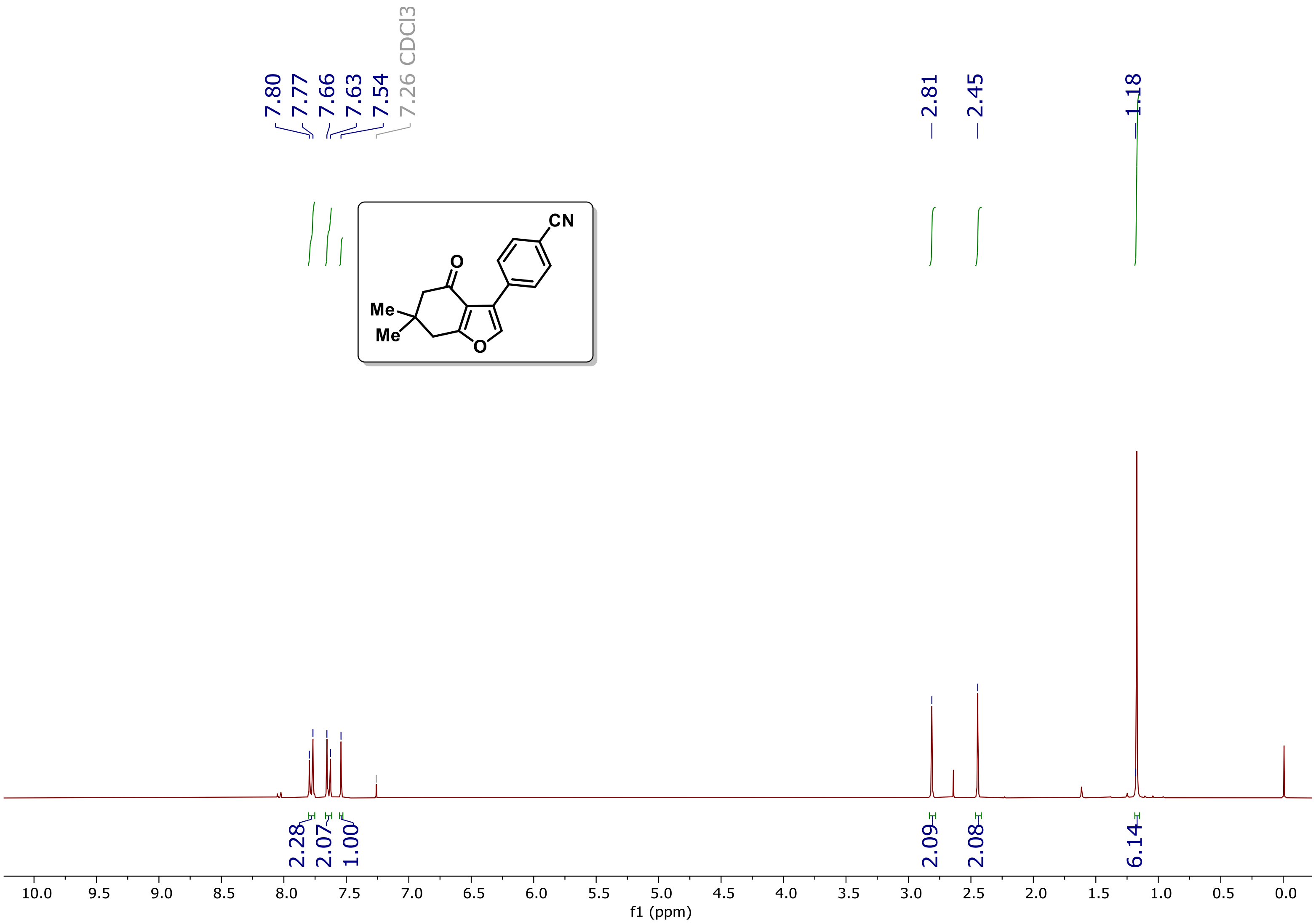

$^{13}C\{^{1}H\}$ NMR ($CDCl_3$, 75 MHz) of **24d**.

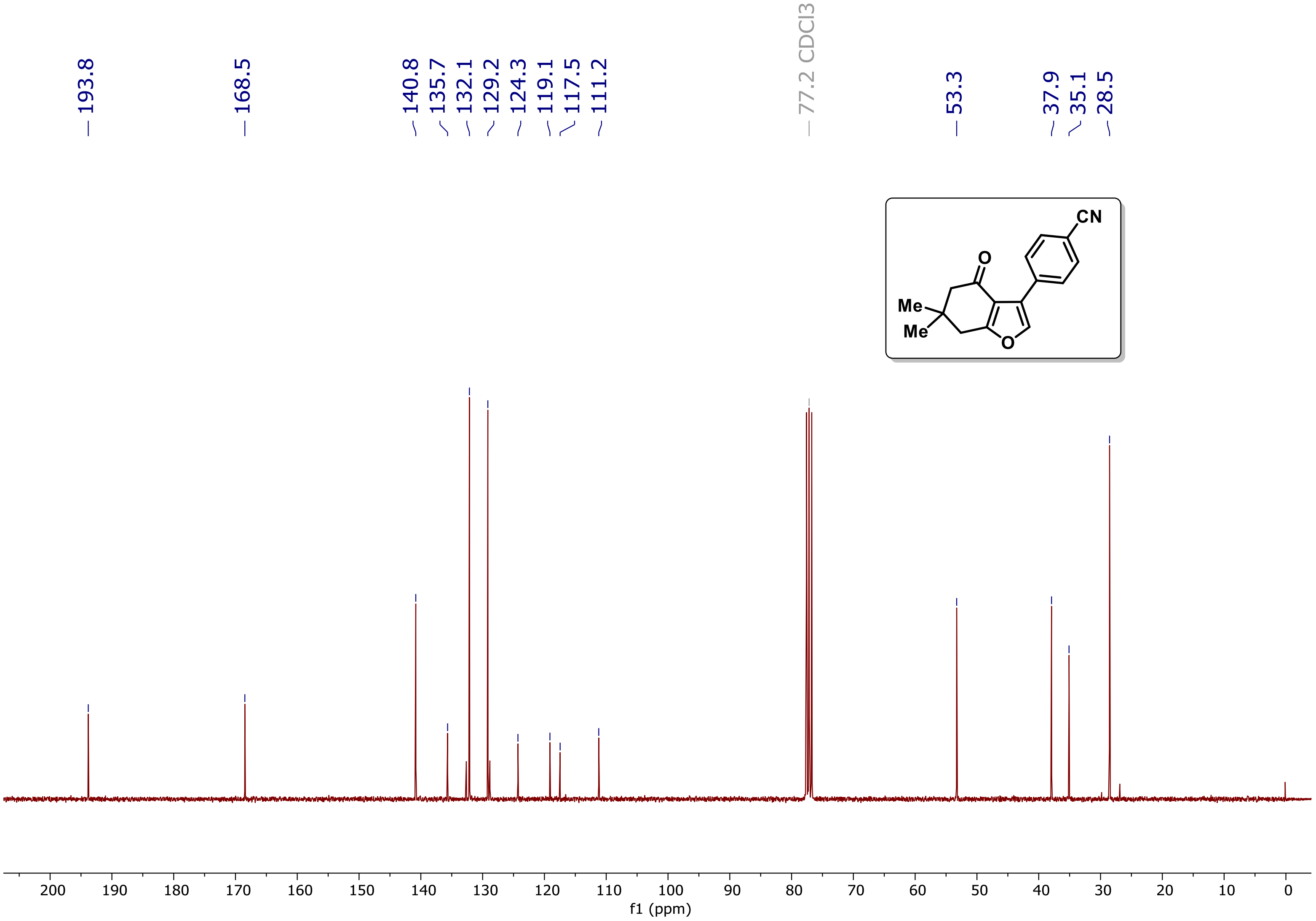

^{1}H NMR ($CDCl_3$, 300 MHz) of **24e**.

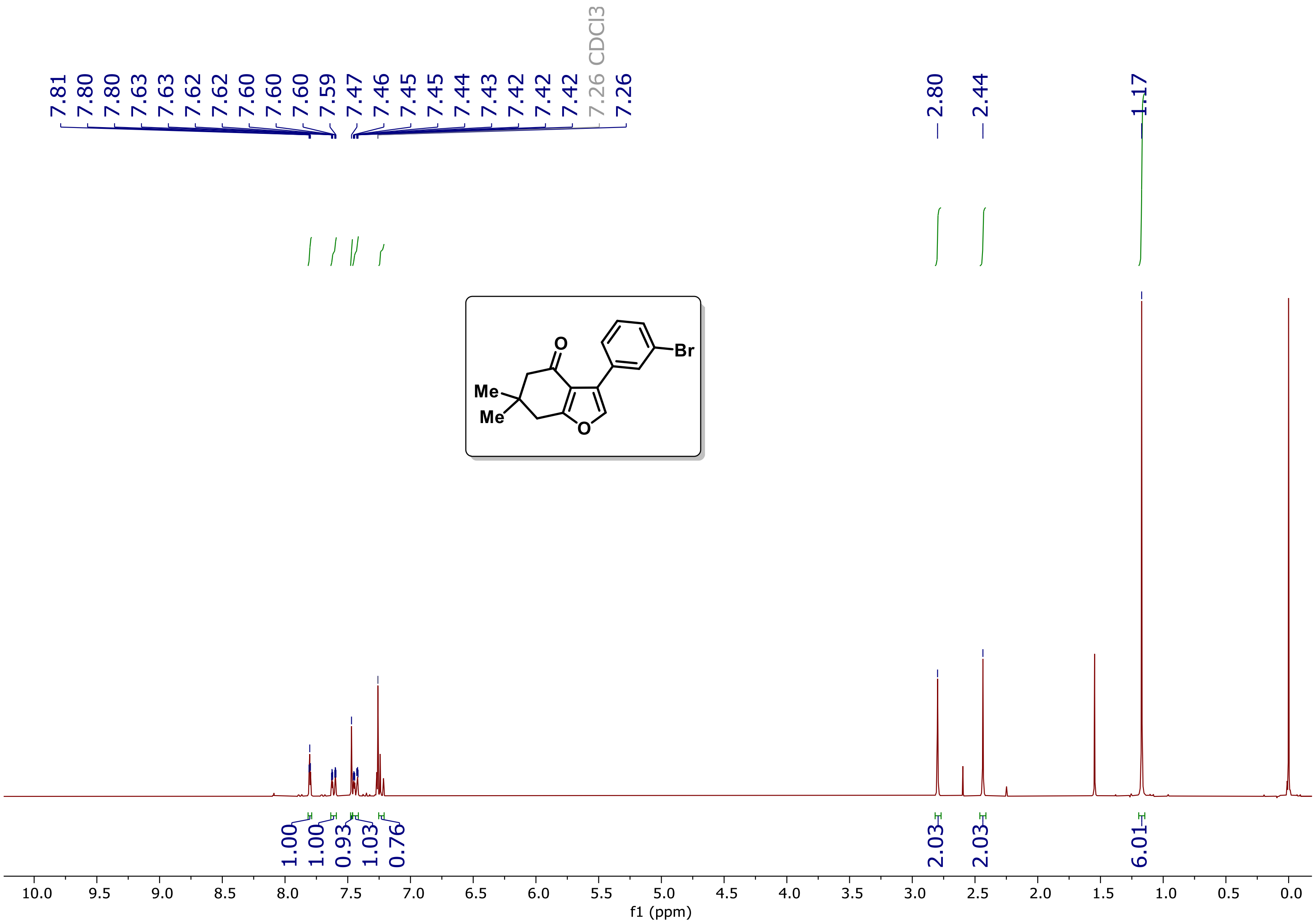

$^{13}C\{^{1}H\}$ NMR ($CDCl_3$, 75 MHz) of **24e**.

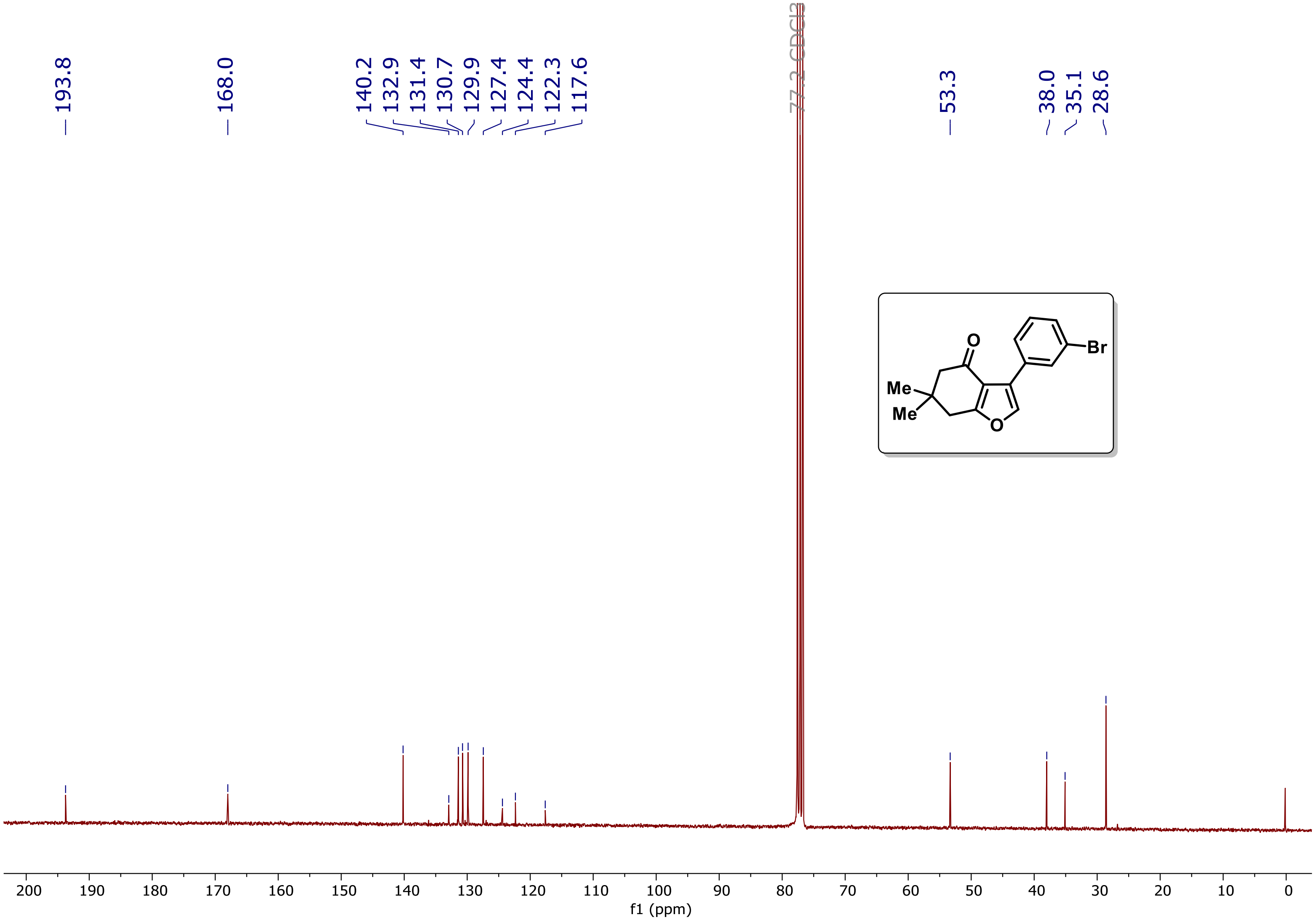

^{1}H NMR ($CDCl_3$, 300 MHz) of **24f**.

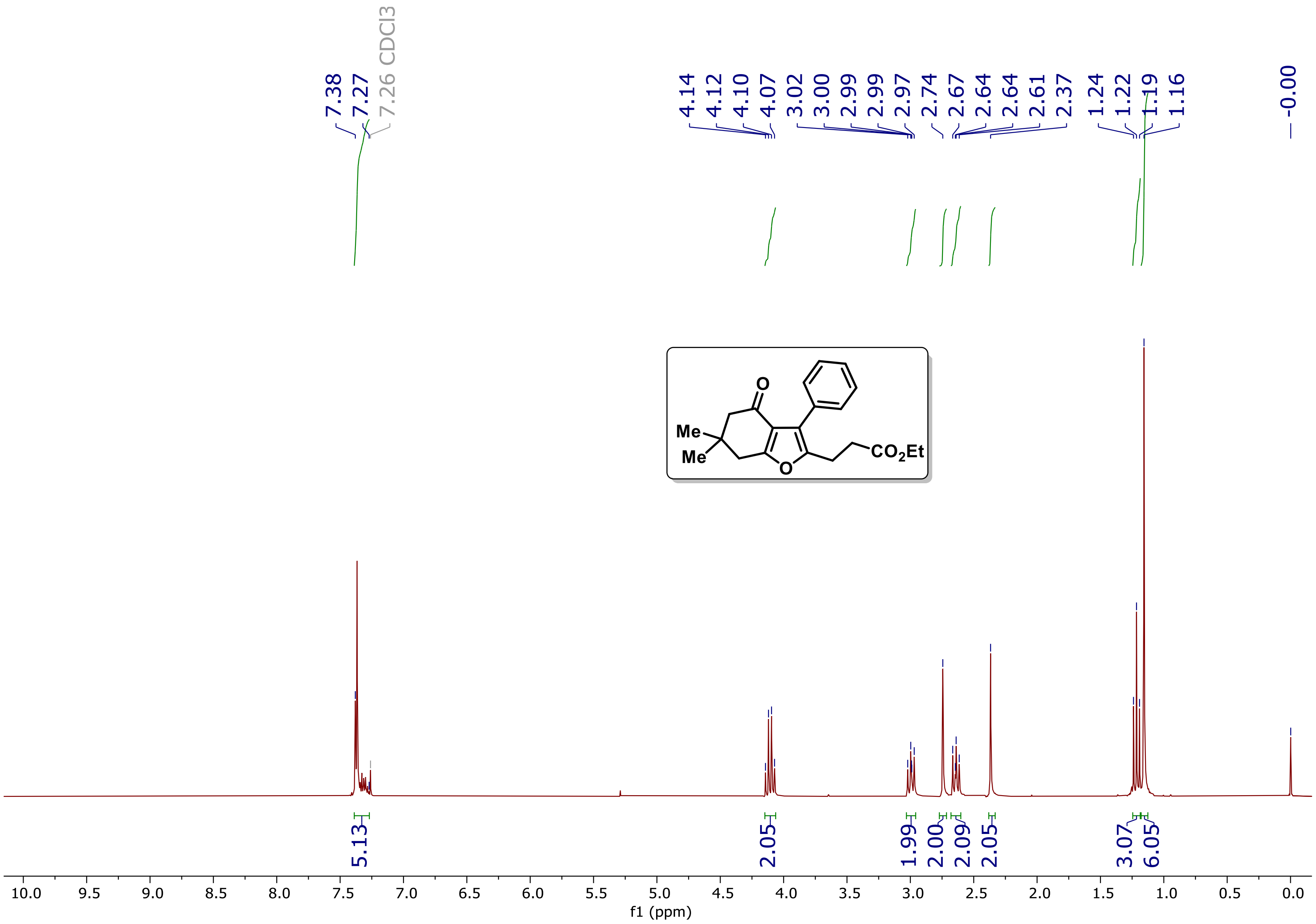

$^{13}C\{^1H\}$ NMR ($CDCl_3$, 75 MHz) of **24f**.

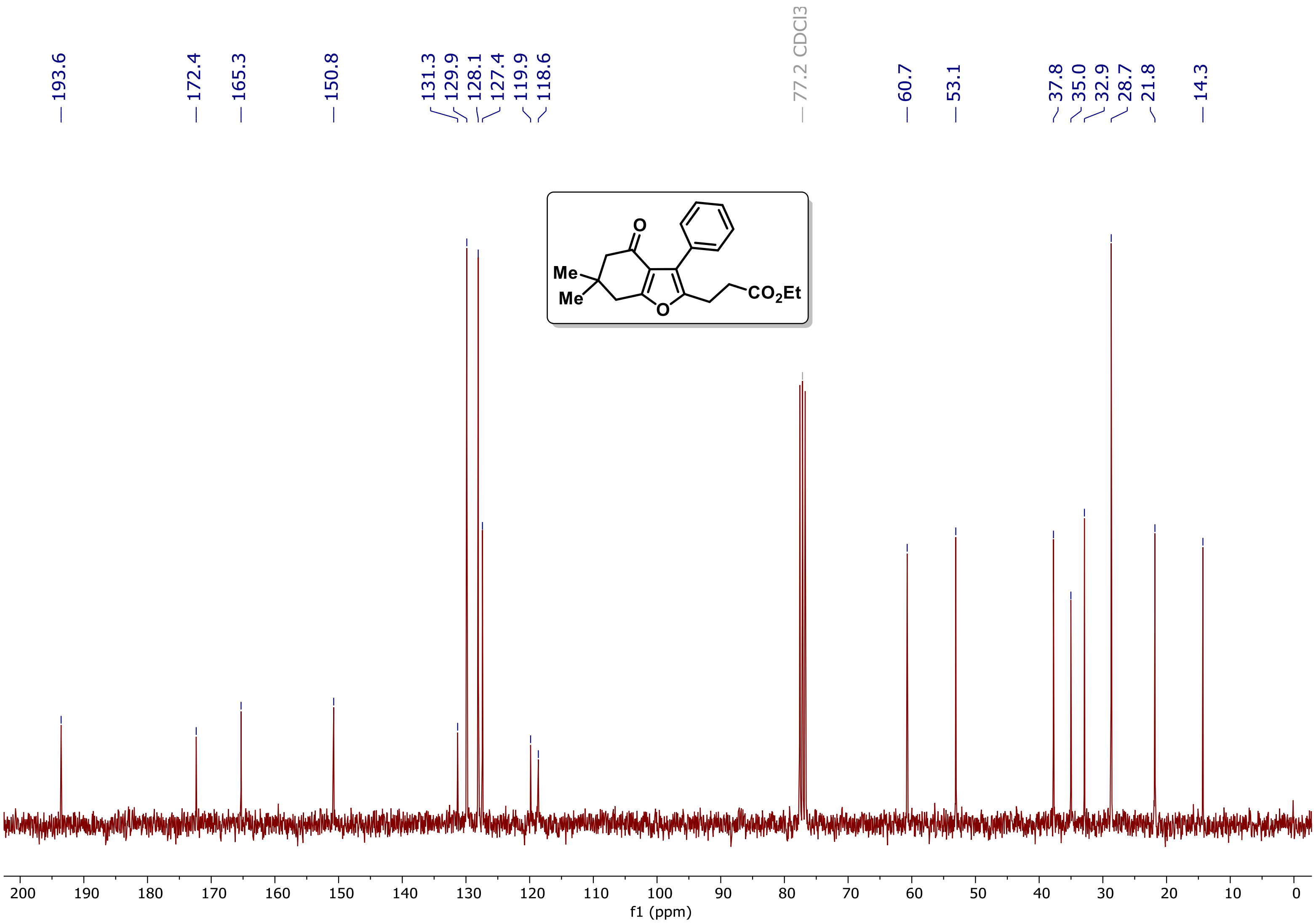

^{1}H NMR ($CDCl_3$, 300 MHz) of **24g**.

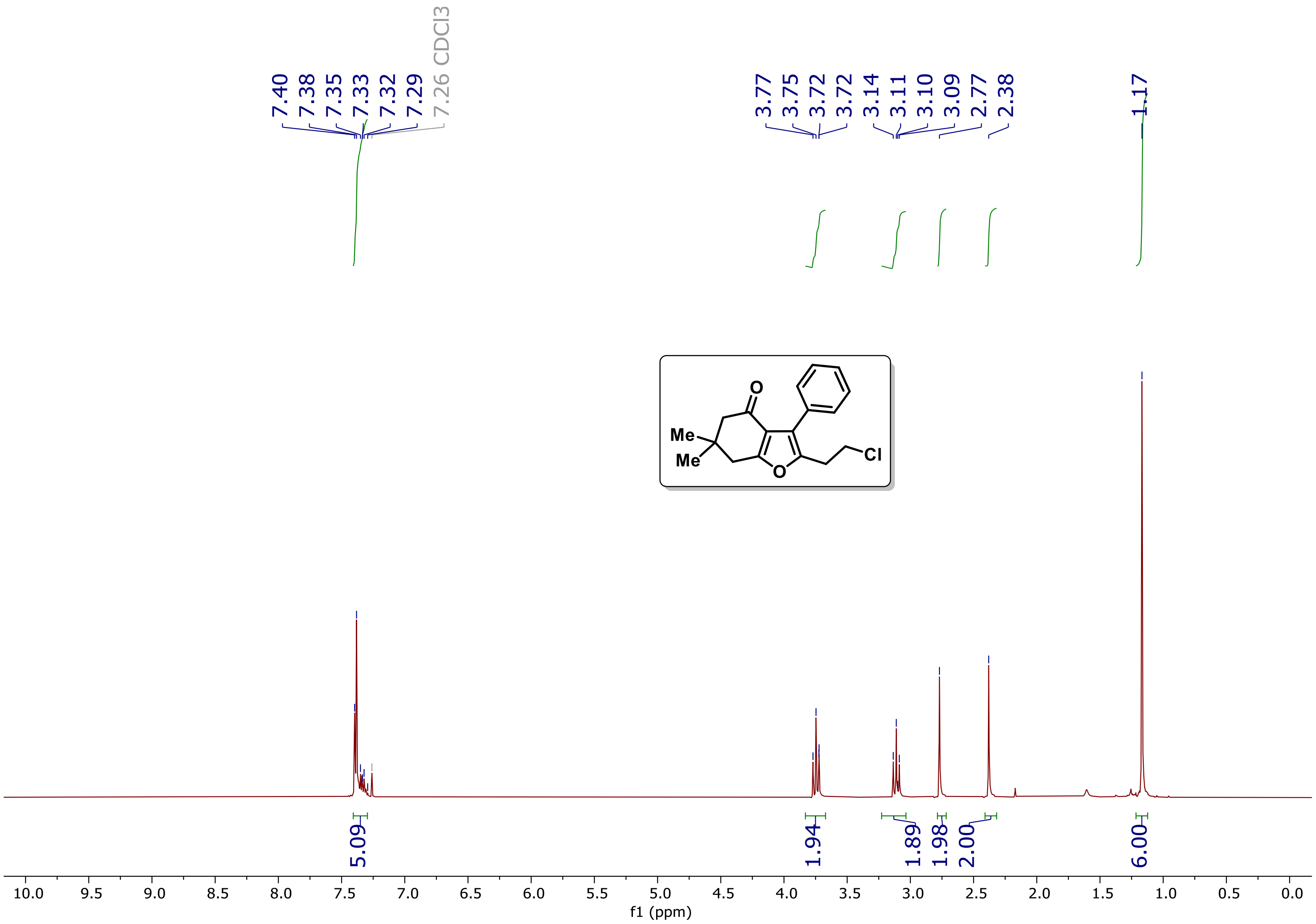

$^{13}C\{^{1}H\}$ NMR ($CDCl_3$, 75 MHz) of **24g**.

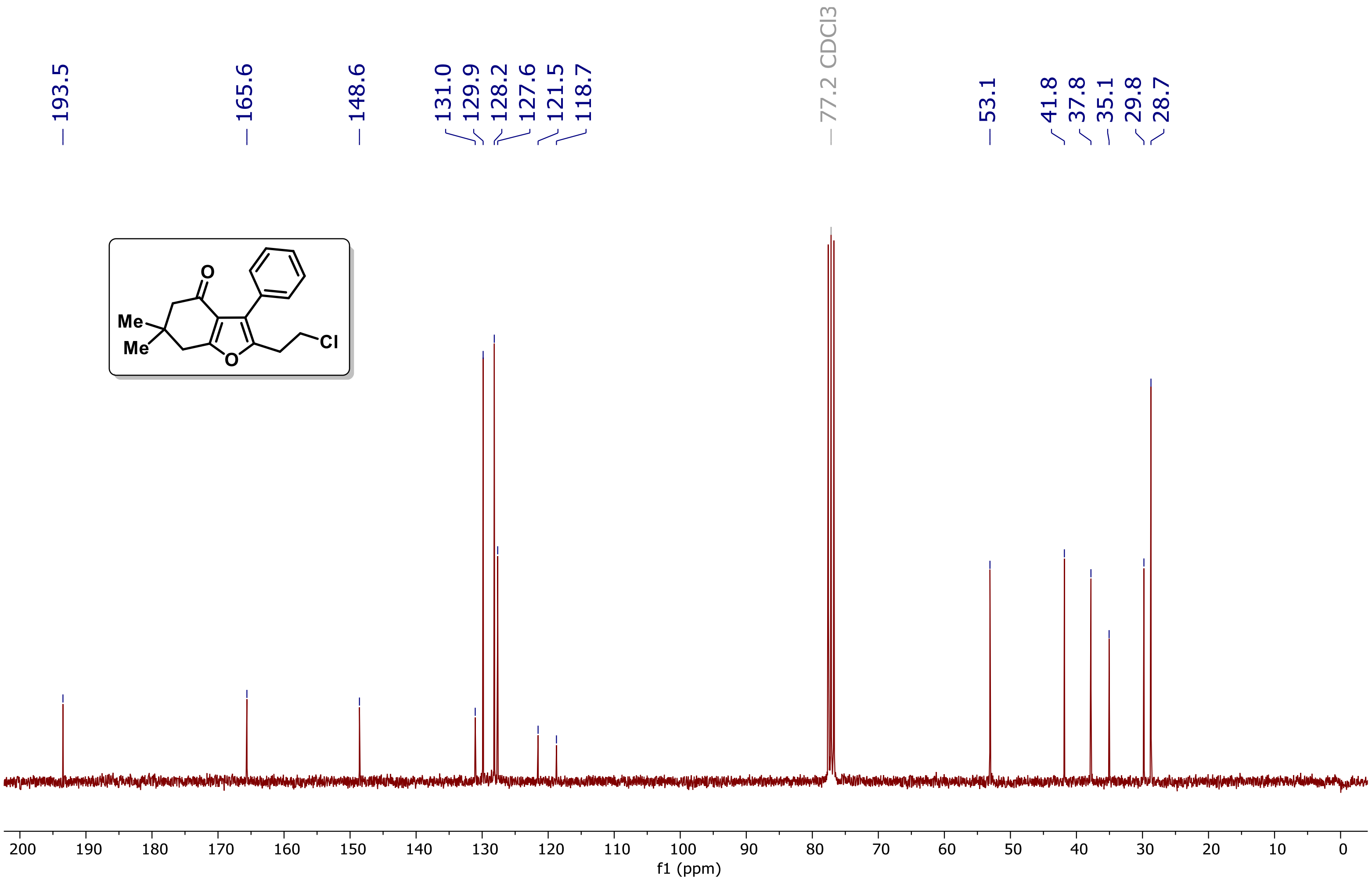

^{1}H NMR ($CDCl_3$, 300 MHz) of **24h**.

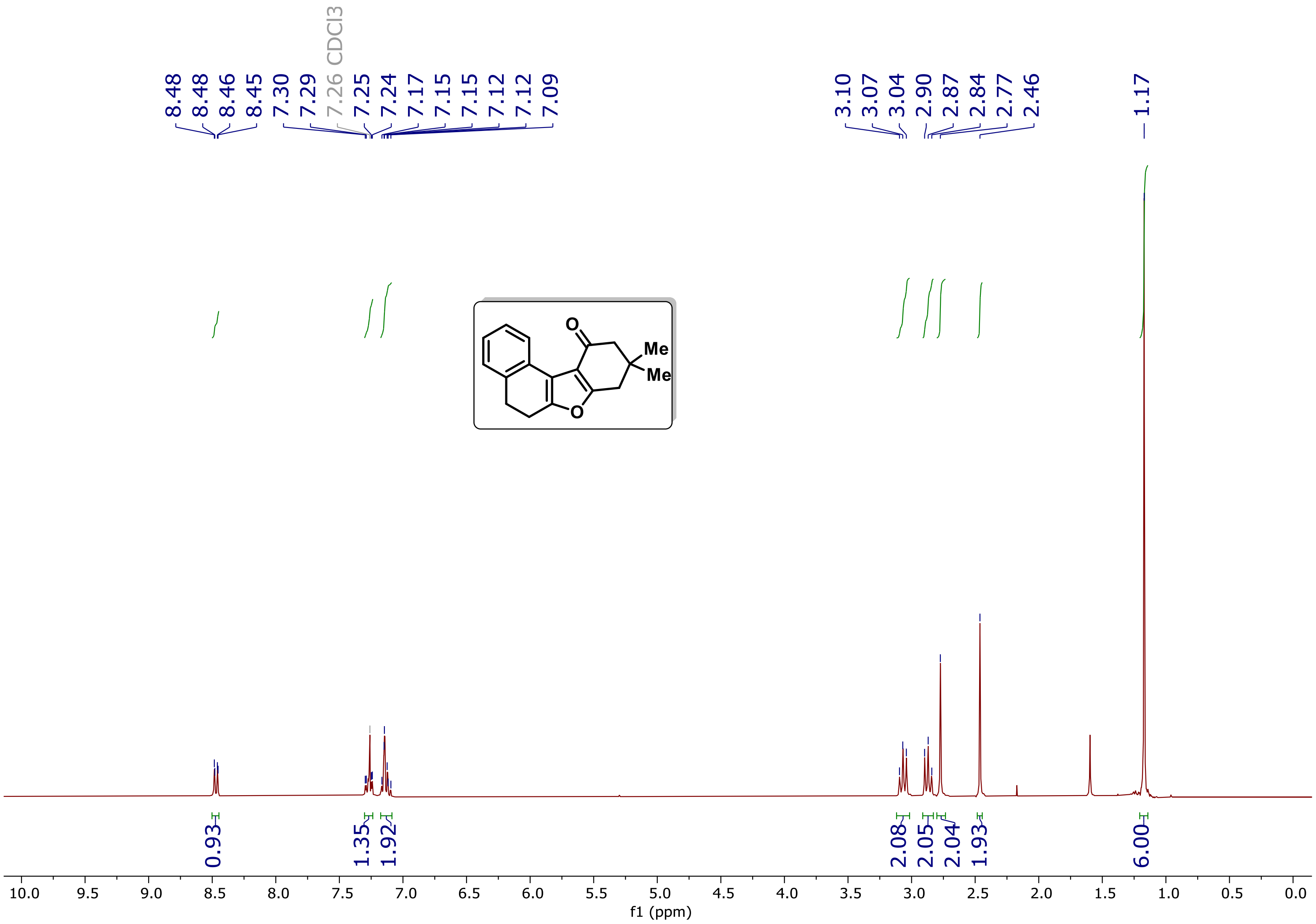

$^{13}C\{^{1}H\}$ NMR ($CDCl_3$, 75 MHz) of **24h**.

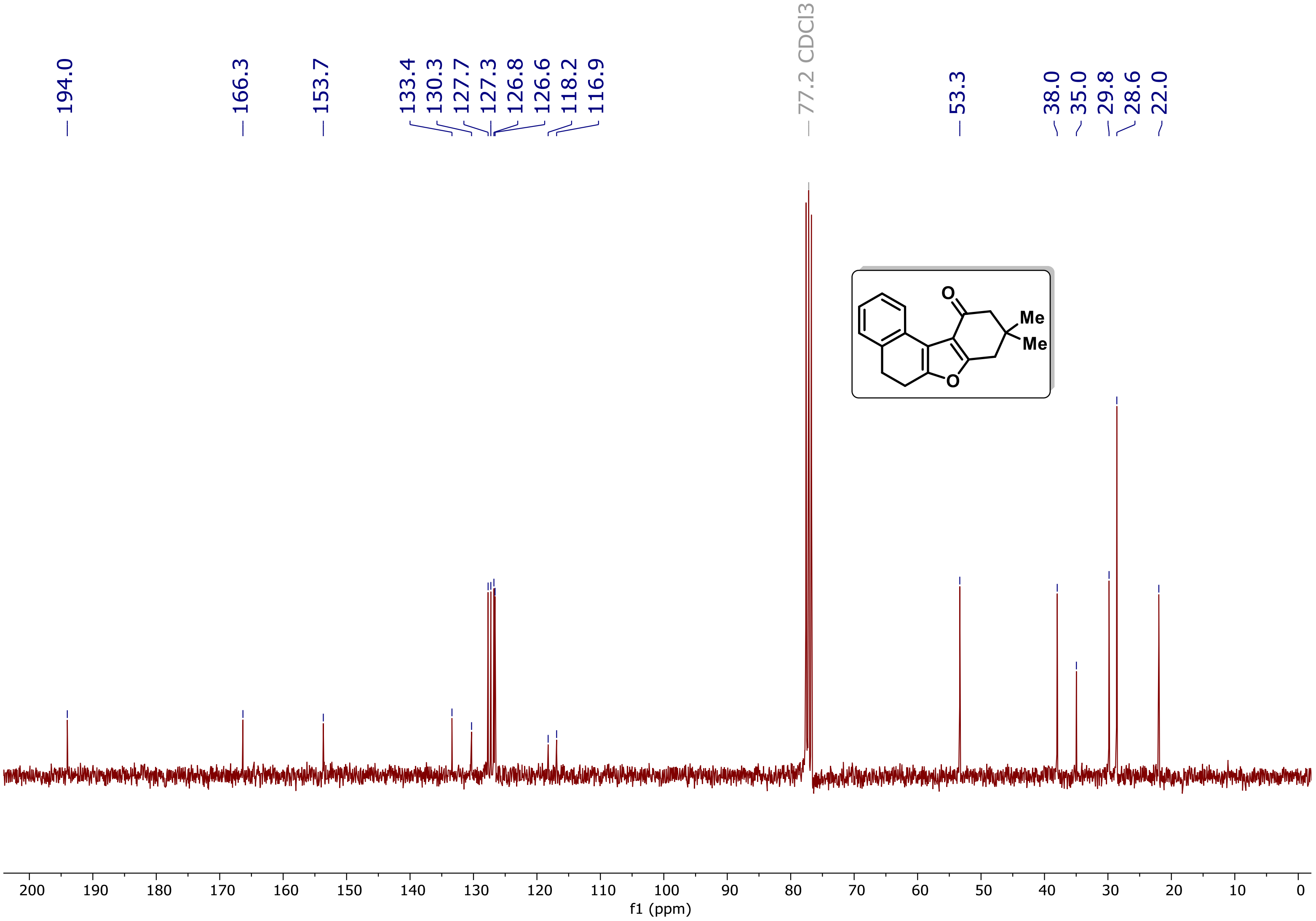

^{1}H NMR ($CDCl_3$, 300 MHz) of **24i**.

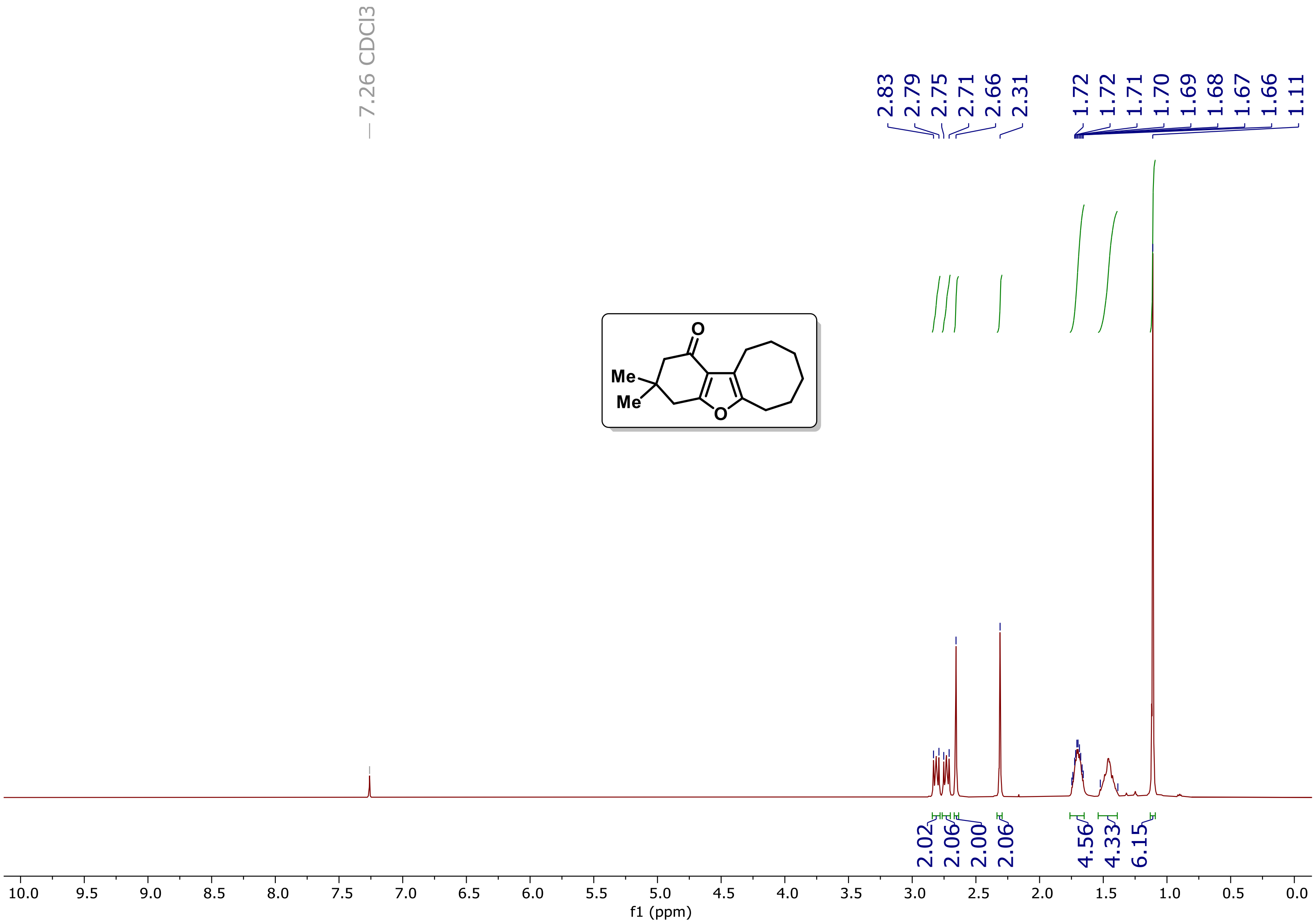

$^{13}C\{^{1}H\}$ NMR ($CDCl_3$, 75 MHz) of **24i**.

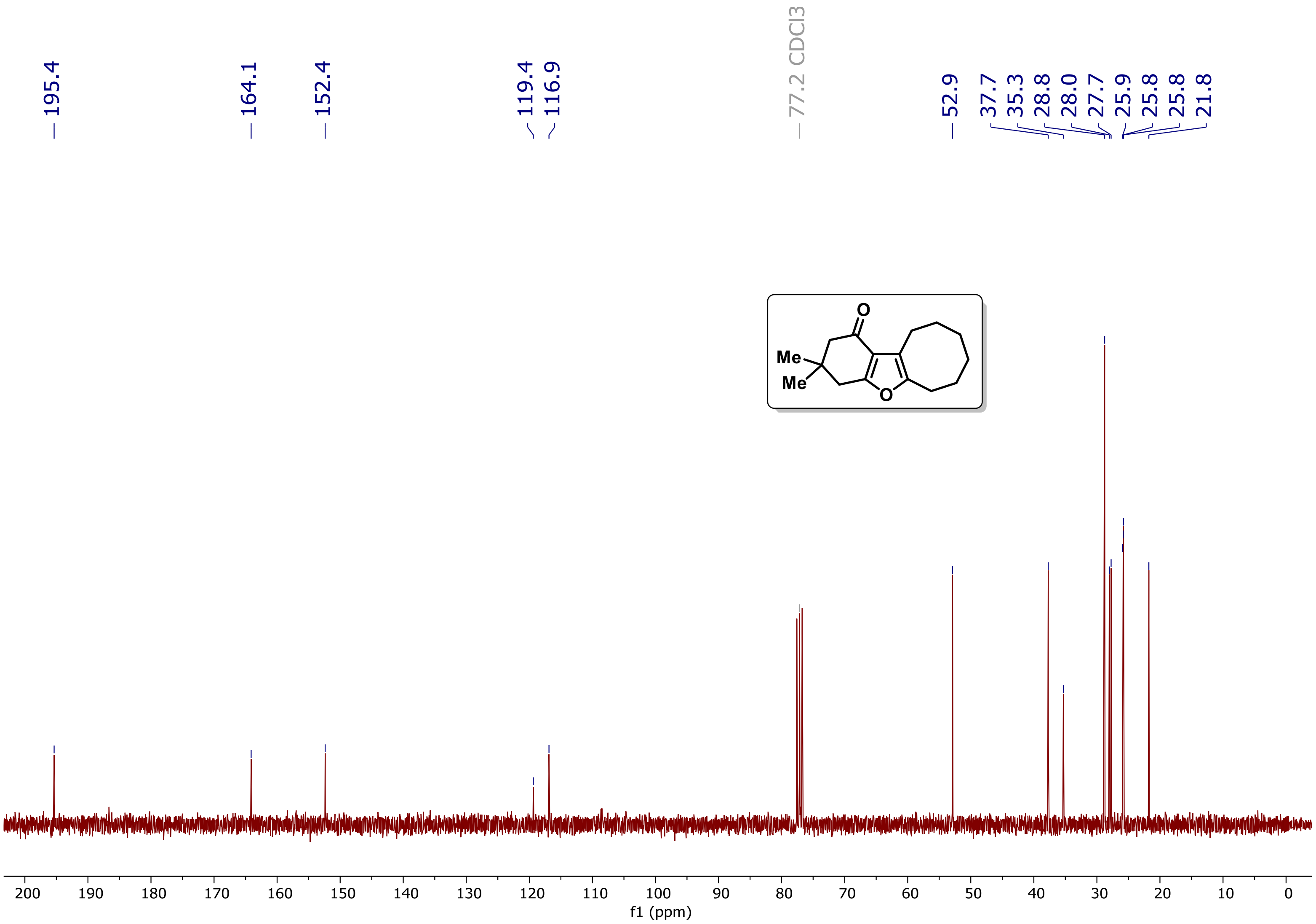

^{1}H NMR ($CDCl_3$, 300 MHz) of **24j**.

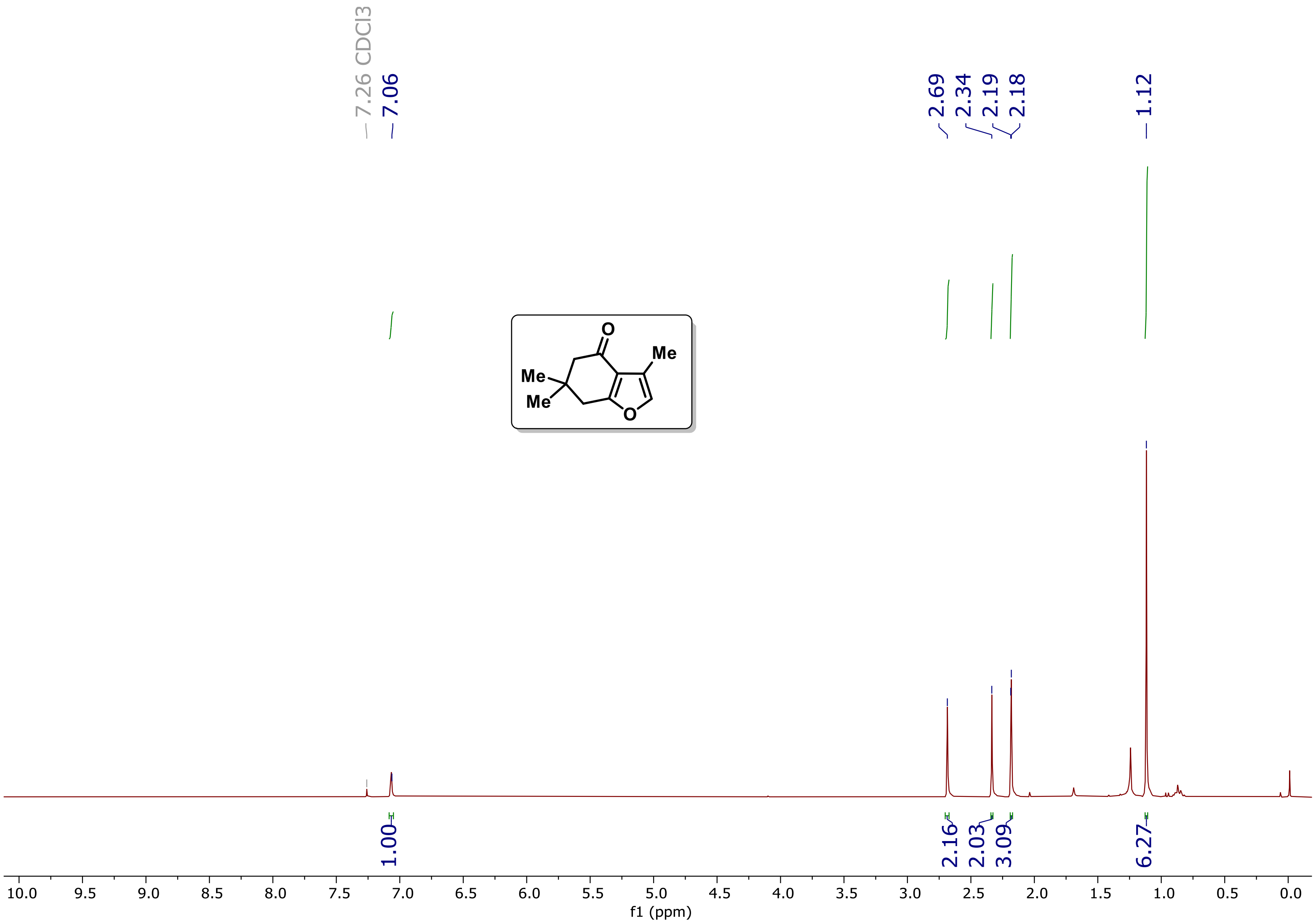

^1H NMR ($CDCl_3$, 400 MHz) of **24k**.

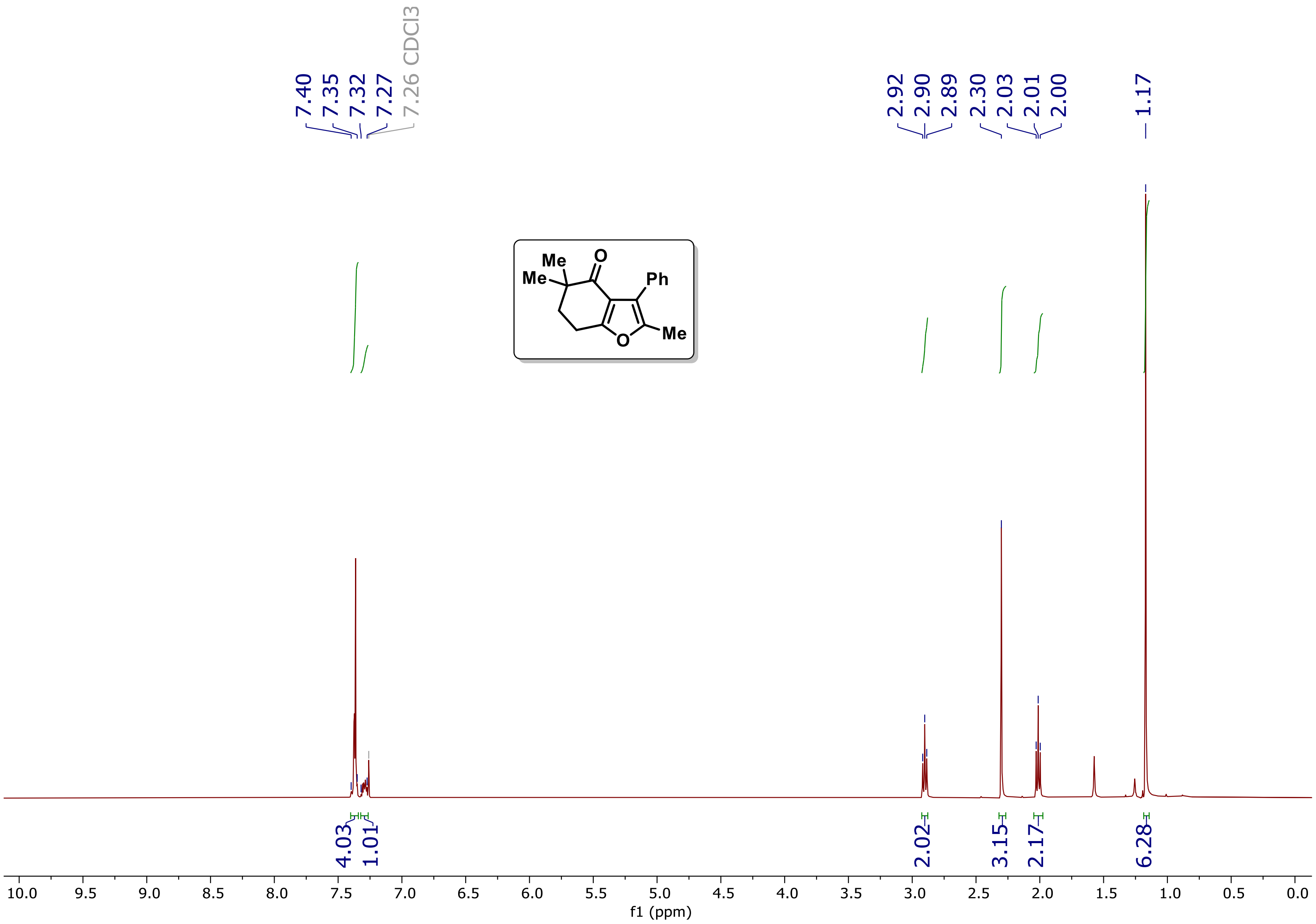

$^{13}C\{^{1}H\}$ NMR ($CDCl_3$, 100 MHz) of **24k**.

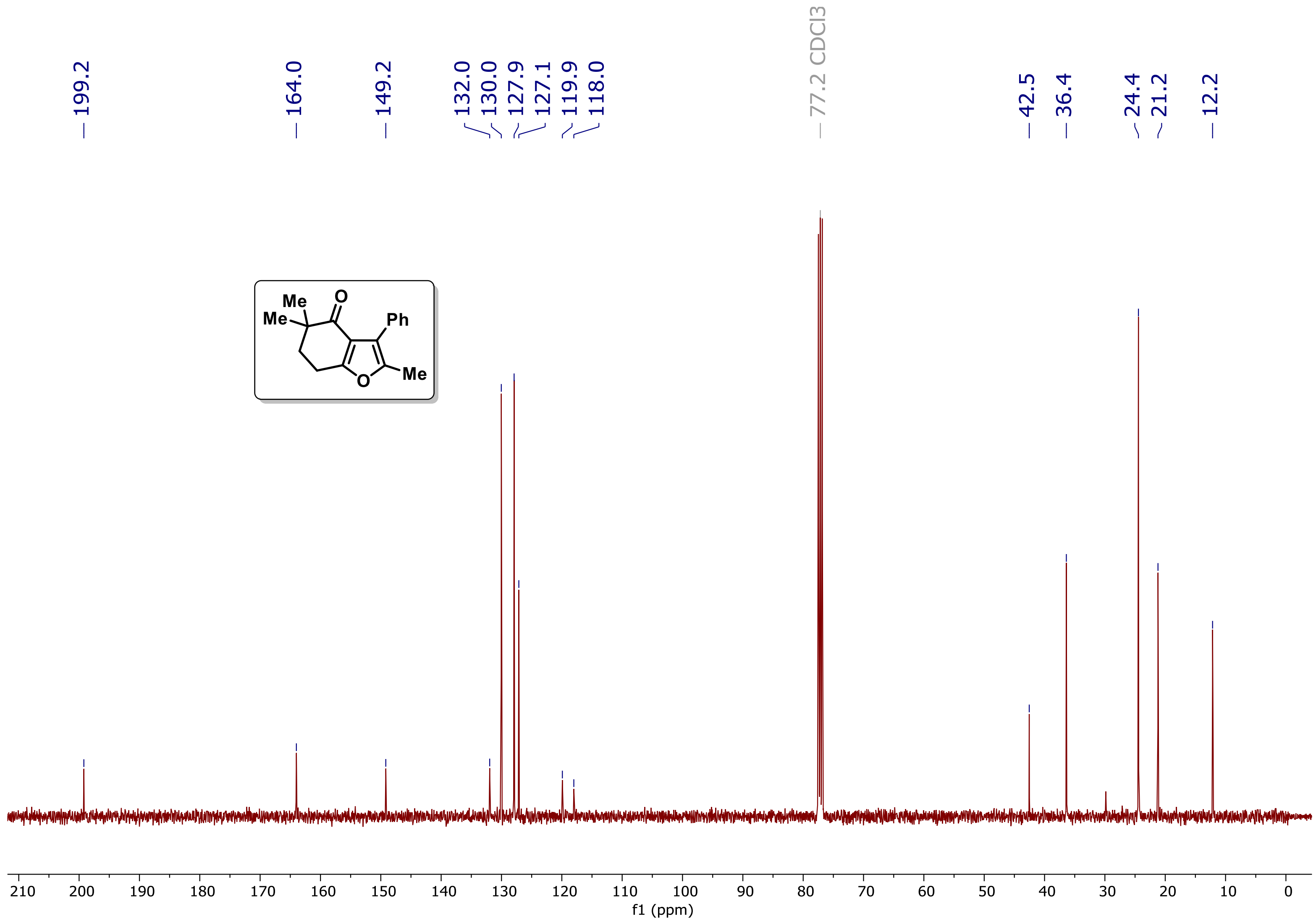

^{1}H NMR ($CDCl_3$, 400 MHz) of **24k´**.

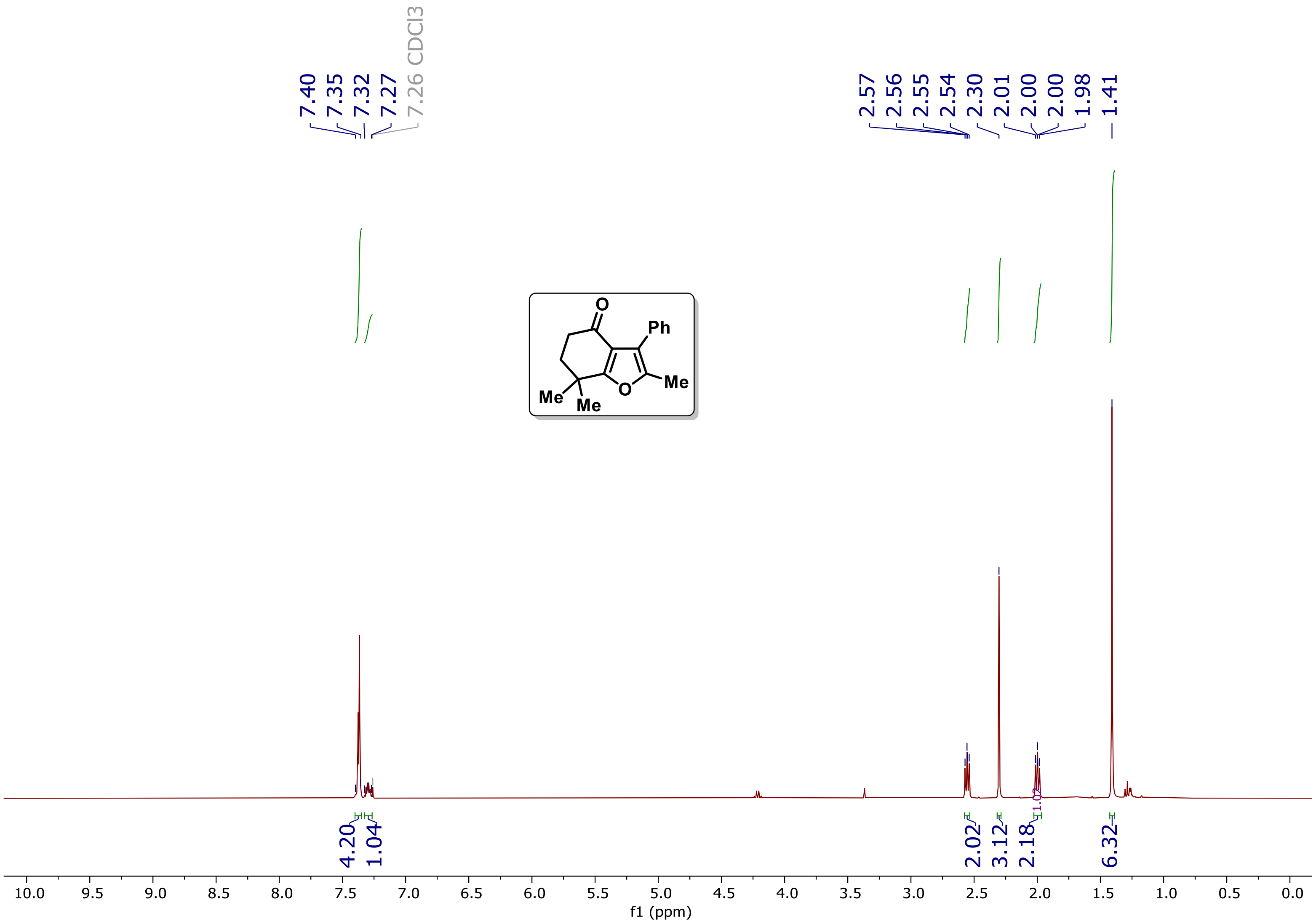

$^{13}C\{^{1}H\}$ NMR ($CDCl_3$, 100 MHz) of **24k′**.

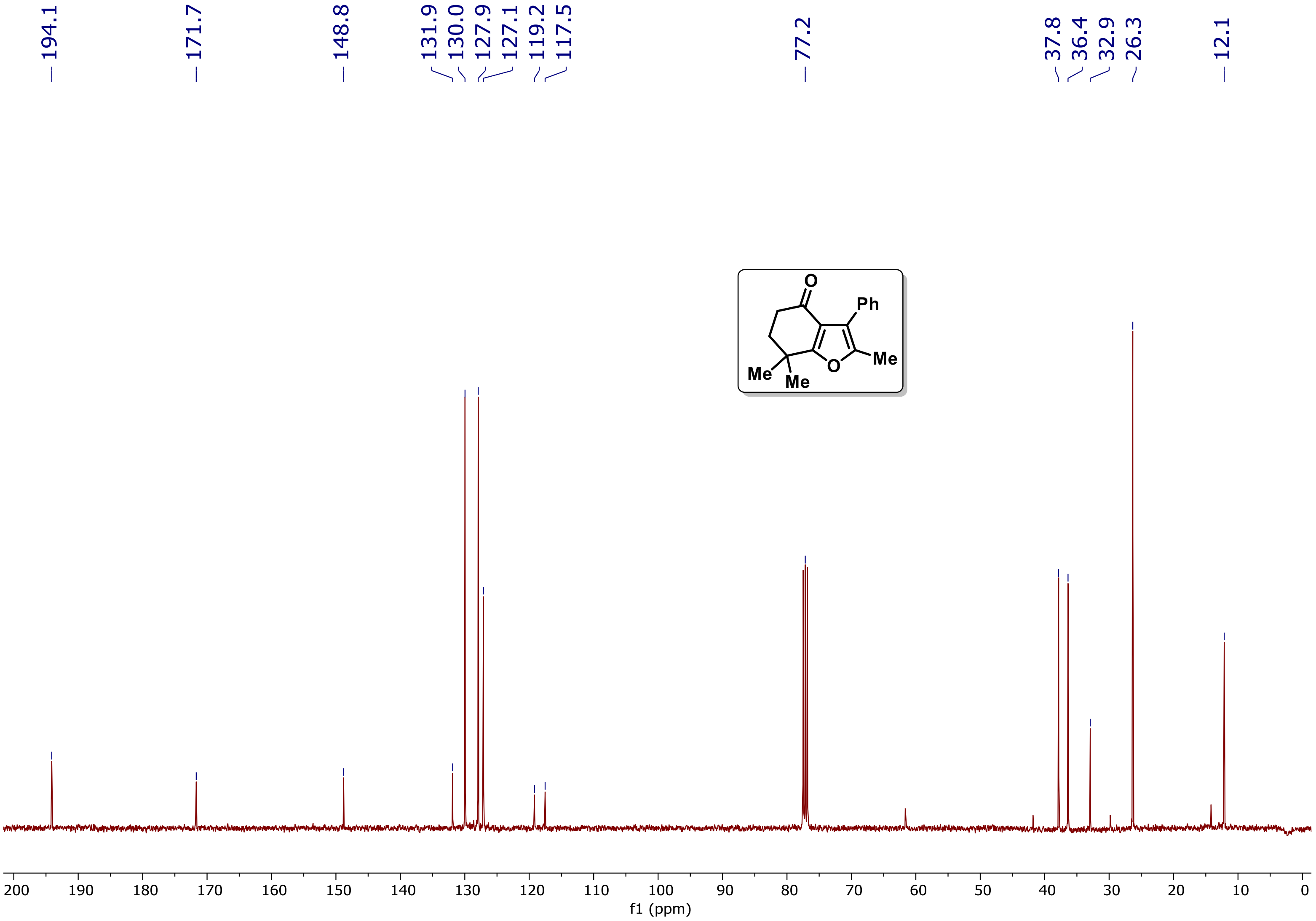

^{1}H NMR ($CDCl_3$, 300 MHz) of **24l**.

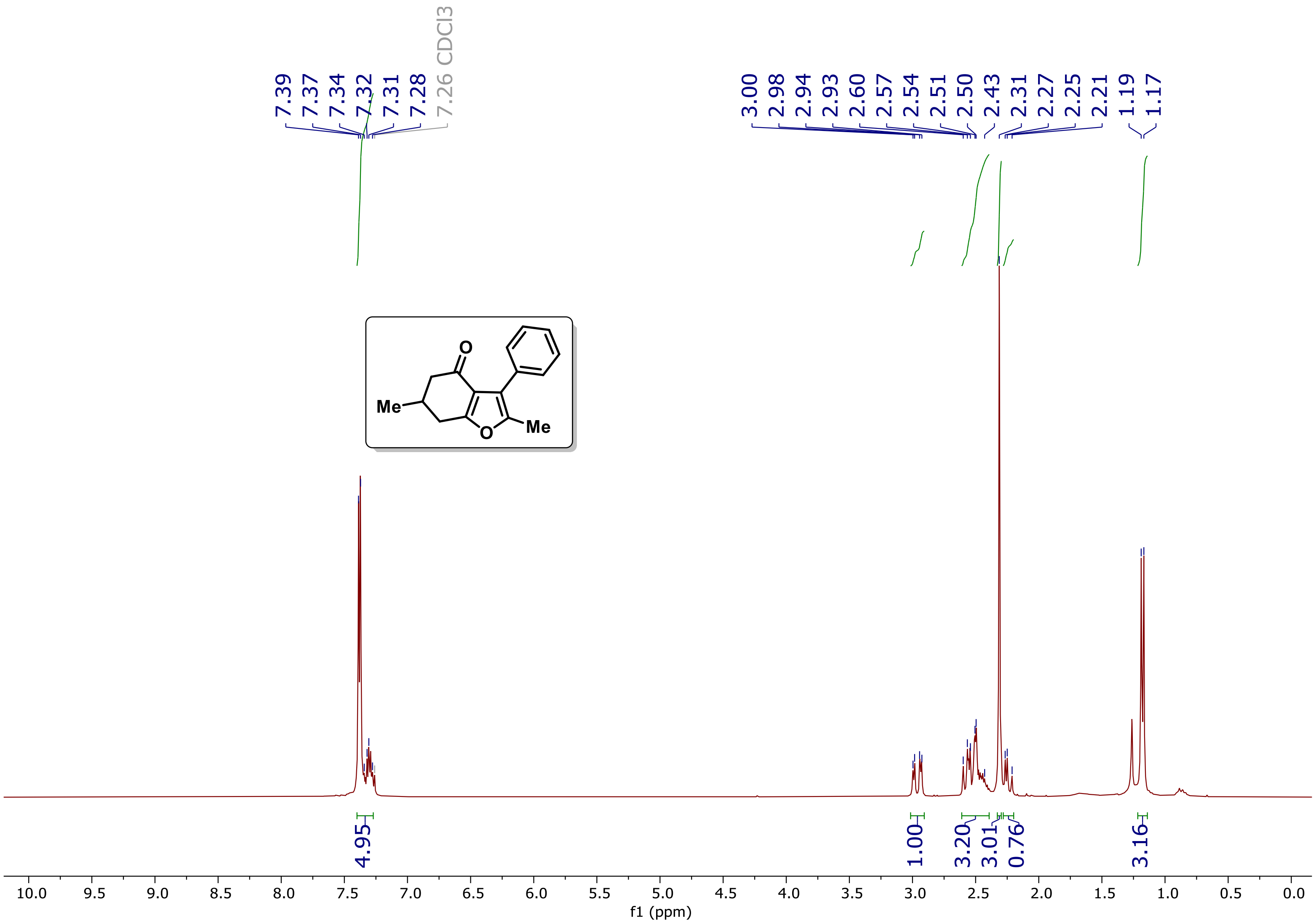

$^{13}C\{^{1}H\}$ NMR ($CDCl_3$, 75 MHz) of **24l**.

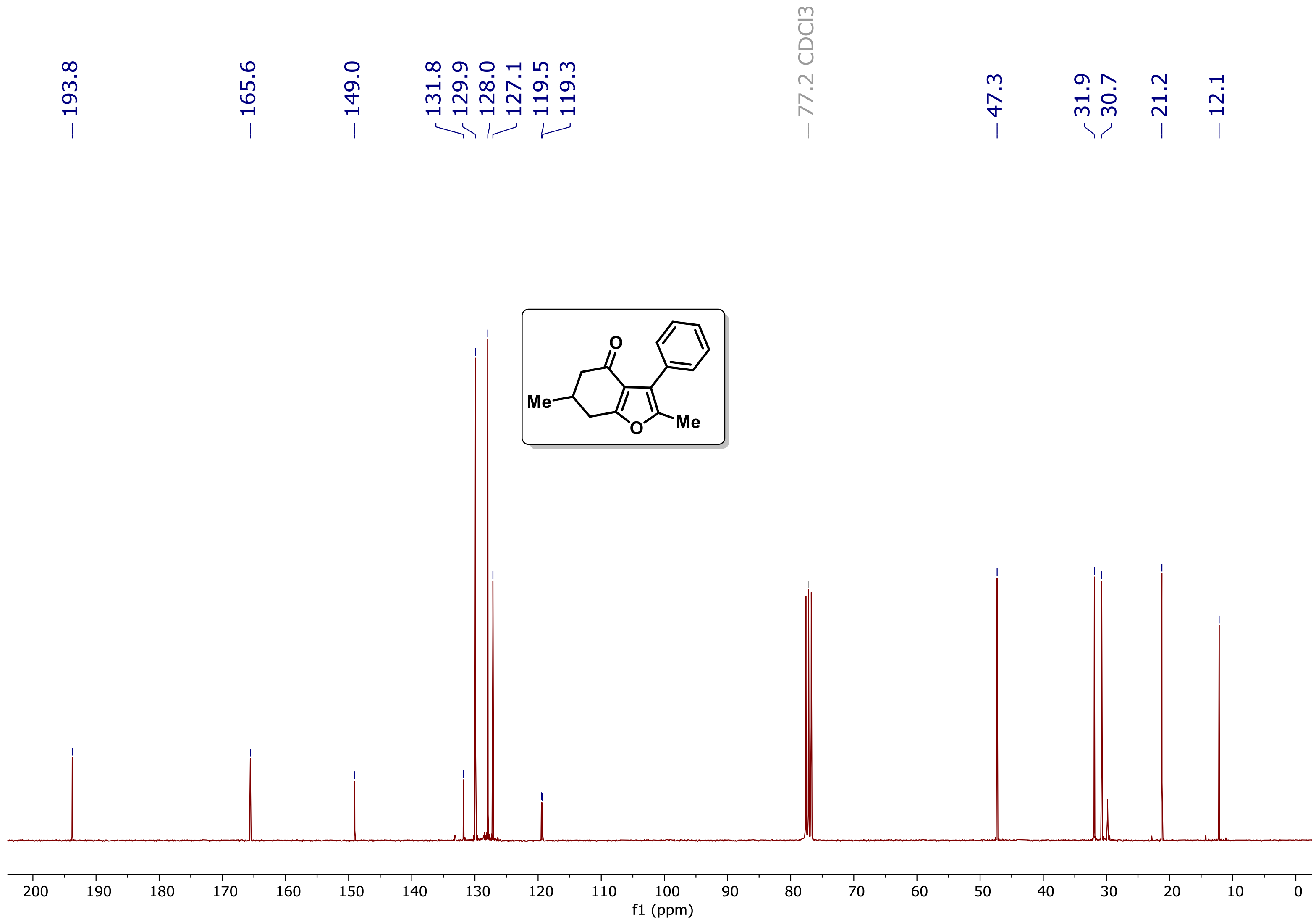

^{1}H NMR ($CDCl_3$, 400 MHz) of **24m**.

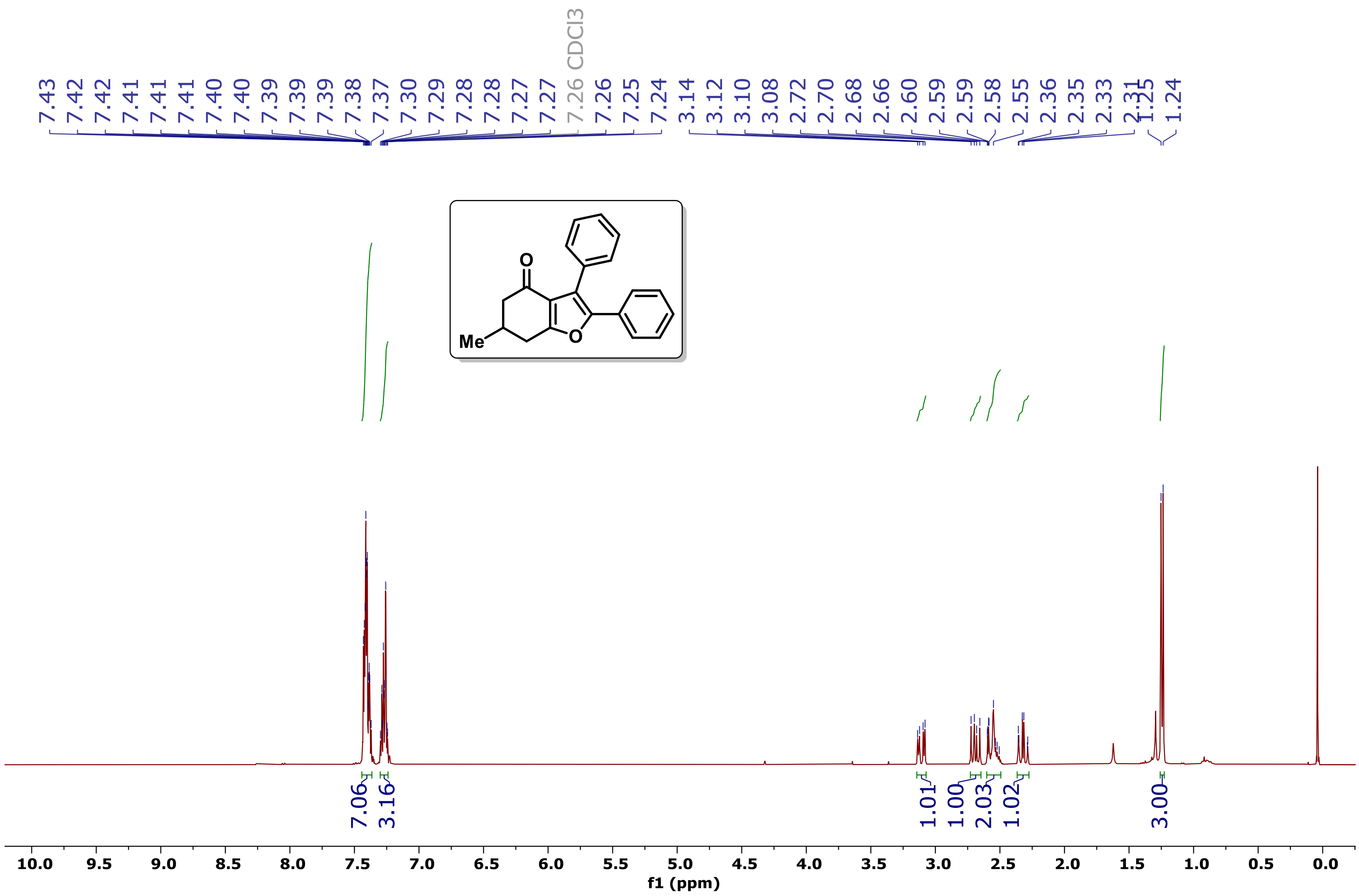

$^{13}C\{^1H\}$ NMR ($CDCl_3$, 100 MHz) of **24m**.

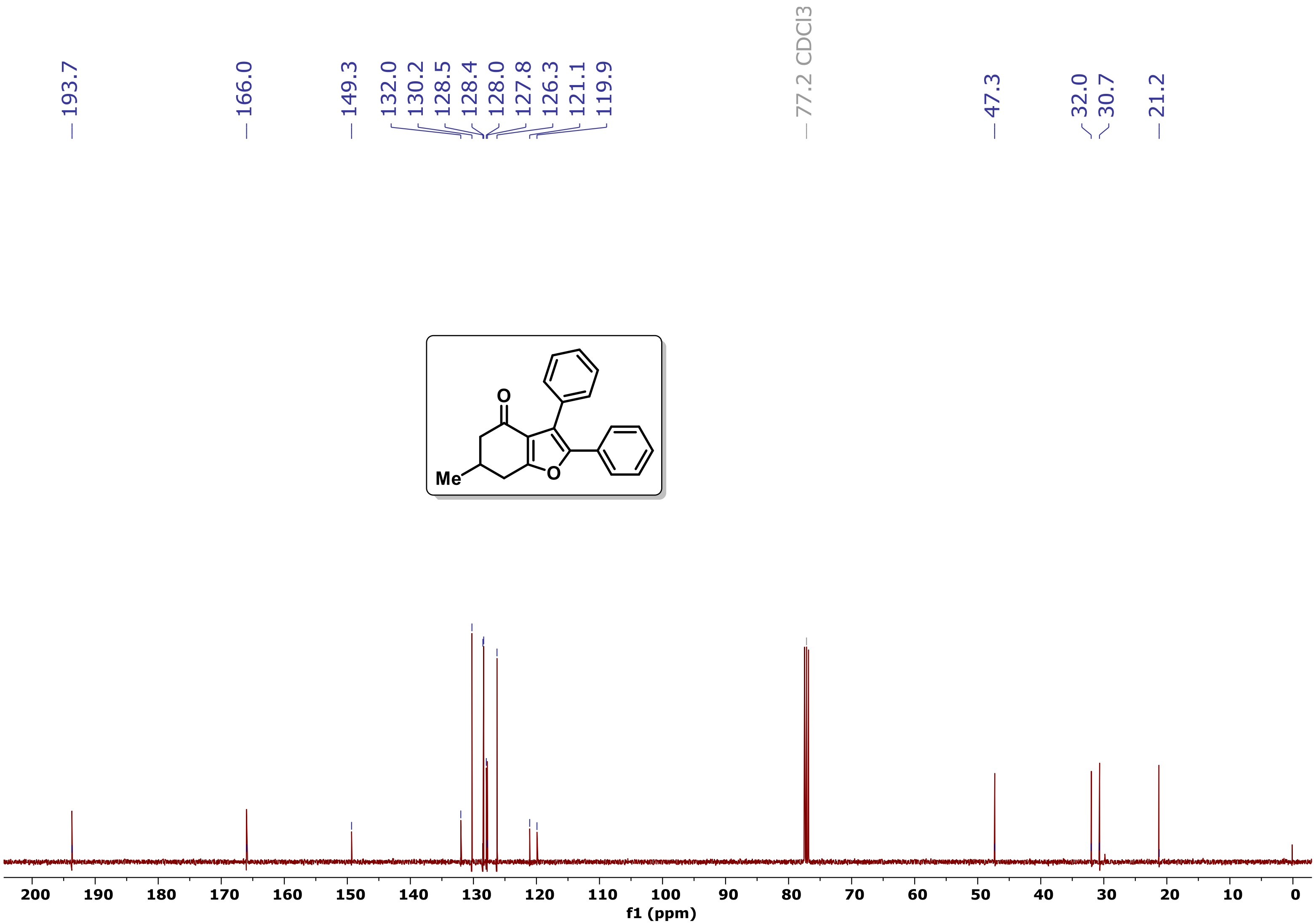

^{1}H NMR ($CDCl_3$, 300 MHz) of **24n**.

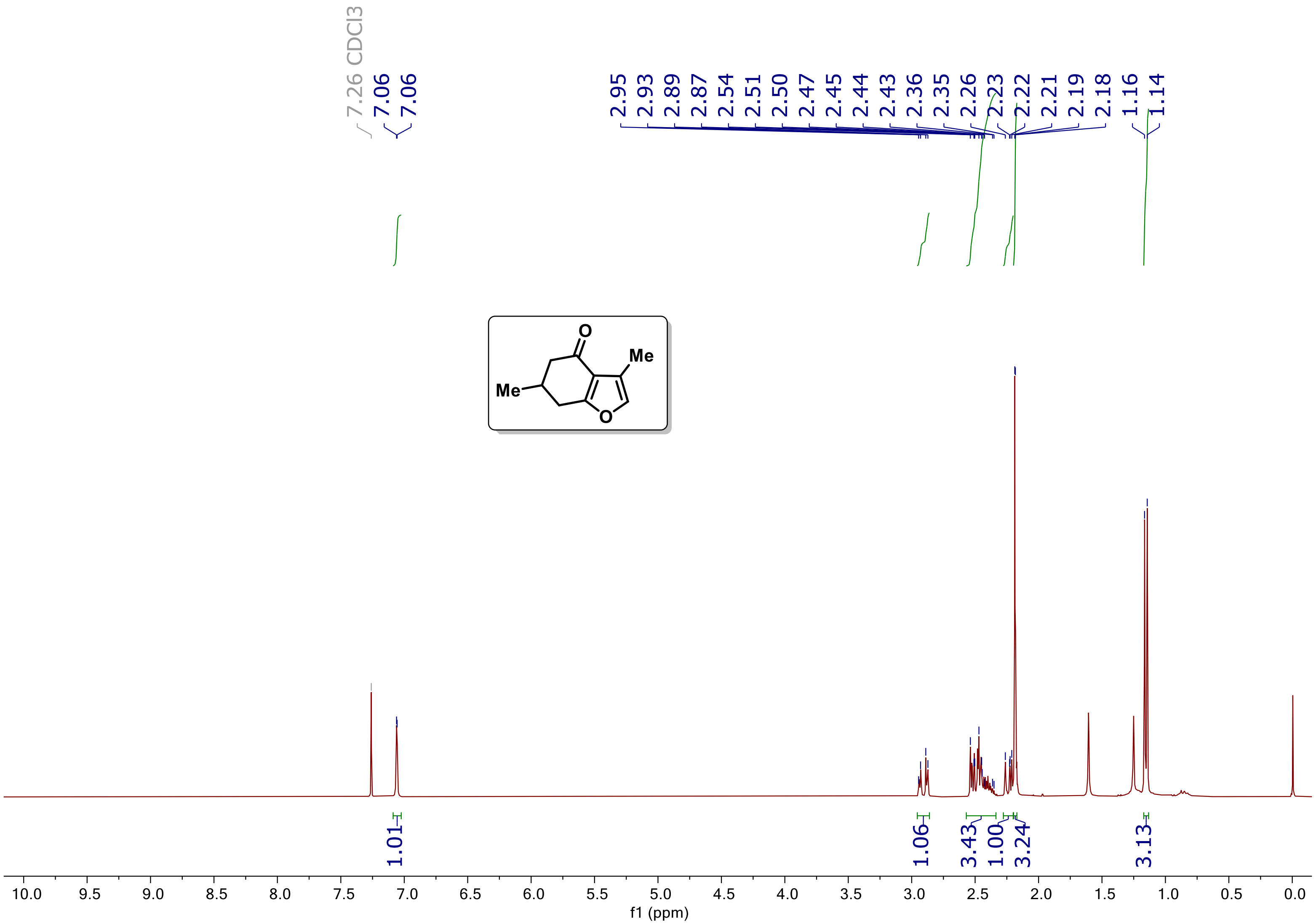

$^{13}C\{^1H\}$ NMR ($CDCl_3$, 75 MHz) of **24n**.

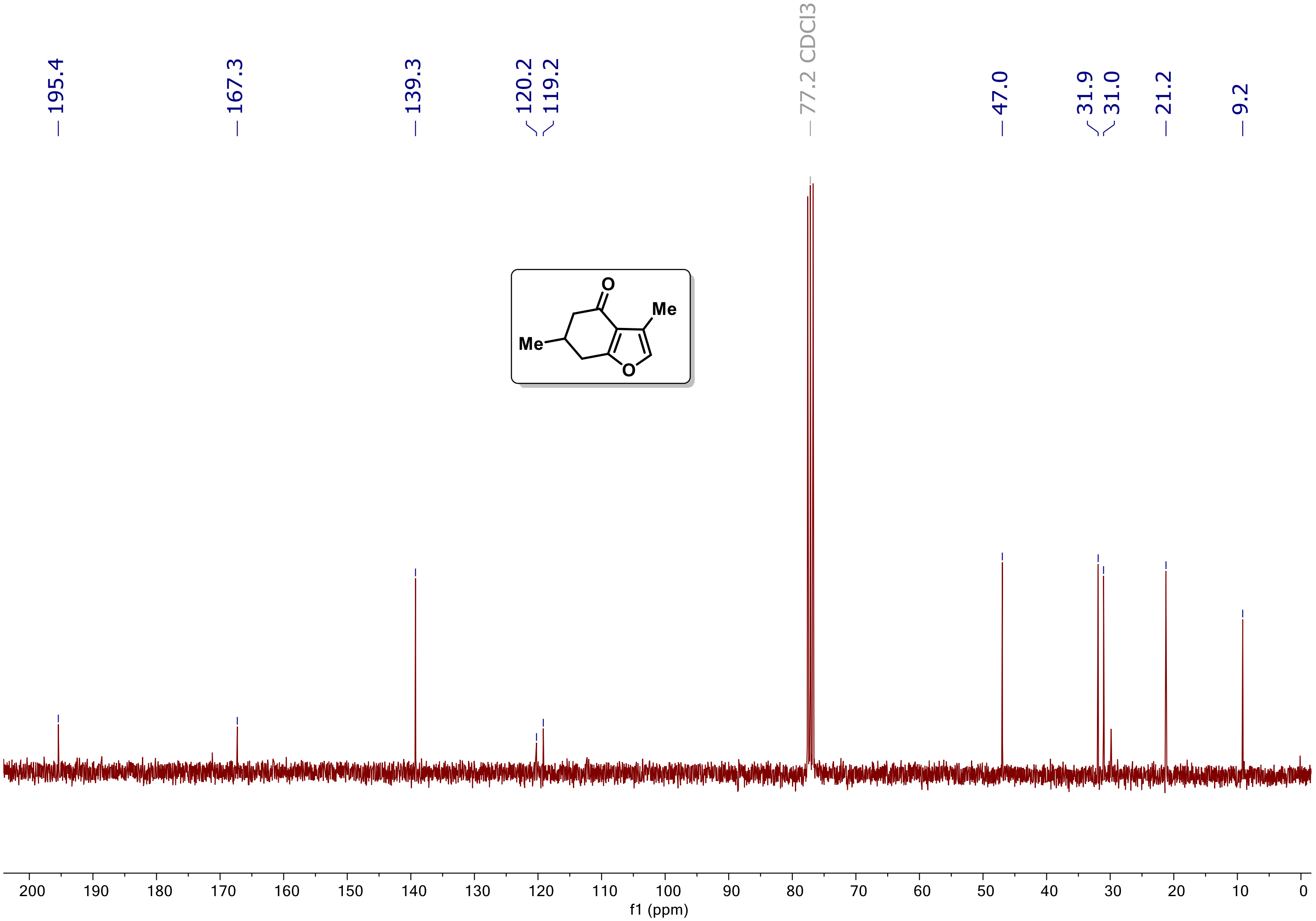

^{1}H NMR ($CDCl_3$, 400 MHz) of **24o**.

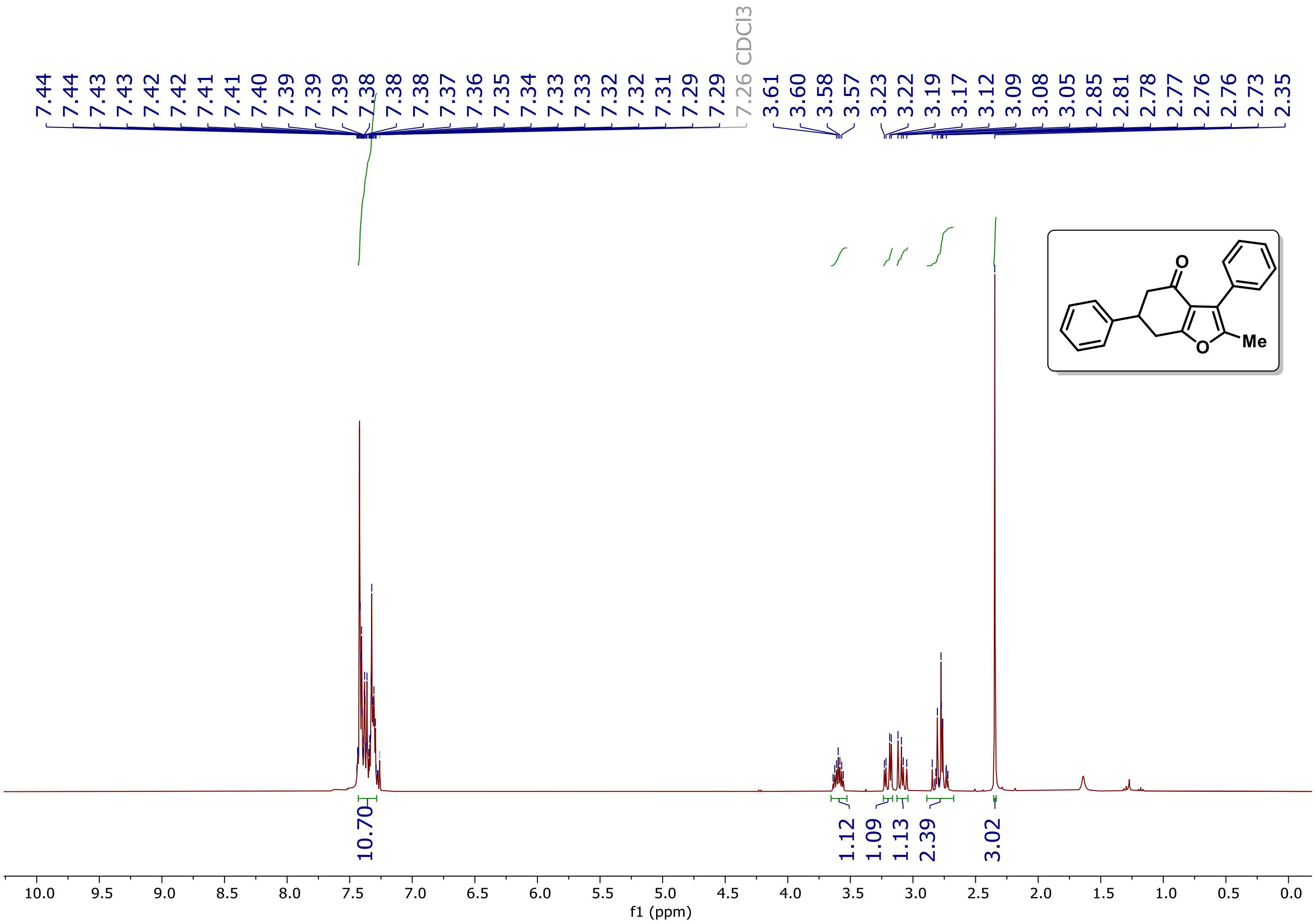

$^{13}C\{^{1}H\}$ NMR ($CDCl_3$, 100 MHz) of **24o**.

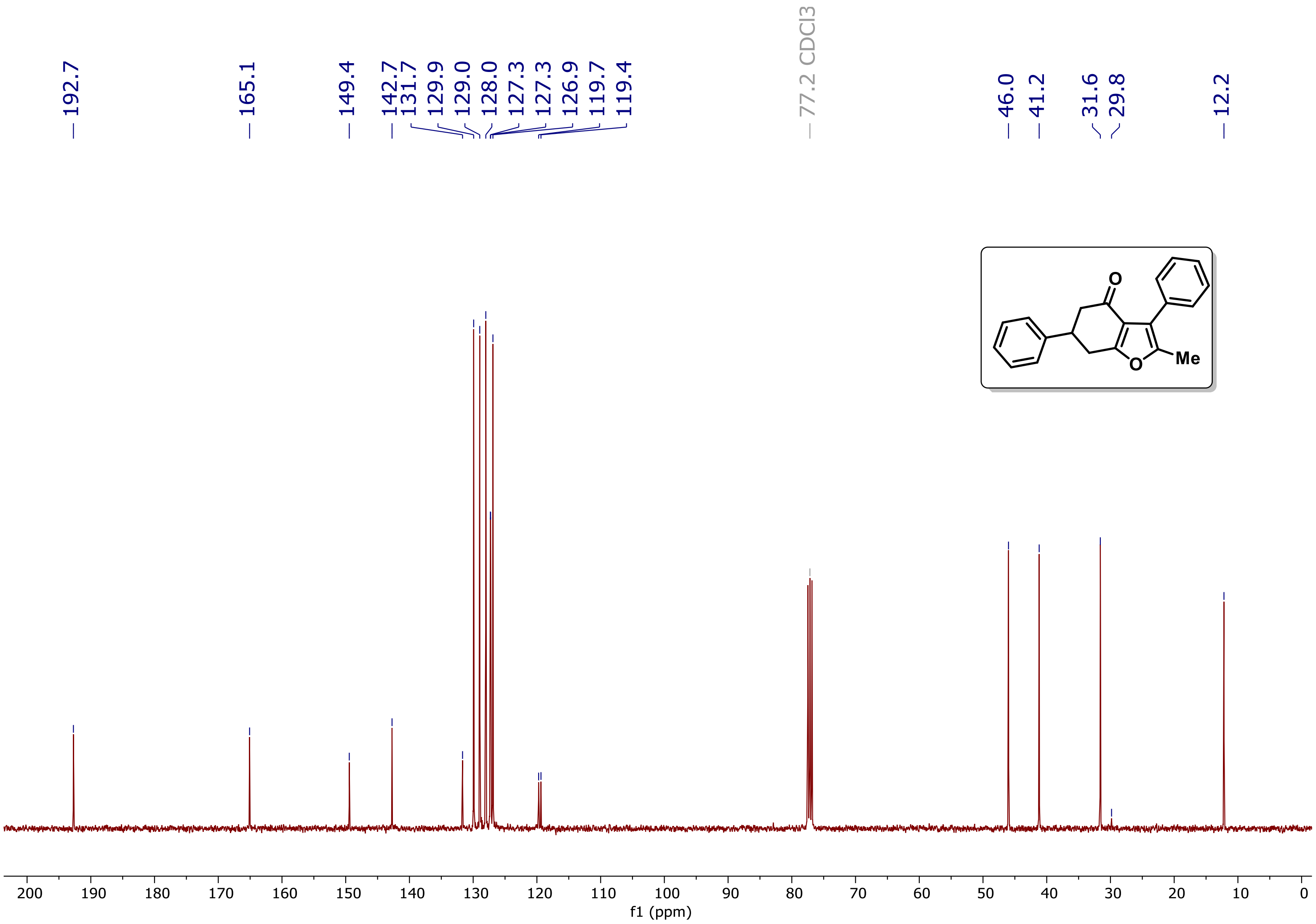

^{1}H NMR ($CDCl_3$, 300 MHz) of **24p**.

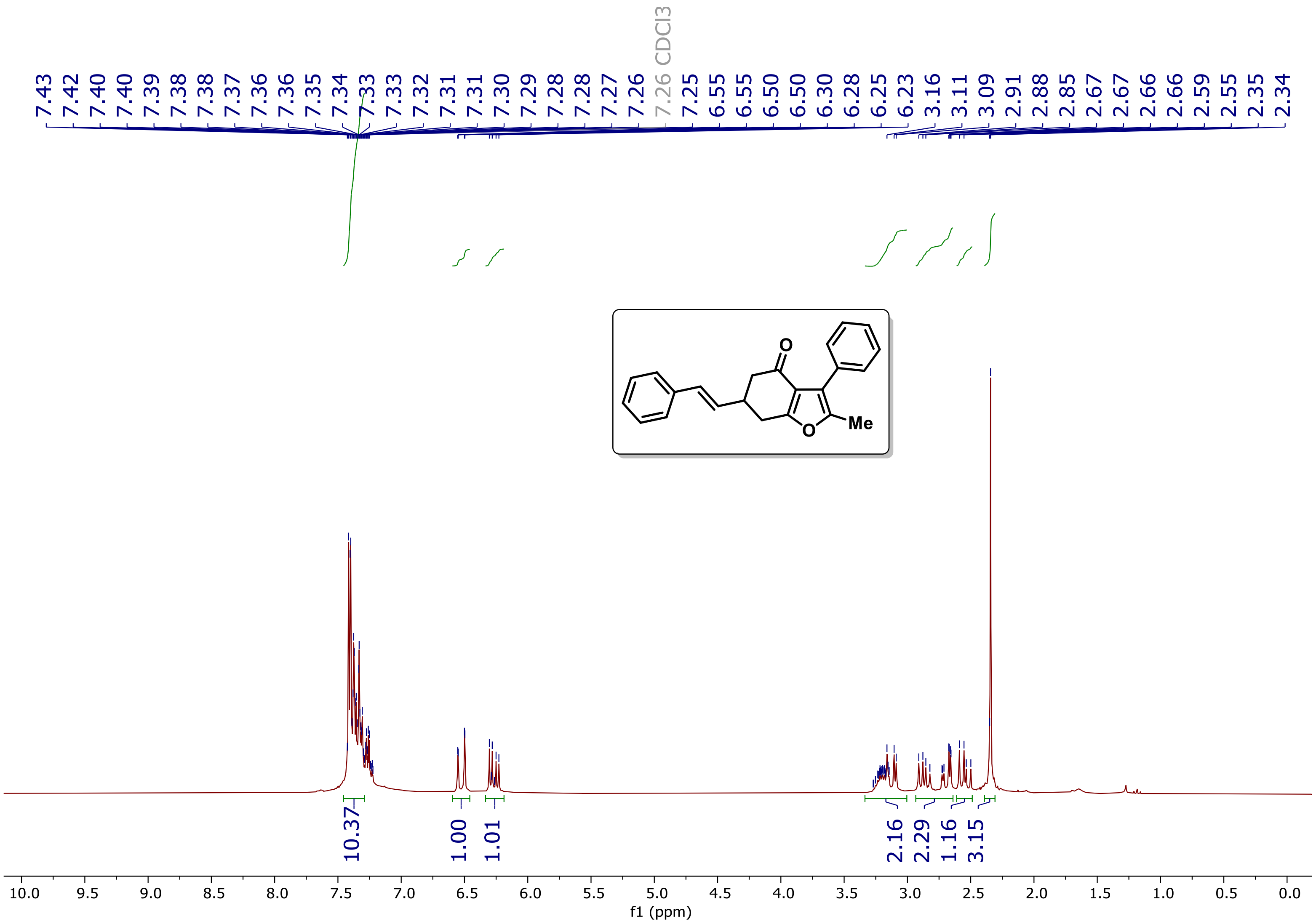

$^{13}C\{^{1}H\}$ NMR ($CDCl_3$, 75 MHz) of **24p**.

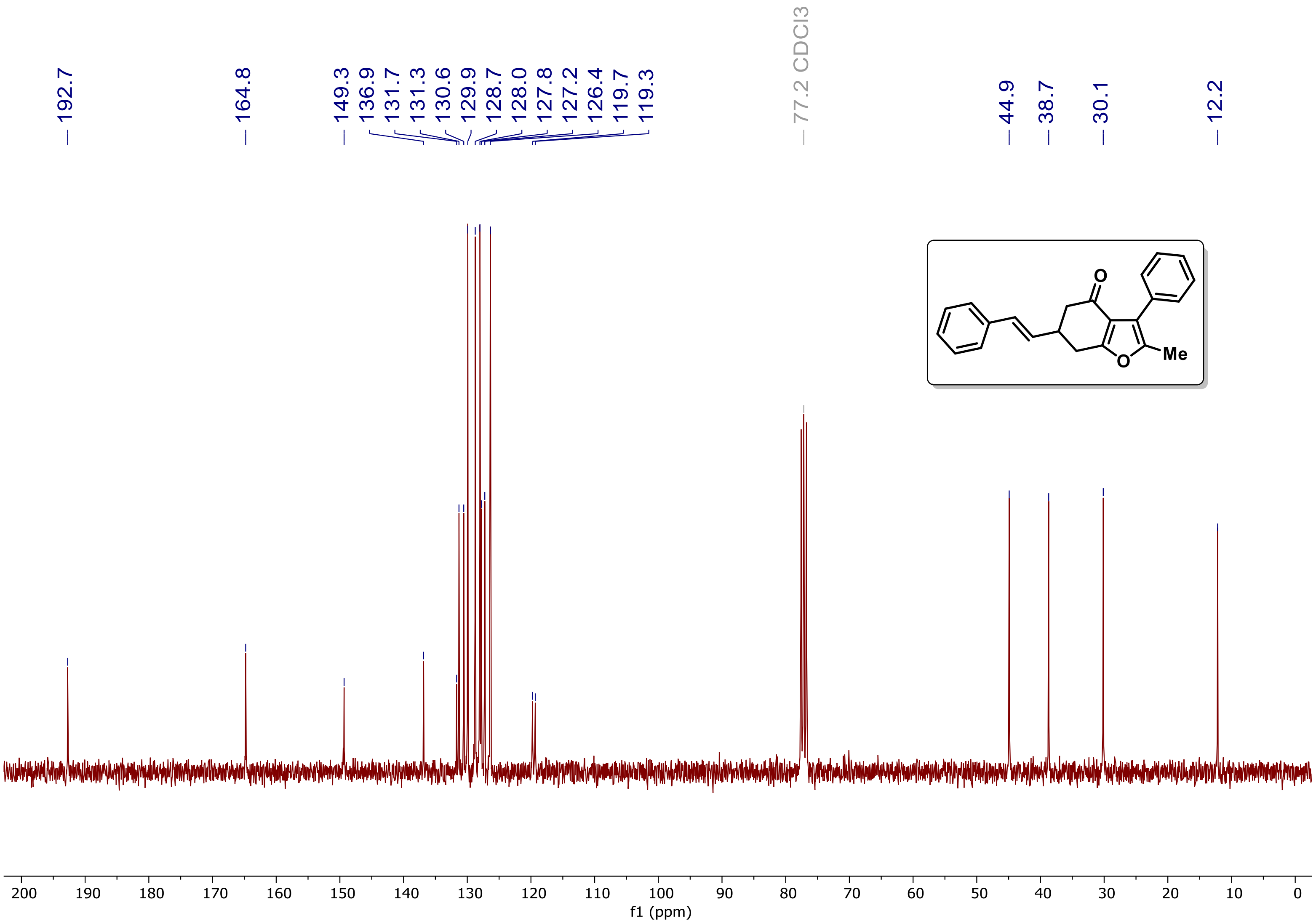

^{1}H NMR ($CDCl_3$, 300 MHz) of **24q**.

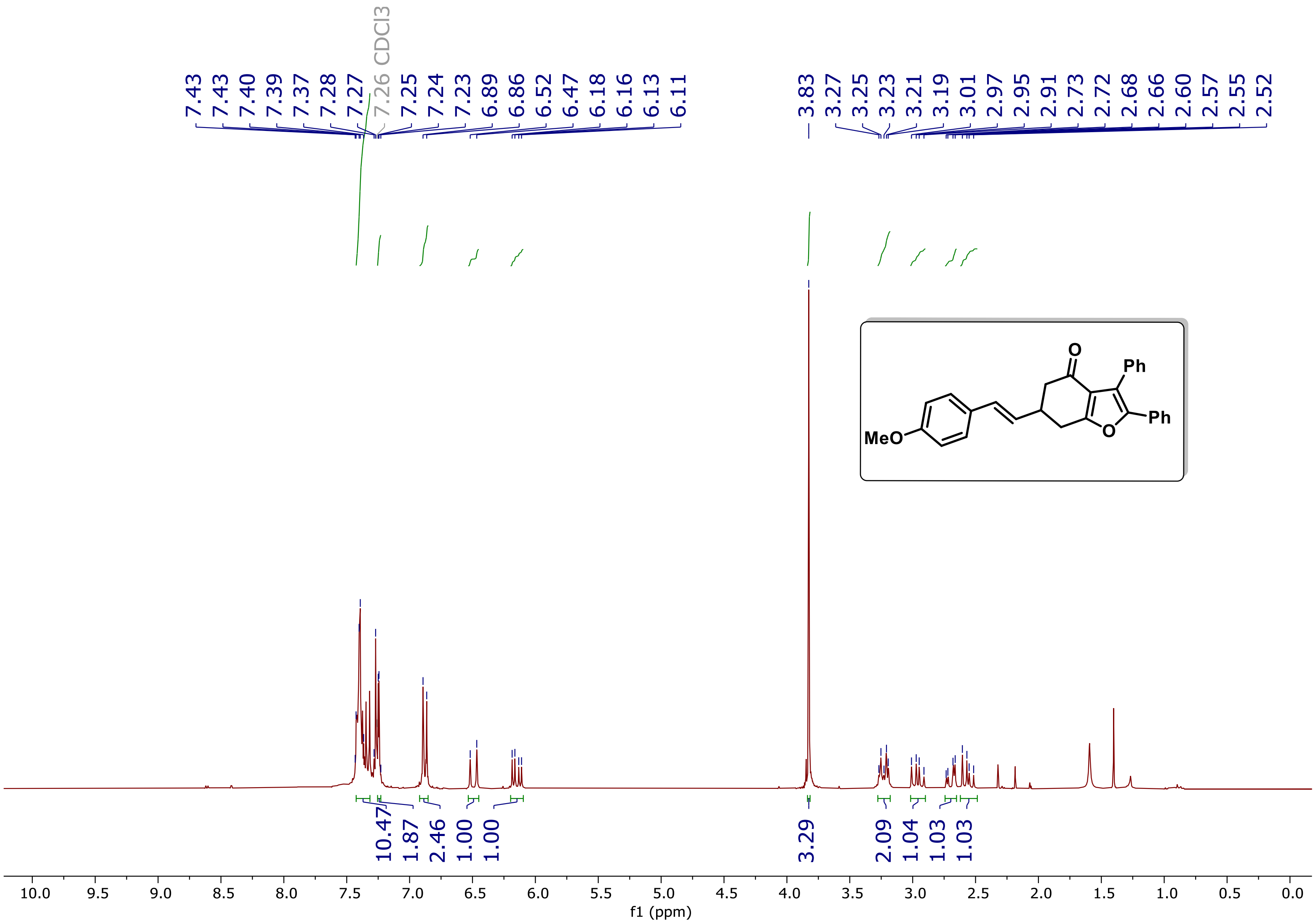

$^{13}C\{^{1}H\}$ NMR ($CDCl_3$, 75 MHz) of **24q**.

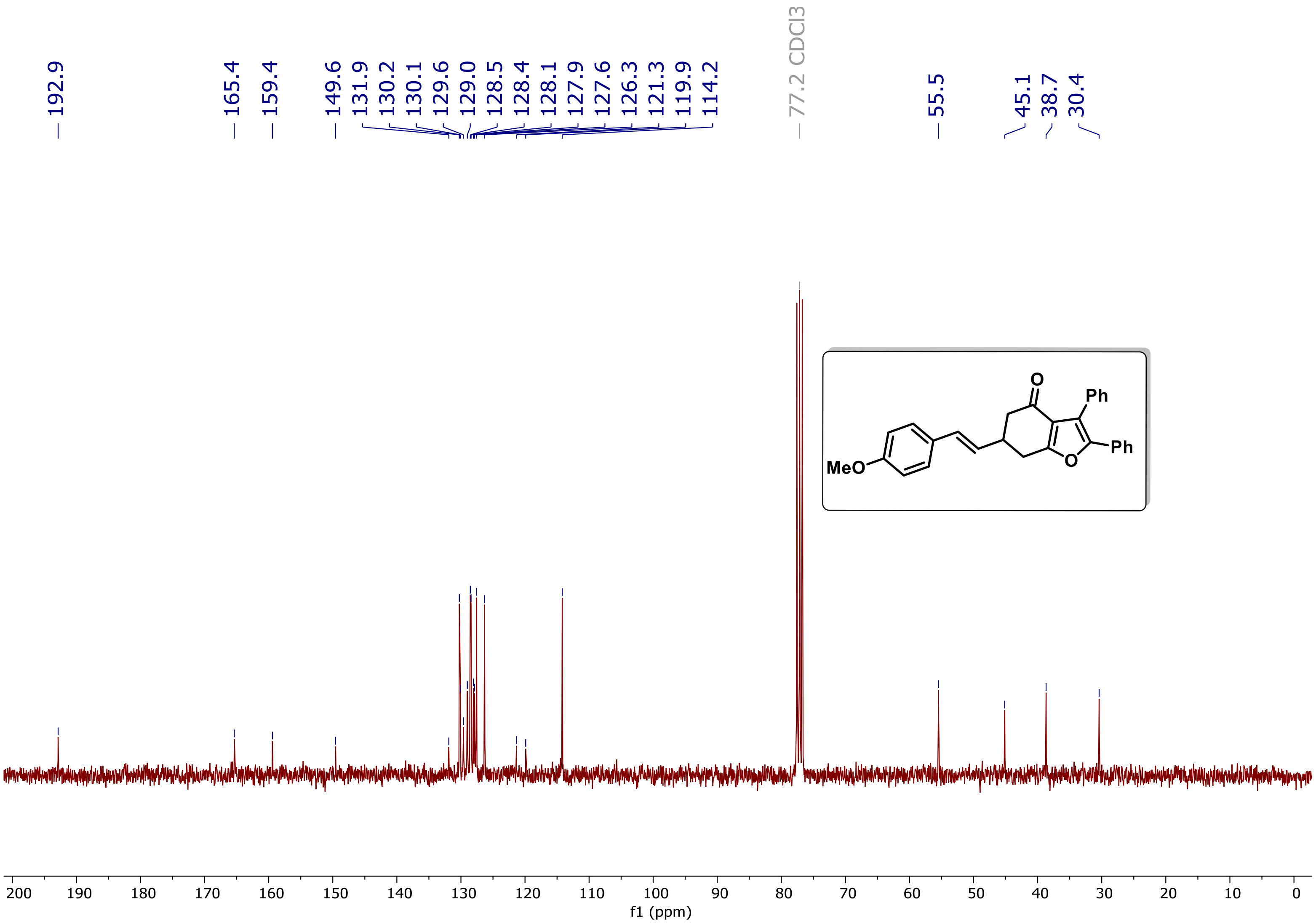

^{1}H NMR ($CDCl_3$, 400 MHz) of **24r**.

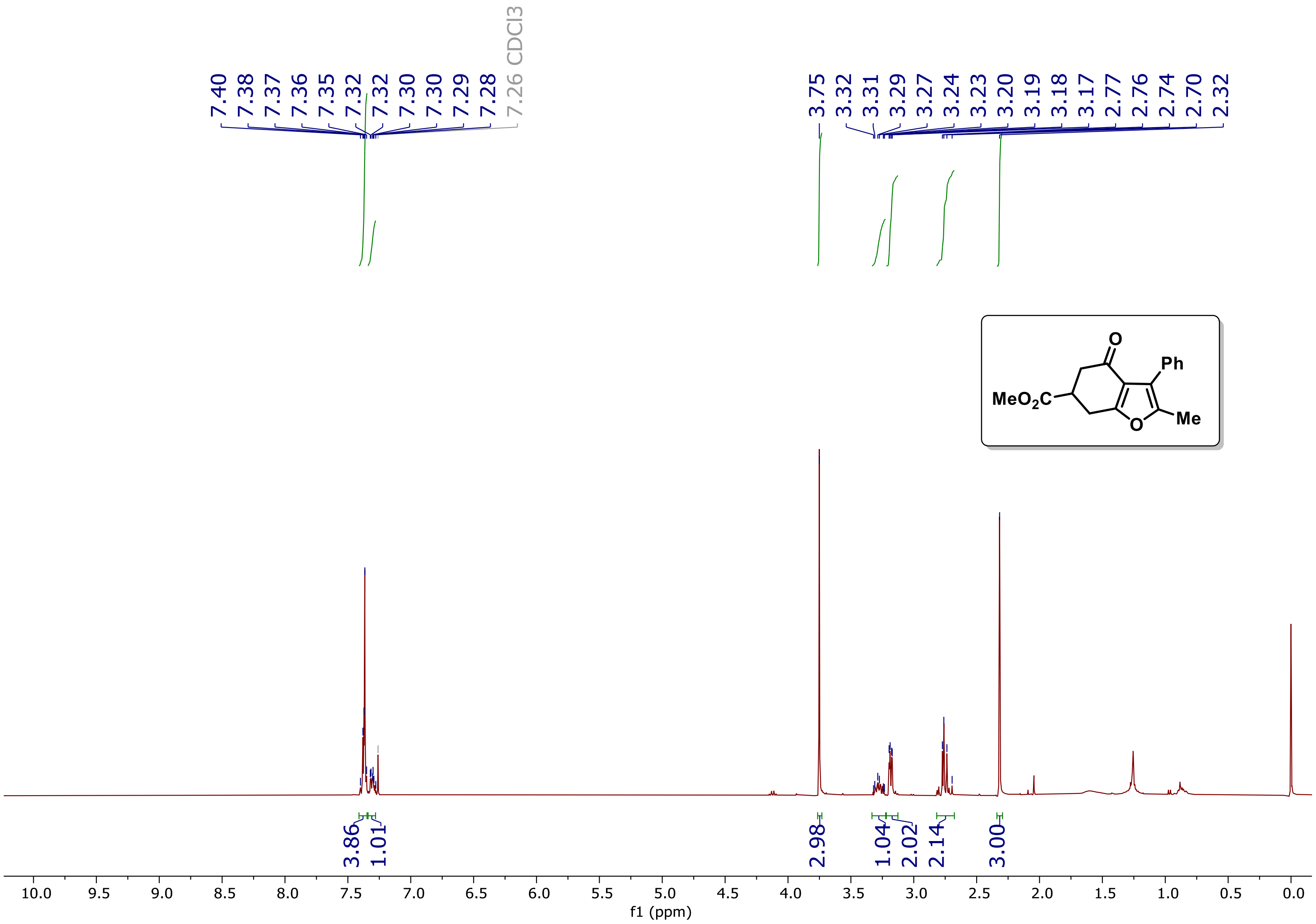

$^{13}C\{^1H\}$ NMR ($CDCl_3$, 100 MHz) of **24r**.

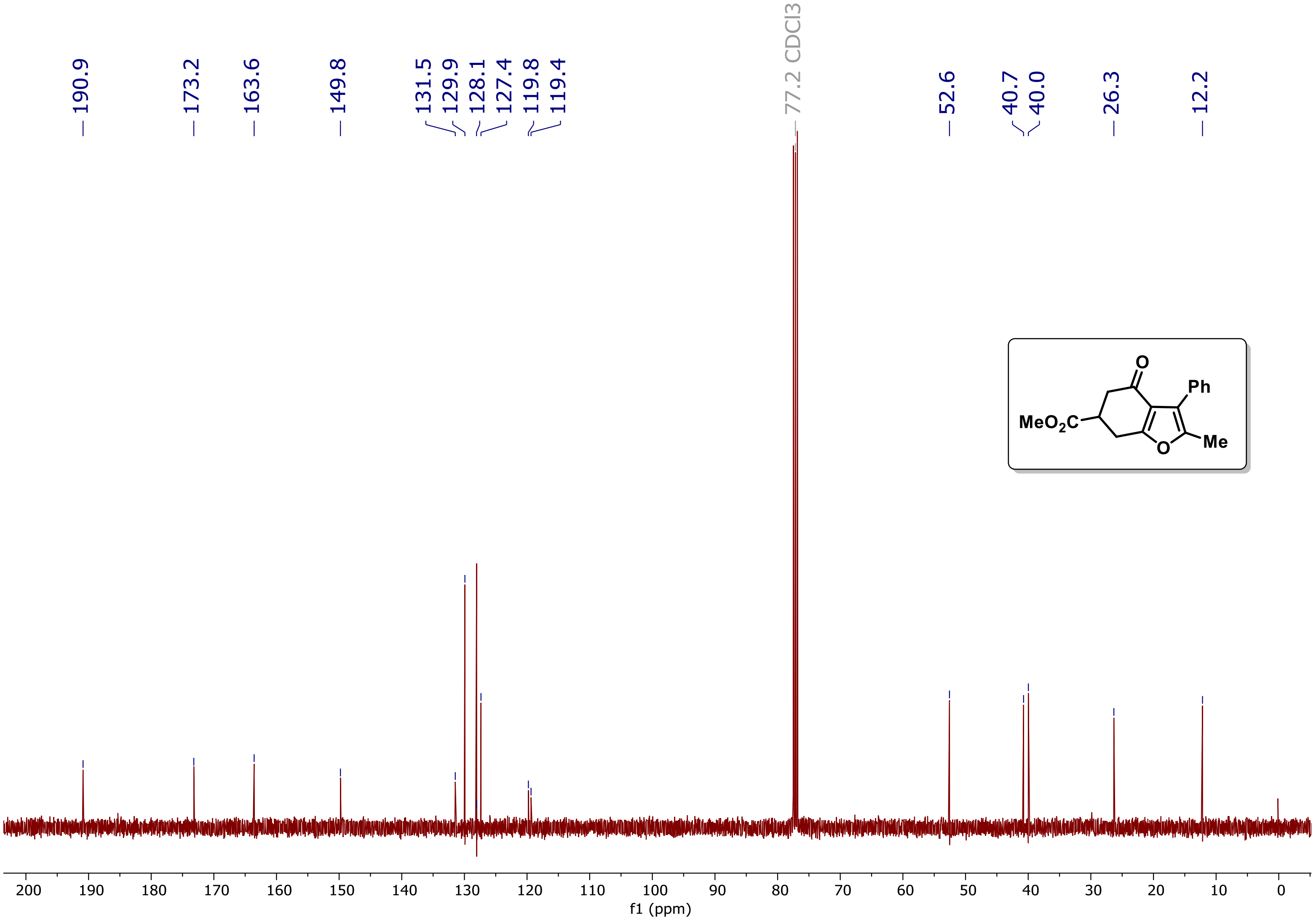

^{1}H NMR ($CDCl_3$, 400 MHz) of **24s**.

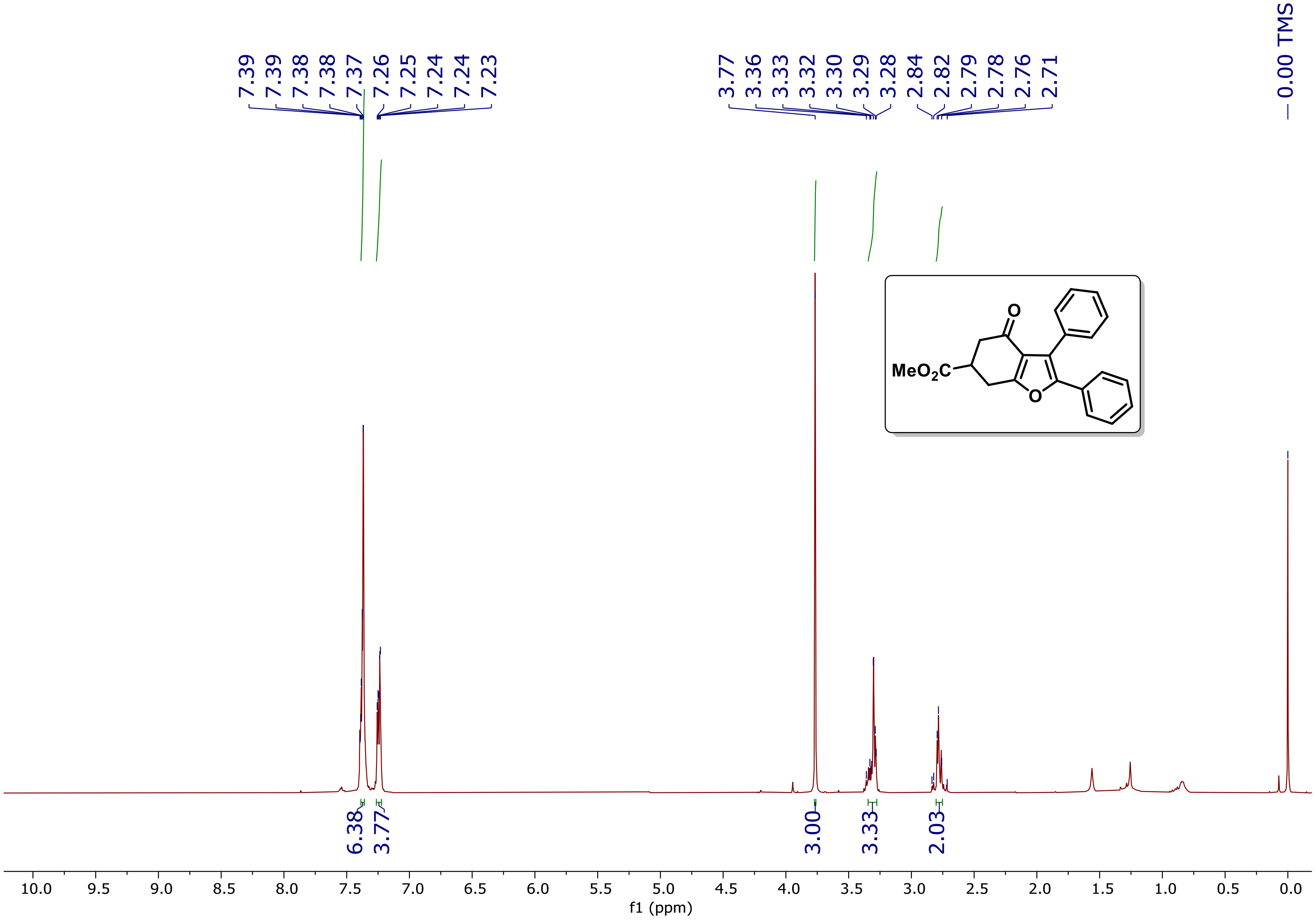

$^{13}C\{^{1}H\}$ NMR ($CDCl_3$, 100 MHz) of **24s**.

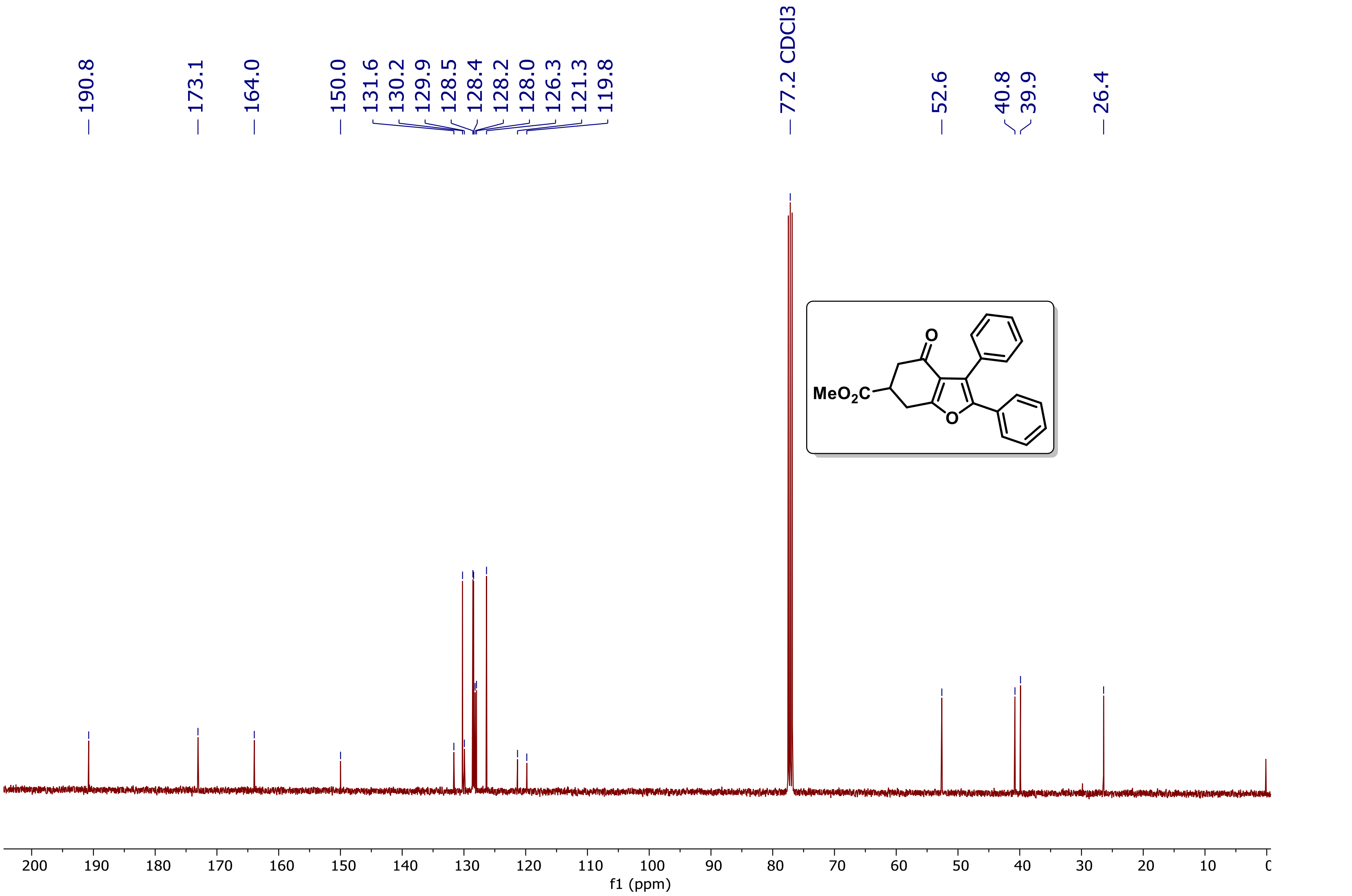

^{1}H NMR (CDCl3, 300 MHz) of **24t**.

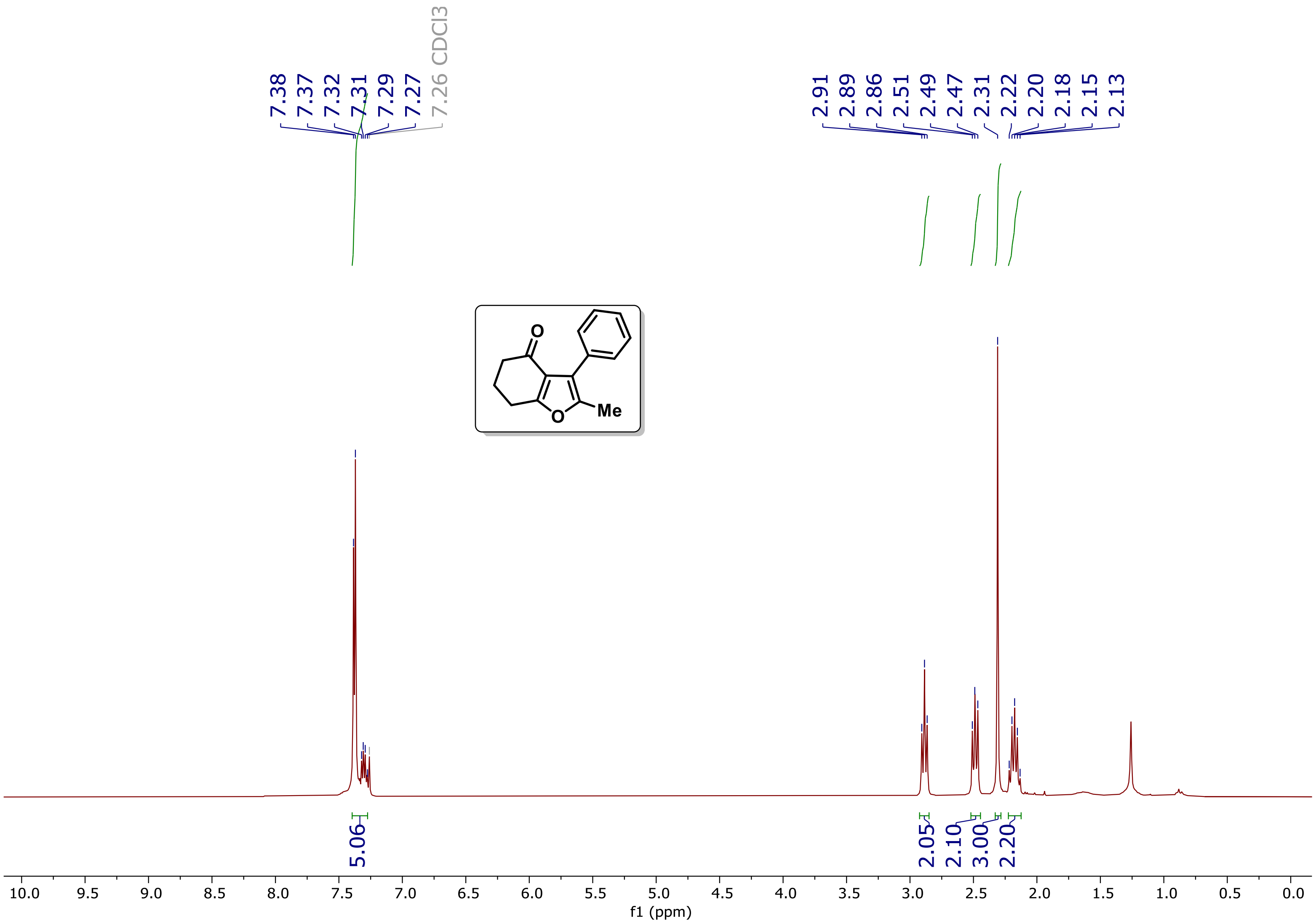

$^{13}C\{^{1}H\}$ NMR ($CDCl_3$, 75 MHz) of **24t**.

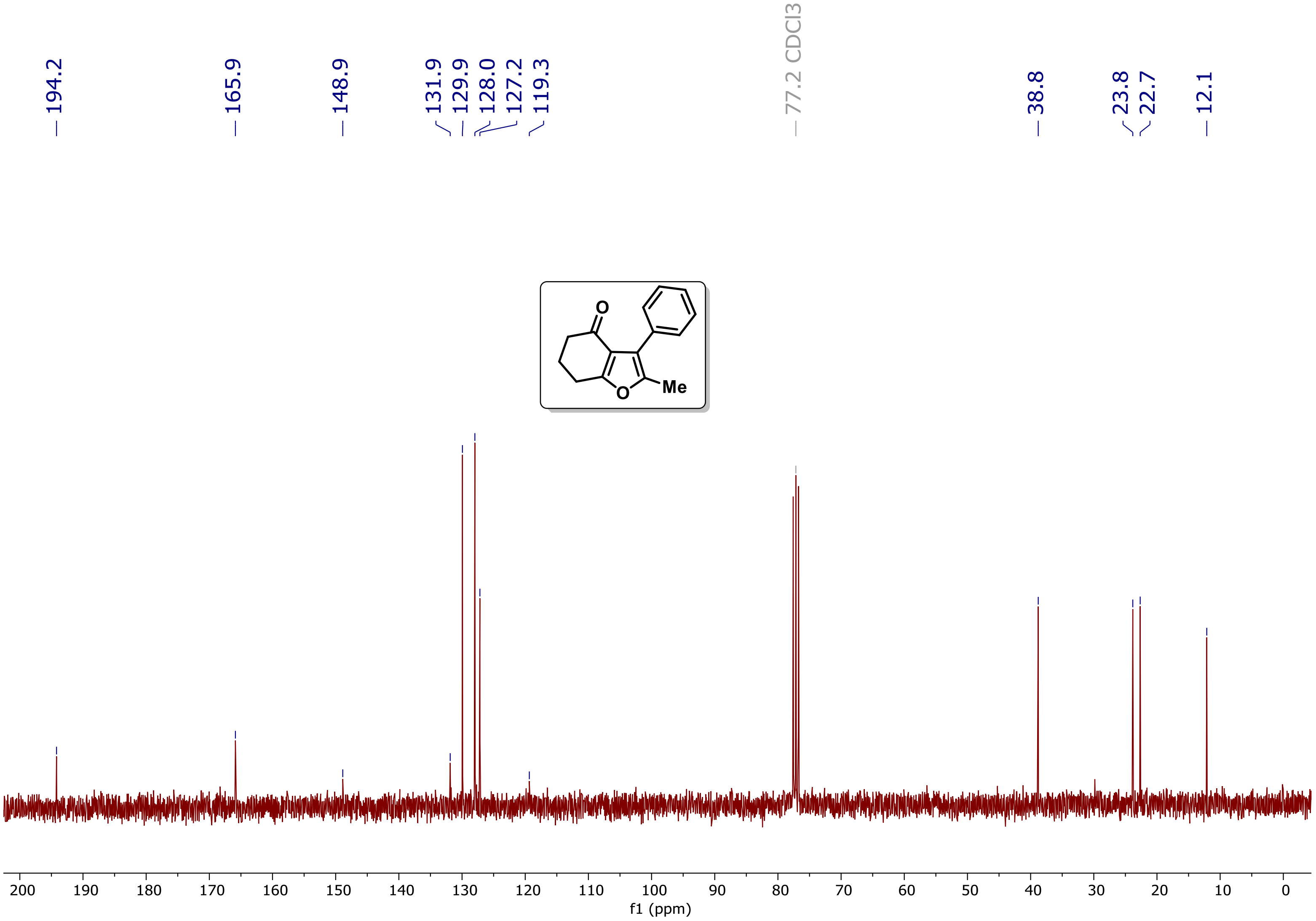

^{1}H NMR ($CDCl_3$, 300 MHz) of **24u**.

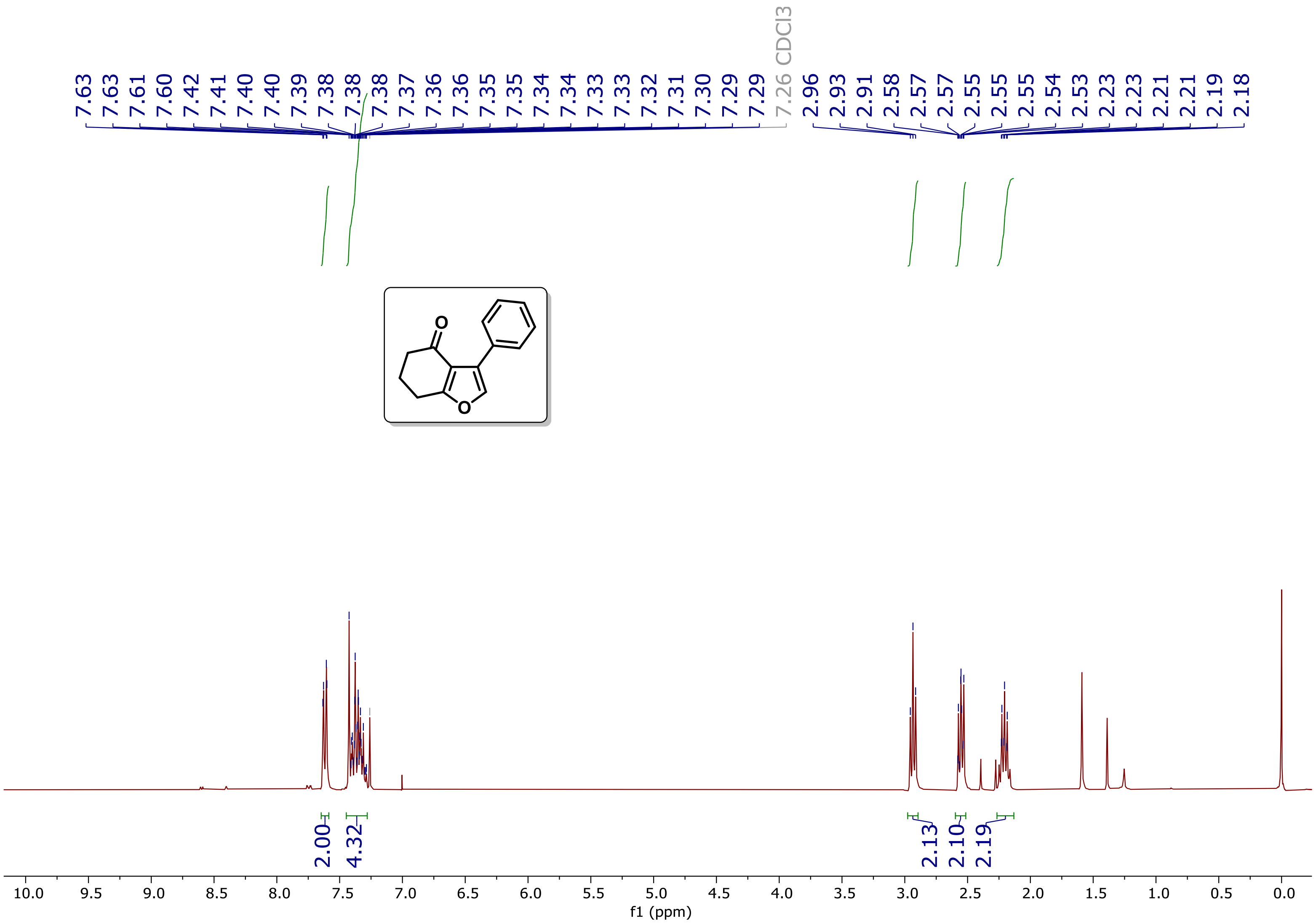

^{1}H NMR ($CDCl_3$, 500 MHz) of **24v**.

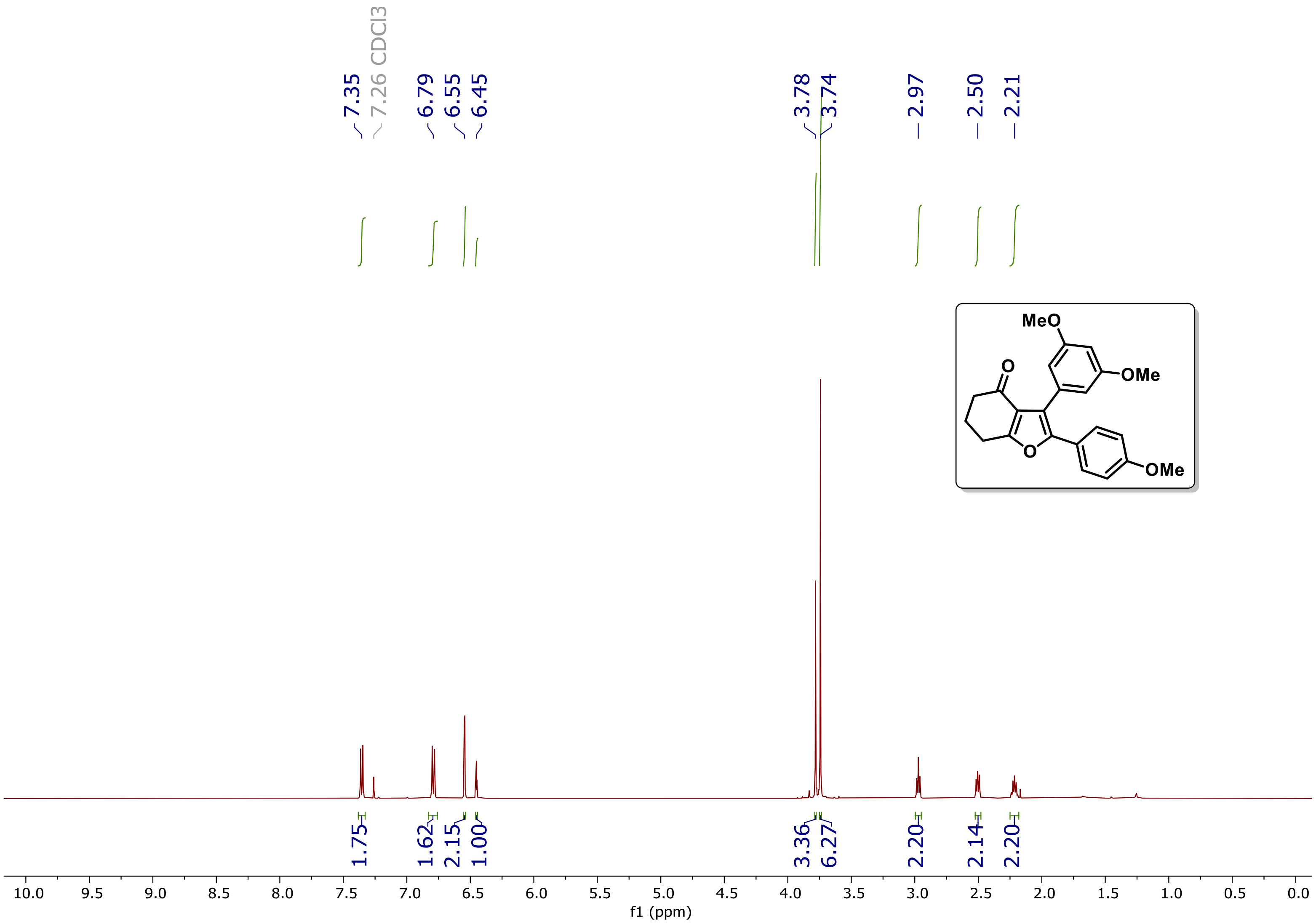

$^{13}C\{^{1}H\}$ NMR ($CDCl_3$, 126 MHz) of **24v**.

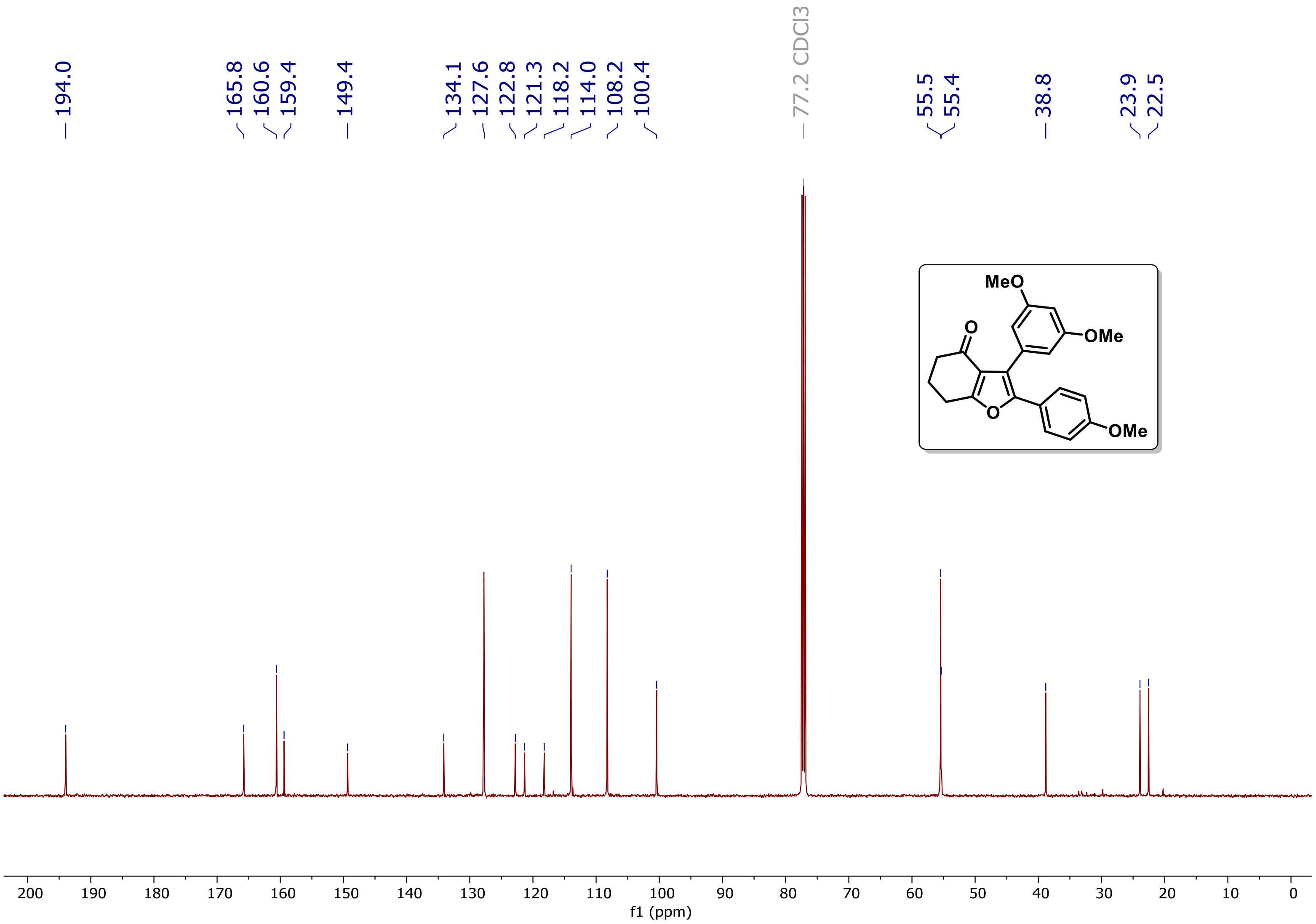

^{1}H NMR ($CDCl_3$, 300 MHz) of **24w**.

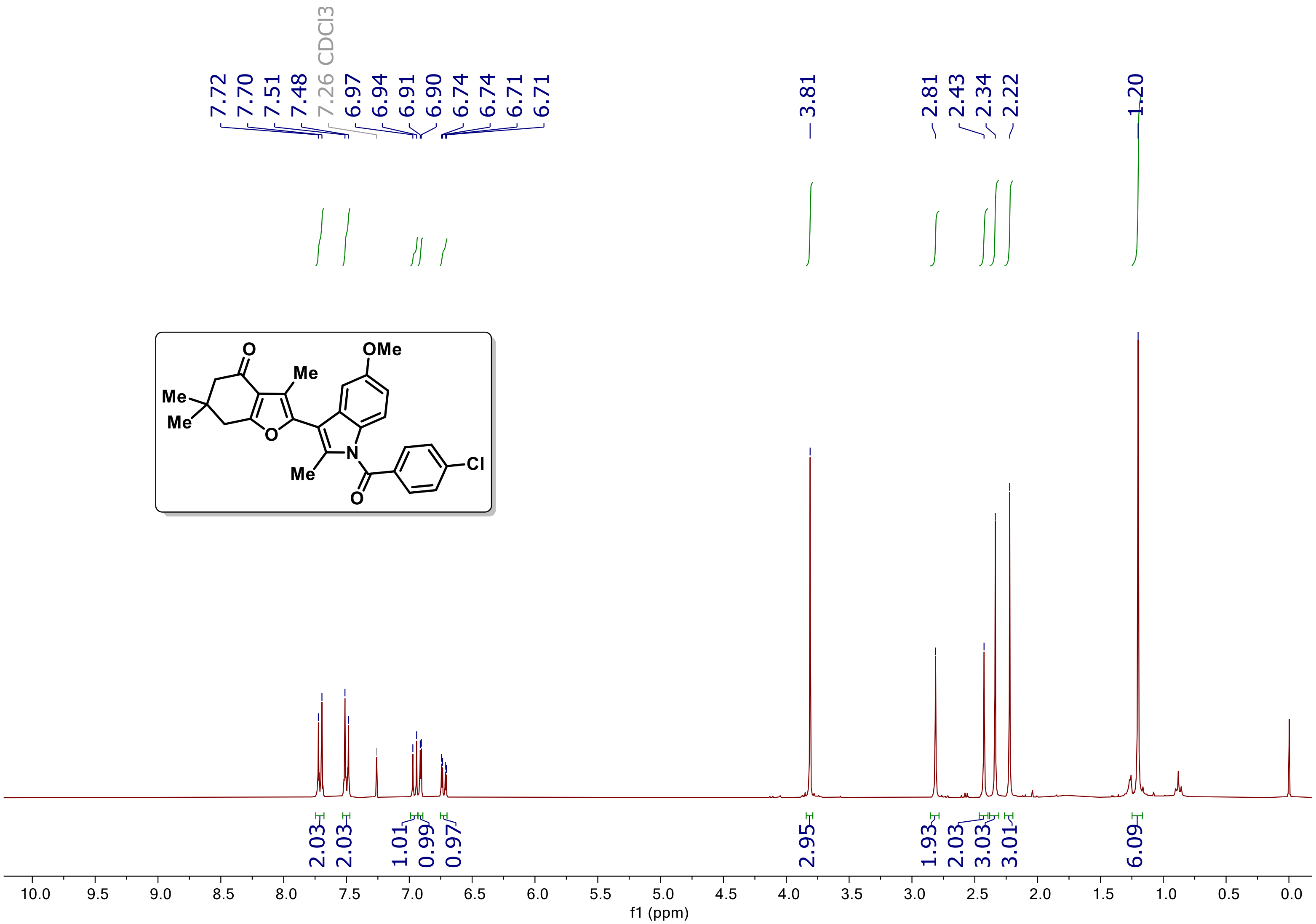

$^{13}C\{^1H\}$ NMR ($CDCl_3$, 75 MHz) of **24w**.

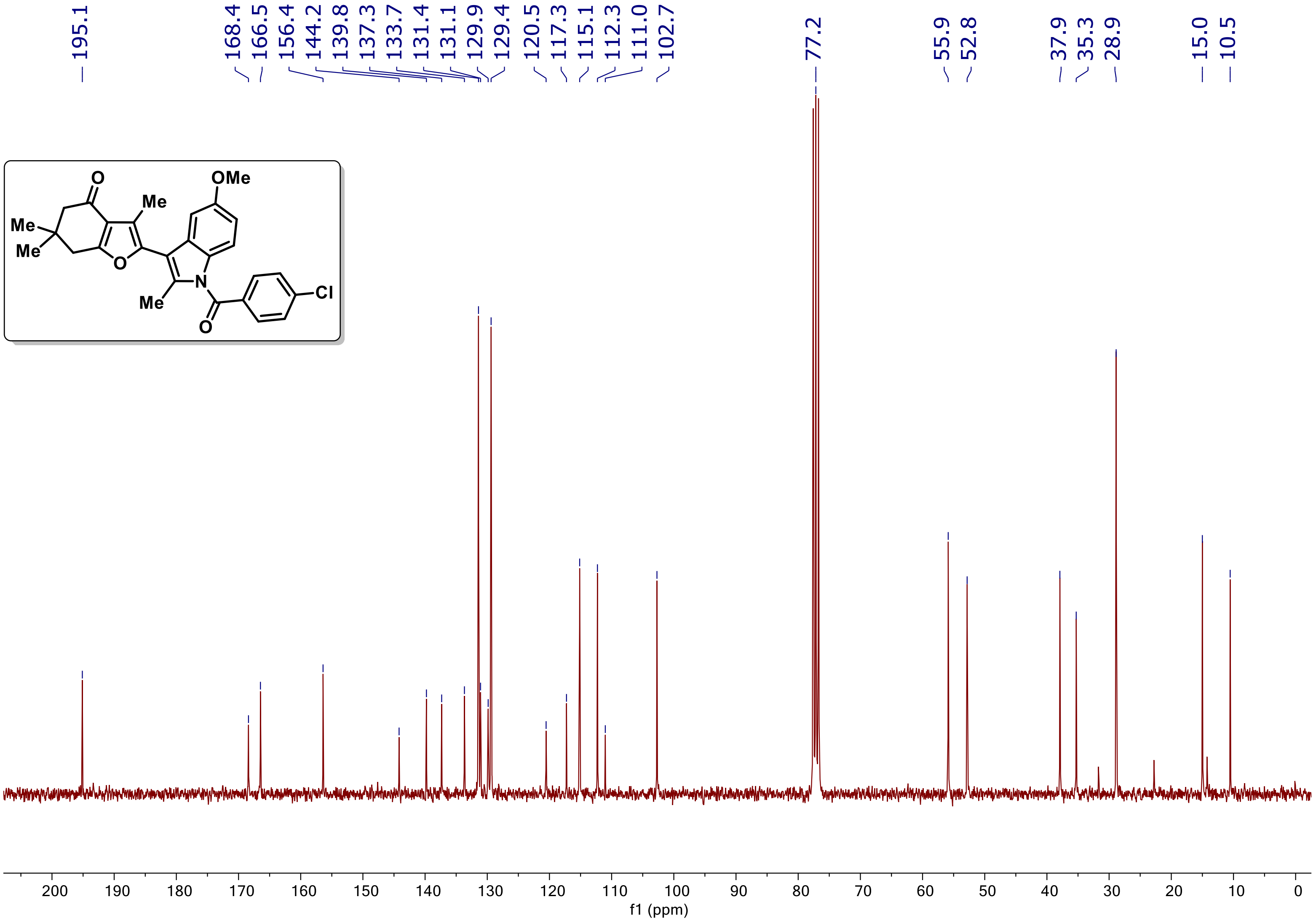

^{1}H NMR ($CDCl_3$, 300 MHz) of **24x**.

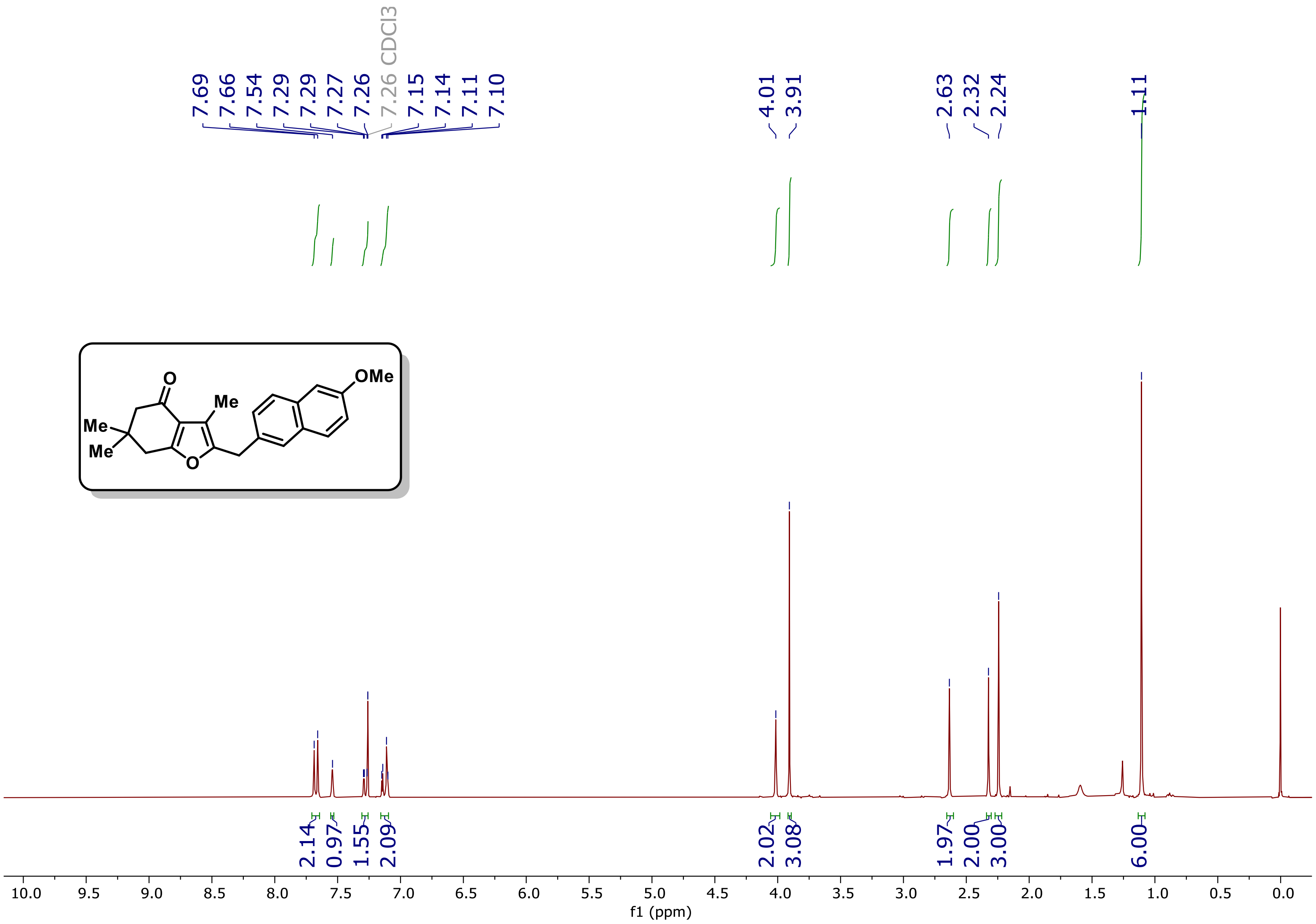

$^{13}C\{^1H\}$ NMR ($CDCl_3$, 75 MHz) of **24x**.

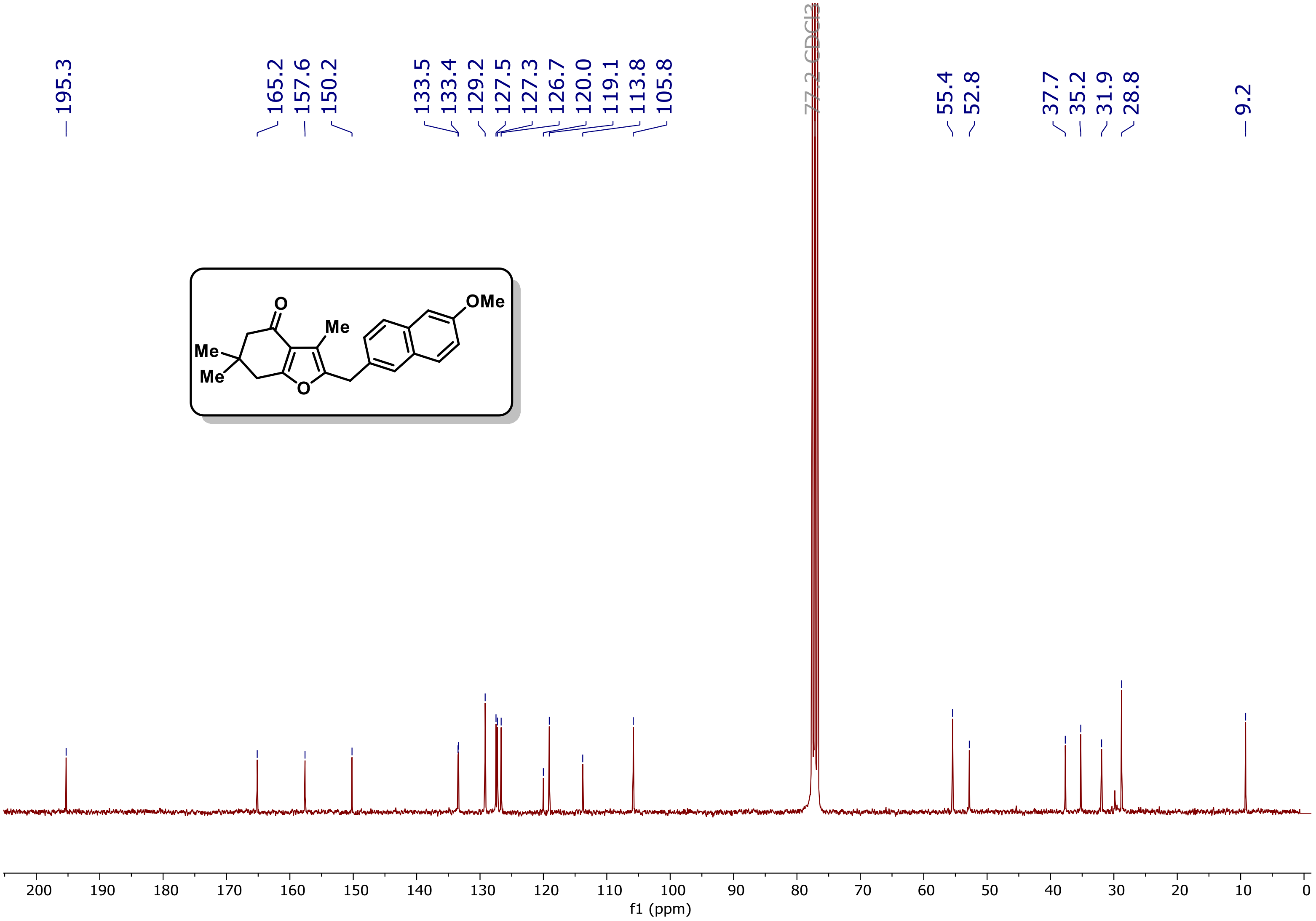

^{1}H NMR ($CDCl_3$, 300 MHz) of **25**.

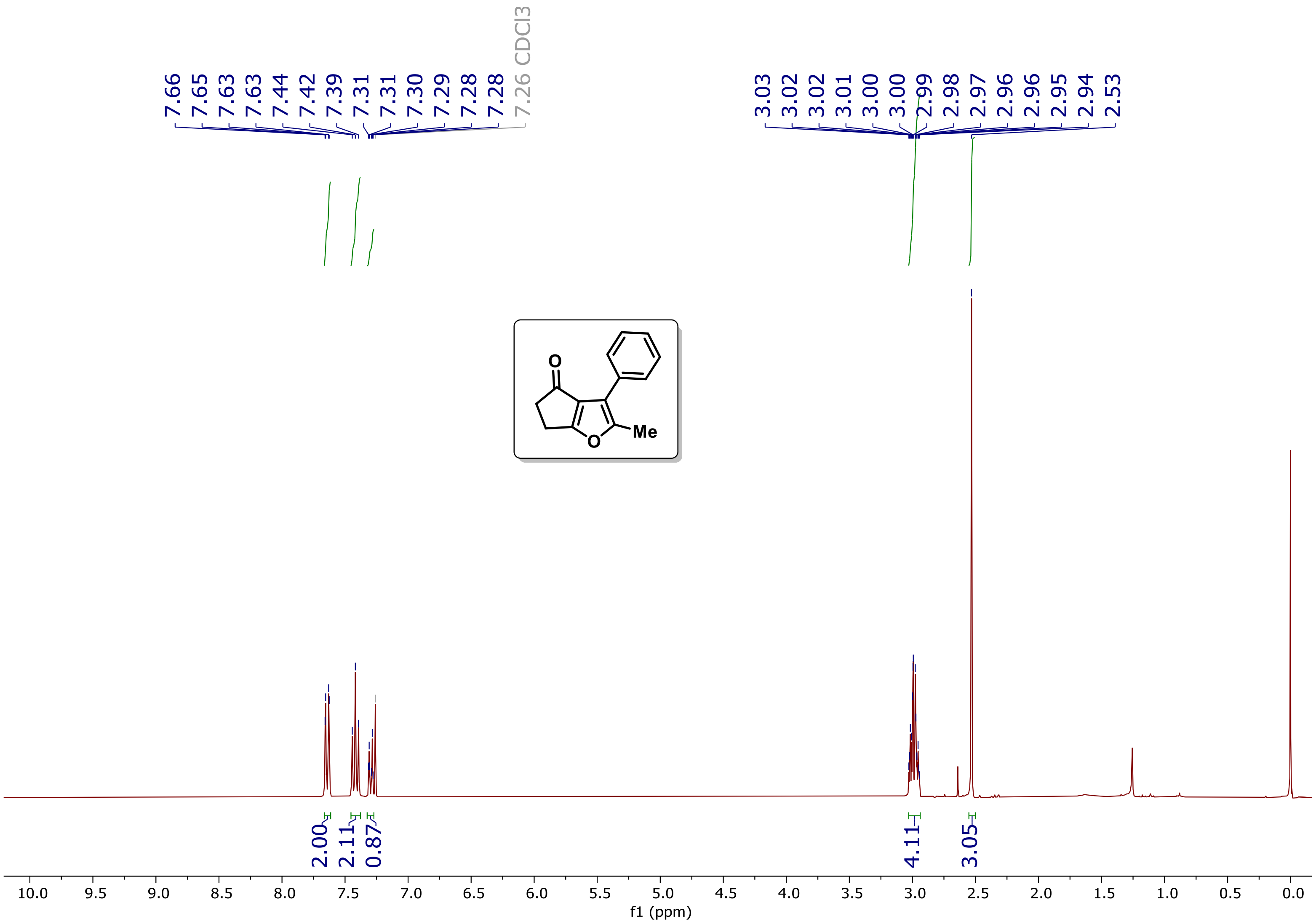

$^{13}C\{^{1}H\}$ NMR ($CDCl_3$, 75 MHz) of **25**.

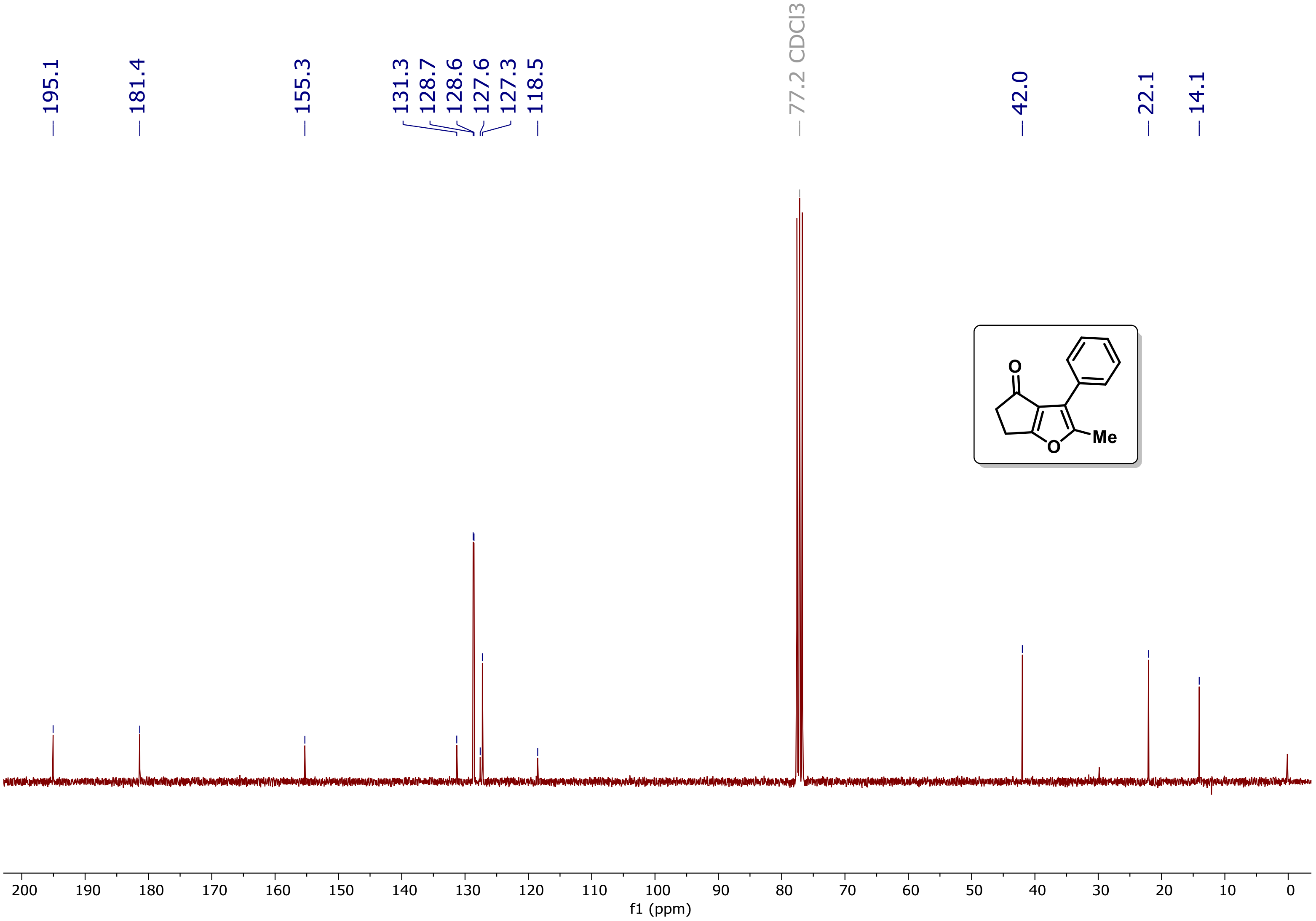

^{1}H NMR ($CDCl_3$, 400 MHz) of **26**.

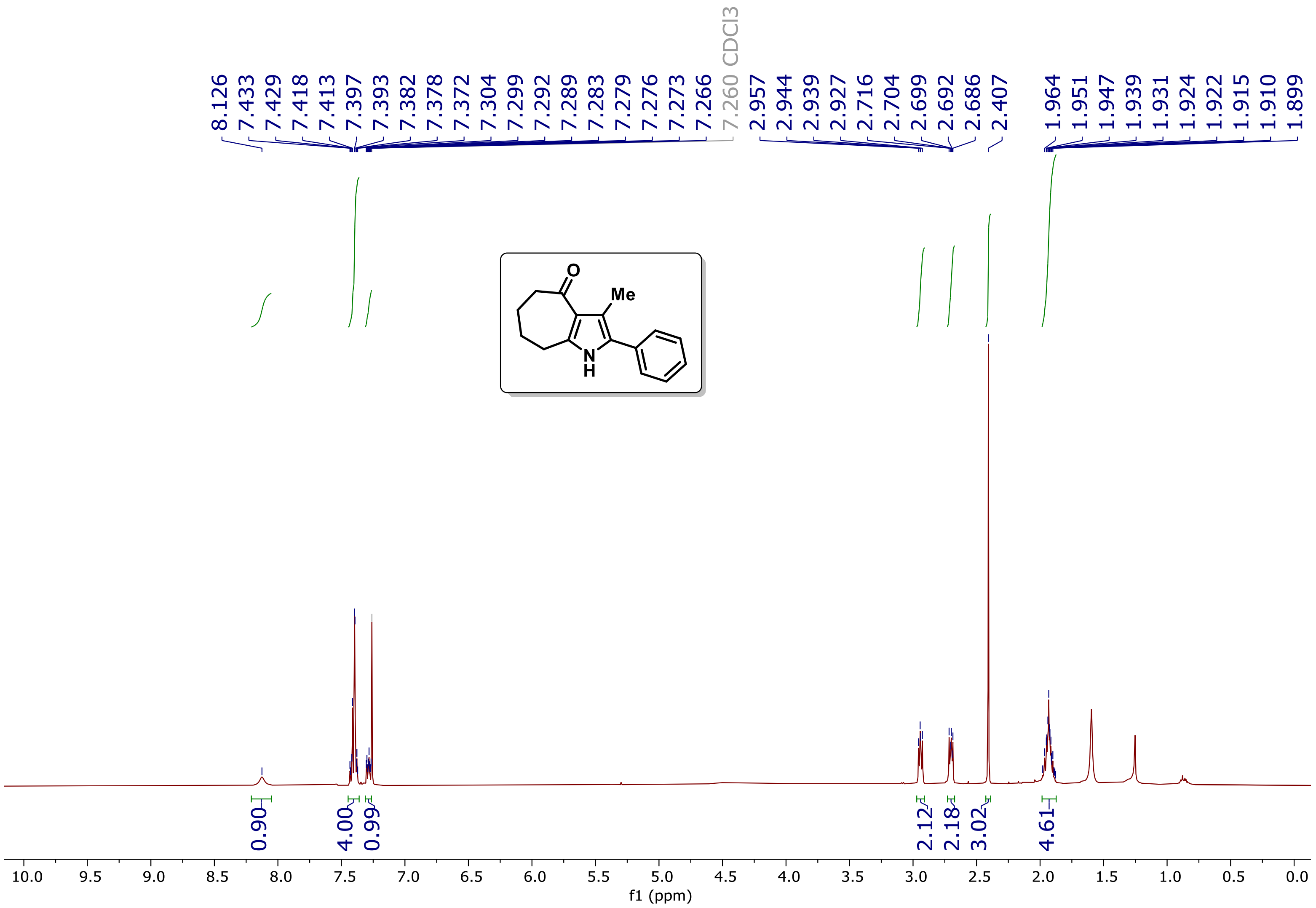

$^{13}C\{^{1}H\}$ NMR ($CDCl_3$, 100 MHz) of **26**.

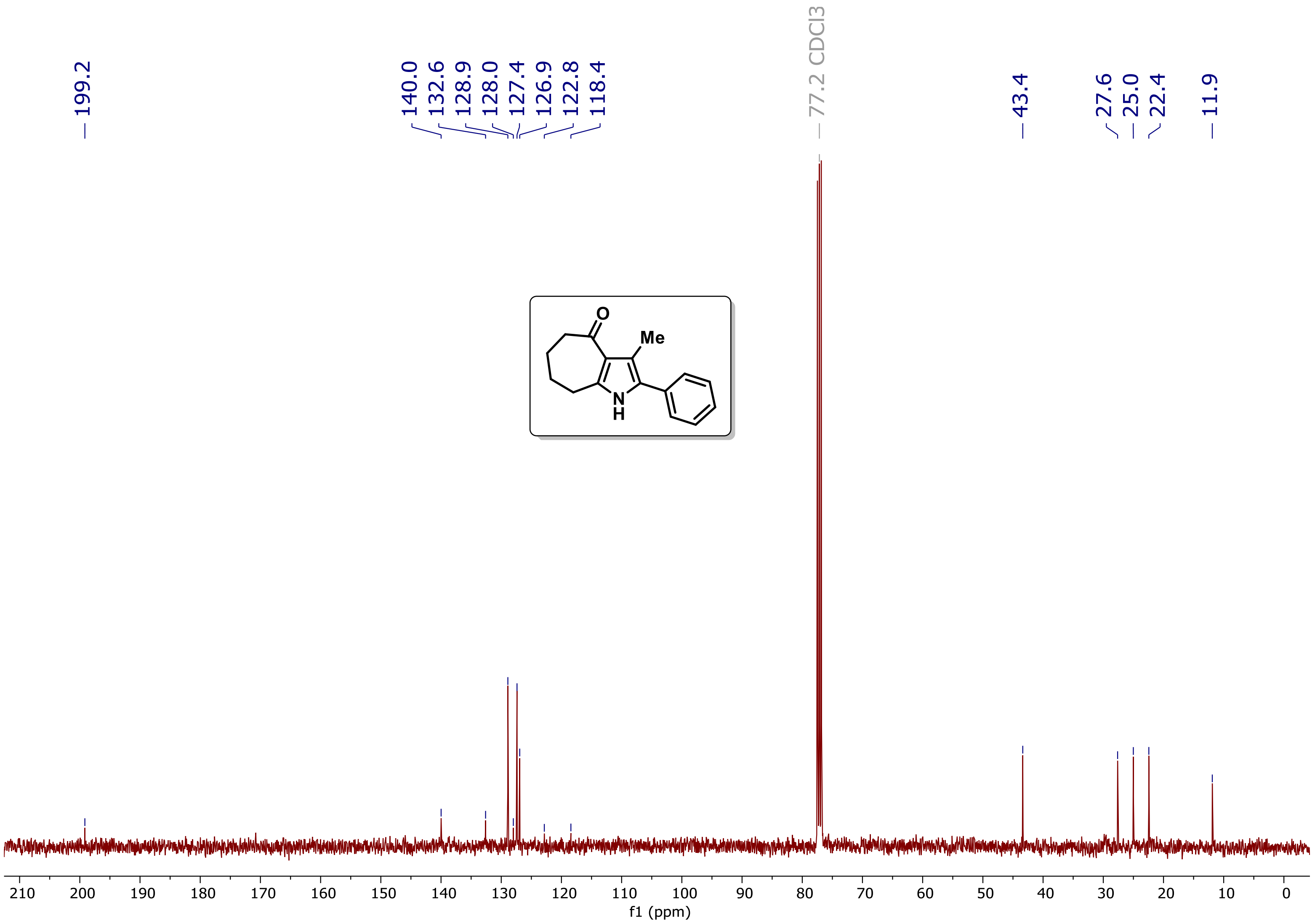

^{1}H NMR ($CDCl_3$, 300 MHz) of **27**.

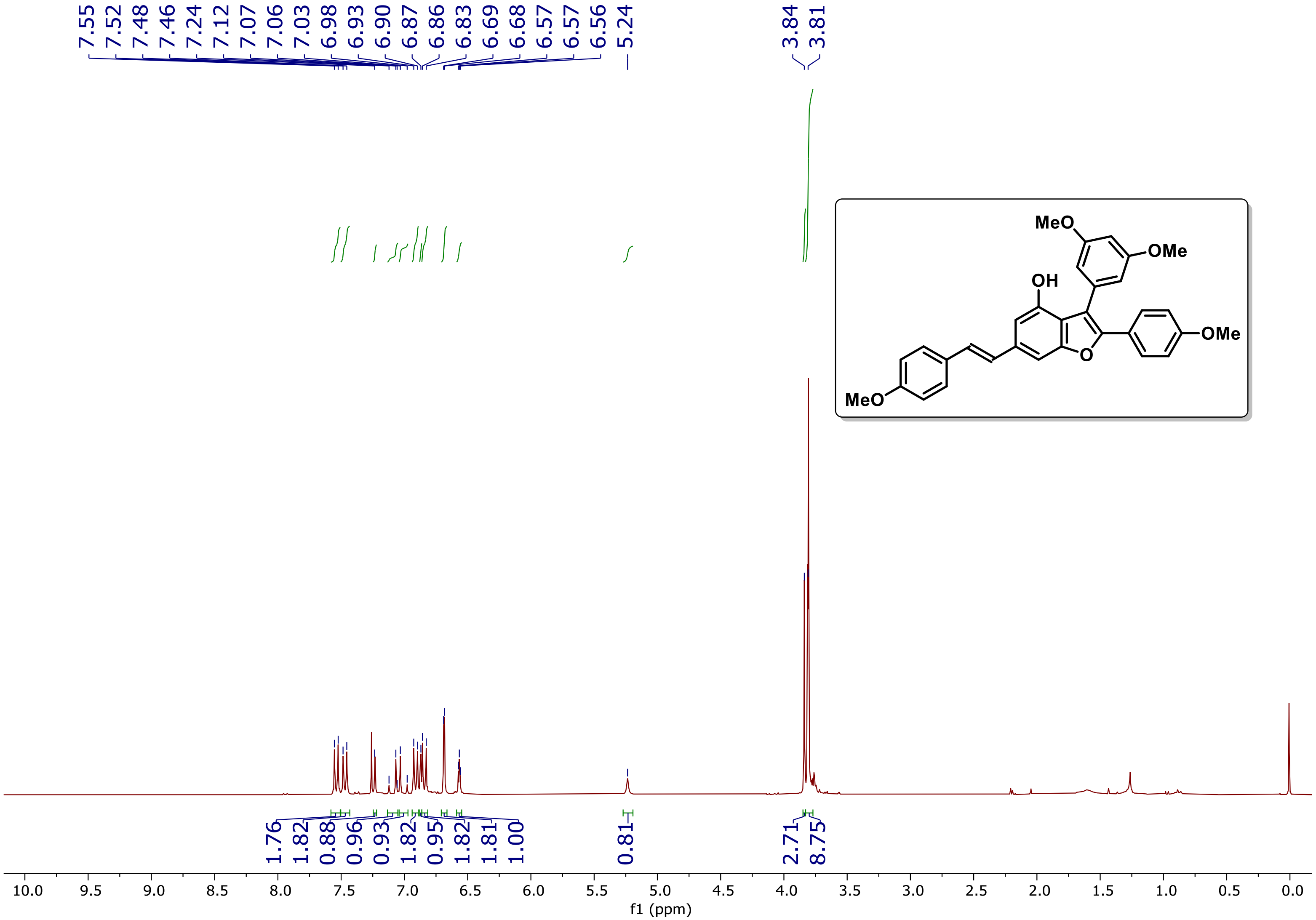

^{13}C NMR (CDCl$_3$, 75 MHz) of **27**.

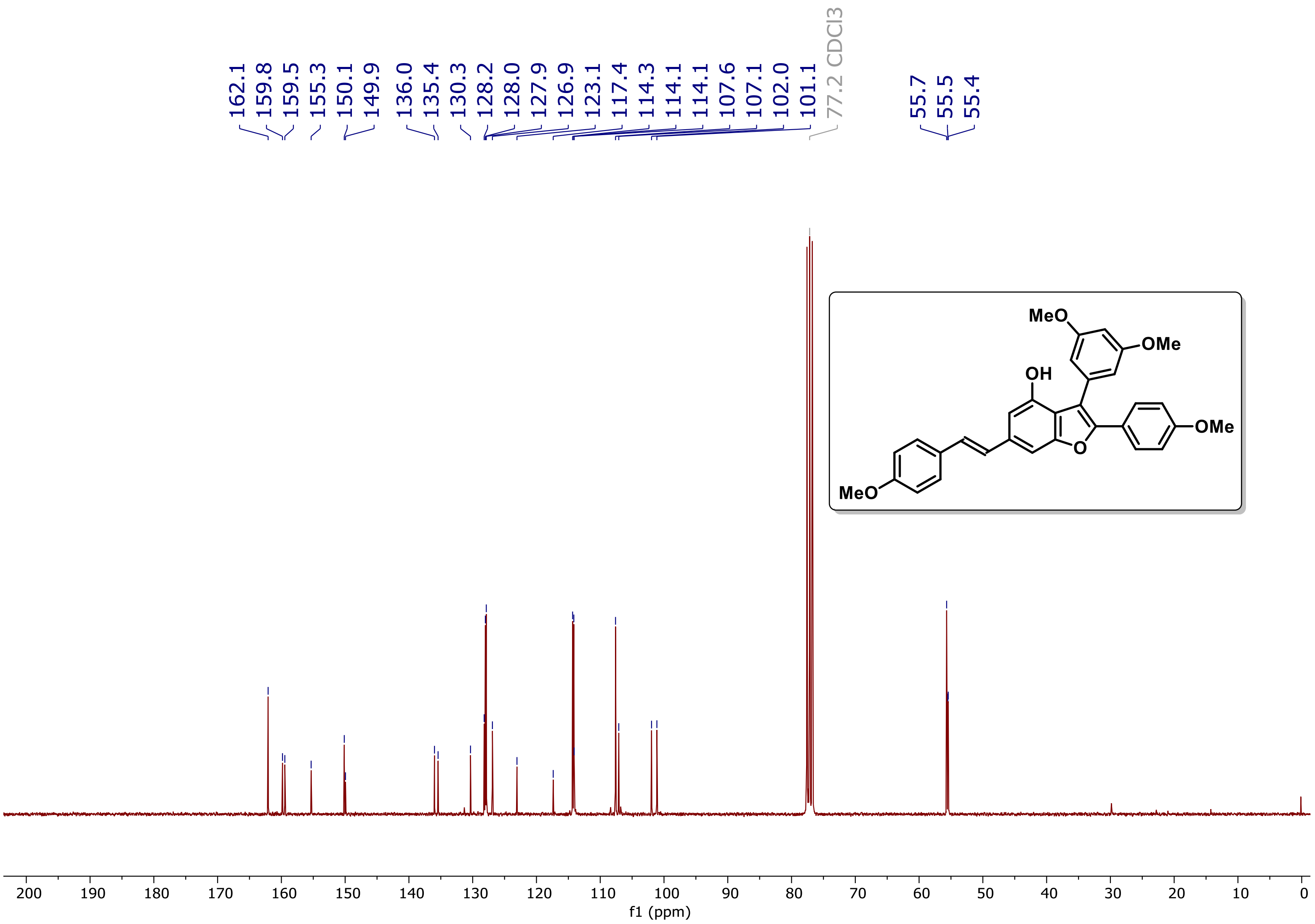

^{1}H NMR ($(CD_3)_2CO$, 400 MHz) of **6**.

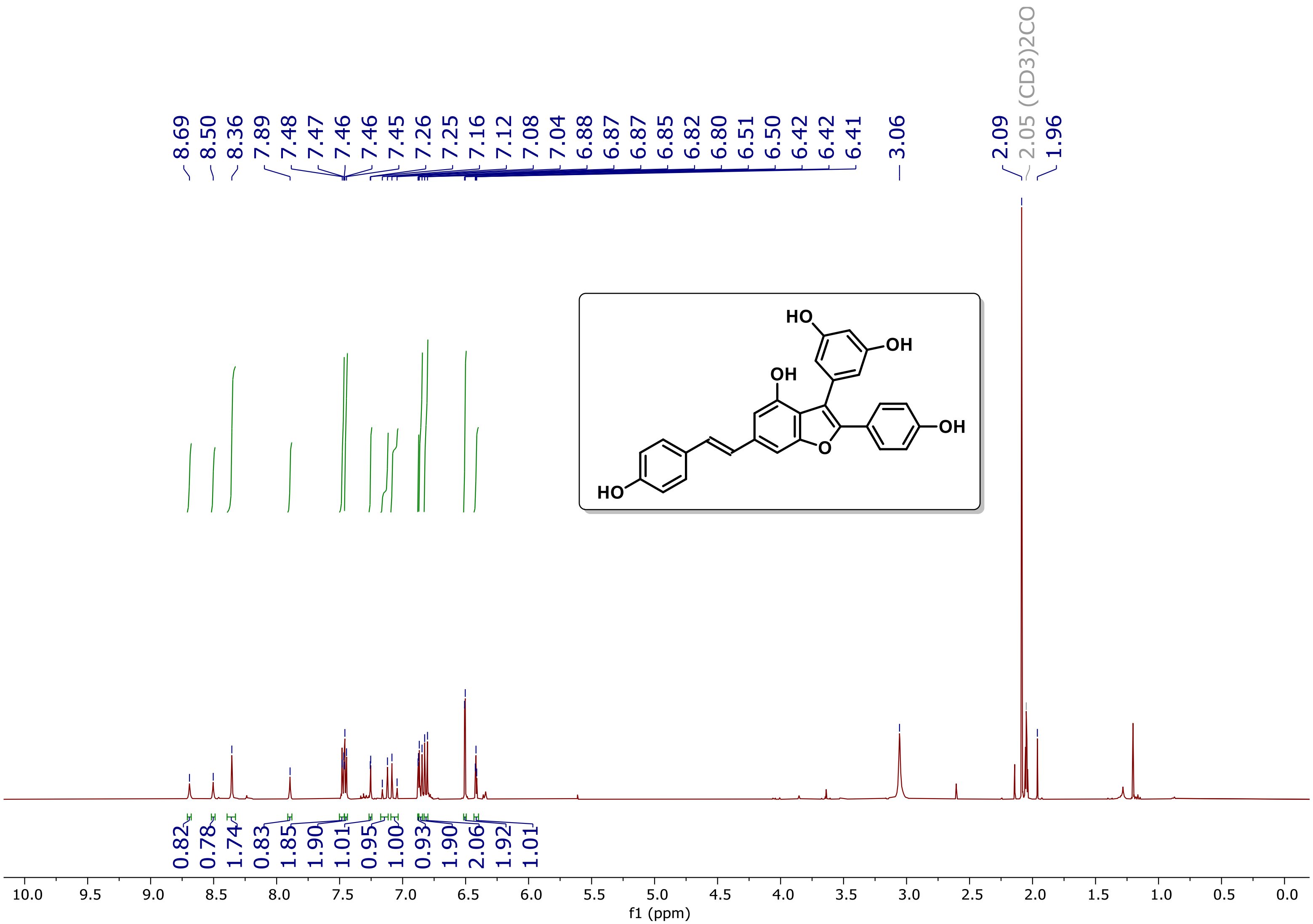

^{13}C NMR ($(CD_3)_2CO$, 100 MHz) of **6**.

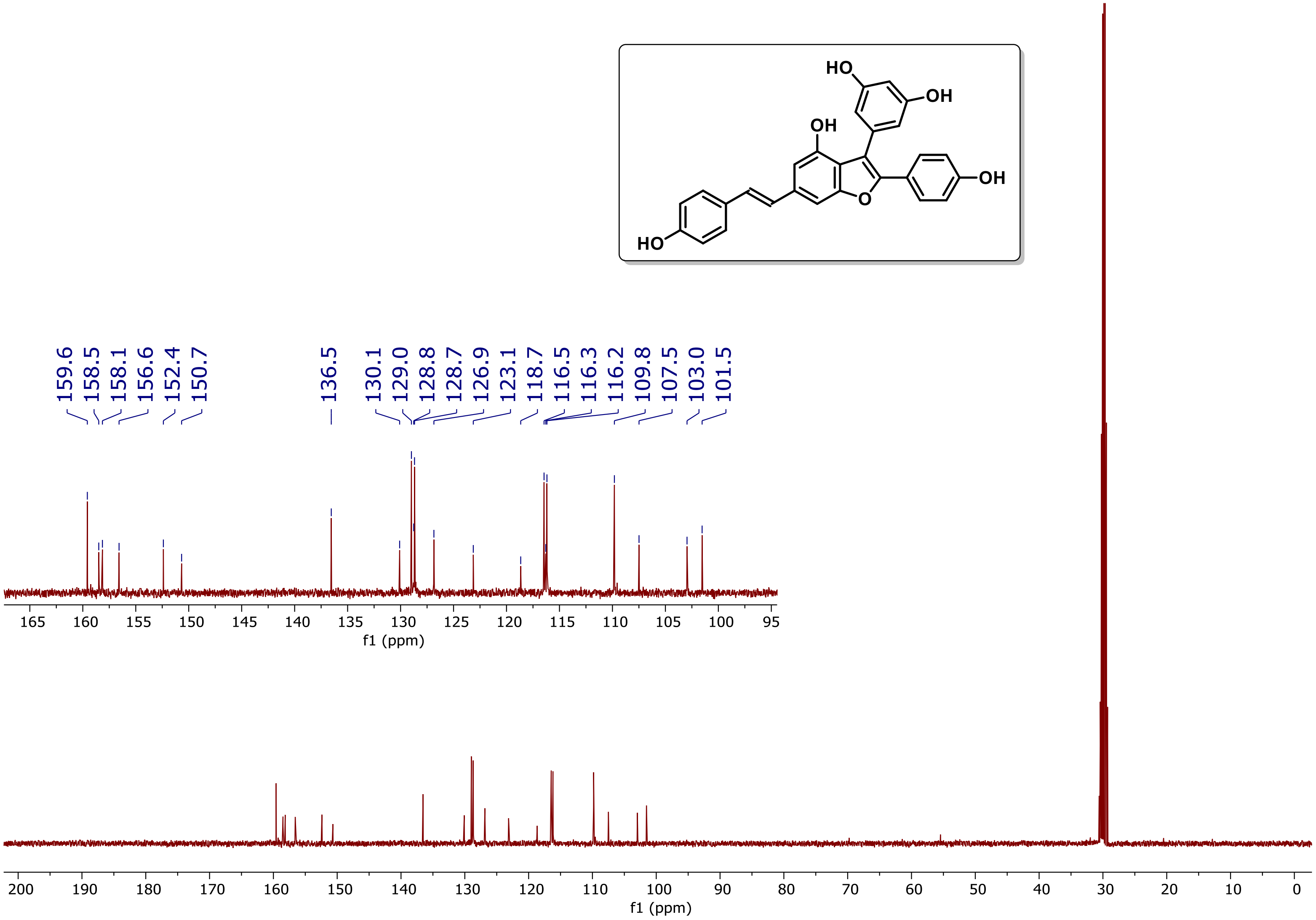

^{1}H NMR ($CDCl_3$, 300 MHz) of **S14**.

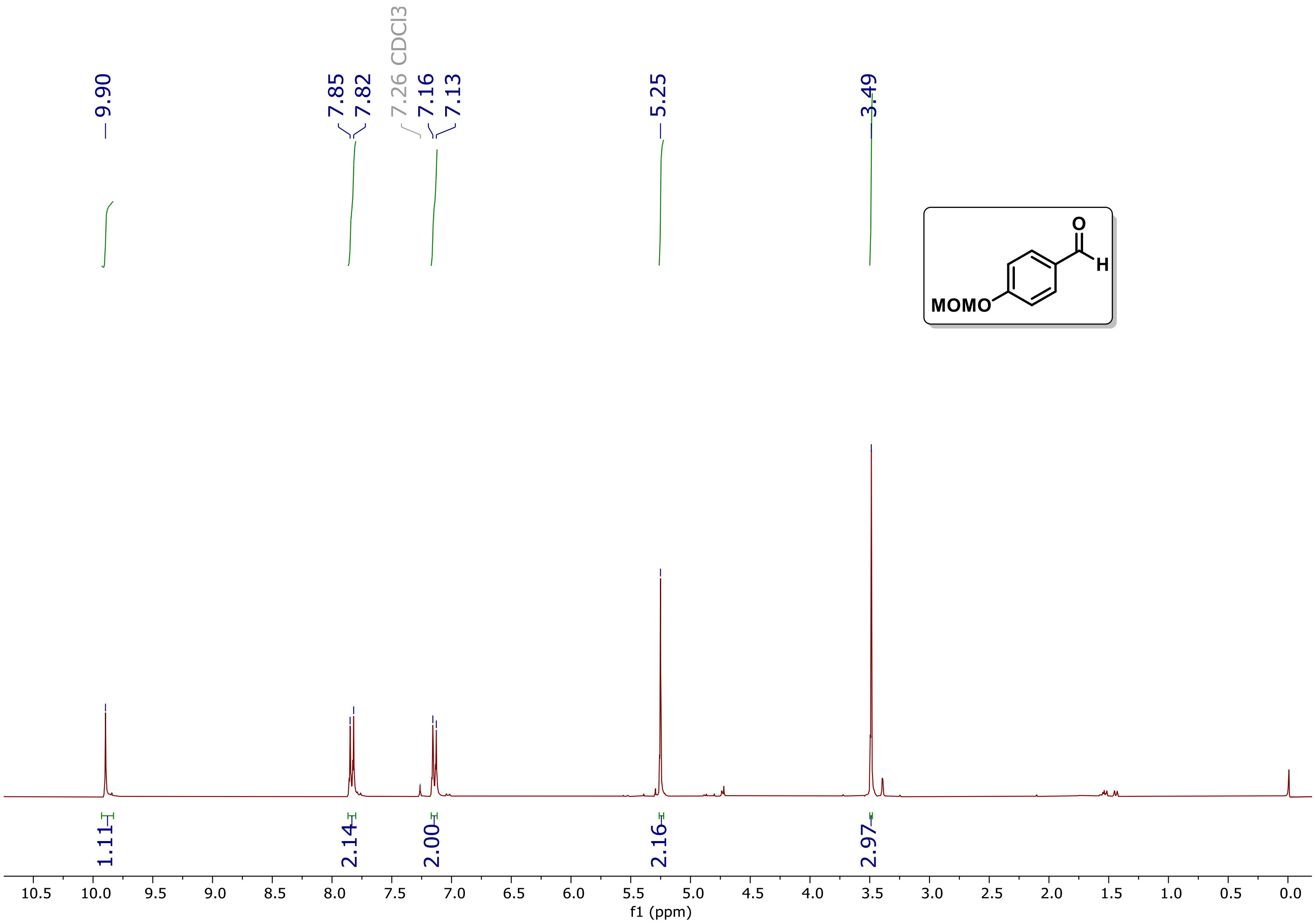

^{1}H NMR ($CDCl_3$, 300 MHz) of **S15**.

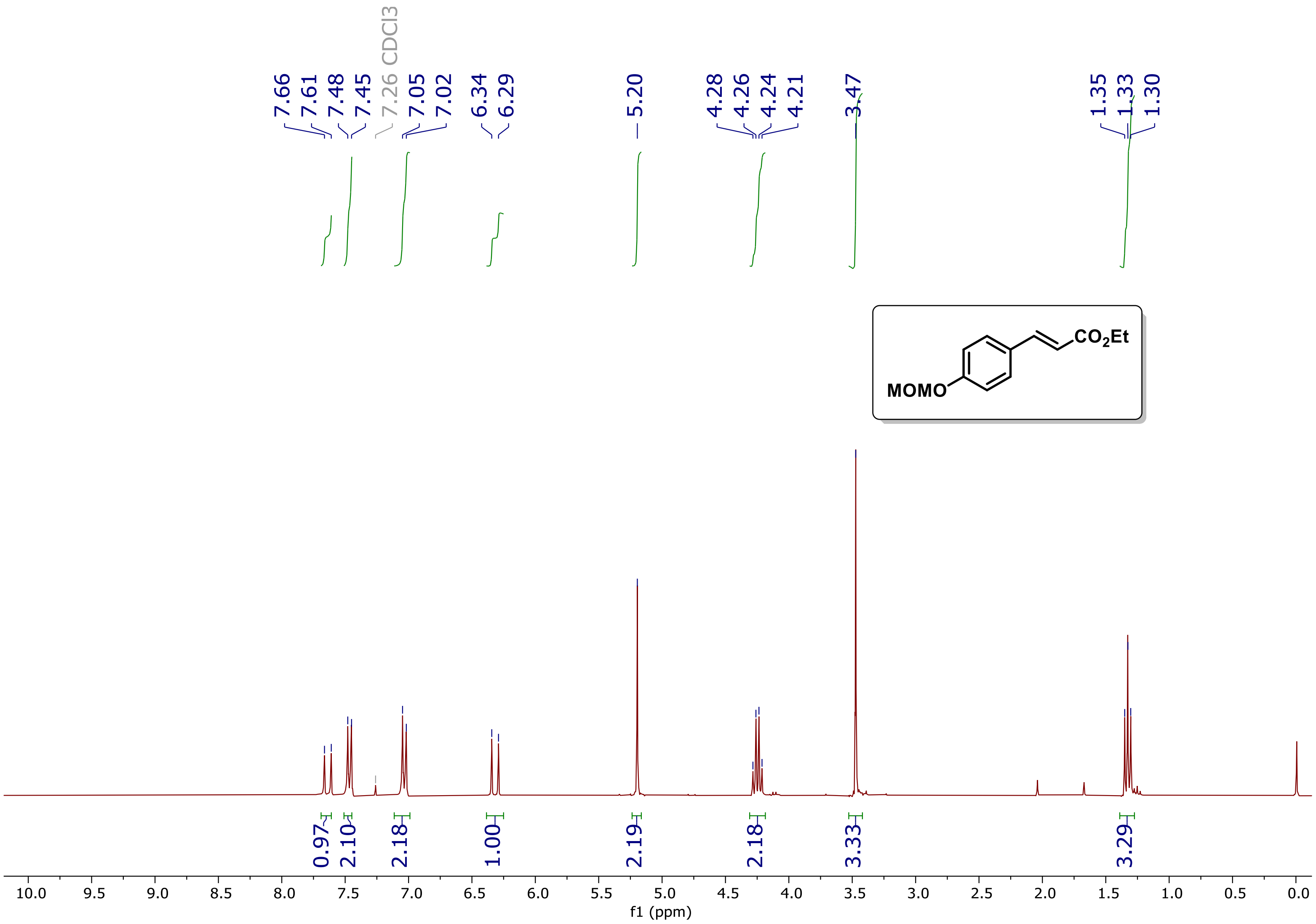

^{1}H NMR ($CDCl_3$, 300 MHz) of **29**.

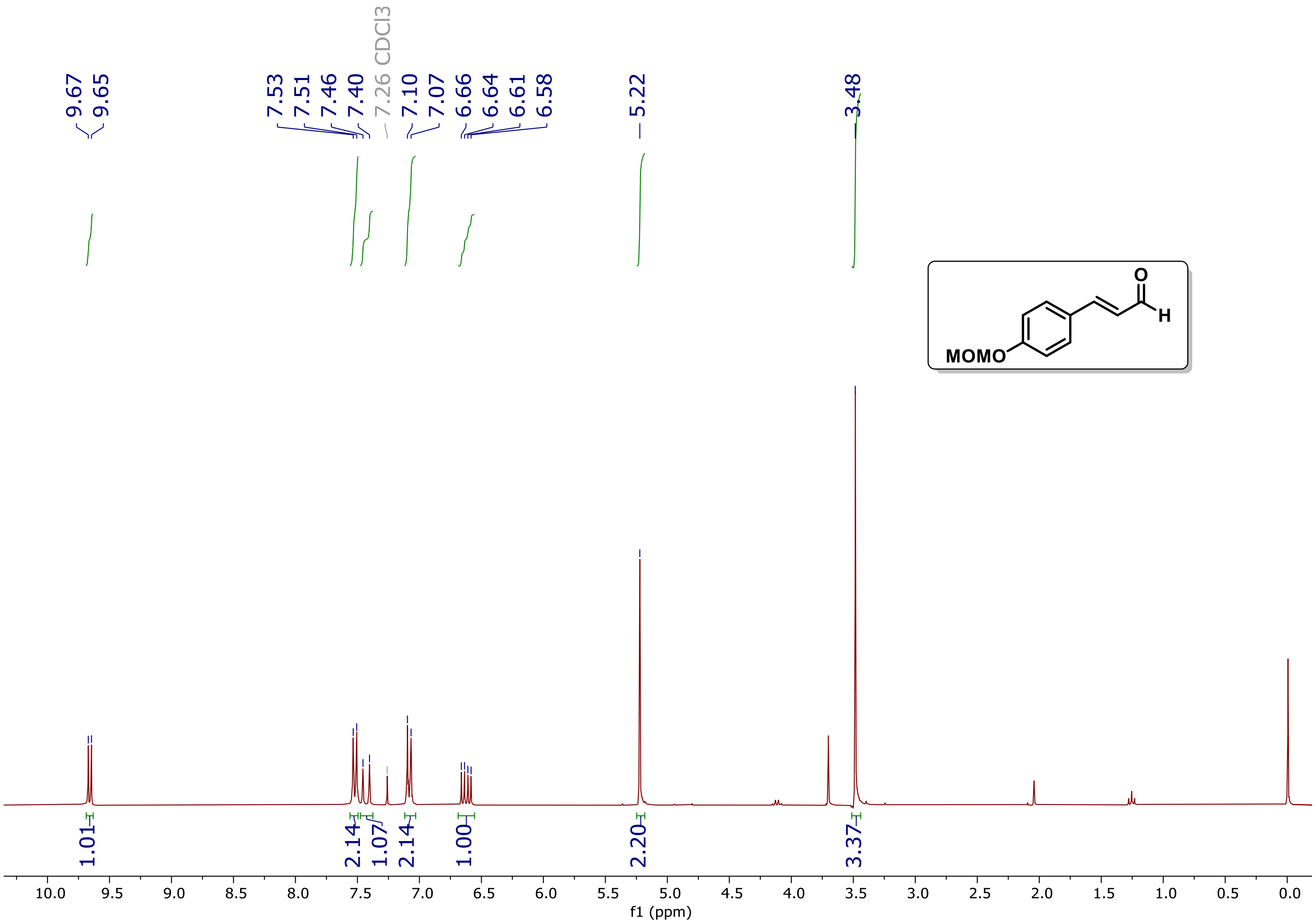

^{1}H NMR ($CDCl_3$, 300 MHz) of **19a**.

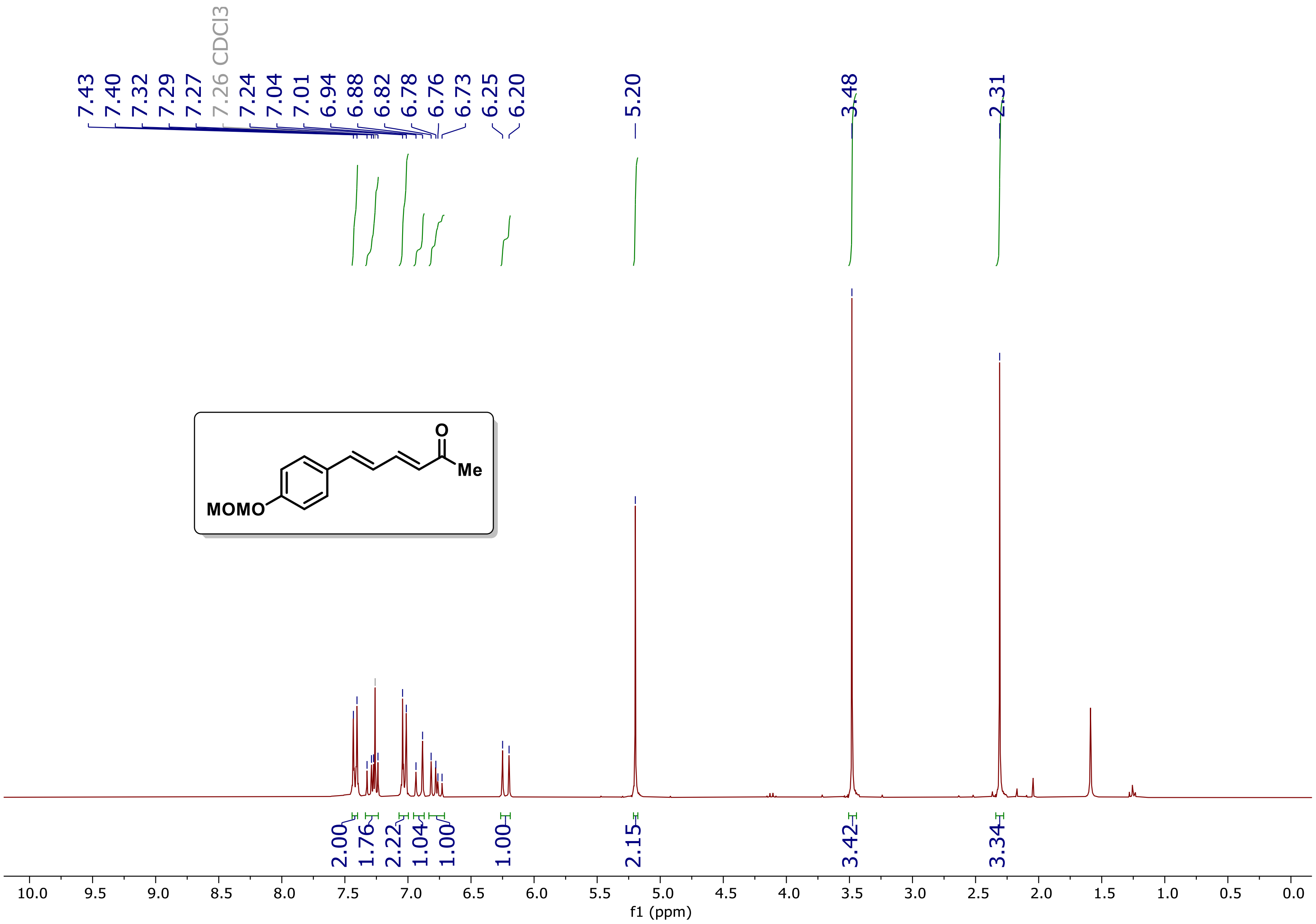

^{1}H NMR ($CDCl_3$, 300 MHz) of **12a**.

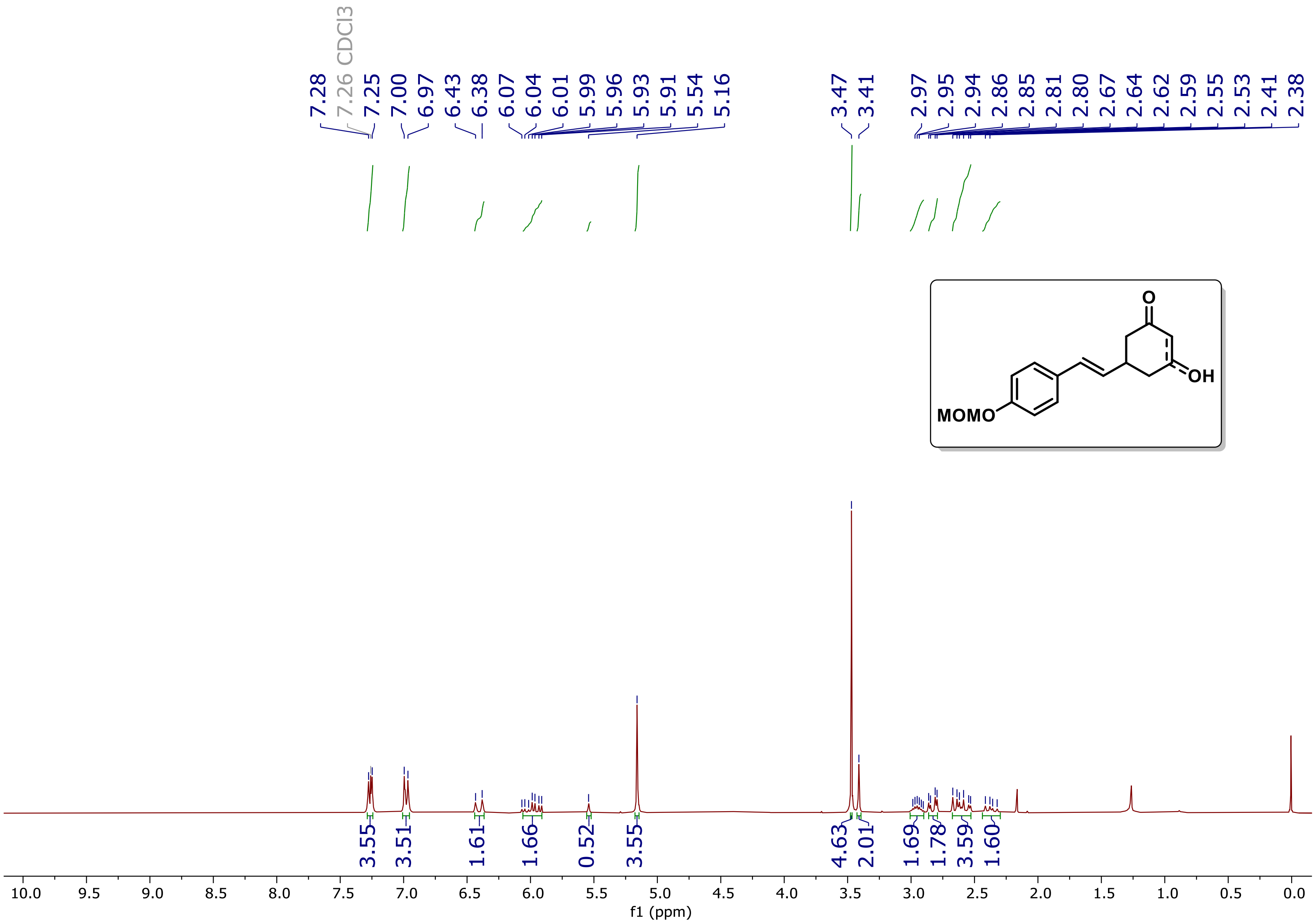

$^{13}C\{^1H\}$ NMR ($CDCl_3$, 75 MHz) of **12a**.

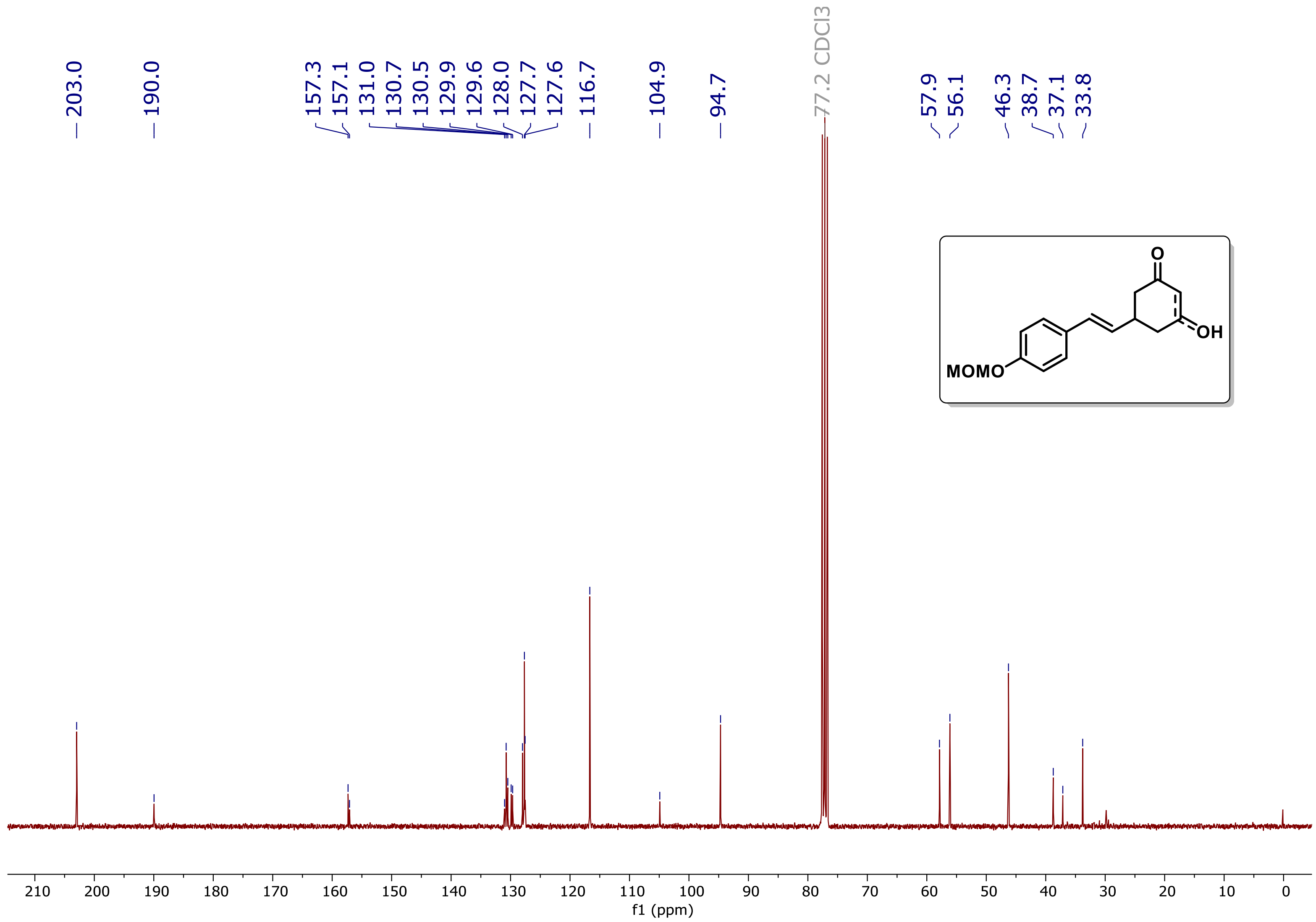

^{1}H NMR ($CDCl_3$, 300 MHz) of **31**.

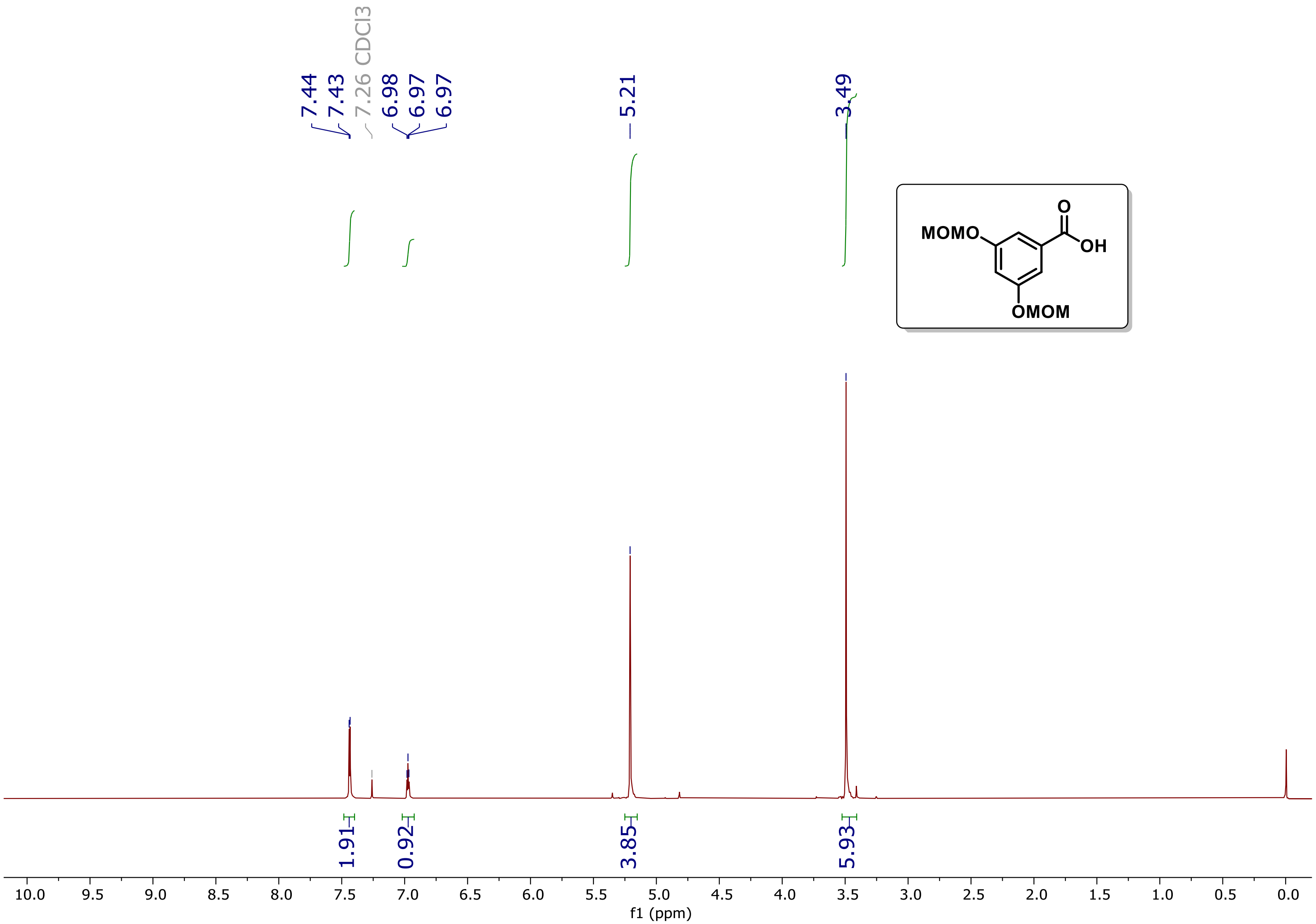

^{1}H NMR (CD_3OD, 300 MHz) of **S17**.

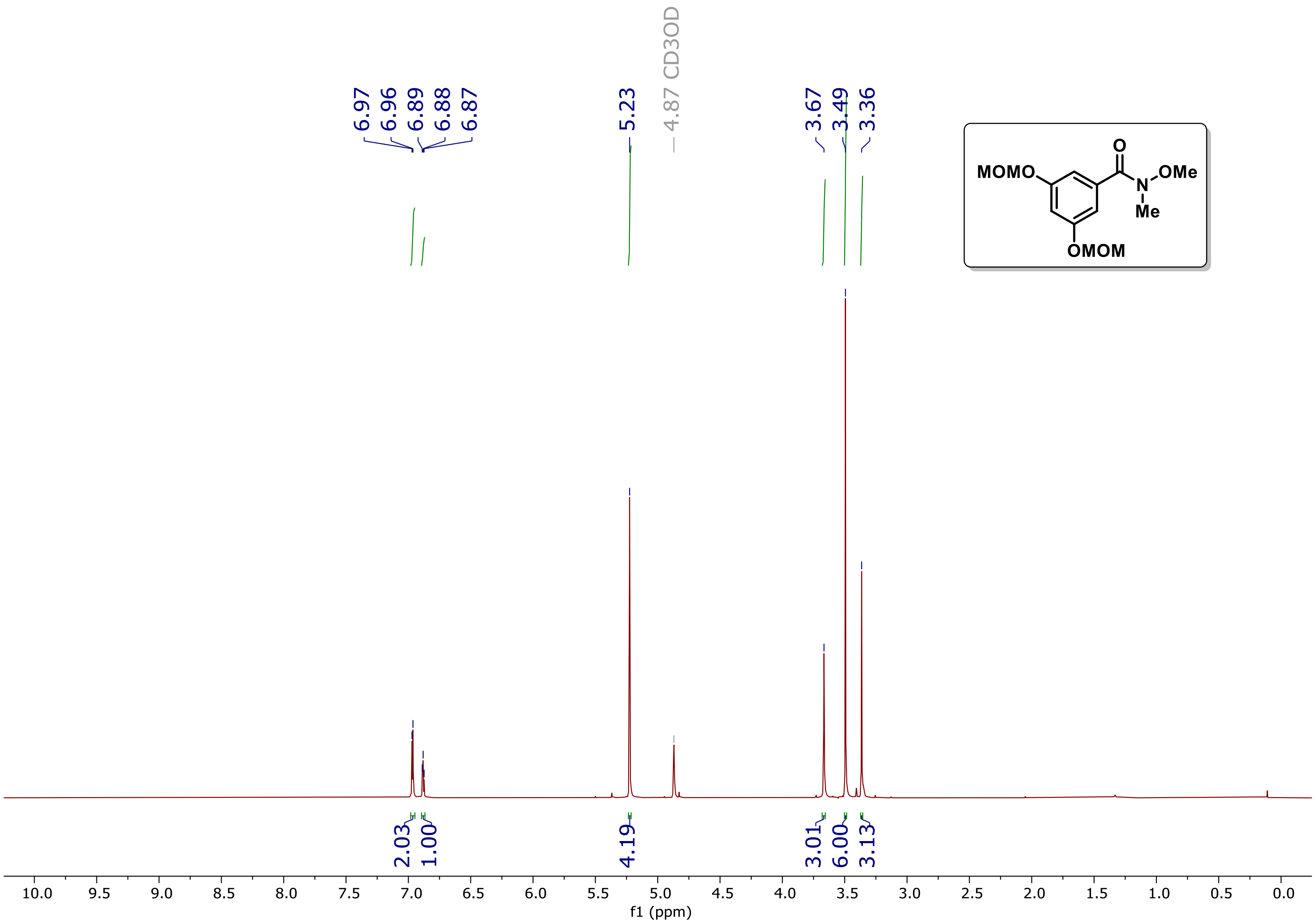

$^{13}C\{^{1}H\}$ NMR (CD_3OD, 75 MHz) of **S17**.

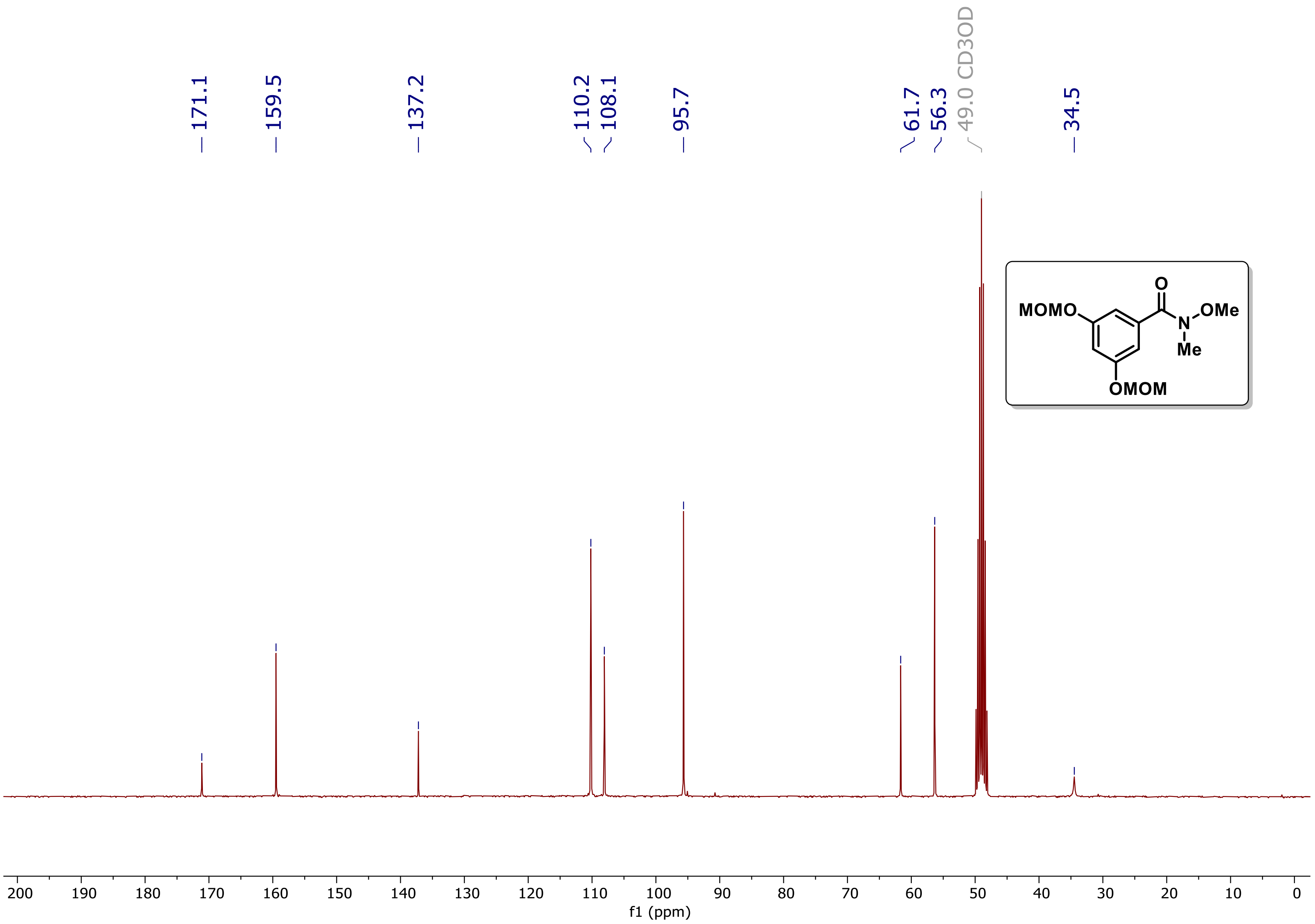

^{1}H NMR ($CDCl_3$, 300 MHz) of **21a**.

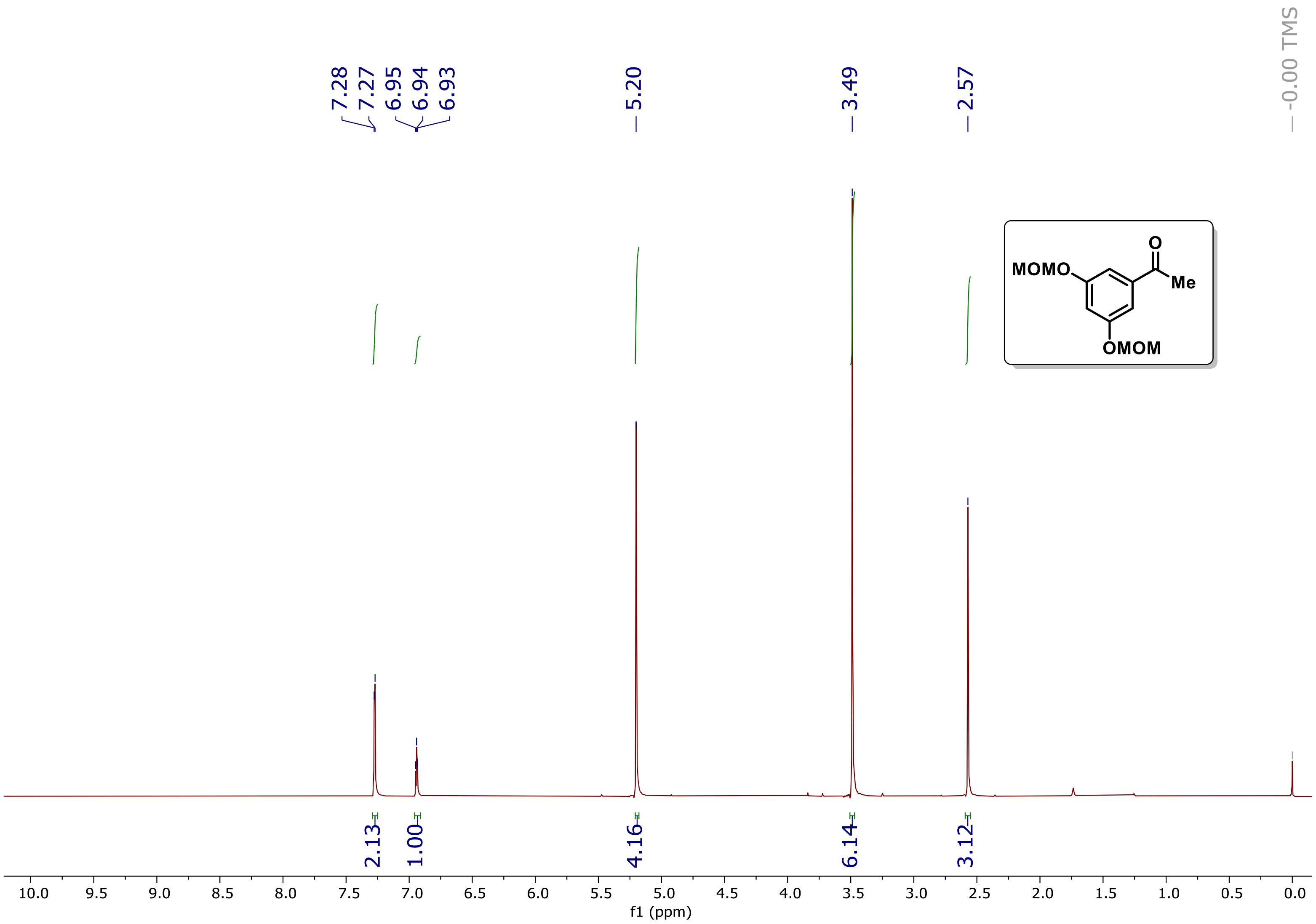

$^{13}C\{^{1}H\}$ NMR ($CDCl_3$, 75 MHz) of **21a**.

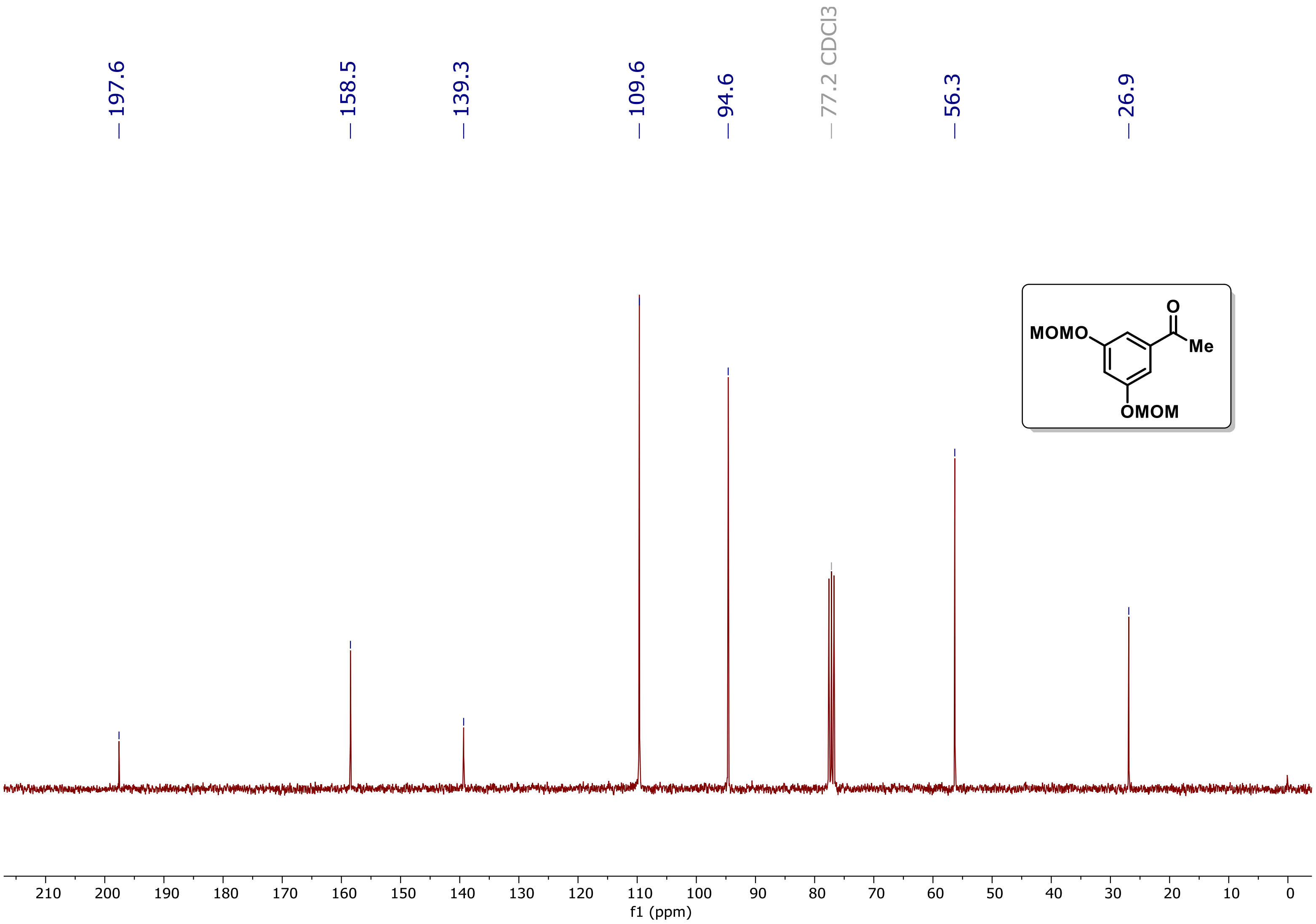

^{1}H NMR ($CDCl_3$, 300 MHz) of **13a**.

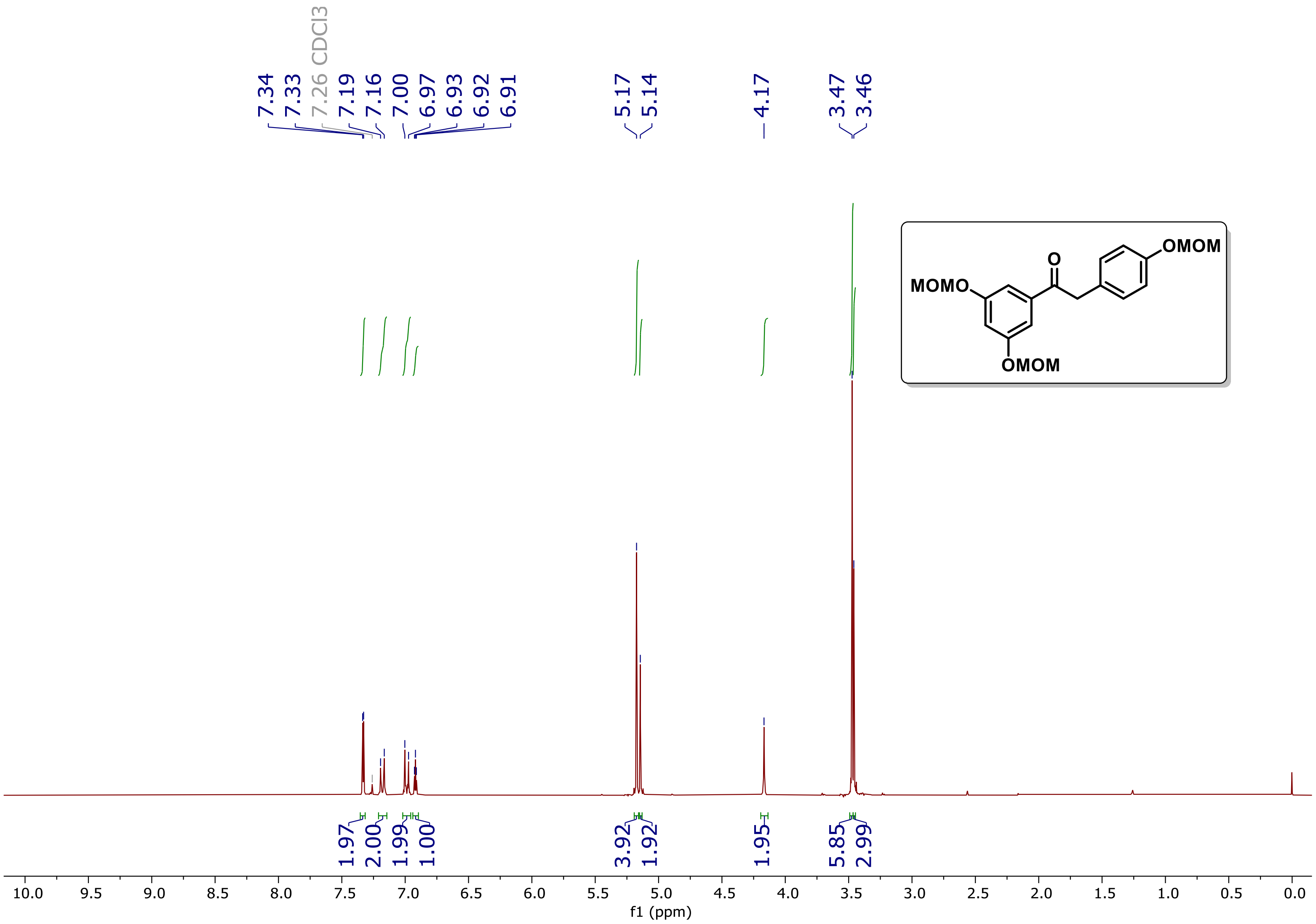

$^{13}C\{^{1}H\}$ NMR ($CDCl_3$, 75 MHz) of **13a**.

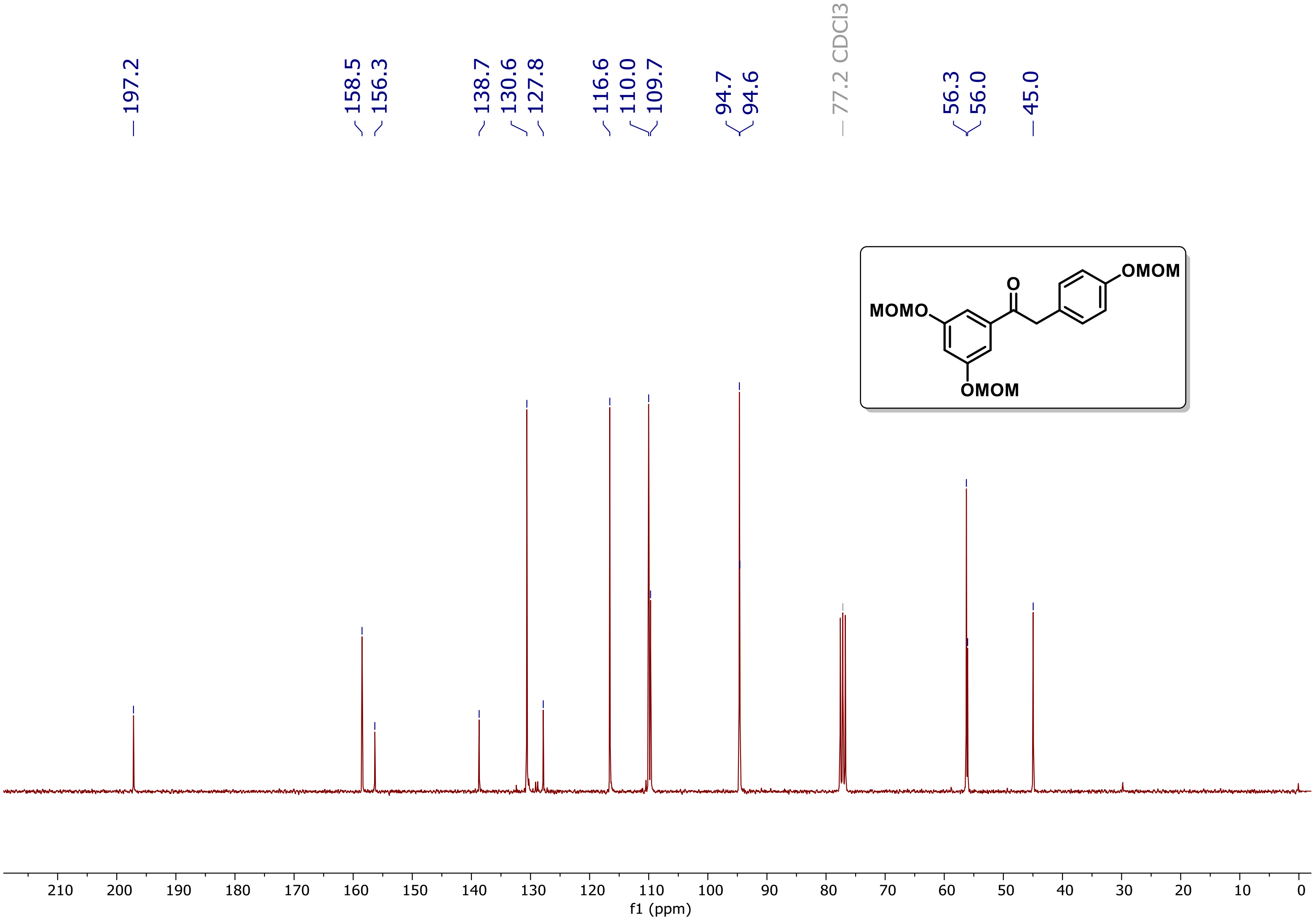

^{1}H NMR ($CDCl_3$, 300 MHz) of **S10o**.

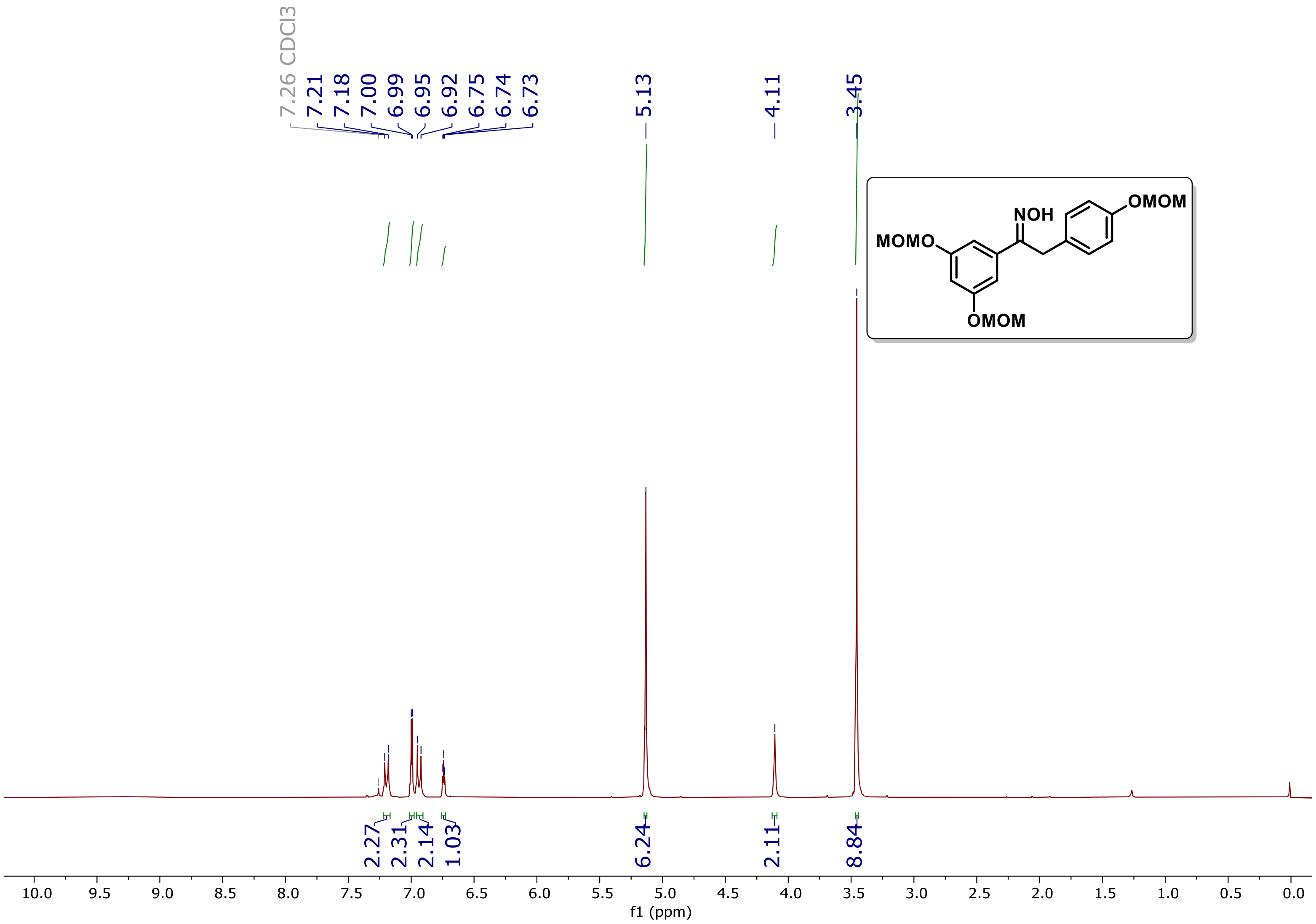

$^{13}C\{^{1}H\}$ NMR ($CDCl_3$, 75 MHz) of **S10o**.

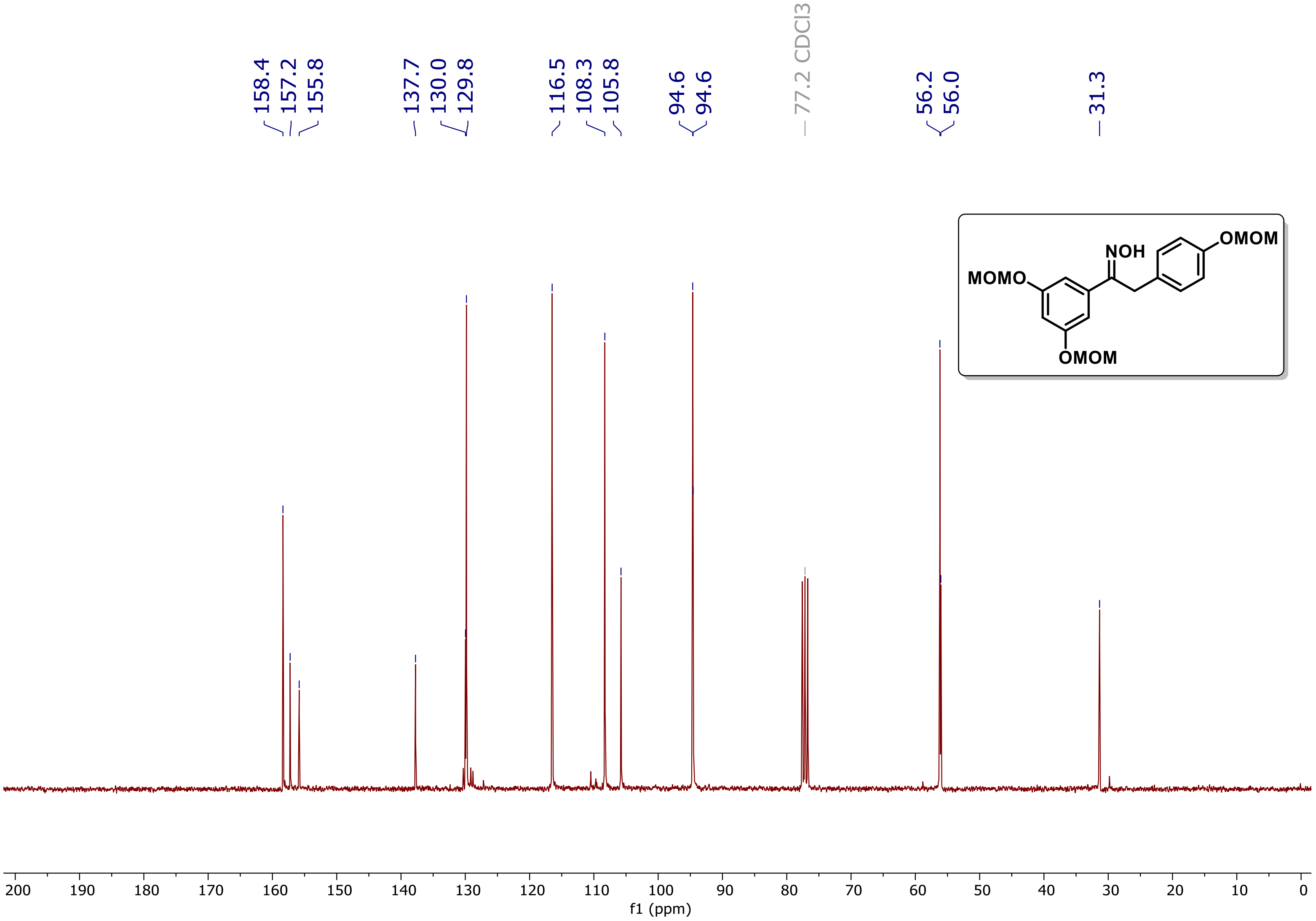

^{1}H NMR ($CDCl_3$, 300 MHz) of **11a**.

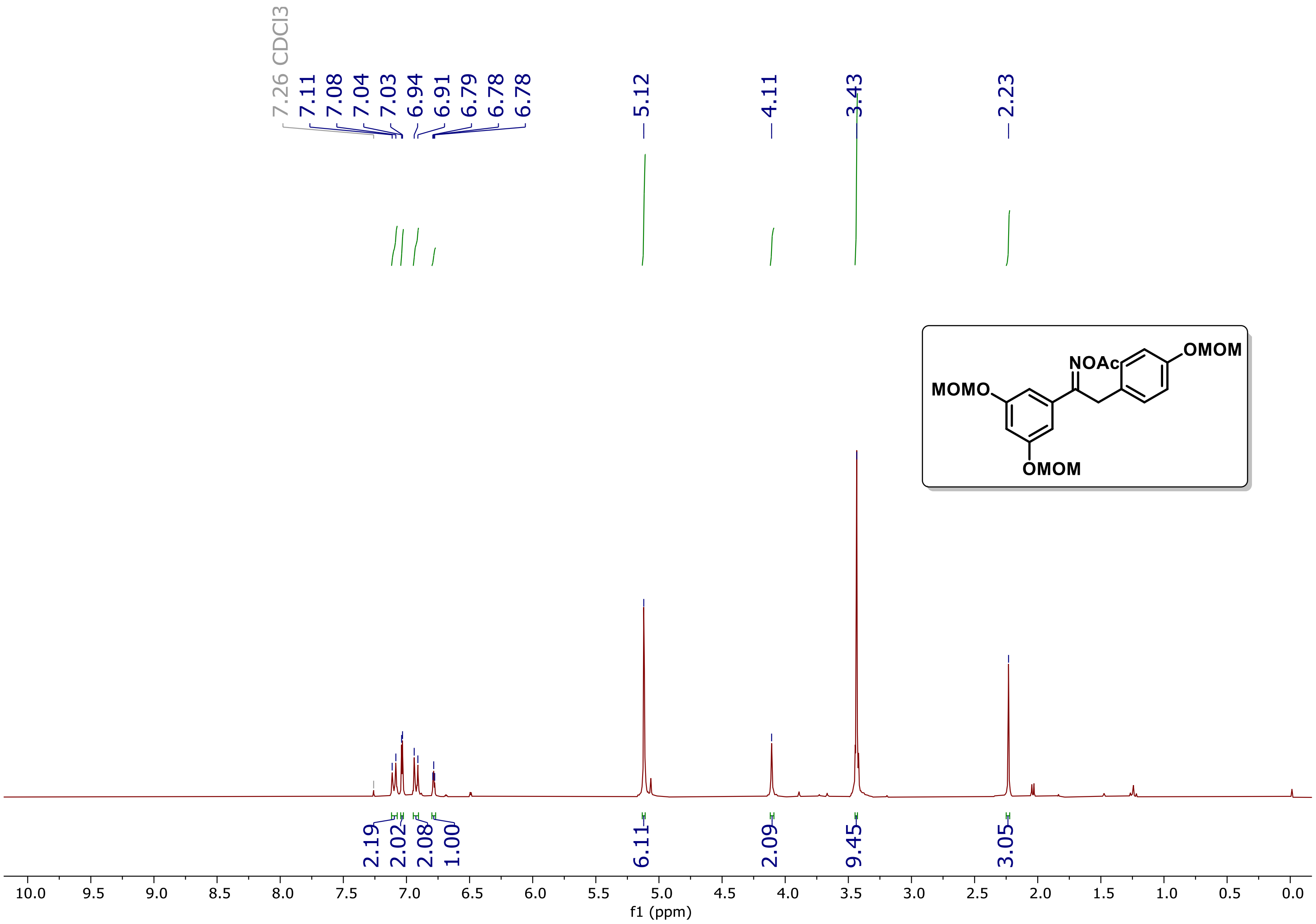

$^{13}C\{^1H\}$ NMR ($CDCl_3$, 75 MHz) of **11a**.

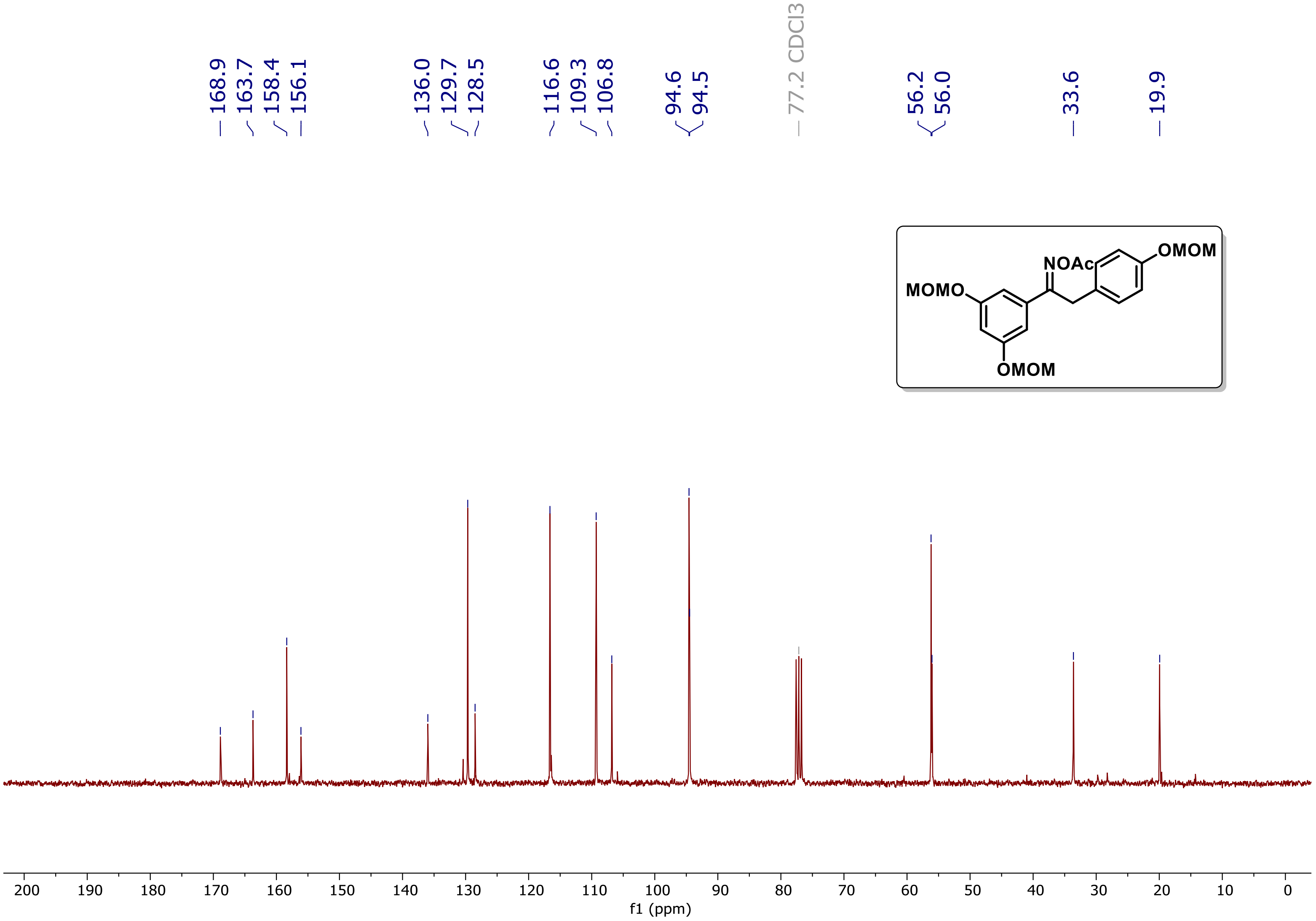

^{1}H NMR ($CDCl_3$, 300 MHz) of **10a**.

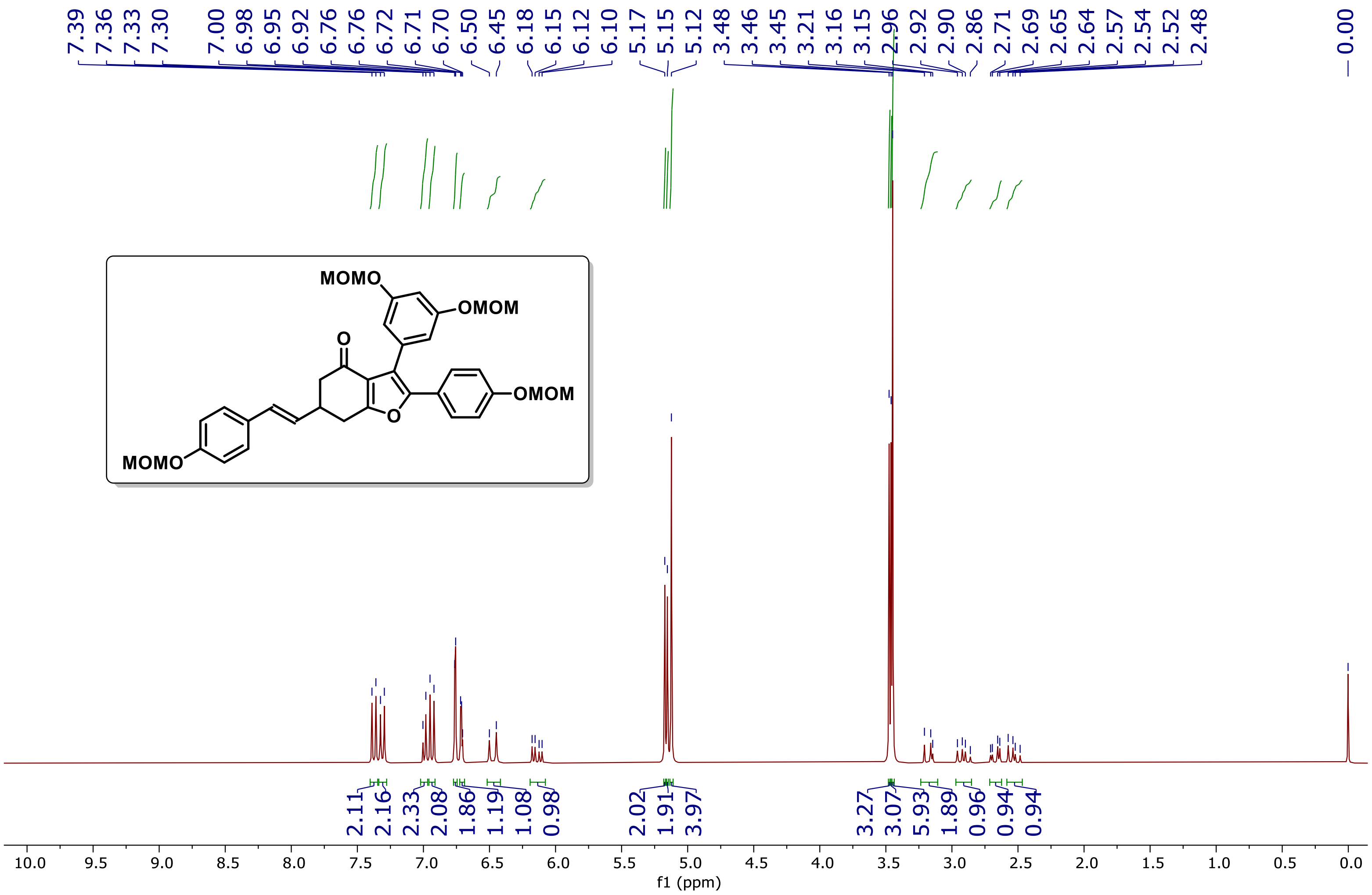

$^{13}C\{^{1}H\}$ NMR ($CDCl_3$, 75 MHz) of **10a**.

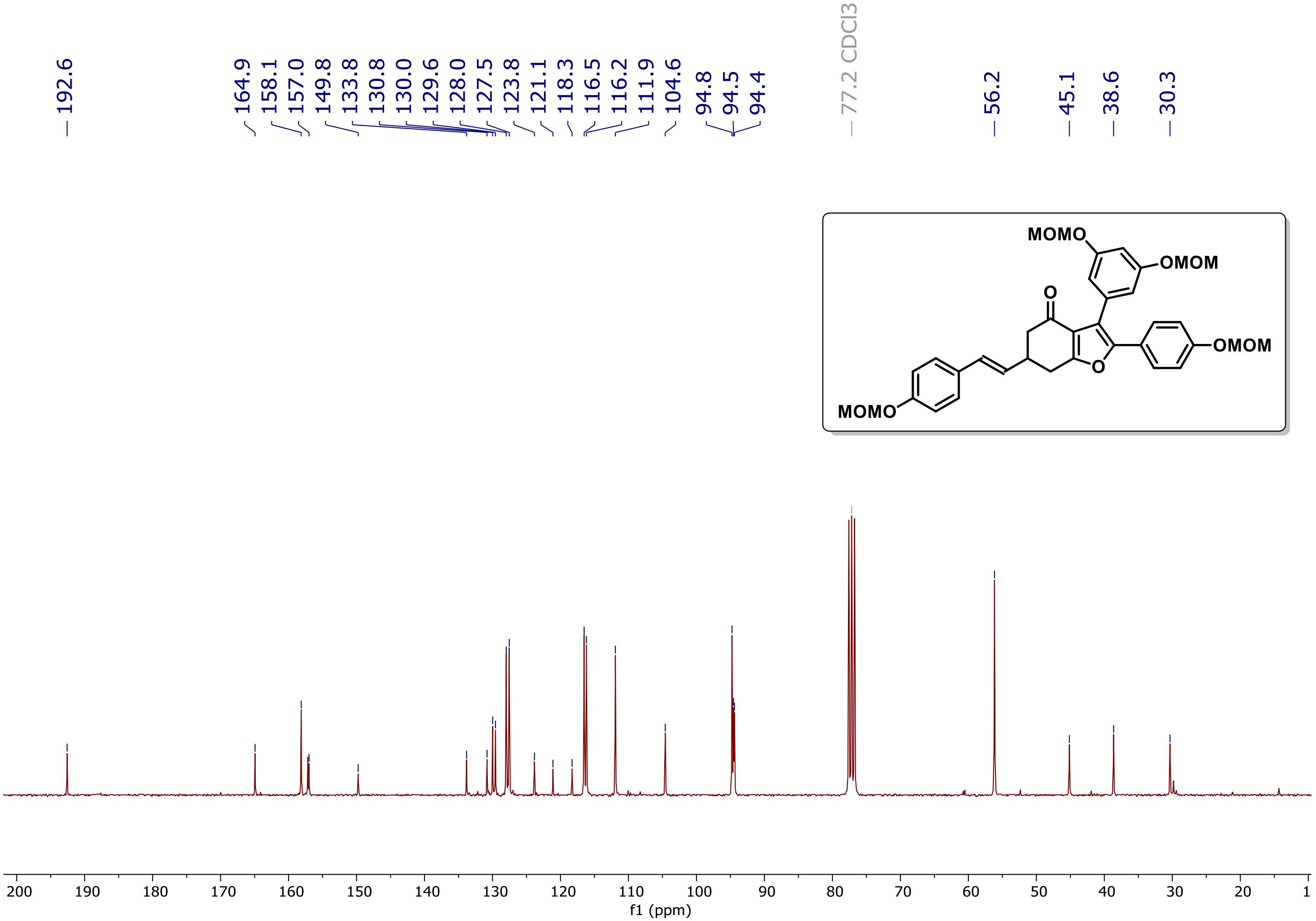

^{1}H NMR ($CDCl_3$, 300 MHz) of **27a**.

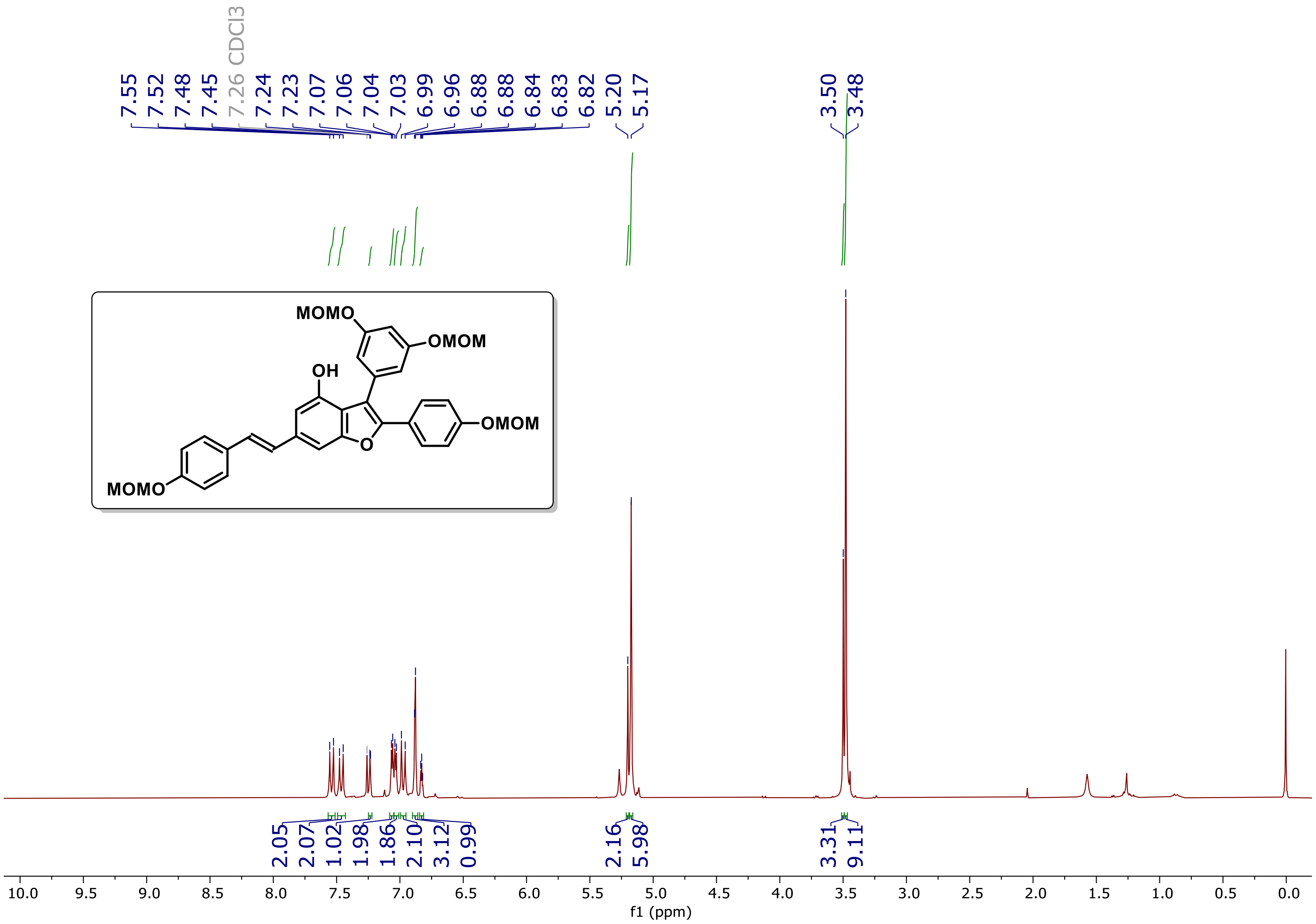

$^{13}C\{^{1}H\}$ NMR ($CDCl_3$, 75 MHz) of **27a**.

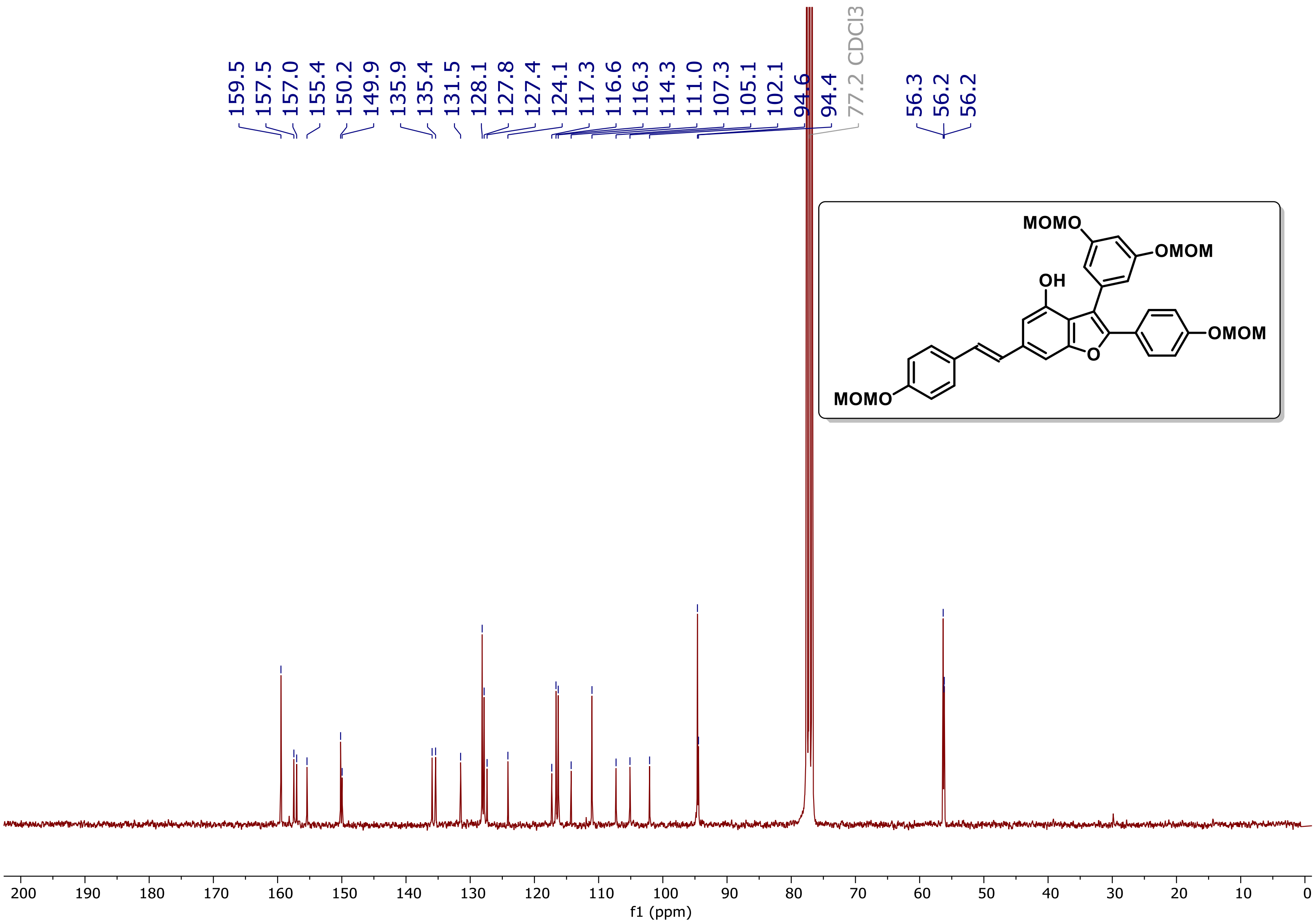

^{1}H NMR ($(CD_3)_2CO$, 700 MHz) of **7**.

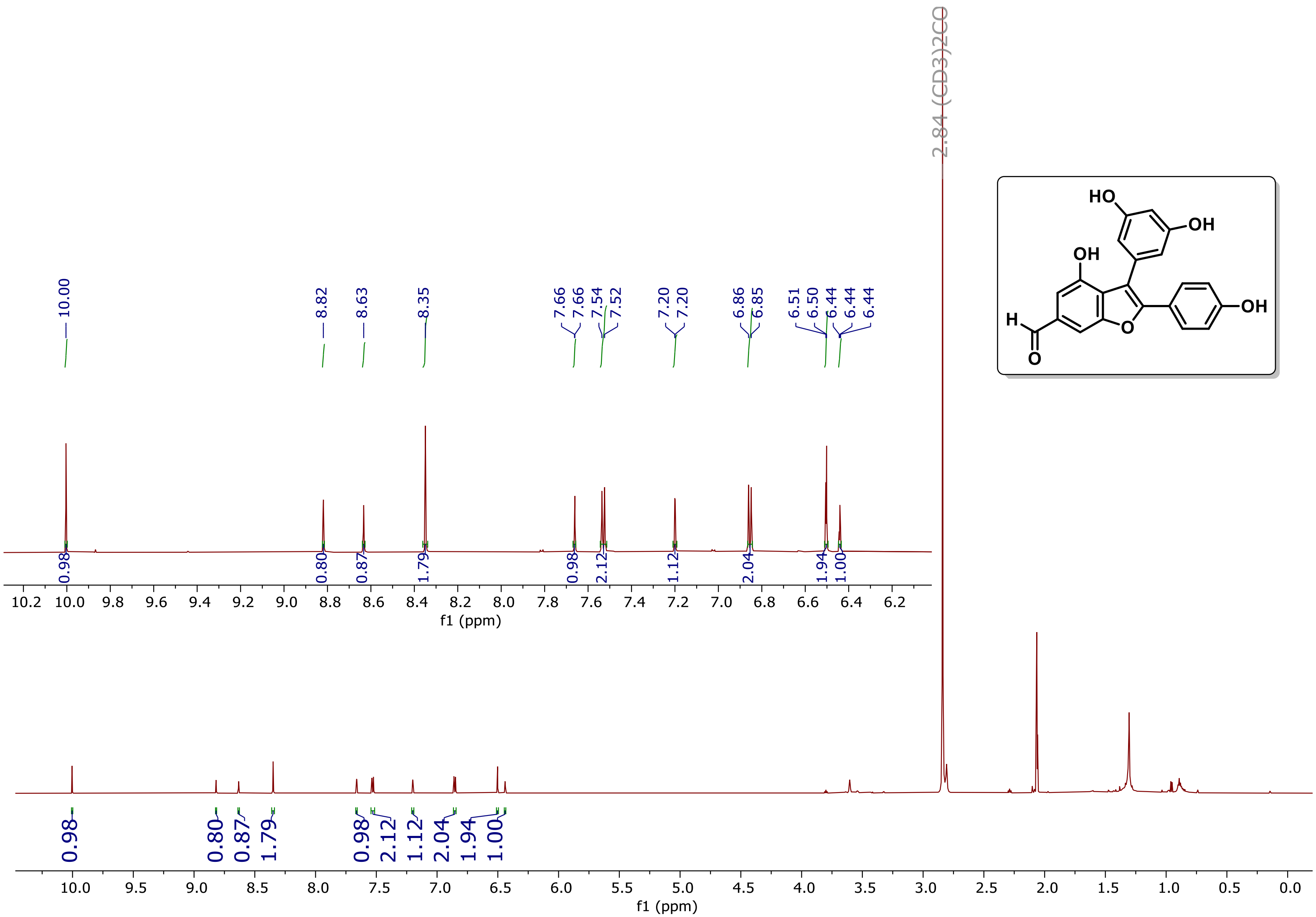

$^{13}C\{^{1}H\}$ NMR ($CDCl_3$, 175 MHz) of **7**.

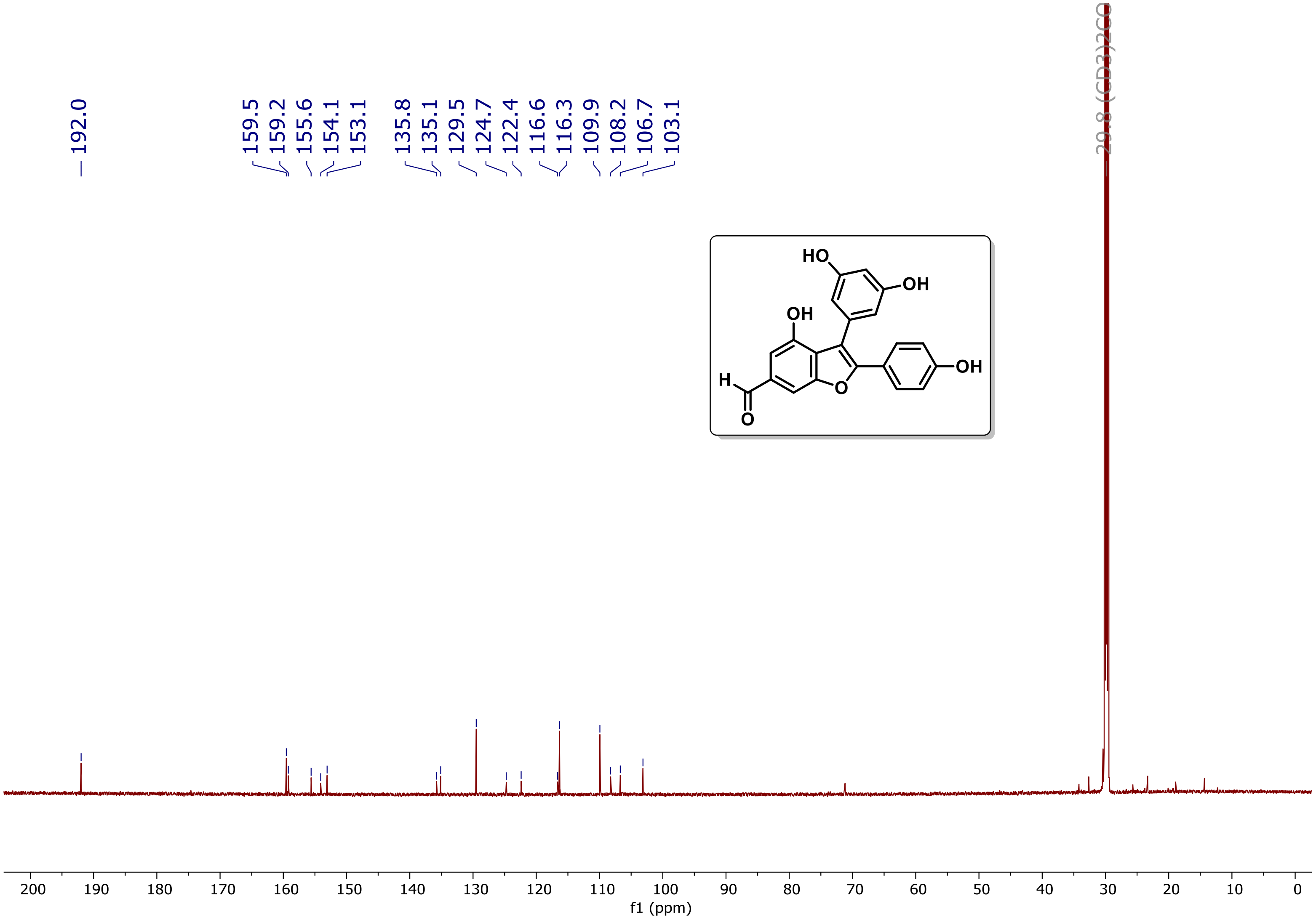

^{1}H NMR ($CDCl_3$, 300 MHz) of **S19**.

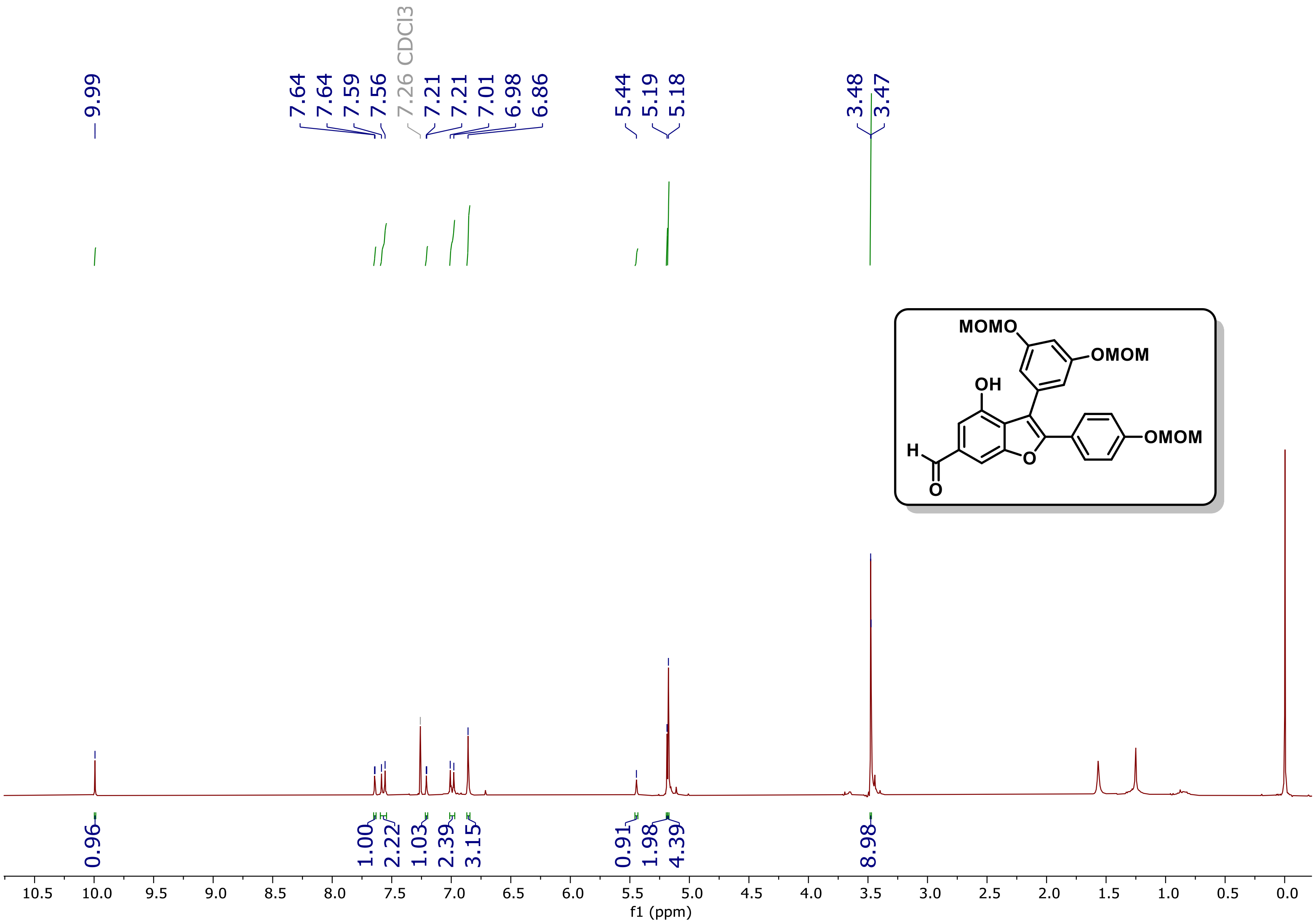

$^{13}C\{^{1}H\}$ NMR ($CDCl_3$, 75 MHz) of **S19**.

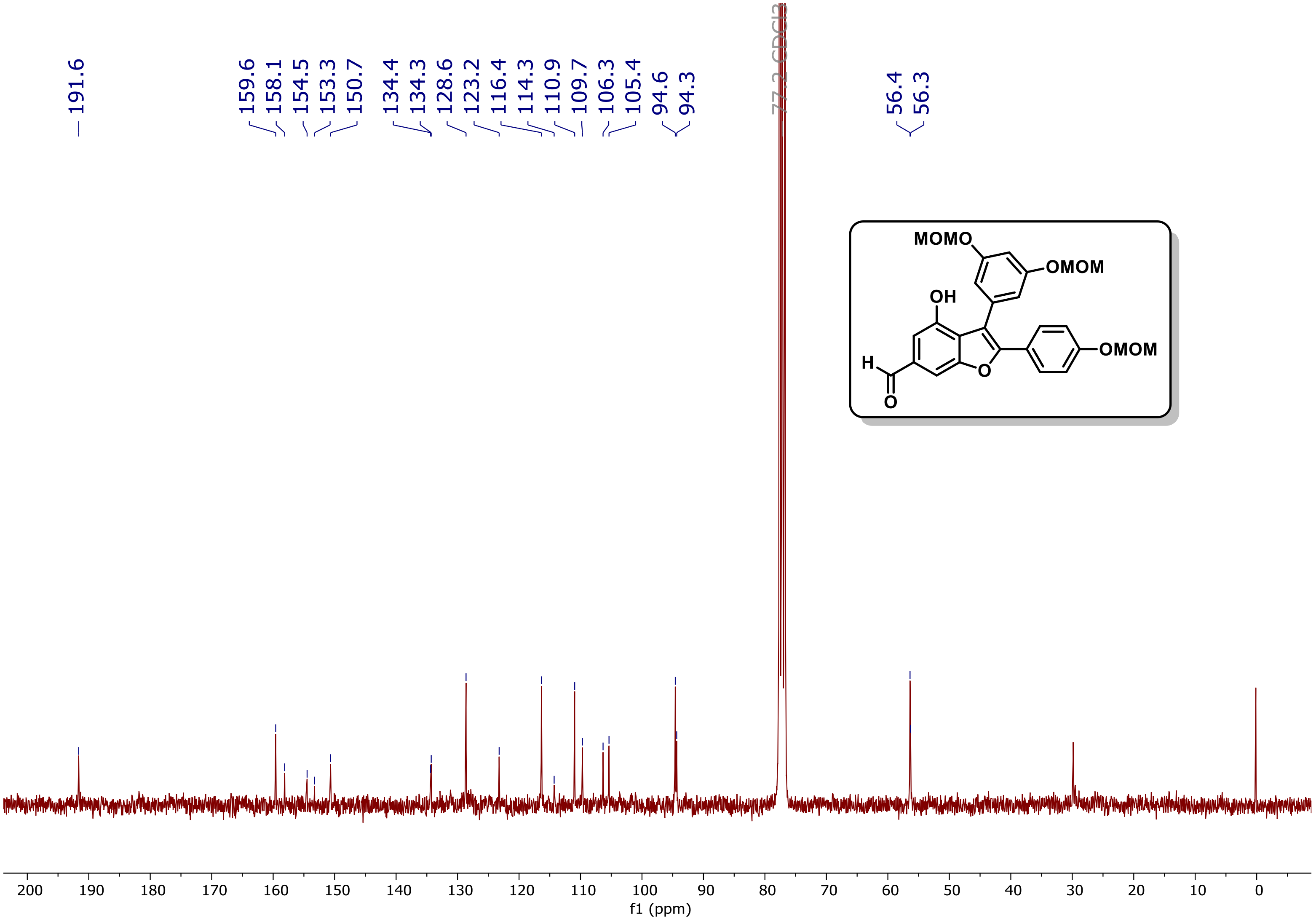

Supplement: Supplementary file 1 [file ol5c05397_si_002.pdf]
